# Inheritance Beyond Plain Heritability: Variance-Controlling Genes in *Arabidopsis thaliana*

**DOI:** 10.1371/journal.pgen.1002839

**Published:** 2012-08-02

**Authors:** Xia Shen, Mats Pettersson, Lars Rönnegård, Örjan Carlborg

**Affiliations:** 1 Department of Cell and Molecular Biology, Uppsala University, Uppsala, Sweden; 2 Statistics Unit, School of Technology and Business Studies, Dalarna University, Borlänge, Sweden; 3 Department of Animal Breeding and Genetics, Swedish University of Agricultural Sciences, Uppsala, Sweden; Stanford University School of Medicine, United States of America

## Abstract

The phenotypic effect of a gene is normally described by the mean-difference between alternative genotypes. A gene may, however, also influence the phenotype by causing a difference in variance between genotypes. Here, we reanalyze a publicly available *Arabidopsis thaliana* dataset [1] and show that genetic variance heterogeneity appears to be as common as normal additive effects on a genomewide scale. The study also develops theory to estimate the contributions of variance differences between genotypes to the phenotypic variance, and this is used to show that individual loci can explain more than 20% of the phenotypic variance. Two well-studied systems, cellular control of molybdenum level by the ion-transporter *MOT1* and flowering-time regulation by the *FRI-FLC* expression network, and a novel association for *Leaf serration* are used to illustrate the contribution of major individual loci, expression pathways, and gene-by-environment interactions to the genetic variance heterogeneity.

## Author Summary

The most well-studied effects of genes are those leading to different phenotypic means for alternative genotypes. A less well-explored type of genetic control is that resulting in a heterogeneity in variance between genotypes. Here, we reanalyze a publicly available *Arabidopsis thaliana* GWAS dataset to detect genetic effects on the variance heterogeneity, and our results indicate that the environmental variance is under extensive genetic control by a large number of variance-controlling loci across the genome. A straightforward extension of current quantitative genetics theory was derived to estimate the contribution of genetic variance heterogeneity to the phenotypic variance for loci detected in the vGWAS. This showed that some variance-controlling loci explained more than 20% of the phenotypic variance. Genetic variance heterogeneity was detected in various biological processes, including cellular control of ion levels in the plant and regulation of flowering. Our findings indicate that further studies of genetically determined variance heterogeneity are important to further understand the extent of its biological importance. Accounting for variance-controlling loci in complex trait genetic studies is a useful way to identify previously unexplained genetic variance, dissect the genetic control of environmental variance, and gain biological insight into the genetic regulation of complex traits.

## Introduction

A central question in genetics is to understand how genetic polymorphisms in genes lead to trait variability in populations. Complex traits are determined both by genes and environmental factors. For these phenotypes, the genetic effects of allelic variability are most often described as shifts in the mean phenotype between individuals with different single- or multi-locus genotypes. These mean effects will result in both additive and non-additive genetic variance, but the main focus in most GWAS studies to date has been to detect additive effects of loci and consequently explain the contribution of individual genes to the narrow-sense heritability (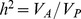). Such analyses therefore miss not only the contributions of mean effects to the non-additive genetic variance, they also ignore other types of genetic effects that influence the phenotypic variance. One such rather unexplored level of genetic control, is that of the variance, *i.e.* how allelic variants of genes regulate the amount of phenotypic variability that individuals with a particular genotype can display.

The topic of genetic variance control has been under investigation for many years in quantitative genetics, primarily motivated by its potential importance in evolutionary biology and agricultural selection programs. Both theoretical and empirical work has improved our understanding of how the genetic regulation of the environmental variance can contribute to observations of fluctuating asymmetry, canalization and genetic robustness [2], [3]. More recent empirical work support the principal idea that genetic control over variation is an inherent feature of biological networks and genes are therefore expected to exhibit control over the environmental variance (see *e.g.* [4] for a review). Further studies have also provided insights to how genetic variance-control contributes to *e.g.* capacitation [5], [6] and maintenance of developmental homeostasis [7].

Already in the mid 1980s it was observed that it was possible to identify QTL with effects the variance, rather than the mean [8]. It is, however, only recently that the topic of mapping of variance-controlling loci contributing to e.g. environmental plasticity [7], canalization [9], developmental stability [10] and natural variation in stochastic noise [11] have started to receive more attention. Although these first reports illustrate the usefulness of this approach, we still know very little about how common the variance-controlling genes are in the genome and how large total contributions they make to trait variation in populations [3], [12]. More studies are thus needed and several newly described statistical methods will facilitate detection of variance-controlling loci, and likely also G
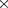
G and G
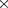
E interactions [13], in both future QTL [11], [12], [14] and GWAS [15], [16] studies.

In this study, we perform a *variance-heterogeneity GWAS*, or *vGWAS* for short, in a publicly available *Arabidopsis thaliana* dataset [1] to identify novel variance-controlling loci that illustrate the biological impact of genetic variance heterogeneity. Our study shows that clear signals from a vGWAS can be obtained using a relatively small, but well-designed, *Arabidopsis thaliana* population without requiring measurements of within-line variation. The study also includes an extension of the available quantitative genetics theory to estimate contributions of variance differences between genotypes to trait variation by individual loci. The vGWAS approach facilitates detection of loci that are involved in the genetic control of environmental variation (as discussed above). It also allows mapping of loci where incomplete LD between the causal polymorphism and the tested marker, multiple functional alleles, gene-gene or gene-by-environment interactions leads to a heterogeneity in variance, rather than a mean difference, between the genotypes [13], [14].

## Results

### Genetically regulated variance heterogeneity in *Arabidopsis thaliana*

We re-analyzed a publicly available *Arabidopsis thaliana* dataset [1]. The dataset contained 199 phenotyped ecotypes, for most of which 107 phenotypes were measured. The phenotypes were classified as either flowering (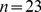), developmental (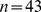), defense (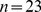) or ionomics (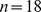) traits. All accessions were genotyped using a 250K SNP chip, resulting in 216,130 SNPs that passed quality control for use in the GWAS (http://arabidopsis.usc.edu). The original GWAS [1] reported signals in several annotated candidate genes across the genome and, in contrast to most results from human association studies, many common alleles were identified to be associated with the studied phenotypes, although the population stratification present in the dataset will affect the interpretation of the findings. The overlap between *a-priori* candidates and the detected association signals was argued to be a useful validation of the GWAS strategy in *Arabidopsis thaliana*.

Here, we performed a vGWAS for the 83, of the 107, measured traits that were quantitative using a Brown-Forsythe test (Table A1 in Text S1). This test is based on an ANOVA of the absolute deviation from the median and test for population-wide between-genotype variance heterogeneity at each evaluated marker (for more details see Methods) and does not account for potential within genotype variance heterogeneity between repeated measurements in the same inbred line. The impact of population stratification was evaluated by comparing the distribution of the genome-wide 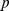-values observed in the vGWAS to their theoretical expectation. The inflation factor for the observed 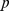-values (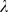) was calculated (see Methods) and found to vary for the traits (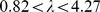). Although this differential inflation across traits might initially seem surprising, as the genomic relationship at the DNA level is identical in all analyses, the observation that the highest inflation-factors were observed for traits that are most likely to have been under selection for local adaption might explain why the analyses of those traits are most affected by population stratification. We decided to report vGWAS results for traits with high overall inflation of 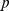-values (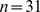, 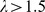) in Text S1 only and not discuss them further in this report. For the other traits, conservative significance thresholds were obtained by using Bonferroni correction for multiple-testing and using genomic control (GC) to correct for genomic inflation (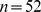, 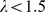). SNPs with a minor allele frequency (MAF) less than 10% were removed. No correction for testing of multiple traits was used. This procedure filtered out many traits and signals, leaving two strongly evidenced variance-controlling loci (Table 1). The conservative strategy is not recommended in studies aiming at a comprehensive exploration of the genetic architecture of a complex trait; for example only two loci of all reported in the original GWAS analysis of this dataset [1] would have met these criteria.

**Table 1:** Significant variance-controlling loci in *Arabidopsis thaliana*.

Locus		Chr	Trait^a^	Type	Alleles/MAF^b^	Dist^c^ (bp)	Mean part		Variance part	
Name	ID						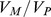 ( 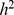 )	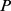 d	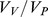	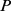
*VS*	AT1G32920	1	Leaf Serr 16	Developmental	A/G/0.14	16 226	0.7%	0.350	7.2%	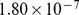
*MOT1*	AT2G25680	2	Molybdenum	Ionomics	C/G/0.31	0	4.6%	0.348	22.8%	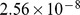

a Traits with *p*-value inflation 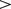
1.5 and SNPs with MAF 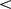
10% were excluded, and significance was determined by a Bonferroni corrected significant threshold with genomic control;

b MAF (Minor Allele Frequency = 1 - LAF (Low-variance Allele Frequency);

c Dist: Distance. A positive/negative distance value indicates that the leading SNP is to the right/left of the gene with the given ID;

d The *p*-values are from GWA (Wilcoxon) and vGWA (Brown-Forsythe) scans after genomic control.

**Figure 1:**
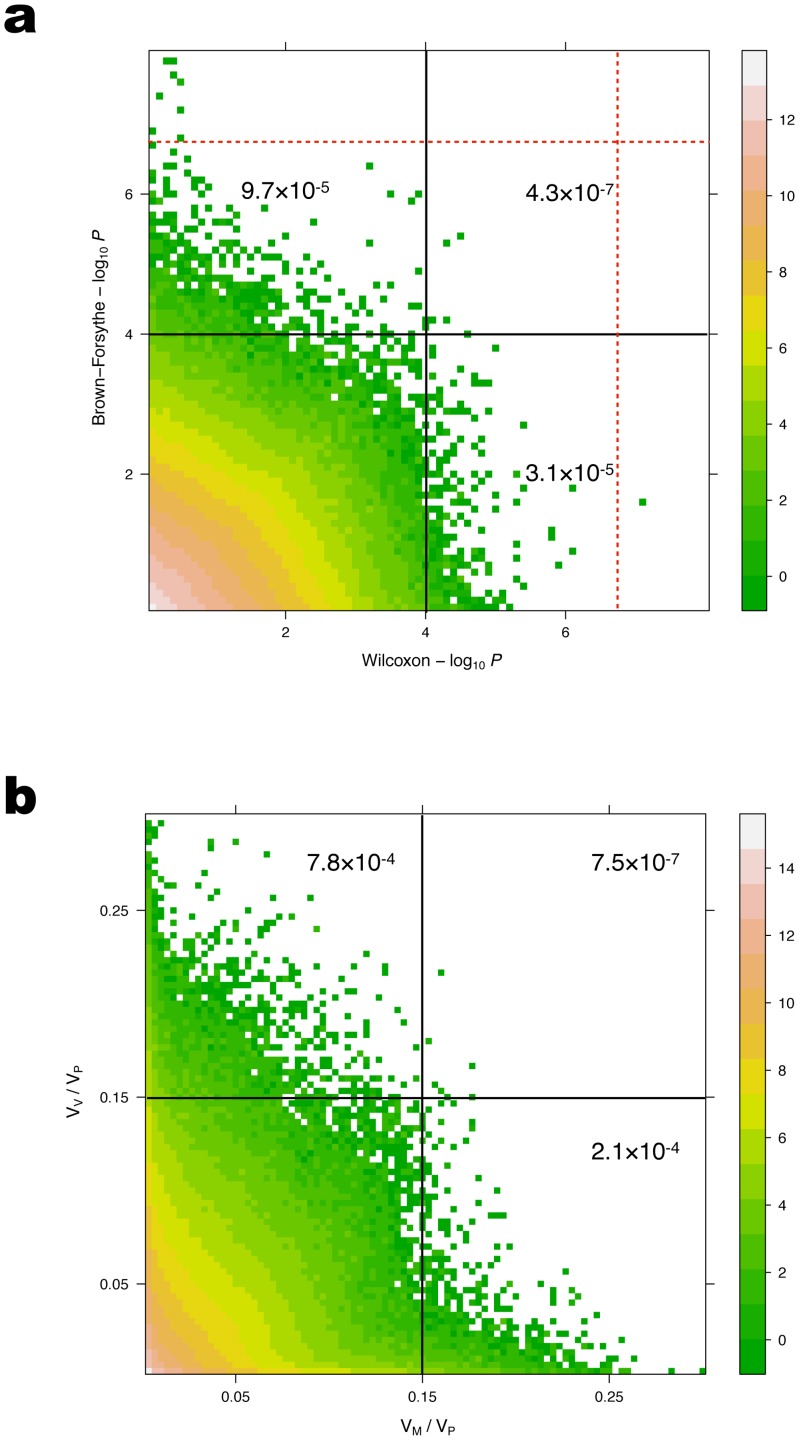
Comparison of *p*-values (a) and proportions of the phenotypic variance explained (b) for loci detected in the GWAS and vGWAS. Wilcoxon and Brown-Forsythe tests were applied for the GWAS and vGWAS analyses, respectively. Plotted GC-corrected p-values are for the association of all SNPs with MAF 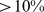 for all the quantitative traits with 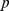-value inflation 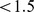. The red dashed lines indicate the Bonferroni-corrected significance threshold. The scatterplots are heat maps for the logarithm of the number of dots in each mesh cell. A sub genome-wide significance threshold of 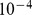 is marked in (a), and a cutoff of 15% is marked in (b). The value in each block shows the ratio of the number of points in the block to the total number of points in the subfigure.

To compare the genome-wide distributions for the 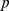-values obtained in the vGWAS and the GWAS, we subjected the results for the Wilcoxon-based GWAS results to the same conservative significance testing strategy employed in the vGWAS. The GC- and Bonferroni corrected 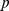-values from the two analyses showed little correlation overall (Figure 1a) and no overlap among the genome-wide significant loci. Even at sub-GWAS levels of significance (Figure 1a), there is little overlap among the loci detected in the two analyses. Using a sub genome wide significance-threshold of 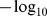
(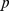-value)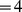, there are approximately three times as many significant SNPs in the vGWAS as in the GWAS and only about 3 out of 1000 significant SNPs reach this level of significance in both analyses. This indicates that by using a vGWAS, one will identify a novel set of loci affecting primarily the variance heterogeneity and that neither the GWAS nor the vGWAS will identify the loci with intermediate effects on both the mean and the variance heterogeneity. The results, however, also indicate that there are also a number of loci that will not be significant in either of these analyses, but that might be significant when simultaneously considering the effects on the mean and the variance (**Figure 1a**). Also, a large number of the loci that are significant in one analysis will also have effects on the other variance component, although not on a genome wide level. The potential importance of such earlier undiscovered effects for loci detected in the original GWAS [1] will be discussed in more detail later.

### Genetic variance heterogeneity can account for a considerable amount of unexplained residual variance

To estimate the contribution of genetic variance heterogeneity between genotypes to the phenotypic variance, the following model can be used:
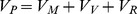
where 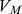 is the variance due to genetic effects on the mean, 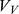 is the variance due to heterogeneity between genotypes and 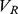 is the remaining environmental variance [3], [17]–[20]. In the population analyzed here, where only the two homozygous genotypes exist, 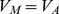 and consequently:
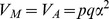
The contribution of the genetic variance heterogeneity is:
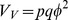
where 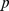 and 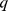 are the frequencies for the low- and high-variance alleles (LAF and HAF) and 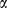 and 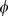 are the differences in the mean/standard deviation between the two homozygous genotypes (see the Methods and Text S1 for more information). This straight-forward single locus extension of available quantitative genetics theory facilitate mapping of individual variance-controlling loci and estimation of their contribution to the phenotypic variance. In Table 1 we give the estimates of 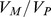 and 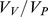 for the two most significant loci in the vGWAS. Using *MOT1* as an example, 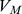, 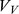 and 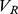 are calculated as:
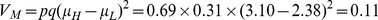


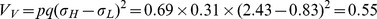


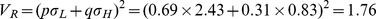
so that we have
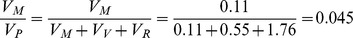


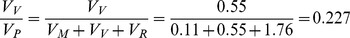
which are the same as given in **Table 1** (ignoring small rounding errors). For some loci, the genetic variance heterogeneity can thus explain a considerably larger proportion of the phenotypic variance than the genetic effect on the mean.

In **Figure 1b** we plot 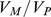 and 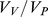 for all the genome-wide evaluated loci across the 52 traits with inflation factor 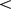
1.5. There is no overlap among the genome-wide significant loci and, as discussed above, there is little overlap even at sub genome-wide significance levels. Many loci thus have significant effects only on the mean (significant and large 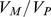 and non-significant and small 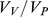) or the variance (large and significant 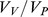 and small and non-significant 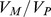). **Figure 1a** and **Figure 1b**, however, indicate that a number of loci make substantial contributions to the phenotypic variance if considering mean- and variance effects jointly. By mapping loci that display a variance heterogeneity between genotypes, and by also including 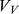 in the decomposition of the phenotypic variance for the loci significant in the standard GWAS, it is possible to detect new loci, account for non-additive genetic variance and genetically dissect the environmental variance. In this way genetic effects that was previously part of the residual variation can be accounted for and more of the total phenotypic variance be explained (**Table 1** **;** **Figure 1b**).

### The power and false positive rate for the vGWAS

**Figure 2:**
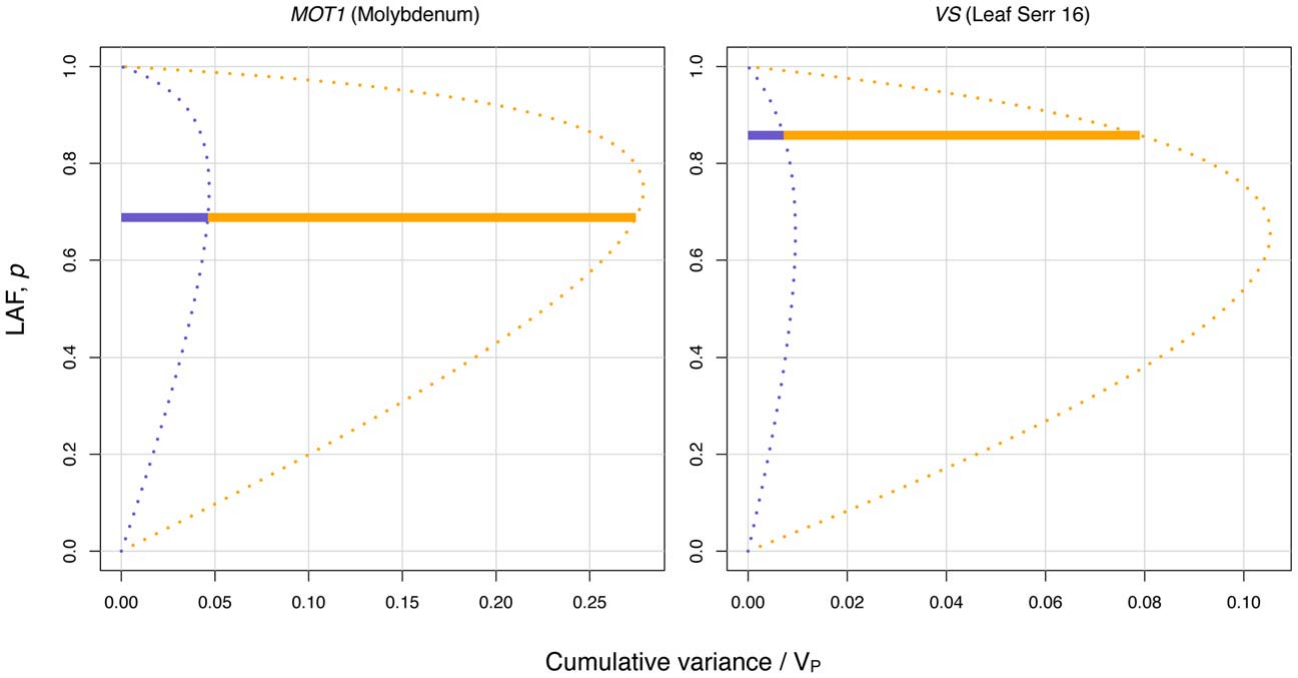
Dissection of the variance for the two most significant variance-controlling loci. The variance due to mean shift (additive variance) and variance heterogeneity are shown in blue and yellow, respectively. The cumulative bar for each locus shows the contributions of the two components of the variance at the observed low-variance allele frequency (LAF). The dotted curves illustrate the change in the variance partitioning as LAF changes.

Our proposed vGWAS strategy is based on the Brown-Forsythe test and we show empirically, and through simulations, that it is powerful while still controlling the false-positive rate: The power of the vGWAS is influenced by 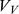 (**Figure A3** in **Text S1**) and by the low-variance allele frequency (LAF). 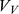 has its maximum at LAF 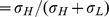, where 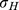 and 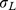 are the phenotypic standard deviations for the high- and low-variance genotypes, respectively (see **Methods** and also **Figure A2** in **Text S1**). Given this, it is not surprising that the most significant variance-controlling loci in the vGWAS have high LAF (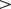
0.5) as well as large 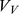 (**Figure 2** **,** **Table 1**). The false positive rate (FPR) of the vGWAS is very low for any sample size and LAF, as shown by simulations (**Figure A3** and **A4** in **Text S1**), which supports the theoretical expectation of a low false positive rate for the Brown-Forsythe test in a vGWAS [16] and that GC is useful for filtering out false positive signals due to population confounding [21].

### vSNPs are enriched in candidate genes

Atwell*et al.* [1] introduced a method for evaluating the enrichment of strong, but not necessarily genome-wide significant, signals for SNPs in candidate genes. An enrichment of such signals indicates that the analysis identifies true signals rather than random noise. Here, we extended this analysis by combining the rank-order lists from the Wilcoxon- and EMMA [22], [23] GWAS analyses performed by Atwell *et al.* [1] with the results from our vGWAS. In this combined rank-order list, where for each trait the highest rank for the listed genes in the GWAS or vGWAS was included, the average rank of the candidate genes listed by Atwell *et al.* [1] improved considerably. For the traits with inflation factor 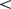
1.5, the ranks of 31 (5.1%) of the listed candidate genes were improved by introducing the vGWAS results and on average their rank increased by 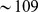 (from 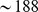 to 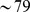; the complete results are available in **Table 1** **–83** in **Text S1**). The vGWAS signals are thus more frequent in regions of known candidate genes and the two most significant signals in our vGWAS both map to candidate genes listed by Atwell *et al.* [1] (**Table 1<~!/emph>**).

### *MOT1* controls the variance heterogeneity for molybdenum transportation in *Arabidopsis thaliana*

**Figure 3:**
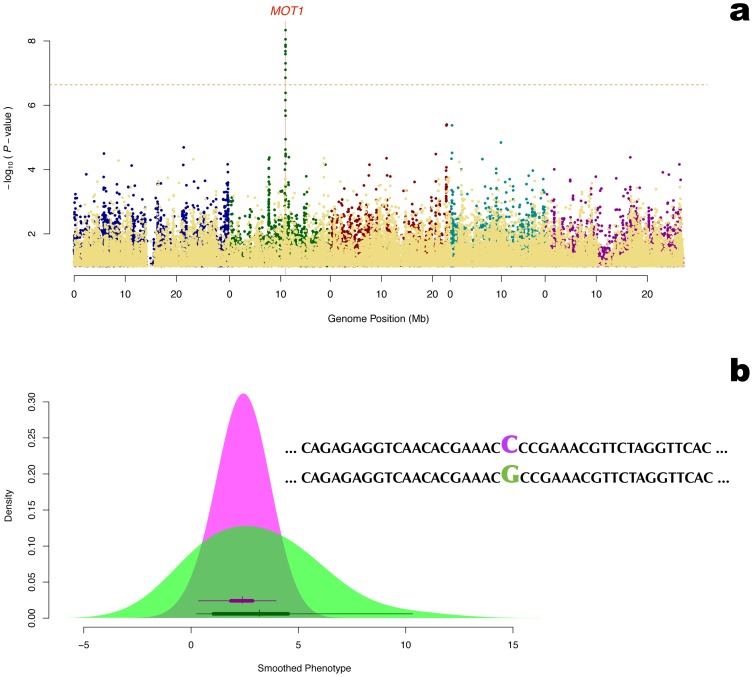
Detection of the molybdenum transporter *MOT1* as a variance-controlling gene using vGWAS. a: Manhattan plot for genetic association with the *Molybdenum concentration* in the plant. The yellow dots in the front are the genomic controlled *p*-values from the Wilcoxon (difference in mean) test. The other colored dots are the genomic controlled *p*-values from the Brown-Forsythe (difference in variance) test, where the colors are used to separate the 5 chromosomes. The horizontal dashed line corresponds to a nominal 5% significance threshold with Bonferroni correction. b: *Molybdenum concentration* distributions for the ecotypes with alternative homozygous genotypes for the only SNP typed in the exon of *MOT1*. The density curves were smoothed using a standard Gaussian kernel. The box plots in the density shades shows the real data distribution for the genotype.

Several SNPs covering the only exon of the gene *MOT1* were in the vGWAS found to be associated with the *molybdenum concentration* (**Figure 3**). *MOT1* was top-ranked in the vGWAS while originally ranked 31 in the GWAS [1] (**Table 25** in **Text S1**). The level of molybdenum in *Arabidopsis* is known to be regulated by the mitochondrial molybdenum transporter encoded by this gene [24], [25] and here *MOT1* explains 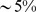 of the phenotypic variance by its effect on the mean. The effect on the variance heterogeneity between genotypes was larger (**Table 1**) and in total the locus explains 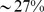 of the phenotypic variance, *i.e.* 57% rather than 10% of the earlier reported broad sense heritability for this trait [25].

Our finding that *MOT1* affects the variance heterogeneity in this population might initially seem surprising, as the original studies only report an effect on the mean. However, a closer inspection of the results by Baxter *et al.* (Figure 1B) [25] and Tomatsu *et al.* (Figure 2C) [24], indicates that variance heterogeneity between genotypes was present also in earlier studies. Using the Baxter *et al.* [25] data (http://www.ionomicshub.org), we re-estimated the differences in the mean (1.35 v.s. 0.22) and the standard deviation (0.59 v.s. 0.10) between Col-0 (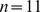) and Ler-0 (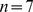) and found that both the differences in mean and variance between the genotypes are signficant (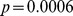 and 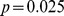 respectively). Under the assumption that the difference between Col-0 and Ler-0 is only due to the effects of *MOT1*, 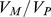 and 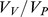 can be estimated using the formulae above to be 58.7% and 11.0%, respectively. The lower effect on the mean and higher on the variance heterogeneity in the Atwell *et al.* dataset [1] is most likely due to the different experimental designs. The earlier studies were based on comparisons between two inbred lines selected to have a large mean difference in molybdenum levels, whereas the more recent study was population-based including lines with highly variable levels of molybdenum content. As the genetic background differs between lines in the population-based studies, effects of multiple alleles and genetic interactions are more likely in the population based data. Given that other genes contribute to the difference between the selected inbred lines, we might over-estimate the mean contribution and under-estimate the variance contribution in the data from Baxter *et al.* [25] and Tomatsu *et al.* [24]. Despite this, these datasets still show evidence of genetic variance heterogeneity.

### Variance heterogeneity in *Leaf serration* is under genetic control

**Figure 4:**
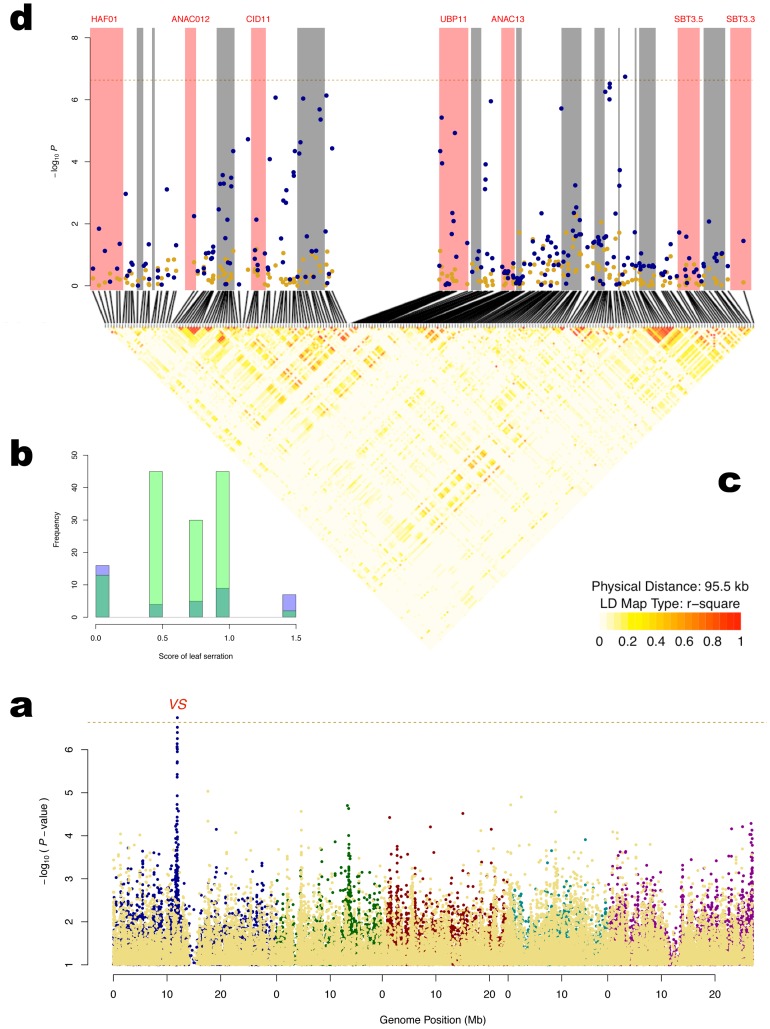
Detection of a variance-controlling locus (*Variance in Serration - VS*) affecting *Leaf serration at 16°C*. a: Manhattan plot for genetic association with *Leaf serration at 16°C* in the plant. The yellow dots in the front are the genomic controlled *p*-values from the Wilcoxon (difference in mean) test. The other colored dots are the genomic controlled *p*-values from the Brown-Forsythe (difference in variance) test, where the colors are used to separate the 5 chromosomes. The horizontal dashed line corresponds to a nominal 5% significance threshold with Bonferroni correction. b: Two overlapping histograms showing the phenotypic distributions per genotype of the *VS* locus. c: The distribution of LD across the *VS* locus. d: Association signals around the *VS* locus where the annotated genes are illustrated by shades in red (gene-name available) or gray (gene-name unavailable).

A novel locus affecting *Leaf serration* at 16°C was identified on chromosome 1 (**Table 1** **;** **Figure 4a**). The genetic variance heterogeneity at this locus is due to a shift in the phenotypic distribution from normal to uniform (**Figure 4b**). The locus is close to the suggested candidate gene *ANAC13* [1]. Earlier studies have described similar effects on the phenotypic variance when disruptive mutations lead to a loss of control in a developmental pathway, leading to an unregulated system displaying a random (uniform) occurrence of the phenotype [26]–[28]. A closer inspection of the vGWAS evidenced region (**Figure 4** **c,d**), however, shows that the signal is very low in the coding region of *ANAC13* and also that the coding region is in low LD with the SNPs that display the strongest association signals. This makes it less likely that the causative mutation leading to the observed effect on the phenotype is located in the coding region of this gene. Further studies of this *Variation in Serration* (*VS*) locus, including e.g. the regulatory regions of *ANAC13*, are needed to identify the biological explanation for the observed effect.

### Genetic control of expression variability in the *FRI-FLC* pathway and downstream effects on variance heterogeneity in flowering

The two main variance-controlling loci detected in the vGWAS, *MOT1* and *VS*, primarily affect the variance heterogeneity between genotypes in this dataset and only have small effects on the mean. When looking beyond these two loci to explore the total contribution of the sub-vGWAS significant loci to the phenotypic variance, many of these were found to also have effects on the mean (**Figure 1**). Also, a number of the loci detected in the GWAS were indicated to also affect the variance heterogeneity. To explore this observation further, we estimated the mean and variance controlling effects for the well-studied locus *FRI* (Frigida) that had the highest significance in the standard GWAS [1]. Genetic variability in this locus is known to influence its own mean expression level [1] and through effects on downstream loci influence flowering as well. Here, we found that this locus also had a significant effect on the genetic variance heterogeneity between the alternative *FRI*-genotypes (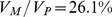, 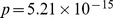, and 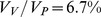, 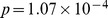) for the trait *FRI Expression*.

**Figure 5:**
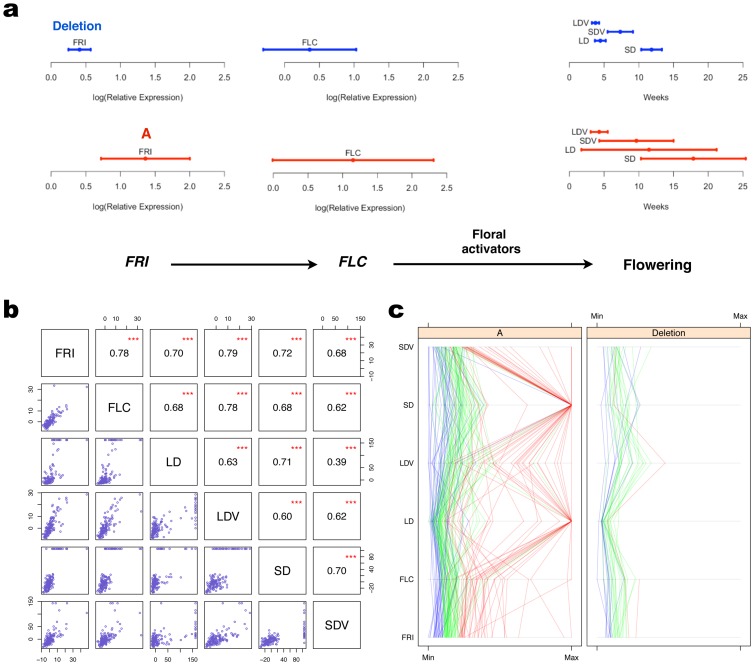
Propagation of variance heterogeneity in the *FRI*-*FLC* pathway. a: The horizontal bars show the variance in *FRI*-expression, *FLC*-expression and flowering times under four different conditions (short (SD)/long (LD) days, with (V) or without (−) vernalization) as the phenotypic mean +/− 1 S.D. within the two alternative genotypes at the *FRI*-locus. *Deletion* represents homozygosity for the loss of function allele and *A* homozygosity for the wild-type allele. The mean and the variance are significantly larger for all traits among the inbred lines with the functional *A* genotype (Table 2). The pathway is adapted from [37]. b: Scatterplots and Spearman's rank correlation coefficients are given for the deviation of *FRI*, *FLC*, and the flowering traits in (a) from the median of each phenotype. *** indicates that the corresponding correlation coefficient is significantly different from zero with 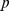-value less than 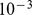. (c): For all the phenotypes in the pathway, the values for individuals are connected by lines. The color of the line for an individual is assigned based on its level of *FRI* expression. Individuals with *FRI* expression below the 25% quantile are in blue, between the 25% and 75% quantiles in green, and above the 75% quantile in red.

It is known that the expression of *FRI* influences flowering by inducing expression of *Flowering Locus C* (*FLC*), which in turn delays flowering (**Figure 5**) [29], [30]. Here, we observe a variance heterogeneity between *FRI* genotypes that is not only present for *FRI* expression, but also for the other traits downstream in this pathway, *i.e. FLC* expression and several flowering traits (**Figure 5a** **;** **Table 2**).

**Table 2:** Differences in mean and variance for traits in the *FRI*-*FLC*-flowering pathway.

		*FRI* genotype					
Trait^a^	Significance	A		Deletion		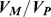 f	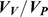 g
	mean^b^/variance^c^	mean 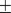 s.d.^d^	CV^e^	mean 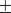 s.d.	CV		
*FRI* expression	***/***	1.36 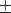 0.64	0.47	0.41 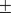 0.16	0.40	26.1%	6.7%
*FLC* expression	***/**	1.15 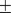 1.16	1.01	0.36 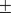 0.67	1.87	7.0%	2.7%
LD	***/***	80.3 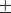 68.2	0.85	31.2 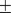 5.5	0.18	8.0%	13.0%
LDV	n.s./**	30.1 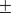 8.6	0.29	26.3 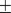 3.7	0.14	3.0%	5.2%
SD	***/***	125.9 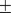 52.8	0.42	82.9 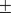 10.4	0.13	10.2%	9.9%
SDV	*/*	67.6 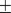 37.5	0.55	51.3 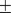 12.8	0.25	3.1%	7.0%

a Flowering-times LD (Long days), SD (Short days), V (Vernalization); ***/**/*/n.s.: 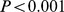/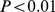/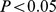/non-significant in significance test for.

b difference in mean (Wilcoxon) and

c difference in variance (Brown-Forsythe) between *FRI*-genotypes;

d s.d.: Phenotypic standard deviation;

e CV: Coefficient of variation;

f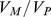: Proportion of the phenotypic variance (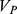) due to the mean-controlling effect of *FRI* on the trait;

g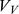: Proportion of the phenotypic variance due to the variance-controlling effect of *FRI* on the trait.

In biology, it is often observed that the phenotypic variance increases with the mean trait value. The mean shift is commonly thought to be of primary functional importance and the change in the variance a by-product of altering the mean. Adaption is, however, driven by selection of individuals based on their phenotype and consequently both the mean and the variance will affect this process. If the increase in the variance is not under genetic control, it will not be able to contribute to adaption and merely increase the noise in the phenotype and decrease the efficiency in selection. If the the heterogeneity in variance on the other hand is under genetic control, it might be selected for and potentially be of adaptive value. It is therefore of interest to understand the biological mechanisms leading to variance heterogeneity between genotypes and how such effects might impact the phenotype under selection. One example of where genetic control of the environmental variance might be of adaptive value is for variation in flowering time [31]. Under selection in a stable environment, the optimum time to flower will be relatively constant across years, suggesting a fitness advantage for alleles decreasing the variability in flowering time for its offspring. In a fluctuating environment, however, high-variance alleles are potentially more adaptive as offspring will flower over a broader time period, allowing a fraction of the offspring to reproduce every season.

Here we observe a variance heterogeneity between *FRI* genotypes in the downstream phenotypes in the *FRI*-*FLC* pathway. Is there then also a functional propagation of the differential variance in *FRI* expression through the downstream pathway? Or is this the result of a mere increase in the stochastic noise? If there is a quantitative, rather than threshold, transmission of signals through the pathway, one could expect that the quantitative differences among individuals in *FRI* levels would result in quantitative differences also in *FLC* expression, resulting in a potentially *FRI* driven adaptive variation in flowering. Such a functional propagation through the pathway would result in a phenotypic correlation between the individuals for the phenotypes in the pathway, i.e. individuals for whom the levels of *FRI* deviate most from the mean would also be those where the deviations were the highest in *FLC* and flowering. The available data supports such a transmission of effects, as there are moderate to high correlations between the deviations from the trait median in the pathway (**Figure 5b**). Furthermore, there is also a clear relationship between the trait values throughout the pathway for individual accessions (**Figure 5c**), where *FRI* expression levels are strongly associated with high *FLC* expression and late flowering. Interestingly, other empirical data also indicate that variance heterogeneity in the *FRI*-*FLC* pathway might be of adaptive advantage. The low-variance (loss of function) *FRI* allele has appeared and remained multiple times in natural populations [32] without replacing the wild-type high-variance allele globally, suggesting that the alternative alleles have fitness advantages in different environments.

### Genetic variance heterogeneity in flowering-time due to gene-by-environment interactions involving the *FRI* locus

**Figure 6:**
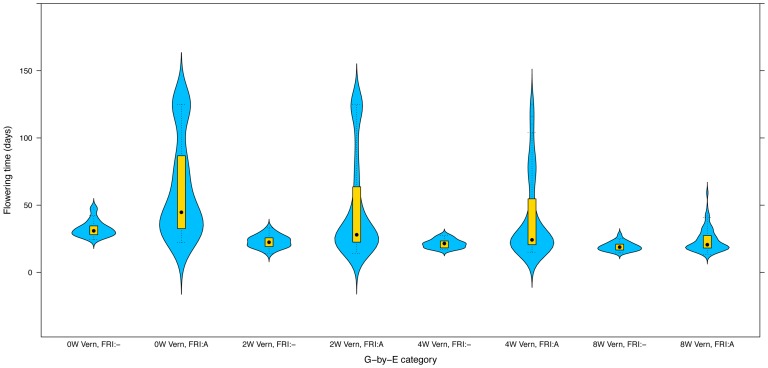
The effect of the *FRI* 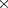 Vernalization interaction on flowering-time in *Arabidopsis thaliana* **.** The columns show the phenotypic distributions for each *FRI* genotype x Vernalization combination. *FRI*:- represents the loss of function genotype and *FRI*:A the wild-type genotype. Flowering-time was measured under long days without (0W)/with 2 (2W)/4 (4W)/8 (8W) weeks of vernalization.

*FRI* plays a central role in the vernalization response in *Arabidopsis thaliana*, where dominant alleles at this locus acts to confer late flowering, which is reverted to earliness by vernalization. Here, we find a gene-by-environment interaction effect between *FRI* and vernalization on both the mean and variance in flowering-time (**Figure 6**). *FRI* shifts the mean flowering time and the degree of variance heterogeneity both in the presence and absence of vernalization. The genetic effect of the wild-type *FRI* genotype on the variance heterogeneity is, however, much more dependent on the level of vernalization than the effect of the non-functional genotype. The observed genetic variance heterogeneity is not a mere general increase in the dispersion, but rather the appearance of very late flowering among a smaller number of accessions with the wildtype *FRI* genotype when there is less vernalization (see also **Figure 5c**). In the absence of vernalization, a bi-modal phenotypic distribution appears, indicating an underlying strong interaction between the *FRI*-genotype, vernalization and at least one more locus or environmental factor.

## Discussion

We have validated the vGWAS strategy used in this study by simulations and shown that it controls the false-positive rate well. To avoid any potential strong influence of the population structure, we focus our discussion on results for traits with lower 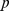-value inflation (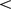
1.5) and also applied GC [15]. Our further analyses of the obtained results, including the thorough investigation of the most significant loci in the vGWAS and the enrichment analysis of *a priori* candidate genes indicate that the analysis provides results of biological significance. Further studies are needed to explore the extent of genetic variance heterogeneity in the genetic architecture of other populations and traits as well as to develop methods for accounting for more severe effects of population structure. Our results, however, strongly indicate that the vGWAS is a promising approach for analyzing genome-wide association data.

Several earlier QTL studies have shown that it is possible to map loci that control the environmental variance of quantitative traits and identify plausible candidate loci for these effects [9]–[11], [33]. These results are thus in line with what was shown in this report. Previous applications of vGWAS in human populations [15], [16], have, however, only found weak signals. The reason for this might be that human GWAS datasets normally contain noisy phenotypic measurements on many genotypes (individuals), whereas this and other datasets from experimental populations contain phenotypic measurements with less non-systematic environmental noise on fewer genotypes. Also, as all inbred lines in this study were grown in the same environment for each phenotype measured, phenotypic plasticity had no effect on the single phenotypes in the study, which removes this as a potential cause for variance heterogeneity between genotypes [34]. The low non-systematic environmental noise, absence of effects from phenotypic plasticity and perhaps also an increased sensitivity of homozygous lines to environmental variation (see [7] and references therein), thus makes the current design a better choice for mapping and exploration of genetic variance heterogeneity.

We have illustrated the biological impact of genetic variance heterogeneity using three examples. *MOT1* illustrates how an individual gene can explain a large fraction of the phenotypic variance by its genetic effect on the variance heterogeneity. The *VS* locus illustrates the potential of the vGWAS to identify loci underlying developmental stability, where the disruptions are likely to cause a random occurrence, rather than a directional shift, in the phenotype [26]–[28]. The *FRI*-*FLC* pathway is a well-studied system in *Arabidopsis thaliana*, and here we indicate that this pathway might not only regulate the average flowering time, but also the heterogeneity in flowering times. This through a potential propagation of genetic heterogeneity in gene-expression through the pathway and a gene-by-environment interaction leading to a differential variance heterogeneity in flowering times depending on the *FRI* genotype and the extent of vernalization.

The dominant paradigm in current GWAS analyses is to identify additive loci through their effect on the mean difference between genotypes. The total contribution of the detected additive loci to the narrow-sense heritability is then estimated as the sum of their individual effects. The discrepancy between the estimates of the heritability for the studied trait in the population and the sum of the effects of the loci detected in the GWAS is often referred to as the “missing heritability”. As this discrepancy appears to be large, even when large populations are analyzed, there has been an intense discussion regarding the potential mechanisms underlying this. The observation has also increased the interest in exploring alternative approaches to analyze GWAS data. Identifying loci contributing to the genetic control of the environmental variation will allow us to better explain the genetic contribution to the phenotypic variation, but not the narrow-sense heritability. Some loci detected in the vGWAS might, however, be involved in gene-gene or gene-environment interactions, result from an incomplete LD between the causal polymorphism and the tested marker, as well as contain multiple functional alleles. In such situations, the loci might make contributions to the narrow-sense heritability that are difficult to detect using a standard GWAS [14]–[16].

By accounting for genetic variance heterogeneity in future analyses of GWAS data, we foresee that more genes that contribute to the phenotypic variation through non-additive genetic effects on the mean and genetic regulation of the environmental variation can be mapped and functionally dissected. Consequently, the vGWAS will allow genetic analysis to proceed beyond the current GWAS paradigm, dissect the genetic regulation of the environmental variance and potentially also detect loci contributing to the currently unexplained genetic variance. The discussion in the field of quantitative genetics regarding the potential importance of genetic heterogeneity between genotypes have historical roots [35]. The results reported here provides insight to the genome-wide effects of variance heterogeneity and show that the genome contain many loci that contribute to the phenotypic variance through a genetic control of the variance heterogeneity.

Earlier studies on the genetic control of robustness in gene-expression indicates that it, at least to some extent, is under genetic control by individual loci with measurable effects [12]. Our finding that genetic variance heterogeneity might also be propagated in gene-expression pathways could have further functional implications for studies of the regulation of gene-expression. Studies are therefore needed to explore whether the extent of regulatory control over variance heterogeneity in expression pathways is of functional importance. If this regulation proves to be important, it adds a new dimension to the complexity in regulatory models. Such studies of the propagation of regulatory effects on the variability of expression could *e.g.* be performed by mapping of *cis*-regulated variance-controlling loci in genetical genomic studies followed by subsequent identification of downstream variance heterogeneity in known pathways, or by searching for co-expression on the level of variance in traditional microarray experiments. It will be interesting to see if this new way of dissecting the regulatory control in the transcriptome, using data that is already publicly available for many species, could provide a new handle on this topic.

## Methods

### Dissection of the phenotypic variance accounting for genetic variance heterogeneity

In a single-locus additive model, the phenotypic variance 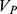 is partitioned as
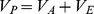
here 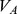 is the additive variance, and 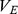 is the residual variance.

This model only accounts for effects of genes on the mean difference between genotypes. For a single locus, we instead suggest to dissect the phenotypic variance into the variance due to the mean shift between genotypes, 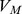, the variance due to the variance heterogeneity, 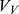, and the remaining residual variance 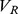, *i.e.*

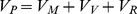
Since inbred lines are analyzed in this paper, there is no dominance, and consequently 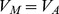. We therefore have 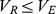, where equality holds if and only if 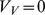, *i.e.* 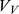 captures a part of 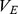 that is not stochastic noise, but actually contributions by genetics.

Several alternative quantitative genetics models have been proposed for modeling the genetic effect on the environmental variance (see e.g. [3] for a review). Here, we review and use the well-established quantitative genetics estimation equation for 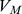 and also explicitly derive the proportion of 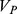 due to variance heterogeneity, 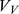, for a single locus in this quantitative genetics framework. This is to clearly present and investigate the properties of these quantities when applied in a vGWAS context (For details on the derivations, see **Text S1**). 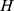/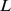 here denotes the high/low-variance allele (HA/LA), respectively. Our quantitative derivation resembles the “standard deviation model” in [3], which assumes an additive model for the standard deviation per genotype.



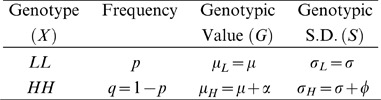




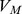
 is here the phenotypic variance explained by 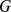 and identical to 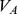. From basic probability theory, we have
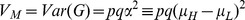
Similarly, 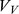 is measured as the variance of 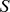,
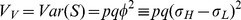
The total variance of the phenotype is
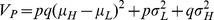
where 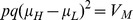, and 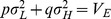 is the mean environmental variance. 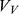 is a part of 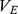, and the remaining residual variance is
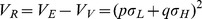
The proportion of 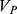 due to variance heterogeneity is thus
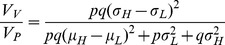
We investigated properties of the above quantities in detail (see **Text S1**), and it is worth noting that both the narrow sense heritability 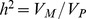 and 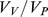 are maximized when
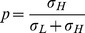
which is 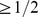 (see *e.g.* **Figure 2**). Only when no variance heterogeneity exists, 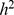 is maximized at 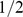.

### Screening the genome for variance-controlling loci—the vGWAS

For testing variance-controlling SNPs in the vGWAS, we use the Brown-Forsythe (Levene) test. The Brown-Forsythe is a statistical test for the equality of group variances and is based on an ANOVA of the absolute deviation from the median. It has earlier been shown to be robust to deviations from normality of the phenotypic distribution in GWAS applications [16]. If the phenotypic value is 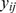 for individual 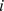 with genotype 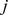, where 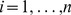, and 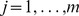, the absolute deviations from the median of each genotype are
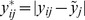
where 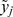 is the median of the phenotypic values of the individuals that have genotype 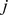. Performing a one-way ANOVA on 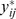, we have the ANOVA 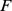 statistic
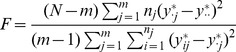
where 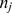 is the number of observations in group 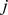. This 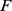 statistic follows an 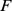 distribution with 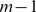, 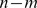 degrees of freedom. Usually, 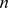 is sufficiently large to approximate the 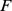 statistic as a 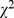 statistic with 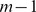 degrees of freedom. The nominal 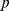-values calculated using such 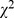-statistics are used in the vGWAS with a Bonferroni corrected significance threshold.

### Genomic control for vGWAS

In an ordinary GWAS, genomic control (GC) is used to shrink any existing inflation of the test scores (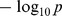-values). When testing for the single genetic effect in the GWAS, the null distribution of the test statistic for the nominal 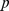-values is 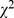 with 1 degree of freedom. Since most of the SNPs are not expected to be associated with the trait, the sample distribution of the chi-squares across the genome should resemble the null distribution. If there is inflation, the chi-squares are adjusted using 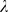, *i.e.* the inflation factor estimated by comparing the distribution of the sample 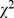
's and 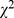 distribution with 1 degree of freedom.

As the sample size in this study is sufficient to approximate the 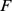-statistic of the Brown-Forsythe test using a 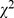 statistic, the ordinary GC methods can be applied. Here, we regress the sample 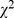
's on the null 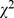
's with a zero-intercept and take the slope as an estimate of 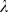, which is the approach used in the current version of the GWAS analysis package GenABEL [36]. This approach was selected as it is expected to be more conservative than, or similar to, the alternative way of estimating 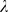 using the ratio of the observed median of 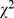
's to the theoretical median of 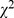 with 1 degree of freedom [21].

## Supporting Information

Text S1 Details of the genetic dissection of the analyzed traits. Table A1: Quantitative phenotypes analyzed. Figure A1: Dissection of the phenotypic variance with respect to the level of variance heterogeneity. Figure A2: Dissection of the phenotypic variance with respect to the low-variance allele frequency (LAF). Figure A3: Power of the Brown-Forsythe test with respect to the level of variance heterogeneity. Figure A4: A simulation study comparing the genome-wide p-values of vGWAS before and after genomic control (GC). Figure B1–B83: Summary of vGWAS results for the 83 analyzed quantitative traits. Table 1–83: GWAS and vGWAS scores and ranks of the 83 analyzed quantitative traits. Tutorial of the R package vGWAS together with its documentation.
(PDF)
