# Supplementary material for: Inheritance Beyond Plain Heritability: Variance-Controlling Genes in Arabidopsis thaliana
Source: PLoS Genet. 2012 Aug 2;8(8):e1002839. doi: 10.1371/journal.pgen.1002839 (PMC3410891; doi:10.1371/journal.pgen.1002839)
Supplement: Text S1 — Details of the genetic dissection of the analyzed traits. Table A1: Quantitative phenotypes analyzed. Figure A1: Dissection of the phenotypic variance with respect to the level of variance heterogeneity. Figure A2: Dissection of the phenotypic variance with respect to the low-variance allele frequency (LAF). Figure A3: Power of the Brown-Forsythe test with respect to the level of variance heterogeneity. Figure A4: A simulation study comparing the genome-wide p-values of vGWAS before and after genomic control (GC). Figure B1–B83: Summary of vGWAS results for the 83 analyzed quantitative traits. Table 1–83: GWAS and vGWAS scores and ranks of the 83 analyzed quantitative traits. Tutorial of the R package vGWAS together with its documentation. (PDF) [file pgen.1002839.s001.pdf]

SUPPORTING INFORMATION S1

This document contains the supplementary materials of the paper "*Inheritance beyond plain heritability: variance controlling genes in Arabidopsis thaliana*". There are 5 parts:

1. **Details of the quantitative variance dissection**
2. **Supplementary Tables and Figures A*** - descriptions and analytical results.
3. **Supplementary Figures B*** - figures of GWAS and vGWAS results for each studied trait. The horizontal dashed lines correspond to a nominal 5% significance threshold with Bonferroni correction.
4. **Supplementary Tables** - tables of scores and ranks from GWASs and vGWASs of the candidate genes suggested in Atwell et al. (2010) *Nature*.
5. **Tutorial** of the **R package vGWAS** together with its **documentation**.

Details of the quantitative variance dissection

In the **Methods** section of the paper, we dissect the phenotypic variance into the variance due to the mean shift between genotypes, V_M, the variance due to the variance heterogeneity, V_V, and the remaining residual variance V_R, *i.e.*

$$V_P = V_M + V_V + V_R$$

Here, we give detailed derivations of these quantities and the related properties. H/L here denotes the high/low-variance allele (HA/LA), respectively, and we assume an additive model for the standard deviation per genotype.

Genotype (X)	Frequency	Genotypic Value (G)	Genotypic S.D. (S)
LL	p	$\mu_L = \mu$	$\sigma_L = \sigma$
HH	$q = 1 - p$	$\mu_H = \mu + \alpha$	$\sigma_H = \sigma + \phi$

V_M is here the phenotypic variance explained by G and identical to V_A.

$$\begin{aligned} Var(G) &= E[G^2] - E^2[G] \\ &= p\mu^2 + q(\mu+\alpha)^2 - [p\mu + q(\mu+\alpha)]^2 \\ &= pq\alpha^2 \\ &\equiv pq(\mu_H - \mu_L)^2 \end{aligned}$$

Similarly, V_V is measured as the variance of S,

$$\begin{aligned} Var(S) &= E[S^2] - E^2[S] \\ &= p\sigma^2 + q(\sigma+\phi)^2 - [p\sigma + q(\sigma+\phi)]^2 \\ &= pq\phi^2 \\ &\equiv pq(\sigma_H - \sigma_L)^2 \end{aligned}$$

The total variance of the phenotype P can be derived as

$$\begin{aligned} Var(P) &= Var(E[P|X]) + E[Var(P|X)] \\ &= Var(G) + E[S^2] \\ &= pq\alpha^2 + p\sigma^2 + q(\sigma+\phi)^2 \\ &= pq\alpha^2 + pq\phi^2 + (\sigma + q\phi)^2 \\ &\equiv pq(\mu_H - \mu_L)^2 + pq(\sigma_H - \sigma_L)^2 + (p\sigma_L + q\sigma_H)^2 \end{aligned}$$

where $pq(\mu_H - \mu_L)^2 = V_M$, $pq(\sigma_H - \sigma_L)^2 = V_V$, and $(p\sigma_L + q\sigma_H)^2 = V_R$. The proportion of V_P due to variance heterogeneity is thus

$$\frac{V_V}{V_P} = \frac{pq\phi^2}{pq\alpha^2 + p\sigma^2 + q(\sigma+\phi)^2}$$

The mean environmental variance is $V_E = V_V + V_R = p\sigma^2 + q(\sigma+\phi)^2$. Here, we investigate important properties of V_V/V_P. V_V/V_P as a measurement of the proportion explained by the genetically controlled difference in the environmental variance, has the following essential properties:

Unit free: The value of V_V/V_P does not change with the choice of measurement unit, *i.e.* it does not change if the trait is measured in centimeters versus meters or grams versus kilograms.

Between 0 and V_E/V_P: From our derivations, we have $0 < V_V/V_P < V_E/V_P < 1$.

Bounded by H^2: The value of V_V/V_P does not exceed the broad sense heritability H^2 when the underlying cause of variance heterogeneity is an epistatic interaction (**Supplementary Figure A5**).

Has an asymptote of LAF: When the shift in variance goes to infinity, namely, $\phi \to \infty$, we have $V_V/V_P \to pq/q = p$, which is the low-variance allele frequency (LAF).

Maximized when LAF $> 1/2$: Regarding V_M/V_P and V_V/V_P as functions of p, the roots of

$$\frac{\partial}{\partial p}\frac{V_M}{V_P} = 0, \qquad 0 \leqslant p \leqslant 1$$

and

$$\frac{\partial}{\partial p}\frac{V_V}{V_P} = 0, \qquad 0 \leqslant p \leqslant 1$$

are the same, *i.e.*

$$p = \frac{\sigma+\phi}{2\sigma+\phi} \equiv \frac{\sigma_H}{\sigma_L+\sigma_H}$$

At this frequency of the low-variance allele, both V_M/V_P and V_V/V_P attain their maxima,

$$\max_p \left(\frac{V_M}{V_P}\right) = \frac{\alpha^2}{\alpha^2+(2\sigma+\phi)^2} \equiv \frac{(\mu_H-\mu_L)^2}{(\mu_H-\mu_L)^2+(\sigma_L+\sigma_H)^2}$$

and

$$\max_p \left(\frac{V_V}{V_P}\right) = \frac{\phi^2}{\alpha^2+(2\sigma+\phi)^2} \equiv \frac{(\sigma_H-\sigma_L)^2}{(\mu_H-\mu_L)^2+(\sigma_L+\sigma_H)^2}$$

If no variance heterogeneity exists, $\phi = 0$, V_M/V_P is maximized at $p = 1/2$.

Supplementary Table A1: Quantitative phenotypes analyzed.
Refer to Atwell *et al*. (2010) for further details about phenotype description and scoring.

Phenotype	ID	Type	Sample size	Description	Growth Conditions
LD	1	Flowering	167	Days to flowering time (FT) under Long Day (LD) and Short Day(SD) +/- vernalization.	18°C, 16 hrs daylight.
LDV	2	Flowering	168		18°C, 16 hrs daylight, vernalized (5 wks, 4°C)
SD	3	Flowering	162		18°C, 8 hrs daylight.
SDV	4	Flowering	159		18°C, 8 hrs daylight, vernalized (5 wks, 4°C)
FT10	5	Flowering	194	Flowering time (FT)	10°C, 16 hrs daylight.
FT16	6	Flowering	193		16°C, 16 hrs daylight.
FT22	7	Flowering	193		22°C, 16 hrs daylight.
Seed Dormancy	8	Developmental	83	Seed dormancy level.	Seeds stored in cellulose paper bags and kept in a dark incubator at 20°C, 40% relative humidity.
Lithium (Li7)	14	Ionomics	93	*In planta* ion concentration.	20°C, 16 hrs daylight.
Boron (B11)	15	Ionomics	93		
Sodium (Na23)	16	Ionomics	93		
Magnesium (Mg25)	17	Ionomics	93		
Phosphorus (P31)	18	Ionomics	93		
Sulfur (S34)	19	Ionomics	93		
Potassium (K39)	20	Ionomics	93		
Calcium (Ca43)	21	Ionomics	93		
Manganese (Mn55)	22	Ionomics	93		
Iron (Fe56)	23	Ionomics	93		
Cobalt (Co59)	24	Ionomics	93		
Nickel (Ni60)	25	Ionomics	93		
Copper (Cu65)	26	Ionomics	93		
Zinc (Zn66)	27	Ionomics	93		
Arsenic (As75)	28	Ionomics	93		
Selenium (Se82)	29	Ionomics	93		
Molybdenum (Mo98)	30	Ionomics	93		
Cadmium (Cd114)	31	Ionomics	93		

Phenotype	ID	Type	Sample size	Description	Growth Conditions
0W	39	Flowering	137	Days to FT under LD with varying vernalization.	23°C, 16 hrs daylight. Not vernalized.
2W	40	Flowering	152		23°C, 16 hrs daylight, vernalized (2 wks, 5°C, 8 hrs daylight).
4W	41	Flowering	119		23°C, 16 hrs daylight, vernalized (4 wks, 5°C, 8 hrs daylight).
8W	42	Flowering	155		23°C, 16 hrs daylight, vernalized (8 wks, 5°C, 8 hrs daylight).
FLC	43	Flowering	167	FLC and FRI gene expression.	Growth in greenhouse, ~20-22°C, 16 hrs daylight.
FRI	44	Flowering	164		
8W GH FT	45	Flowering	162	Days to FT and LN at FT.	20-22°C, natural light from the middle of October 2002 till March 2003, vernalized (8 wks, 4°C, 8 hrs daylight).
8W GH LN	46	Flowering	163		
0W GH FT	47	Flowering	153		20-22°C, natural light from the middle of October 2002 till March 2003.
0W GH LN	48	Flowering	135		
FT Field	57	Flowering	180	Days to flowering of plants grown in the field.	Growth in field or greenhouse (20°C, 16 hrs daylight), started in October.
FT Diameter Field	58	Flowering	180	Plant diameter at flowering (field).	
FT GH	59	Flowering	166	Days to flowering (greenhouse).	
FT Duration GH	60	Developmental	147	Flowering period duration.	Growth in greenhouse, 20°C, 16 hrs daylight.
LC Duration GH	61	Developmental	147	Life cycle period.	
LFS GH	62	Developmental	148	Last flower senescence.	
MT GH	63	Developmental	147	Maturation period.	
RP GH	64	Developmental	147	Reproduction period.	
At1	65		175		
At1 CFU2	66		175		
As	67		175		
As CFU2	68		175		
Bs	69		175		

Phenotype	ID	Type	Sample size	Description	Growth Conditions
Bs CFU2	70		175		
At2	71		175		
At2 CFU2	72		175		
As2	73		175		
As2 CFU2	74		175		
FW	75	Developmental	95	Fresh weight of plants.	Plants were grown for 7 weeks at 23°C.
DW	76	Developmental	95	Dry weight of plants.	
LN10	80	Flowering	177	Leaf number at flowering time (LN).	10°C, 16 hrs daylight.
LN16	81	Flowering	176		16°C, 16 hrs daylight.
LN22	82	Flowering	176		22°C, 16 hrs daylight.
Silique 16	158	Developmental	95	Silique length.	16°C, 16 hrs daylight.
Silique 22	159	Developmental	95		22°C, 16 hrs daylight.
Germ 22	163	Flowering	177	Days to germination.	Stratified for 3 days at 4°C in the dark, followed by growth at 22°C with 16 hrs daylight.
Width 10	164	Developmental	176	Plant diameter.	10°C, 16 hrs daylight.
Width 16	165	Developmental	175		16°C, 16 hrs daylight.
Width 22	166	Developmental	175		22°C, 16 hrs daylight.
Leaf serr 10	173	Developmental	174	Level of leaf serration.	10°C, 16 hrs daylight.
Leaf serr 16	174	Developmental	176		16°C, 16 hrs daylight.
Leaf serr 22	175	Developmental	176		22°C, 16 hrs daylight.
Hypocotyl length	182	Developmental	89	Hypocotyl length.	Seeds were vapor sterilized, plated in a random design on 1/2 MS agar plates (-sucrose), stratified for 4 days at 4°C in the dark, before transferring to photocycles (12 h 100 µ E/m^{2}s cool white light / 12 hours dark) and thermocycles (12 h 22°C / 12 h 12°C)
Trichome avg C	183	Defense-related	94	Trichome density.	20°C, 12 hrs daylight.
Trichome avg JA	184	Defense-related	94		
Aphid number	185	Defense-related	95	Aphid offspring.	20°C, 12 hrs daylight.

Phenotype	ID	Type	Sample size	Description	Growth Conditions
Bacterial titer	186	Flowering	95	Bacterial titers of *Pseudomonas syringae pv. tomato* DC3000.	20°C, 12 hrs daylight.
Seedling Growth	272	Developmental	101	Seedling growth rate.	Seeds were grown for one week in the greenhouse under long day (16 hours light).
Vern Growth	273	Developmental	111	Vegetative growth rate during vernalization.	Seeds were grown for one week in the greenhouse under long day (16 hours light), vernalized for 4 weeks (4°C, 16h light, 50% relative humidity).
After Vern Growth	274	Developmental	111	Vegetative growth rate after vernalization.	Seeds were grown for one week in the greenhouse under long day (16 hours light), vernalized for 4 weeks (4°C, 16 hrs light, 50% relative humidity)and then returned to greenhouse.
Secondary Dormancy	277	Developmental	94	Decrease in germination rate after prolonged exposure to cold temperature.	Fully after-ripened seeds were treated with a 1 and 6-week long exposure to 4°C.
Germ in dark	278	Developmental	94	Germination in the dark.	4°C, in the dark.
DSDS50	279	Developmental	110	Duration of seed dry storage required for 50% of the seeds to germinate.	Dry storage, followed by 25°C, 12 hrs day, 20°C, 12 hrs night for 1 week.
Seed bank 133-91	280	Developmental	111	Non-monotonous dynamic of dormancy release.	Between 91 and 133 days of dry storage.
Storage 7 days	281	Developmental	111	Primary dormancy.	7 days dry storage.
Storage 28 days	282	Developmental	111		28 days dry storage.
Storage 56 days	283	Developmental	111		56 days dry storage.

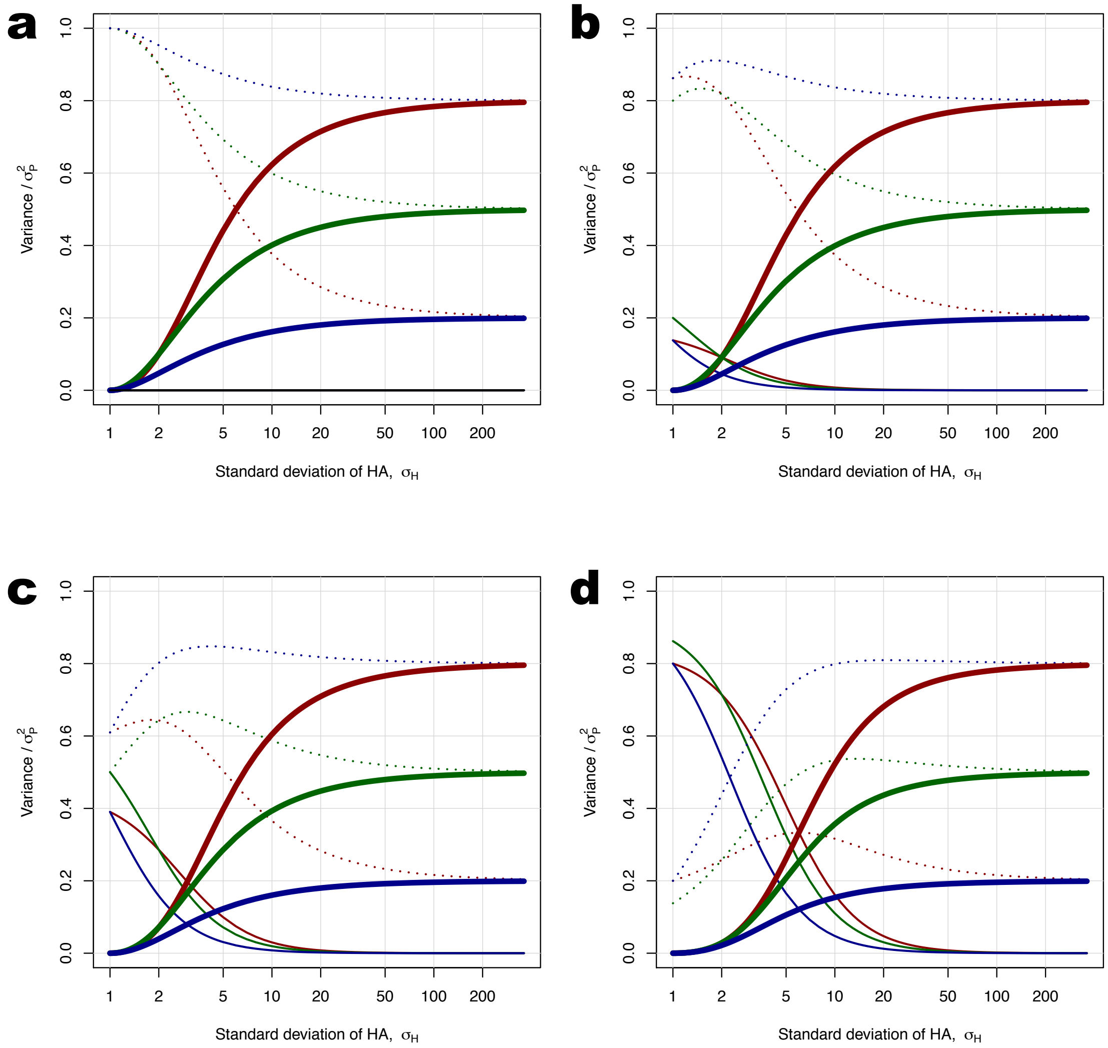

Supplementary Figure A1: Dissection of the phenotypic variance with respect to the level of variance heterogeneity. The standard deviation of the low-variance allele (LA) was set to 1. The additive effect was set to be 0 (**a**), 1 (**b**), 2 (**c**) and 5 (**d**). The red, green and blue colors correspond to low-variance allele frequencies 0.8, 0.5 and 0.2. The phenotypic variance V_P is dissected into variance due to mean shift, V_M (= V_A, thin solid lines), variance due to variance heterogeneity between genotypes, V_V (bold solid lines) and residual variance V_R (dotted lines).

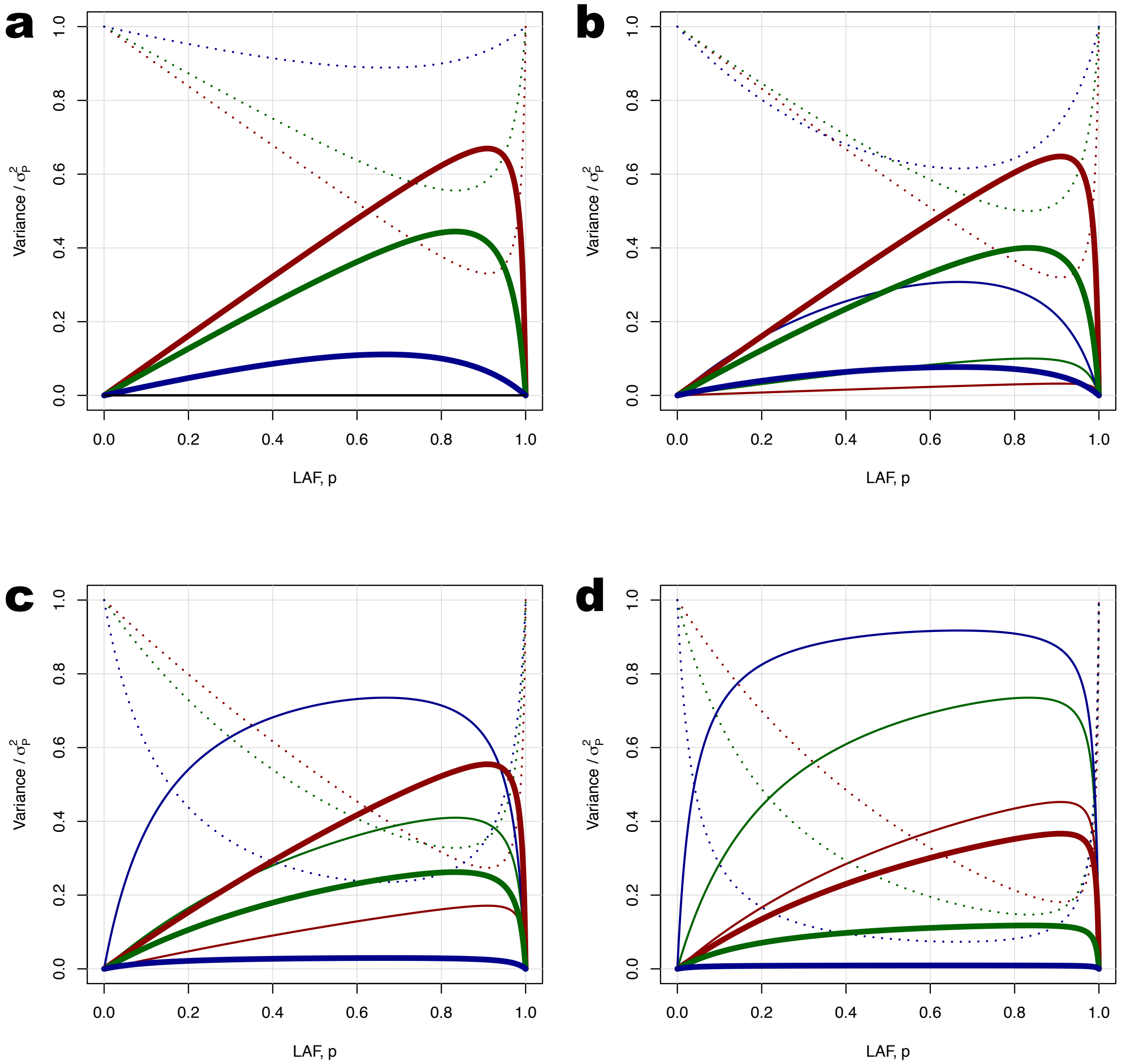

Supplementary Figure A2: Dissection of the phenotypic variance with respect to the low-variance allele frequency (LAF). The standard deviation of the low-variance allele (LA) was set to 1. The additive effect was set to be 0 (**a**), 2 (**b**), 5 (**c**) and 10 (**d**). The red, green and blue colors correspond to standard deviations of the high-variance allele 10, 5 and 2. The phenotypic variance V_P is dissected into variance due to mean shift, V_M (= V_A, thin solid lines), variance due to variance heterogeneity between genotypes, V_V (bold solid lines) and residual variance V_R (dotted lines).

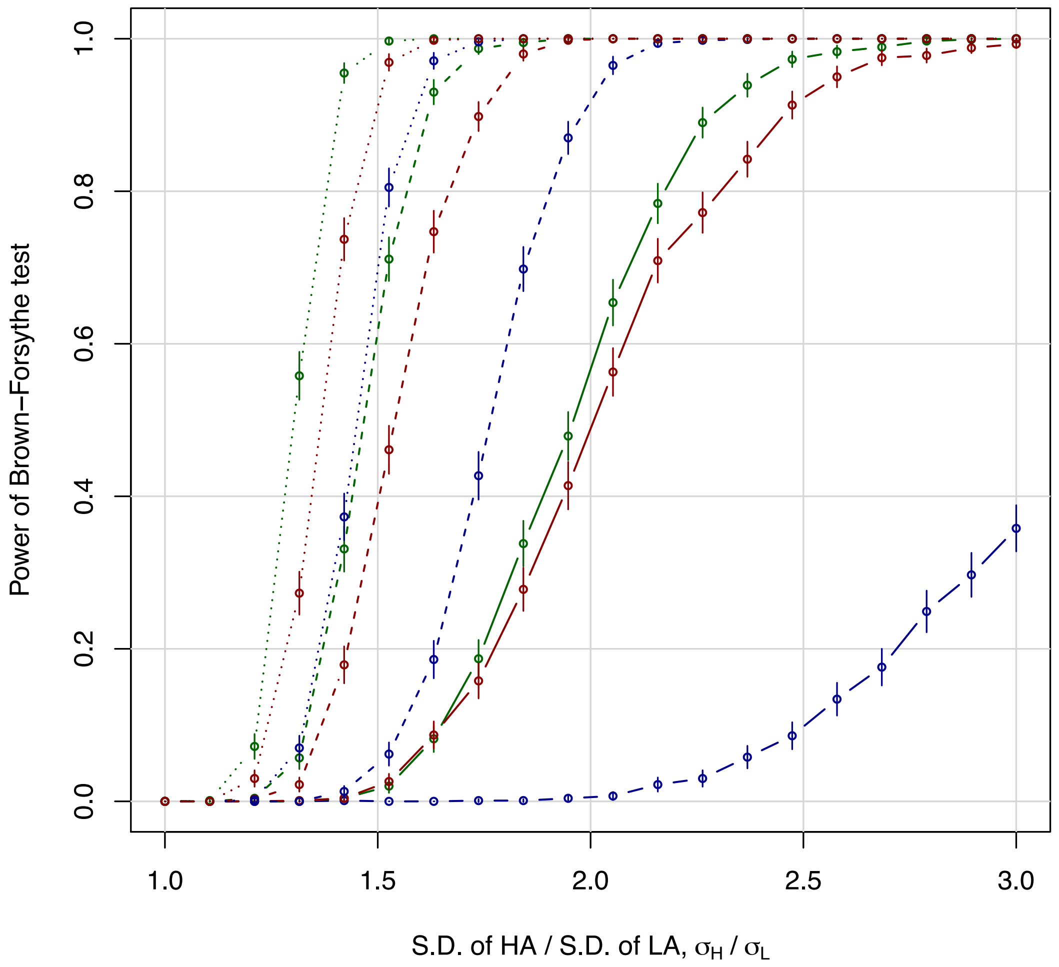

Supplementary Figure A3: Power of the Brown-Forsythe test with respect to the level of variance heterogeneity}. The significant threshold was set to be 5×10^{-8}. The level of variance heterogeneity is represented by the ratio of the standard deviation of the high-variance allele (HA) to that of the low-variance allele (LA). The red, green and blue colors correspond to low-variance allele frequencies (LAFs) 0.8, 0.5 and 0.2. The sample size of 200 (solid lines), 500 (dashed lines) and 1000 (dotted lines) were evaluated. 1000 simulations were performed for each point, and the vertical bars indicate ± twice standard errors.

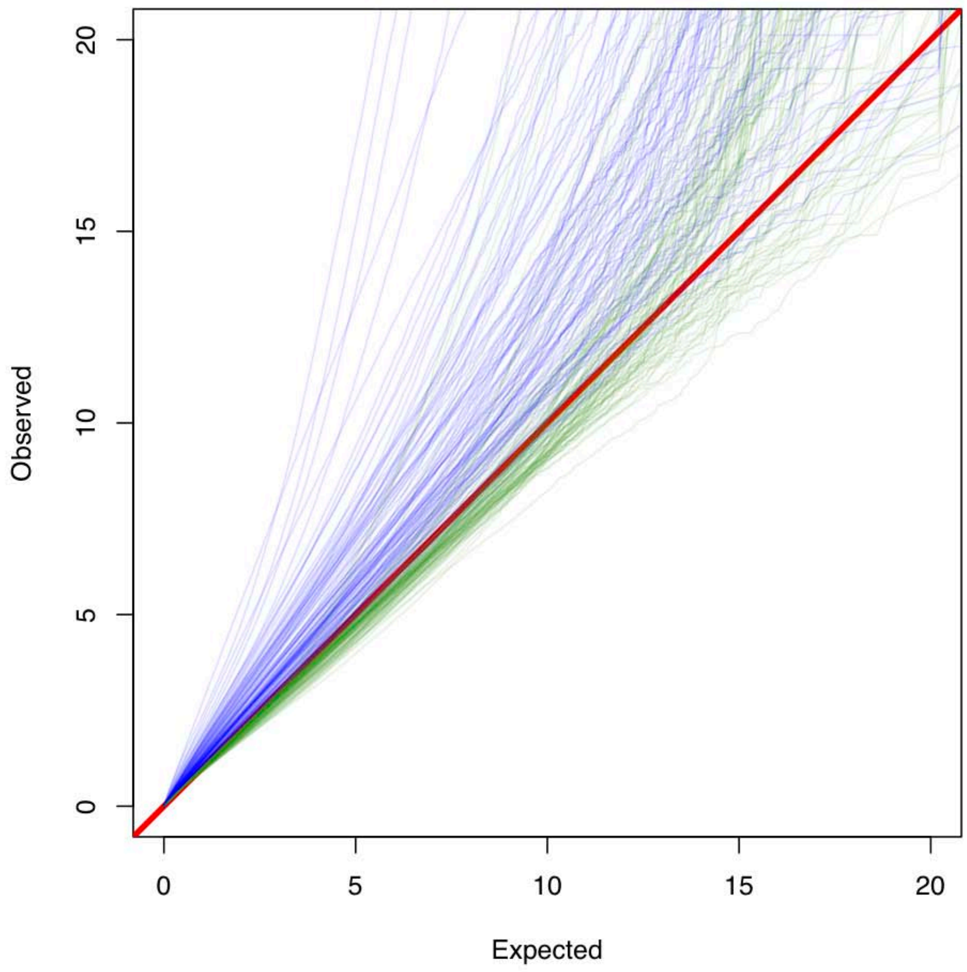

Supplementary Figure A4: A simulation study comparing the genome-wide p-values of vGWAS before (blue) and after (green) genomic control (GC). In each simulation/QQ-line, a SNP was randomly selected from the *Arabidopsis* genome, where a big variance-controlling effect was simulated with the standard deviation of the high-variance allele (HA) ~7.4 times of that of the low-variance allele (LA). 100 simulations were carried out. Genome-widely, the mean false positive rate was $3.9(\pm 1.4)\times 10^{-4}$ before GC and $4.7\ (\pm 1.2)\ \times 10^{-5}$ after GC, where shown in the parentheses are the standard errors. The genome-wide significant threshold was determined by Bonferroni correction.

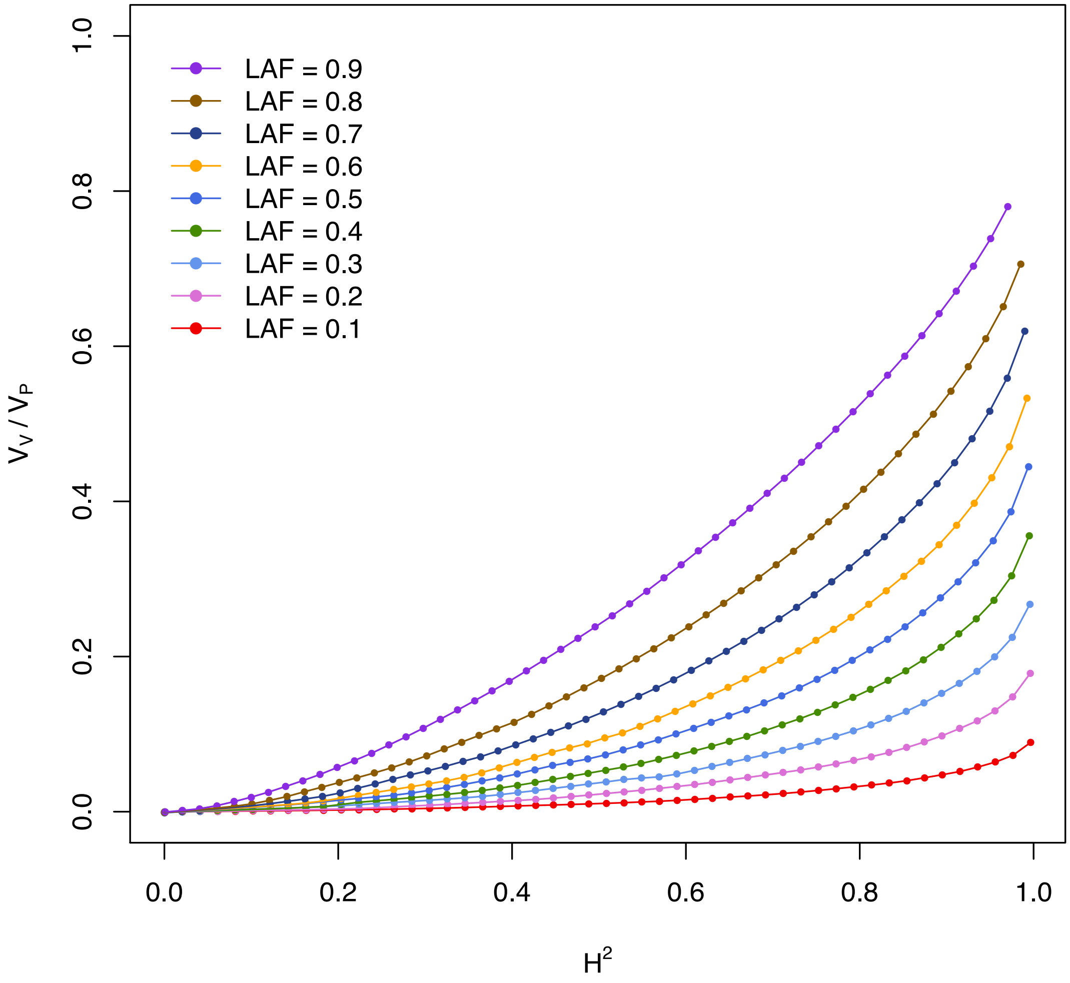

Supplementary Figure A5: The relationship between the variance portion due to variance heterogeneity and the broad sense heritability assuming a 2-way interaction model. The 2-way interaction model includes the main effect of the testing locus, the main effect of the other interacting factor (set to zero), and their interaction effect. The broad sense heritability is estimated as the coefficient of determination of the full interaction model. LAF = low-variance allele frequency.

Histogram of the phenotype & QQ plots of the Brown-Forsythe *p*-values (blue: nominal *p*-values, green: genomic controlled)

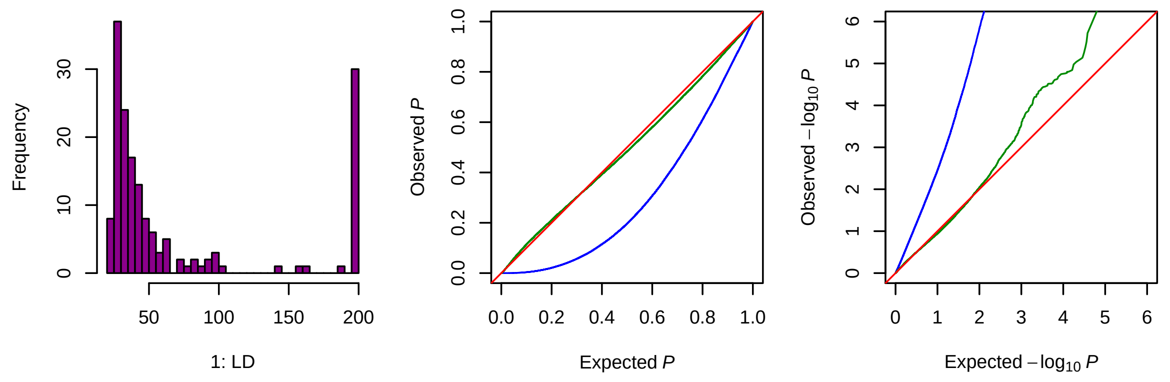

Brown-Forsythe (colored, behind) & Wilcoxon (yellow, in front) results, genomic controlled

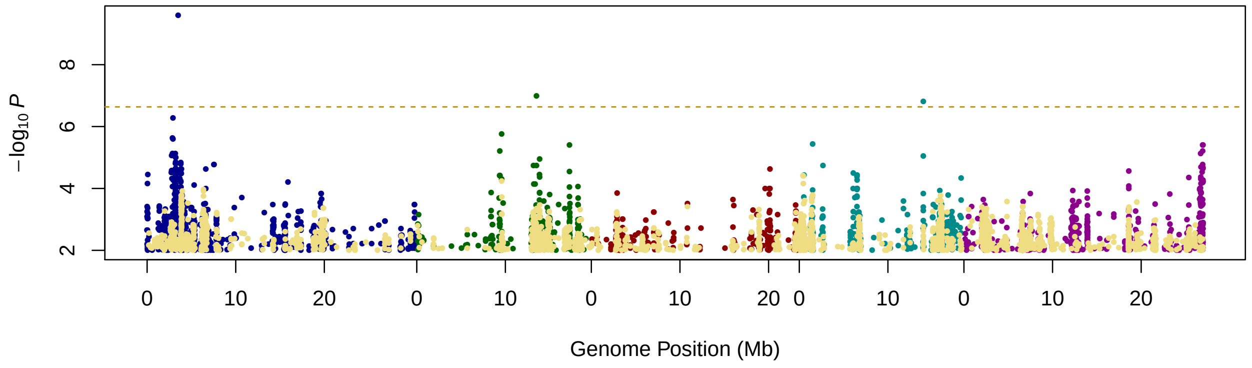

Comparison of Brown-Forsythe & Wilcoxon *p*-values (MAF: minor allele frequency)

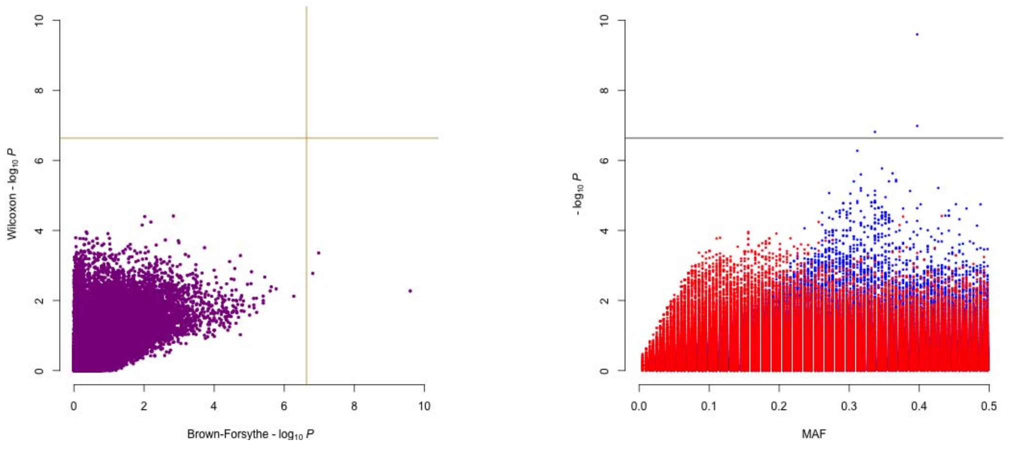

Supplementary Figure B1 - Summary of vGWA results for 1: LD

Histogram of the phenotype & QQ plots of the Brown-Forsythe *p*-values (blue: nominal *p*-values, green: genomic controlled)

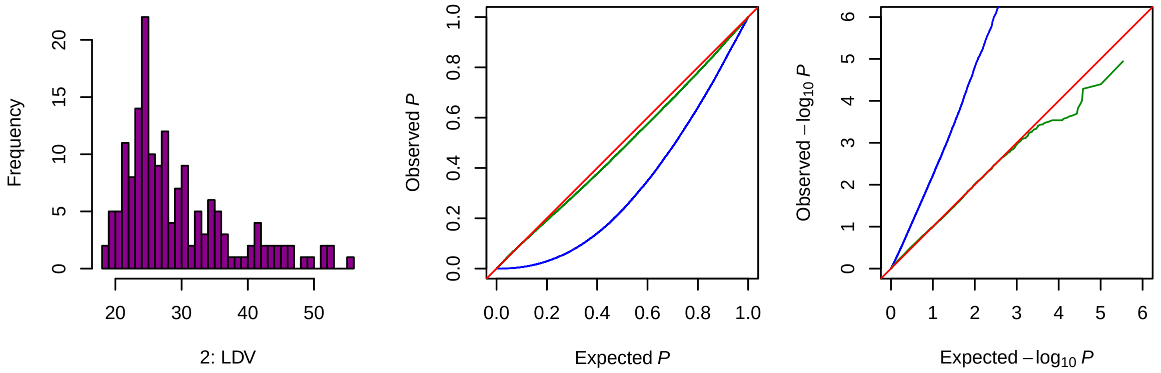

Brown-Forsythe (colored, behind) & Wilcoxon (yellow, in front) results, genomic controlled

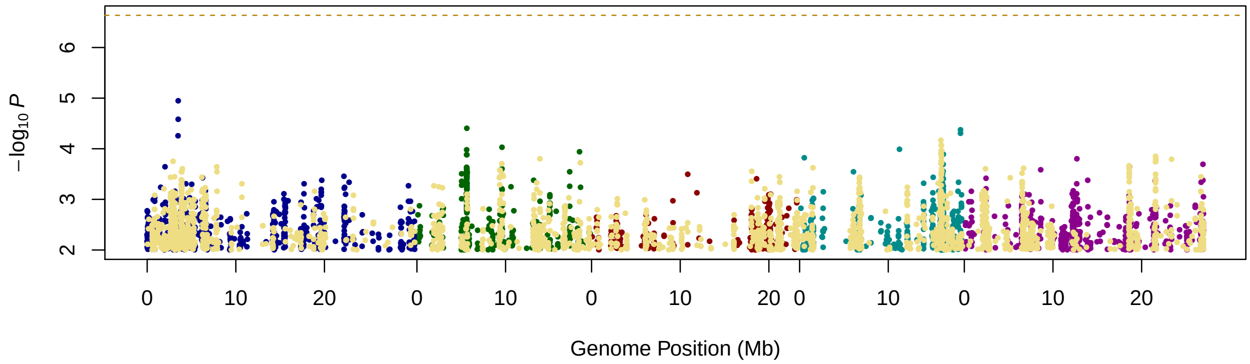

Comparison of Brown-Forsythe & Wilcoxon *p*-values (MAF: minor allele frequency)

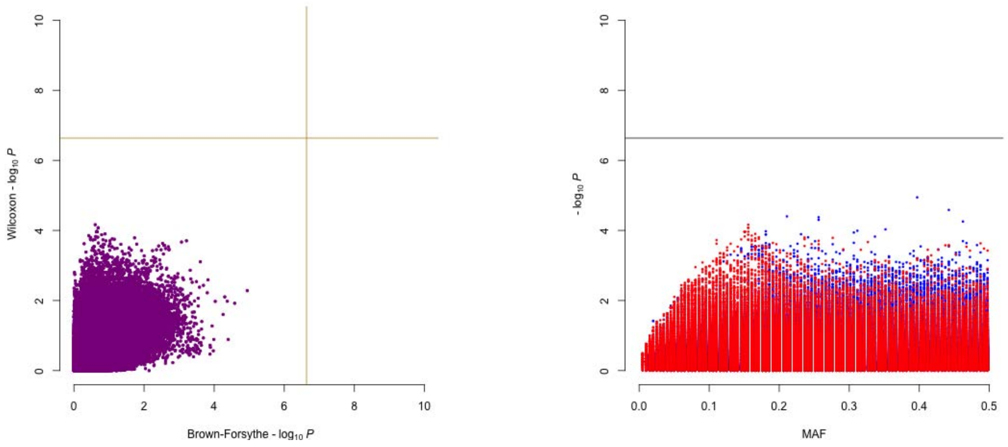

Supplementary Figure B2 - Summary of vGWA results for 2: LDV

Histogram of the phenotype & QQ plots of the Brown-Forsythe *p*-values (blue: nominal *p*-values, green: genomic controlled)

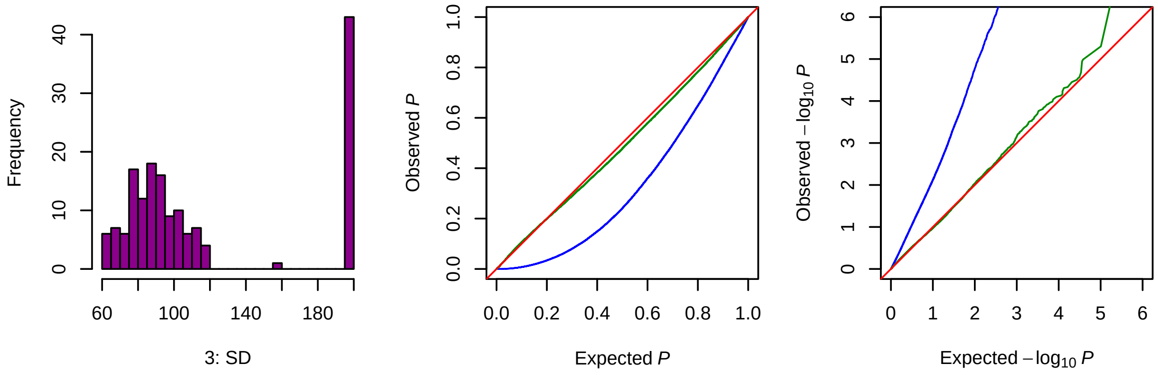

Brown-Forsythe (colored, behind) & Wilcoxon (yellow, in front) results, genomic controlled

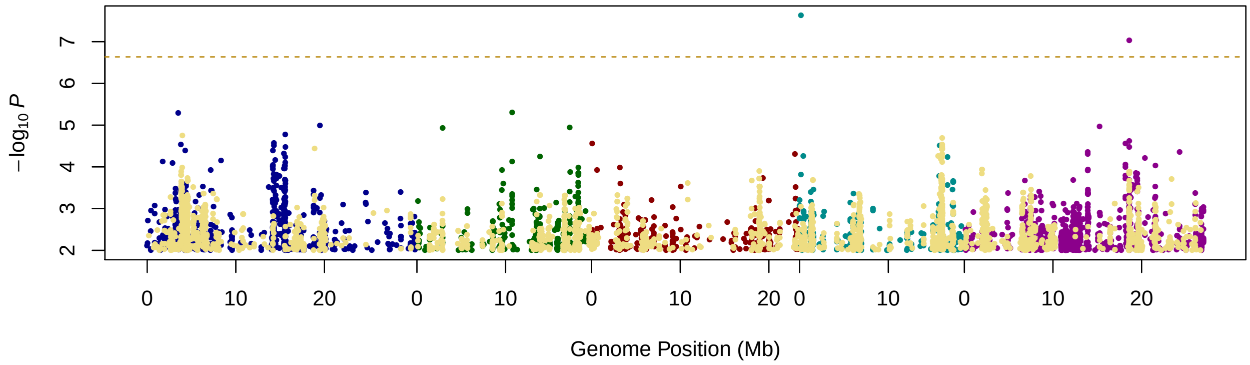

Comparison of Brown-Forsythe & Wilcoxon *p*-values (MAF: minor allele frequency)

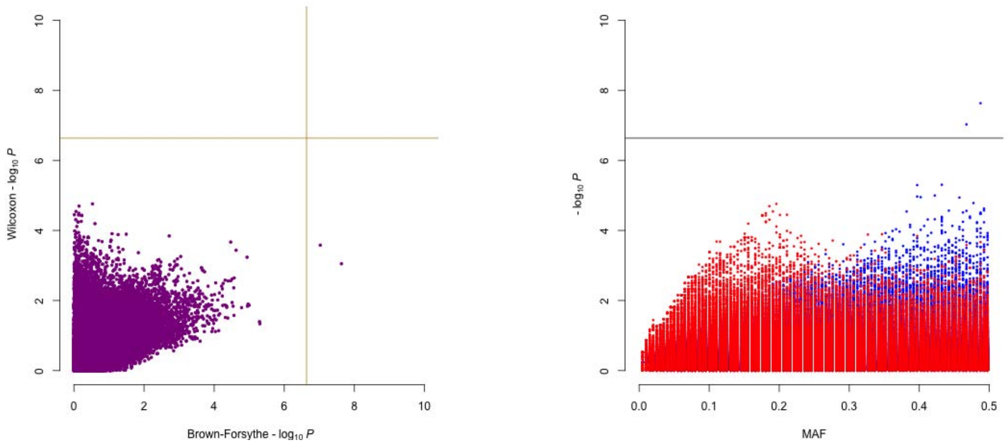

Supplementary Figure B3 - Summary of vGWA results for 3: SD

Histogram of the phenotype & QQ plots of the Brown-Forsythe *p*-values (blue: nominal *p*-values, green: genomic controlled)

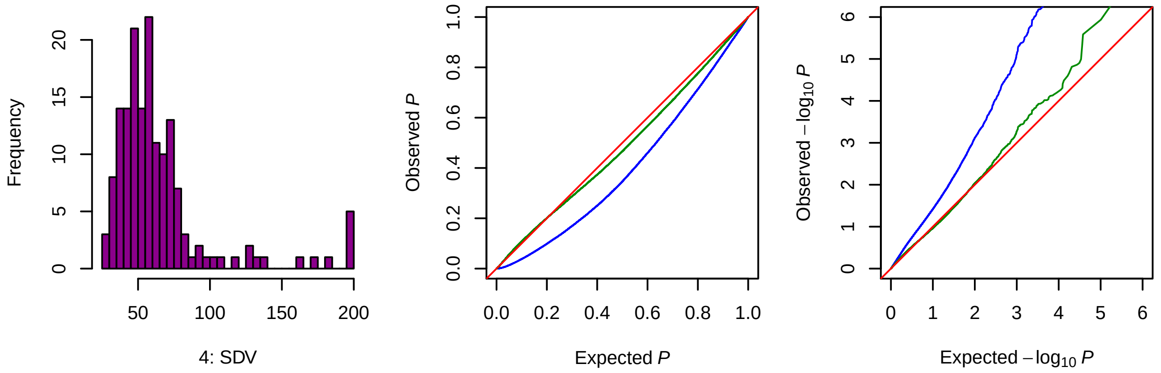

Brown-Forsythe (colored, behind) & Wilcoxon (yellow, in front) results, genomic controlled

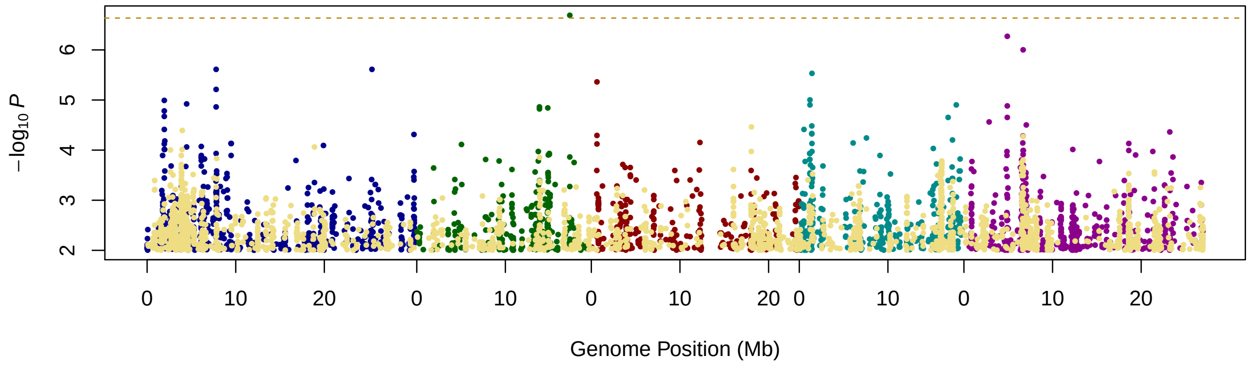

Comparison of Brown-Forsythe & Wilcoxon *p*-values (MAF: minor allele frequency)

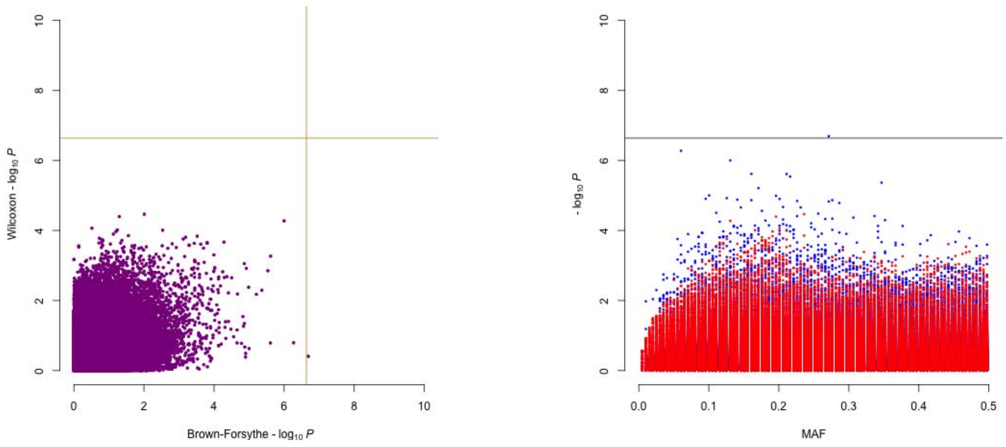

Supplementary Figure B4 - Summary of vGWA results for 4: SDV

Histogram of the phenotype & QQ plots of the Brown-Forsythe *p*-values (blue: nominal *p*-values, green: genomic controlled)

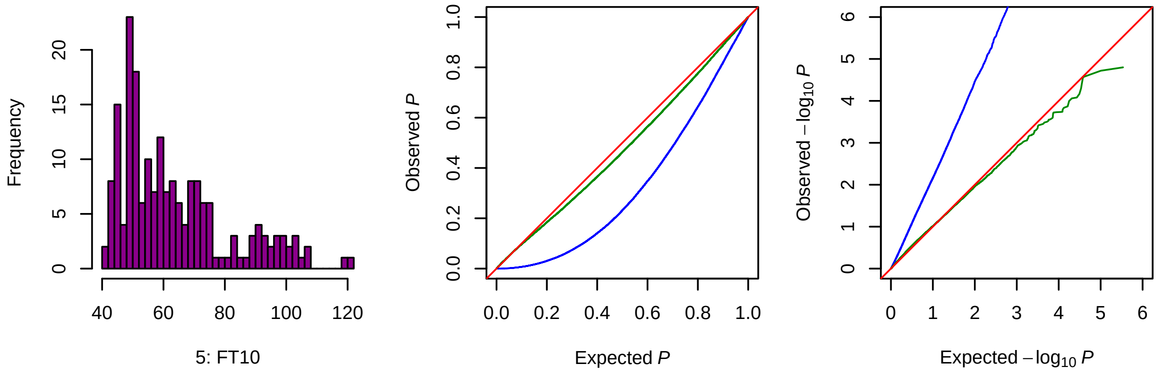

Brown-Forsythe (colored, behind) & Wilcoxon (yellow, in front) results, genomic controlled

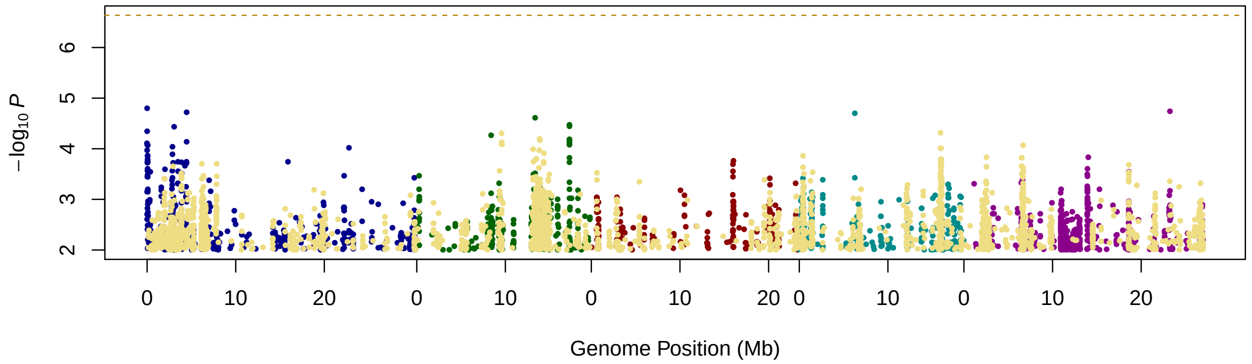

Comparison of Brown-Forsythe & Wilcoxon *p*-values (MAF: minor allele frequency)

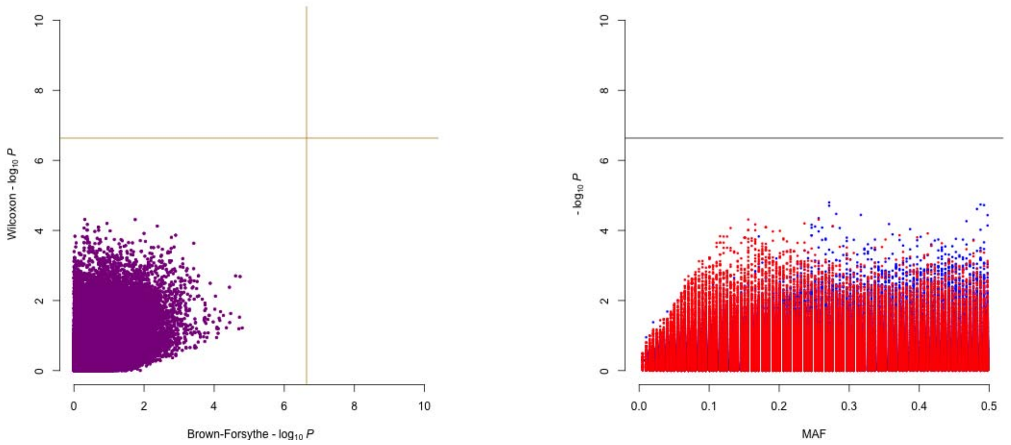

Supplementary Figure B5 - Summary of vGWA results for 5: FT10

Histogram of the phenotype & QQ plots of the Brown-Forsythe *p*-values (blue: nominal *p*-values, green: genomic controlled)

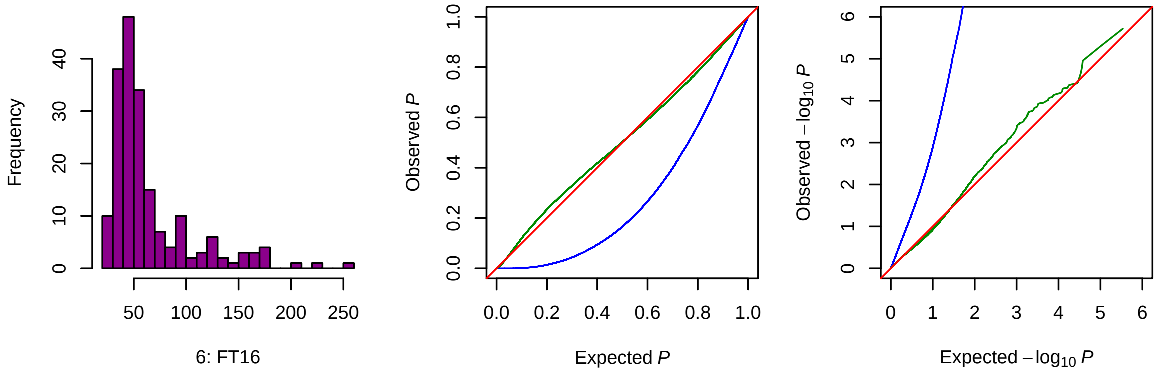

Brown-Forsythe (colored, behind) & Wilcoxon (yellow, in front) results, genomic controlled

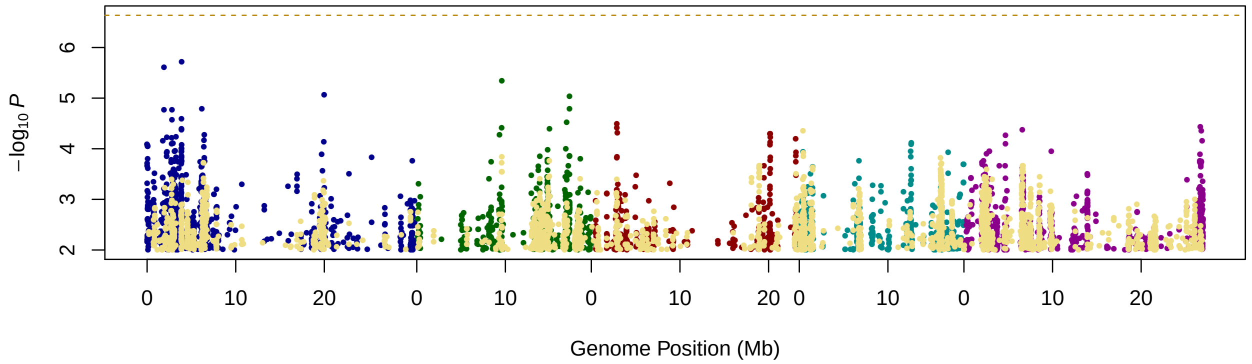

Comparison of Brown-Forsythe & Wilcoxon *p*-values (MAF: minor allele frequency)

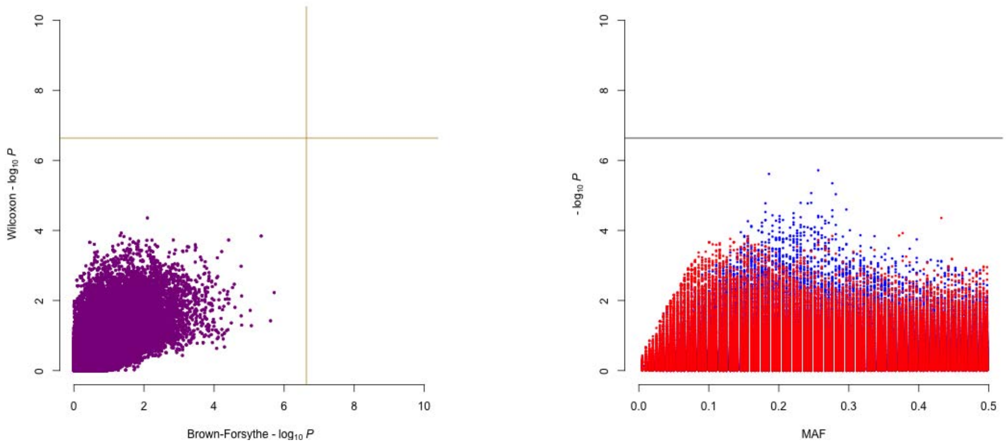

Supplementary Figure B6 - Summary of vGWA results for 6: FT16

Histogram of the phenotype & QQ plots of the Brown-Forsythe *p*-values (blue: nominal *p*-values, green: genomic controlled)

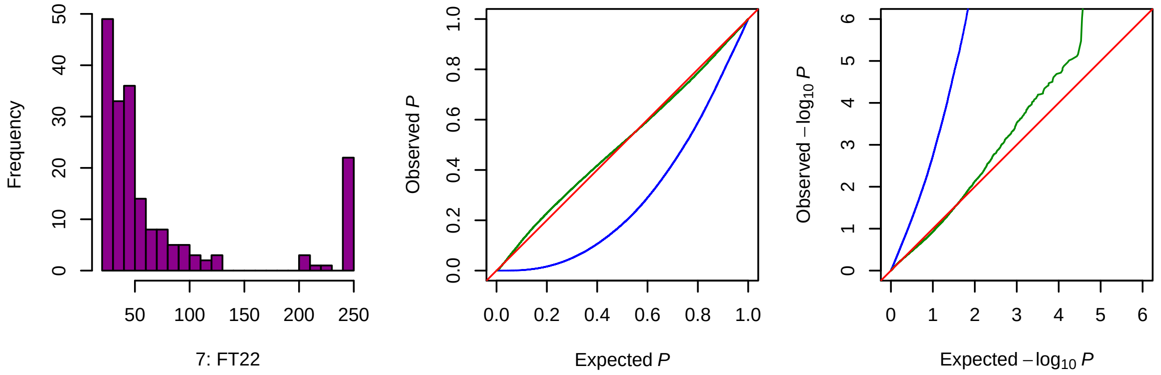

Brown-Forsythe (colored, behind) & Wilcoxon (yellow, in front) results, genomic controlled

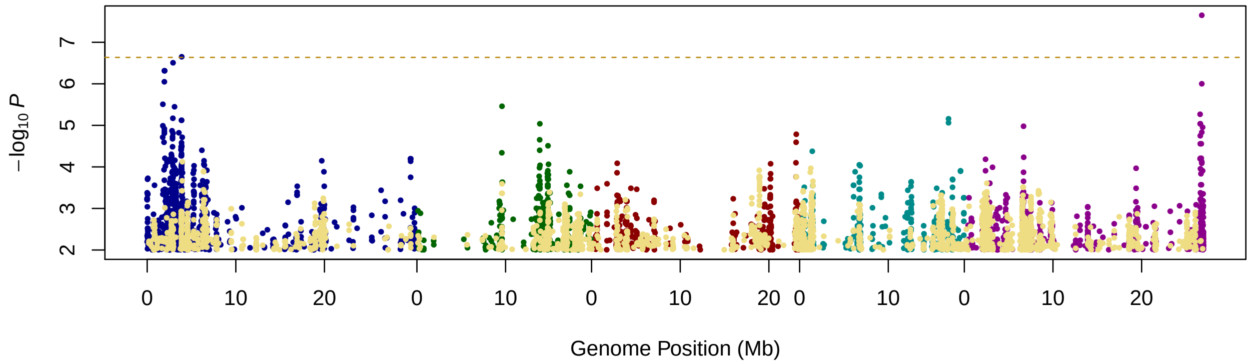

Comparison of Brown-Forsythe & Wilcoxon *p*-values (MAF: minor allele frequency)

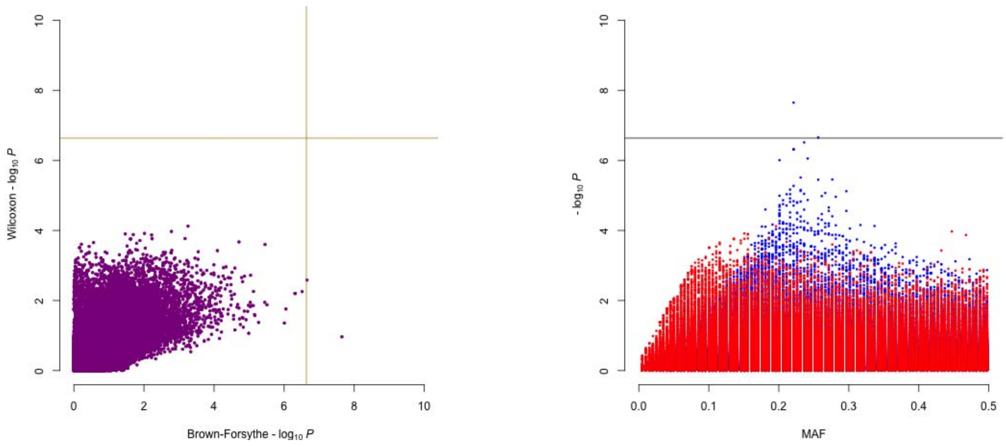

Supplementary Figure B7 - Summary of vGWA results for 7: FT22

Histogram of the phenotype & QQ plots of the Brown-Forsythe *p*-values (blue: nominal *p*-values, green: genomic controlled)

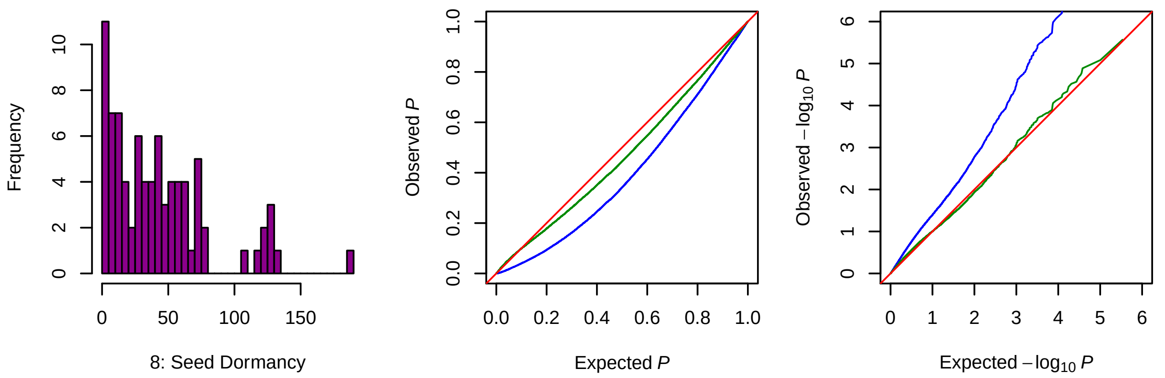

Brown-Forsythe (colored, behind) & Wilcoxon (yellow, in front) results, genomic controlled

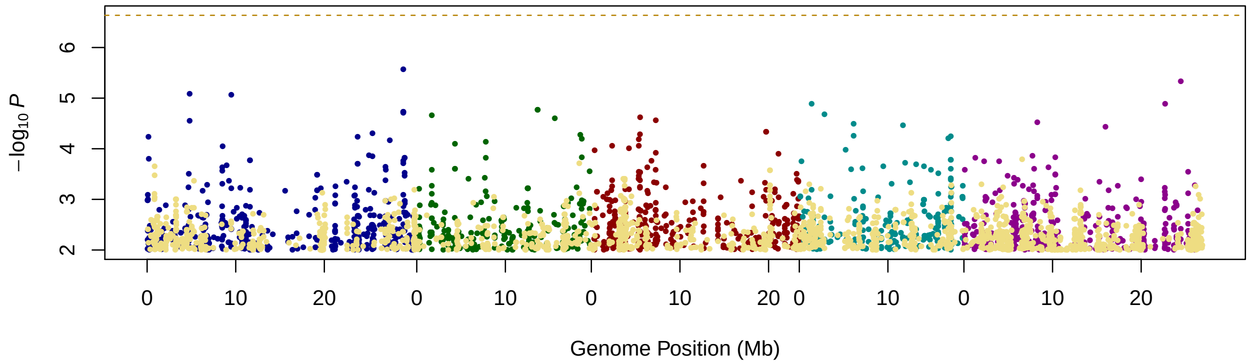

Comparison of Brown-Forsythe & Wilcoxon *p*-values (MAF: minor allele frequency)

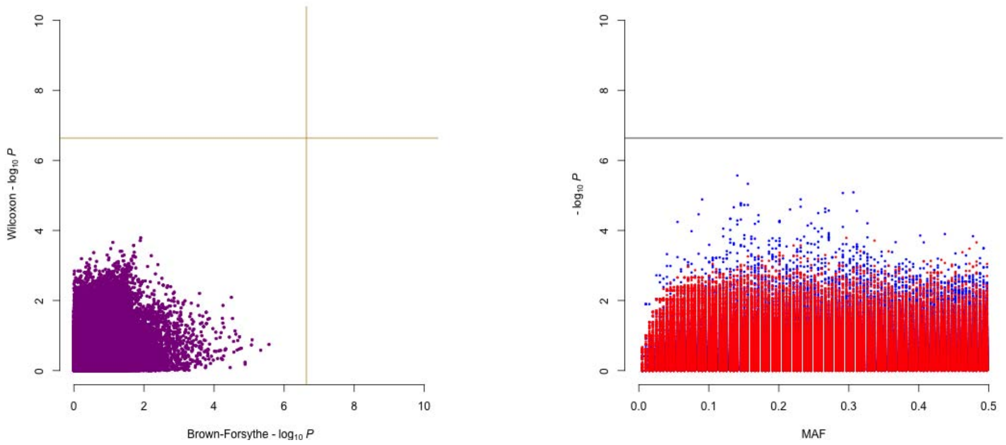

Supplementary Figure B8 - Summary of vGWA results for 8: Seed Dormancy

Histogram of the phenotype & QQ plots of the Brown-Forsythe *p*-values (blue: nominal *p*-values, green: genomic controlled)

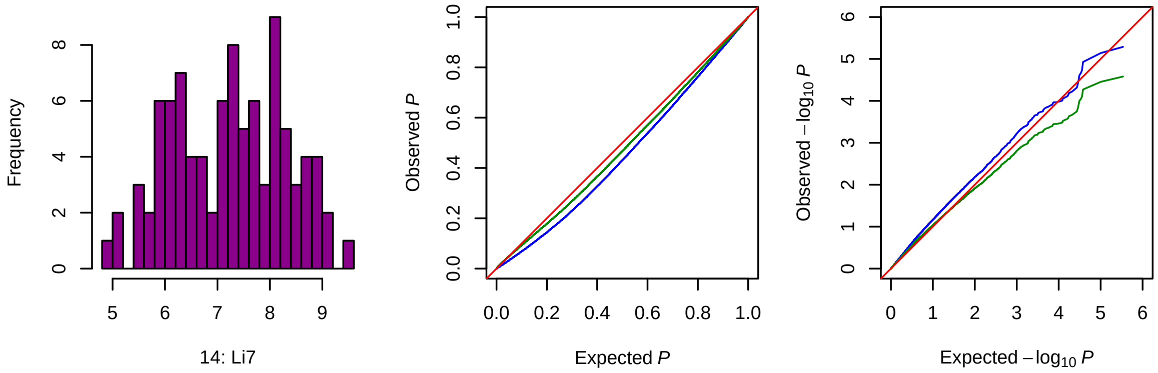

Brown-Forsythe (colored, behind) & Wilcoxon (yellow, in front) results, genomic controlled

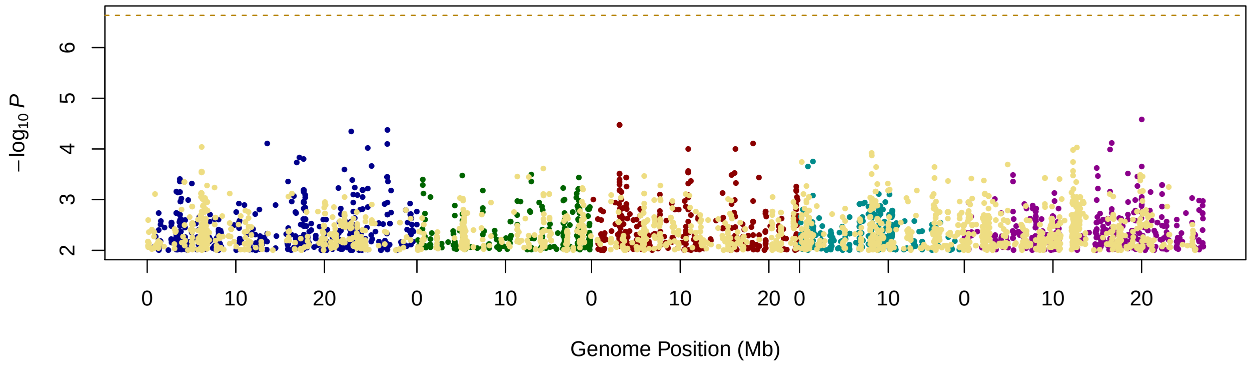

Comparison of Brown-Forsythe & Wilcoxon *p*-values (MAF: minor allele frequency)

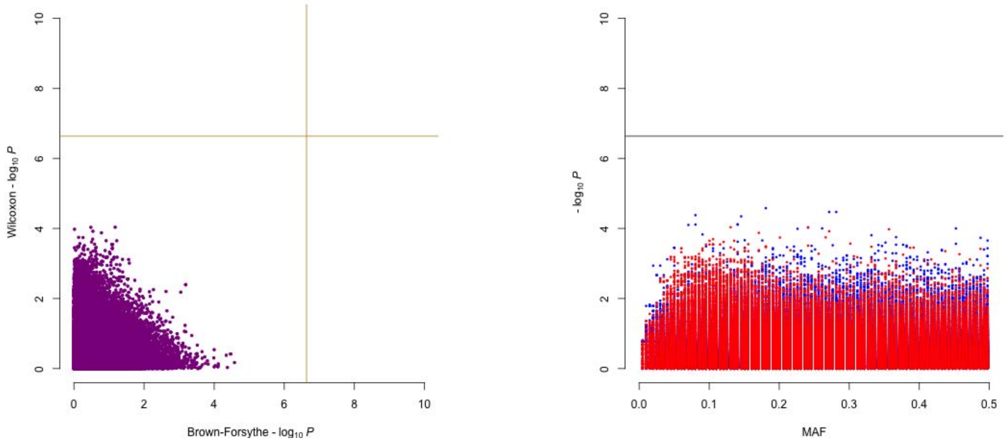

Supplementary Figure B9 - Summary of vGWA results for 14: Li7

Histogram of the phenotype & QQ plots of the Brown-Forsythe *p*-values (blue: nominal *p*-values, green: genomic controlled)

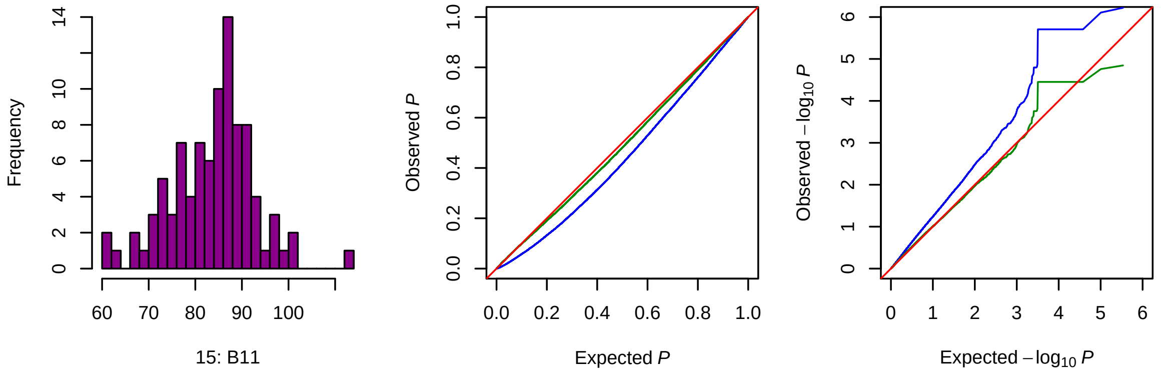

Brown-Forsythe (colored, behind) & Wilcoxon (yellow, in front) results, genomic controlled

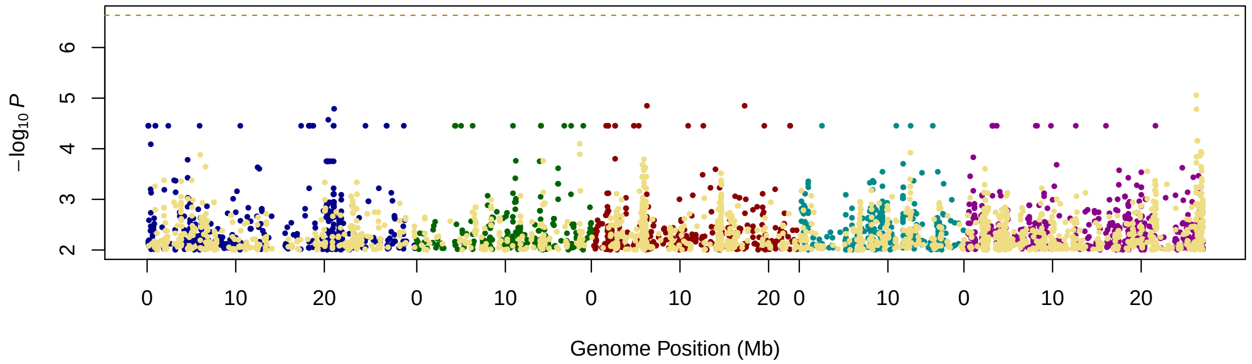

Comparison of Brown-Forsythe & Wilcoxon *p*-values (MAF: minor allele frequency)

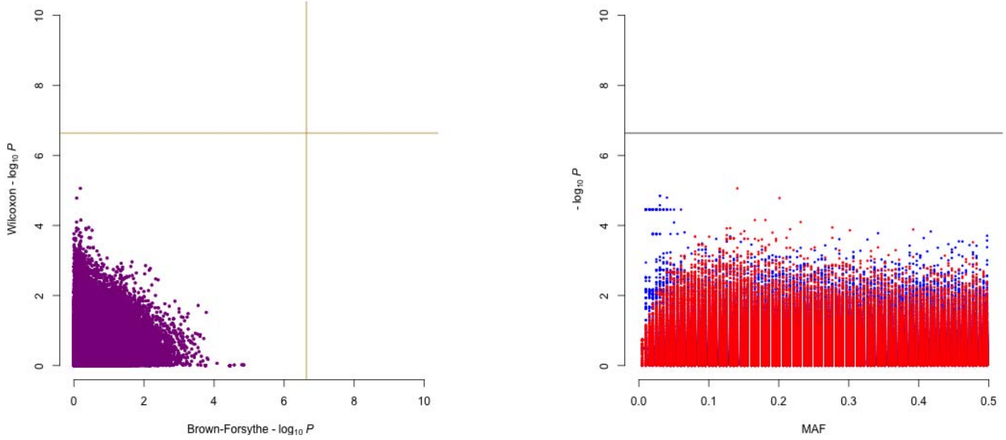

Supplementary Figure B10 - Summary of vGWA results for 15: B11

Histogram of the phenotype & QQ plots of the Brown-Forsythe *p*-values (blue: nominal *p*-values, green: genomic controlled)

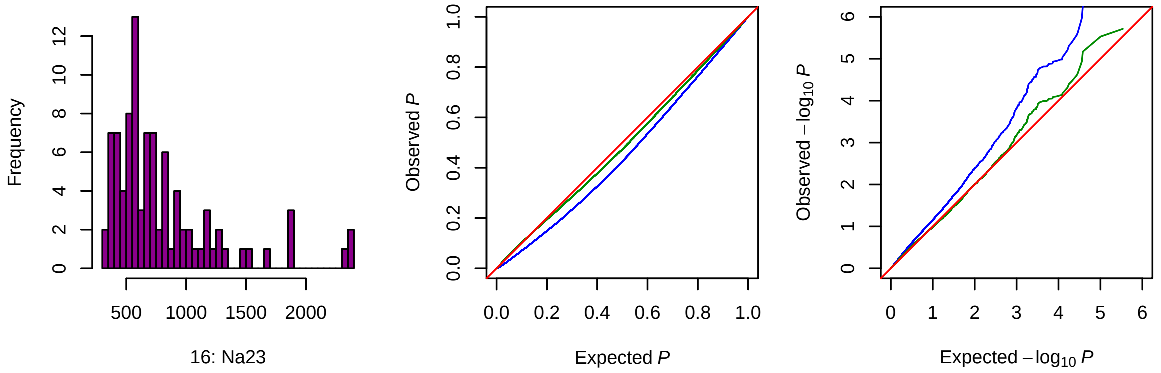

Brown-Forsythe (colored, behind) & Wilcoxon (yellow, in front) results, genomic controlled

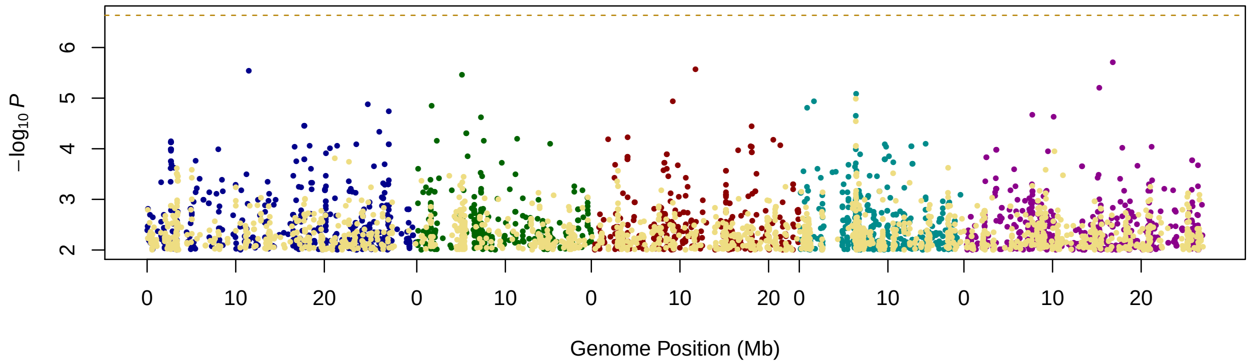

Comparison of Brown-Forsythe & Wilcoxon *p*-values (MAF: minor allele frequency)

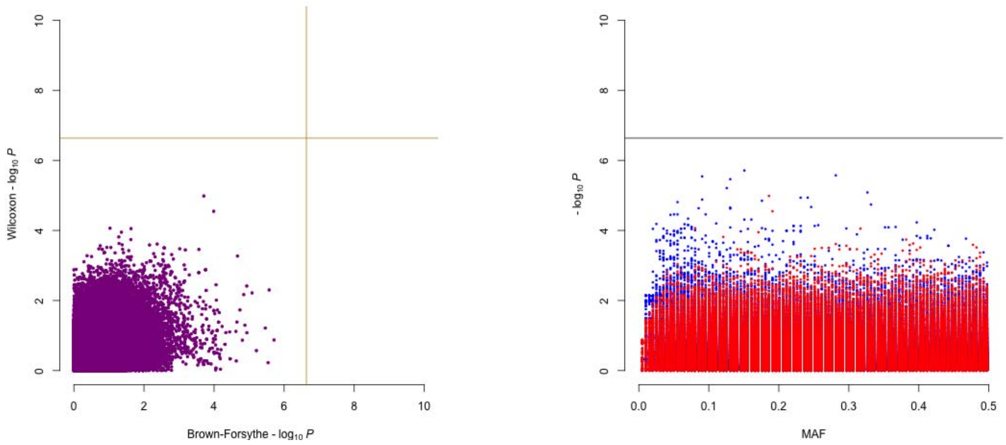

Supplementary Figure B11 - Summary of vGWA results for 16: Na23

Histogram of the phenotype & QQ plots of the Brown-Forsythe *p*-values (blue: nominal *p*-values, green: genomic controlled)

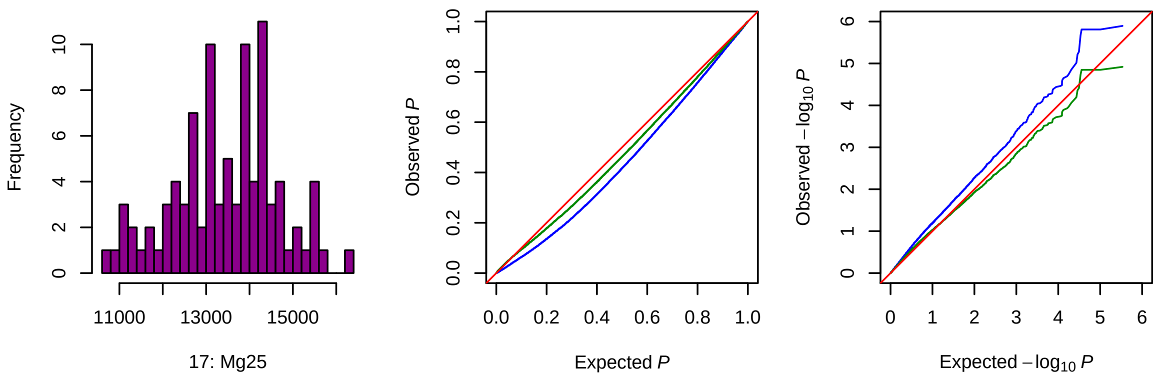

Brown-Forsythe (colored, behind) & Wilcoxon (yellow, in front) results, genomic controlled

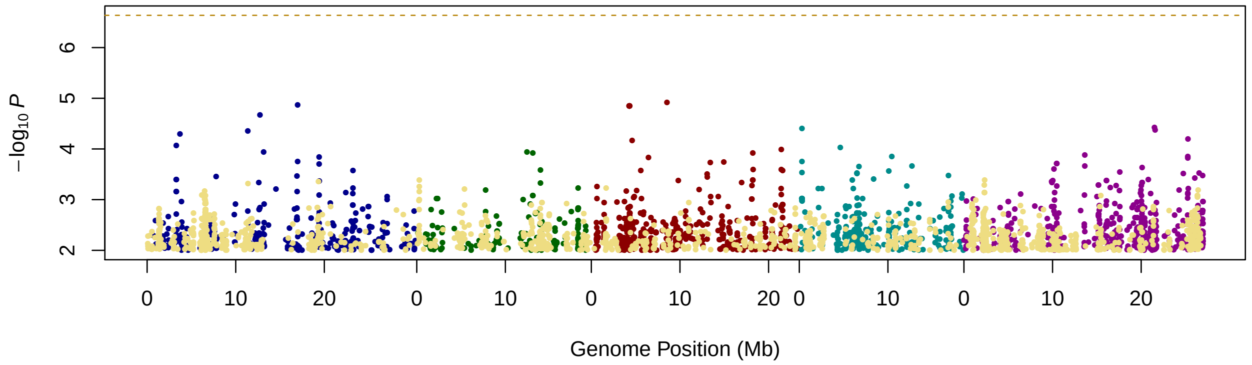

Comparison of Brown-Forsythe & Wilcoxon *p*-values (MAF: minor allele frequency)

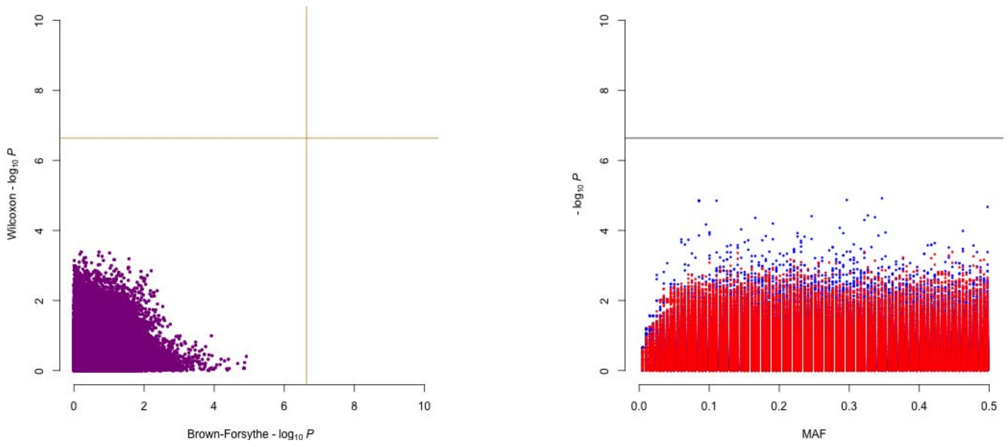

Supplementary Figure B12 - Summary of vGWA results for 17: Mg25

Histogram of the phenotype & QQ plots of the Brown-Forsythe *p*-values (blue: nominal *p*-values, green: genomic controlled)

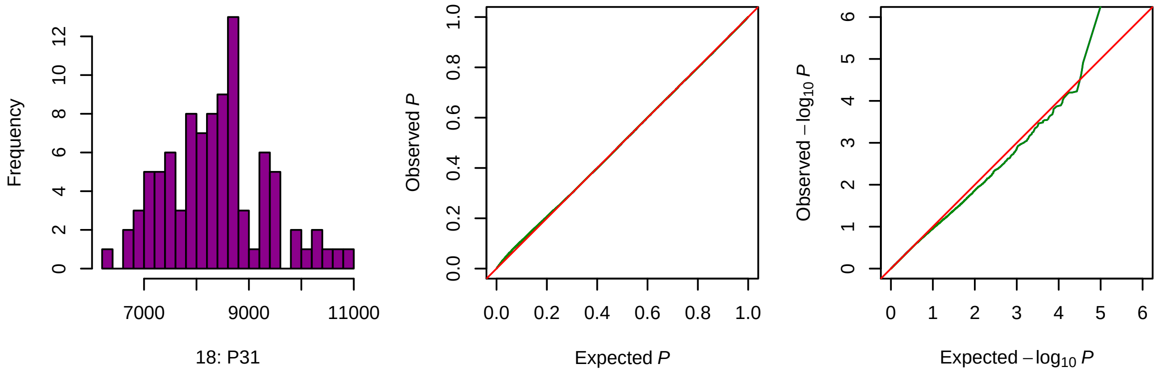

Brown-Forsythe (colored, behind) & Wilcoxon (yellow, in front) results, genomic controlled

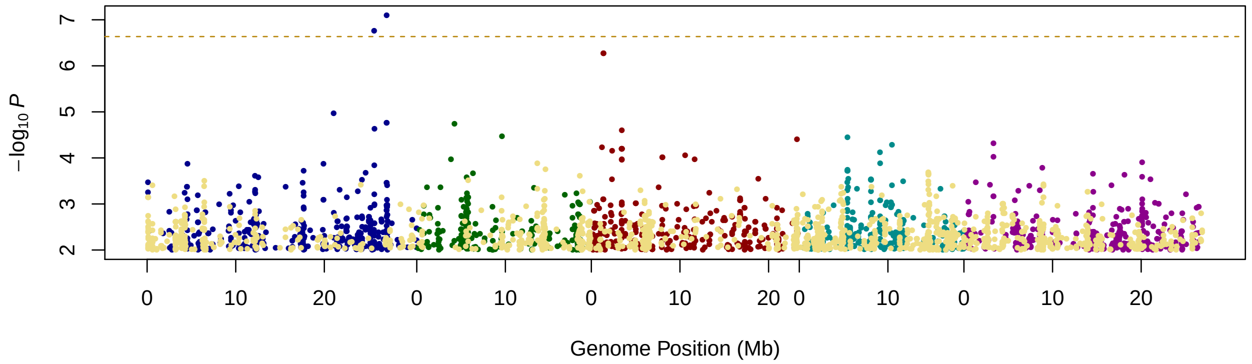

Comparison of Brown-Forsythe & Wilcoxon *p*-values (MAF: minor allele frequency)

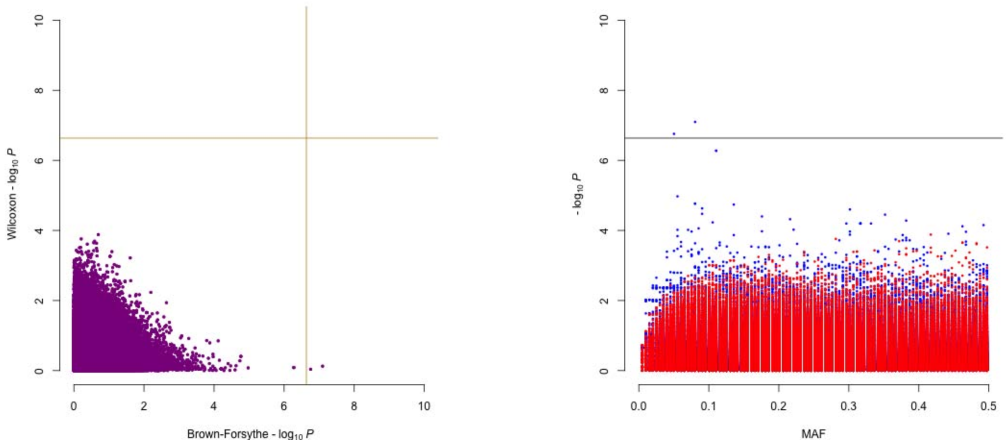

Supplementary Figure B13 - Summary of vGWA results for 18: P31

Histogram of the phenotype & QQ plots of the Brown-Forsythe *p*-values (blue: nominal *p*-values, green: genomic controlled)

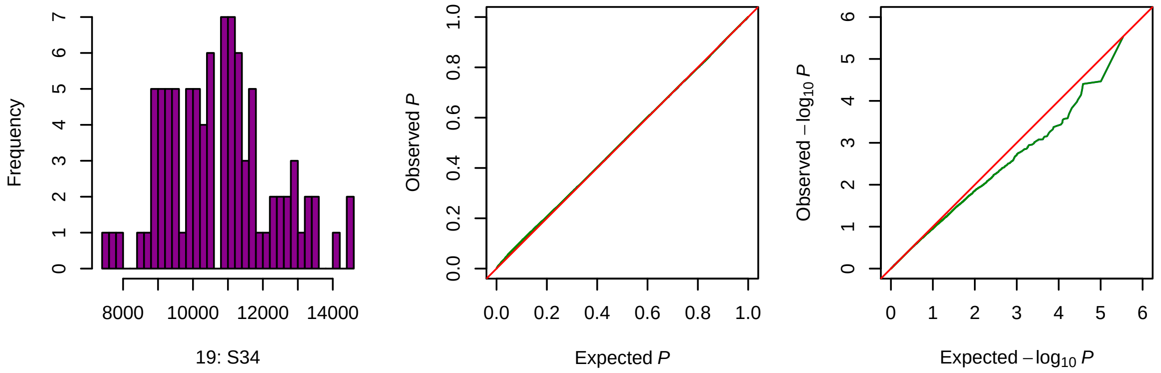

Brown-Forsythe (colored, behind) & Wilcoxon (yellow, in front) results, genomic controlled

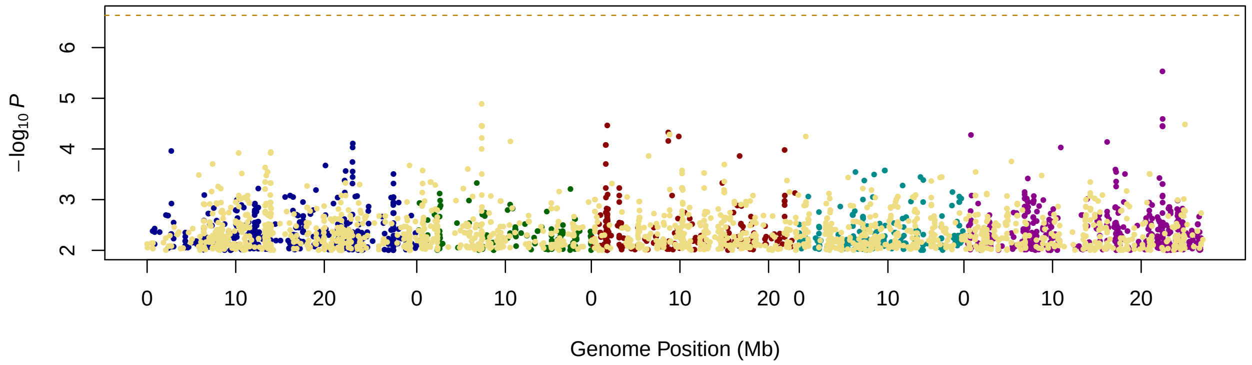

Comparison of Brown-Forsythe & Wilcoxon *p*-values (MAF: minor allele frequency)

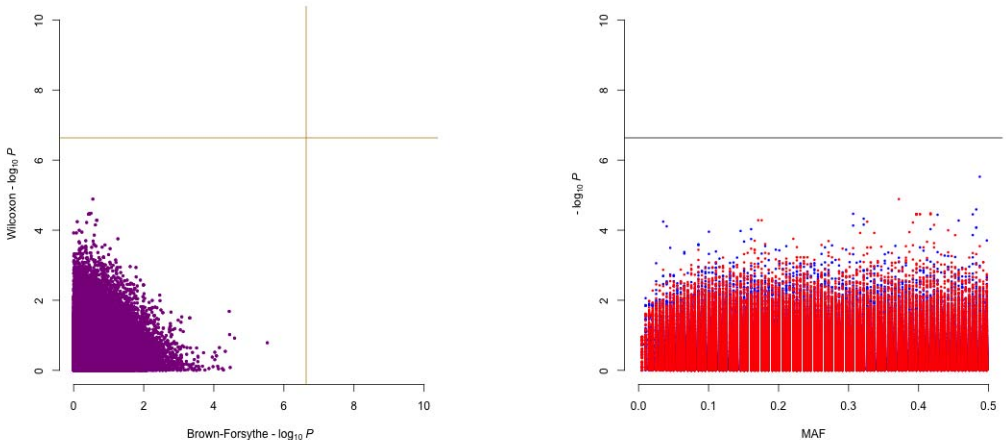

Supplementary Figure B14 - Summary of vGWA results for 19: S34

Histogram of the phenotype & QQ plots of the Brown-Forsythe *p*-values (blue: nominal *p*-values, green: genomic controlled)

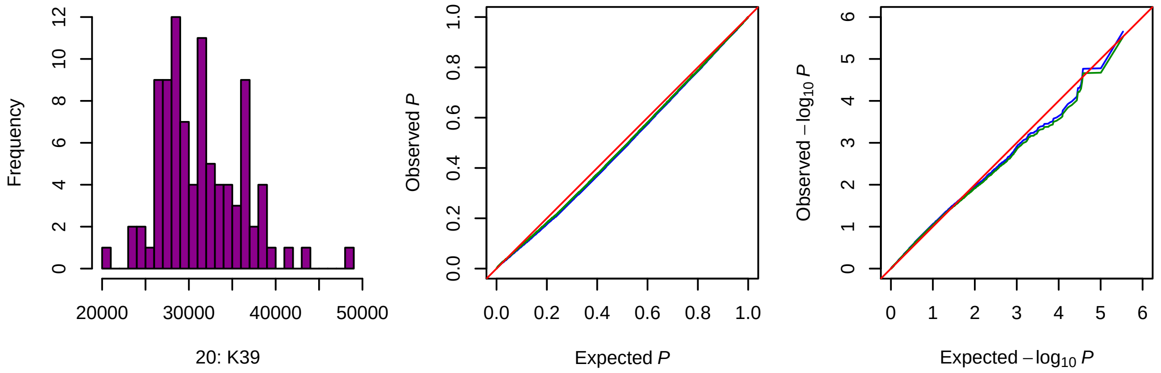

Brown-Forsythe (colored, behind) & Wilcoxon (yellow, in front) results, genomic controlled

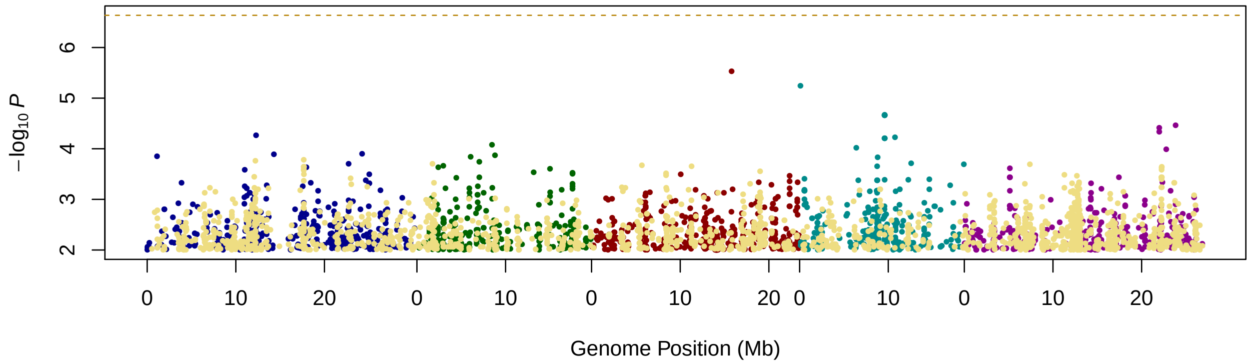

Comparison of Brown-Forsythe & Wilcoxon *p*-values (MAF: minor allele frequency)

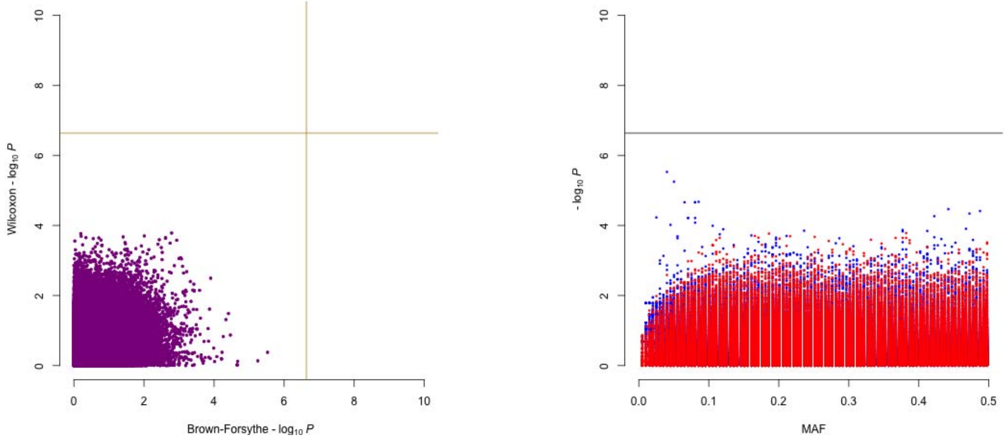

Supplementary Figure B15 - Summary of vGWA results for 20: K39

Histogram of the phenotype & QQ plots of the Brown-Forsythe *p*-values (blue: nominal *p*-values, green: genomic controlled)

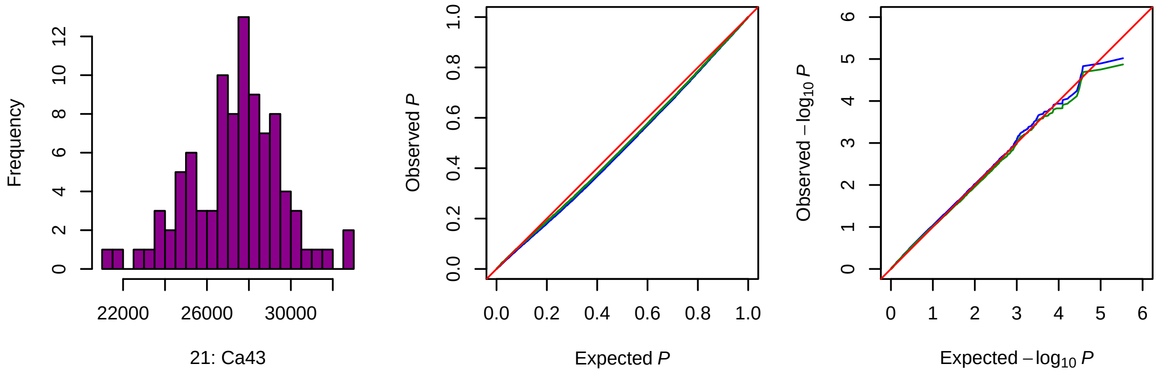

Brown-Forsythe (colored, behind) & Wilcoxon (yellow, in front) results, genomic controlled

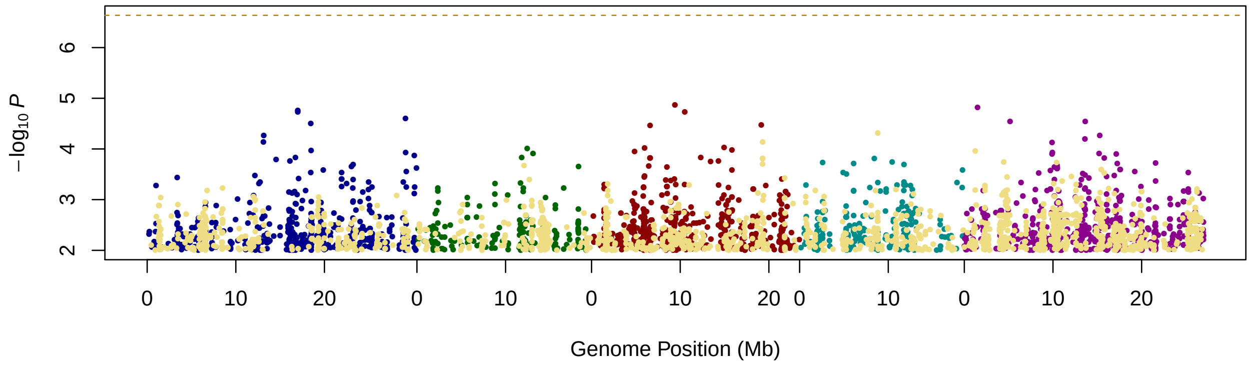

Comparison of Brown-Forsythe & Wilcoxon *p*-values (MAF: minor allele frequency)

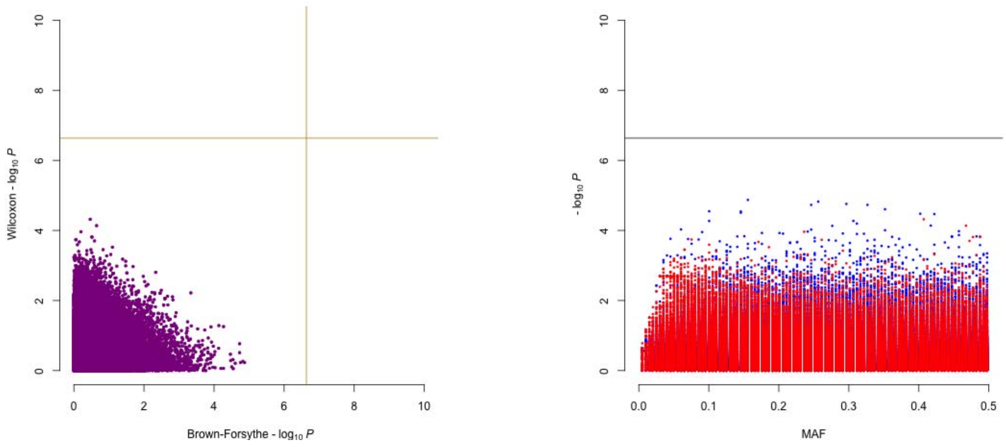

Supplementary Figure B16 - Summary of vGWA results for 21: Ca43

Histogram of the phenotype & QQ plots of the Brown-Forsythe *p*-values (blue: nominal *p*-values, green: genomic controlled)

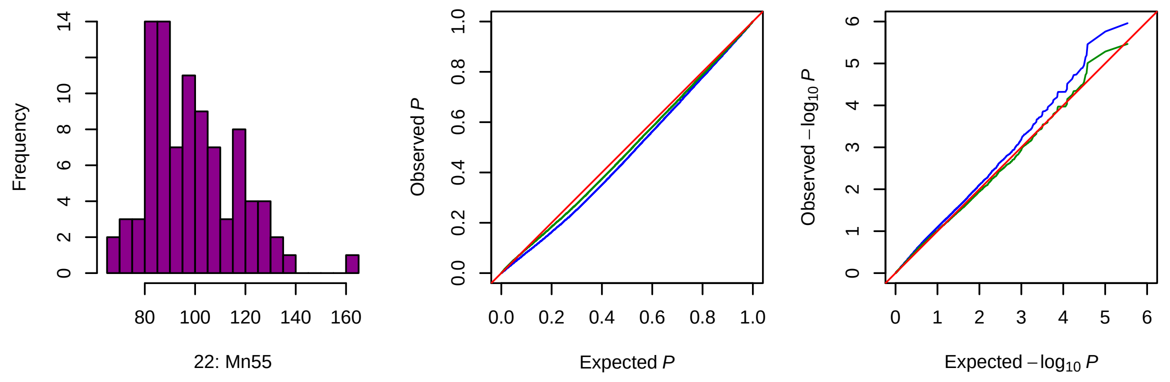

Brown-Forsythe (colored, behind) & Wilcoxon (yellow, in front) results, genomic controlled

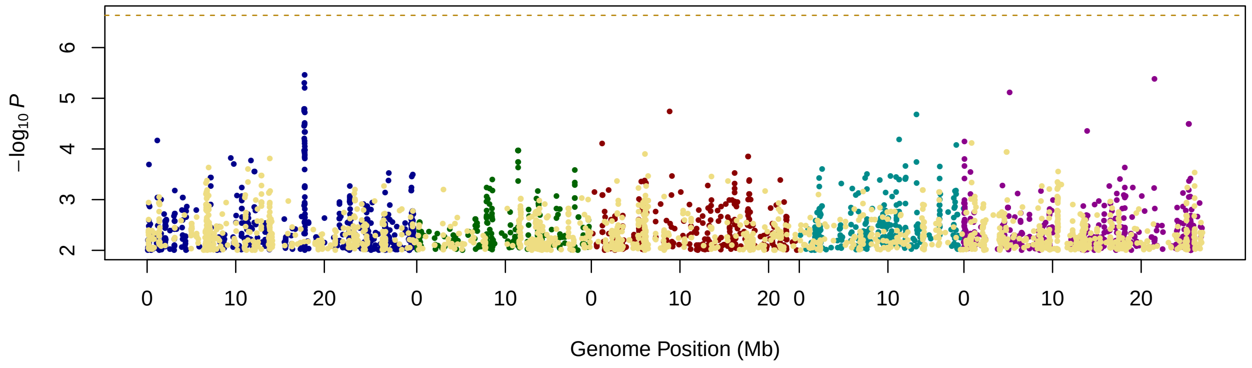

Comparison of Brown-Forsythe & Wilcoxon *p*-values (MAF: minor allele frequency)

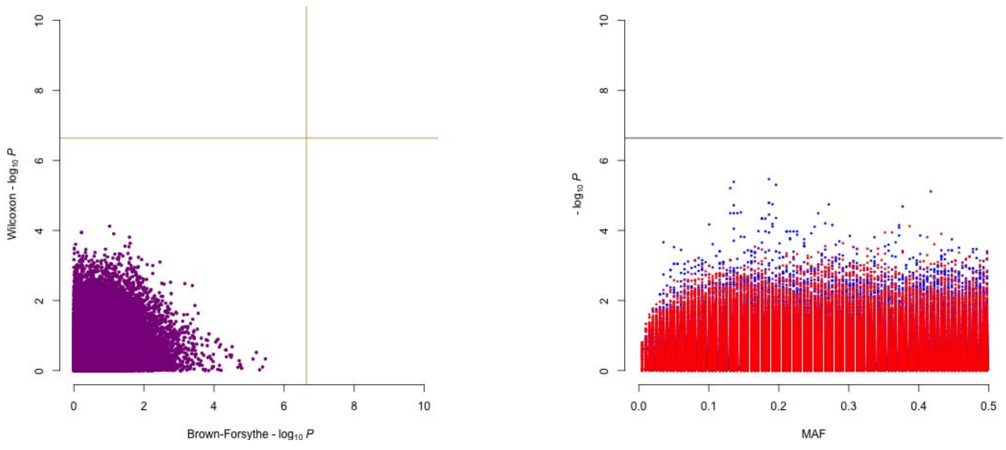

Supplementary Figure B17 - Summary of vGWA results for 22: Mn55

Histogram of the phenotype & QQ plots of the Brown-Forsythe *p*-values (blue: nominal *p*-values, green: genomic controlled)

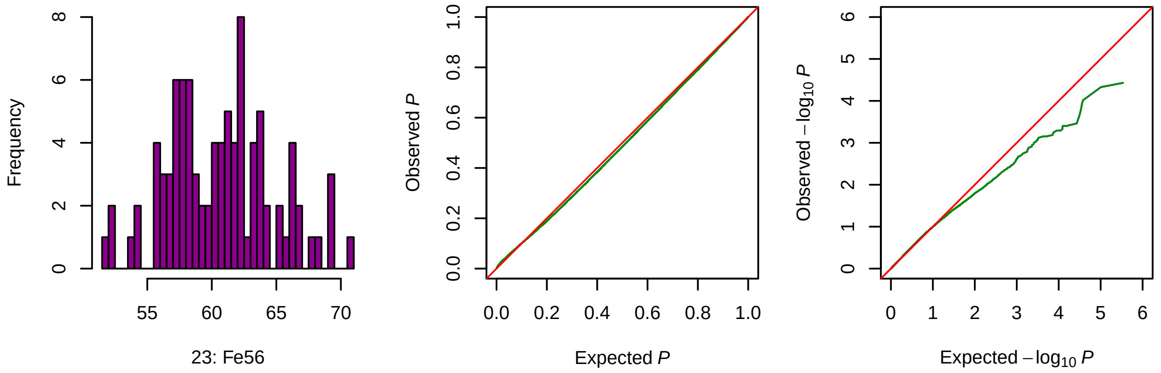

Brown-Forsythe (colored, behind) & Wilcoxon (yellow, in front) results, genomic controlled

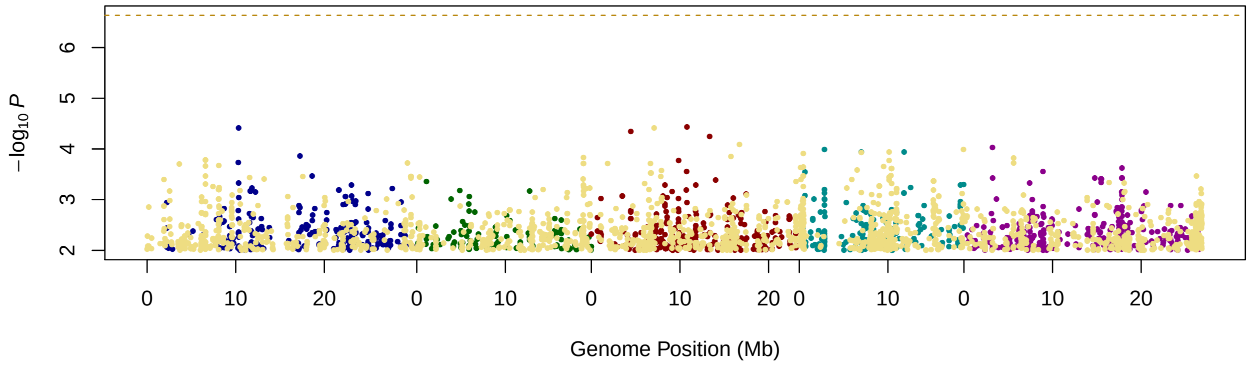

Comparison of Brown-Forsythe & Wilcoxon *p*-values (MAF: minor allele frequency)

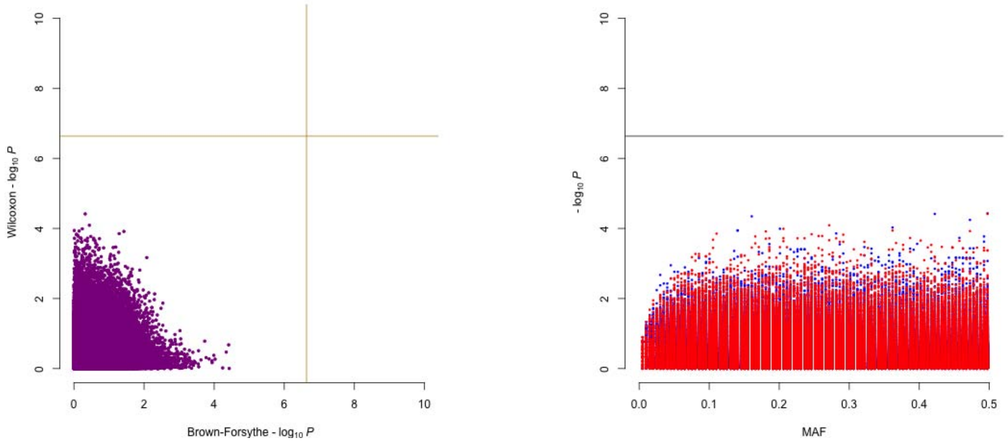

Supplementary Figure B18 - Summary of vGWA results for 23: Fe56

Histogram of the phenotype & QQ plots of the Brown-Forsythe *p*-values (blue: nominal *p*-values, green: genomic controlled)

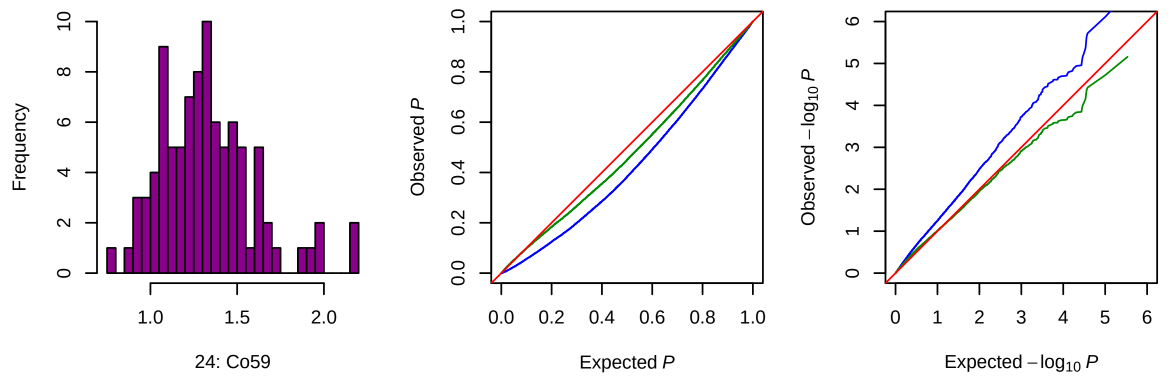

Brown-Forsythe (colored, behind) & Wilcoxon (yellow, in front) results, genomic controlled

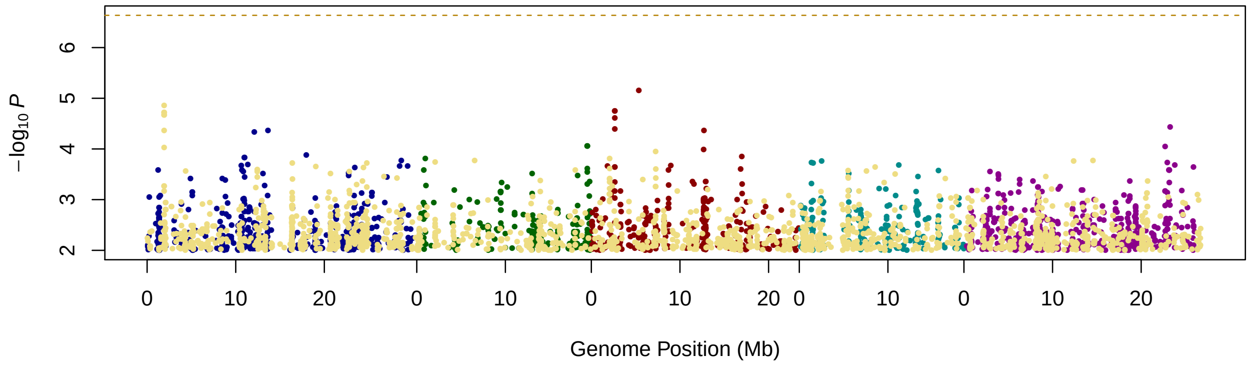

Comparison of Brown-Forsythe & Wilcoxon *p*-values (MAF: minor allele frequency)

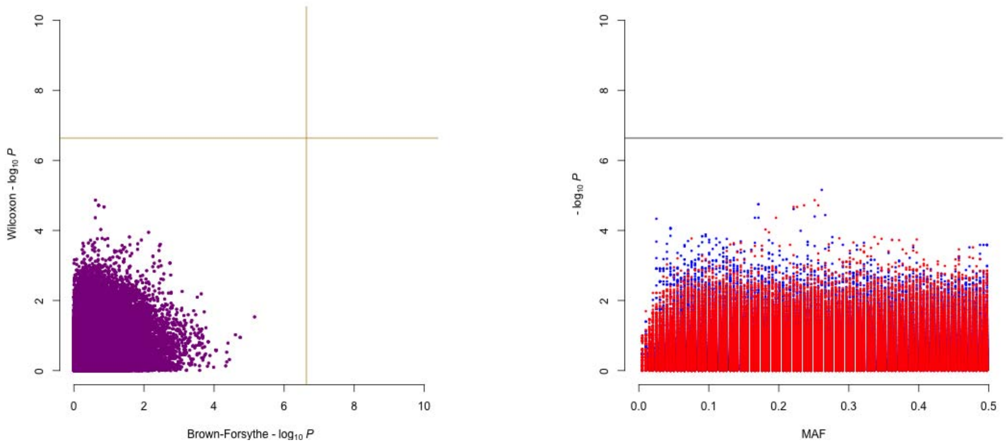

Supplementary Figure B19 - Summary of vGWA results for 24: Co59

Histogram of the phenotype & QQ plots of the Brown-Forsythe *p*-values (blue: nominal *p*-values, green: genomic controlled)

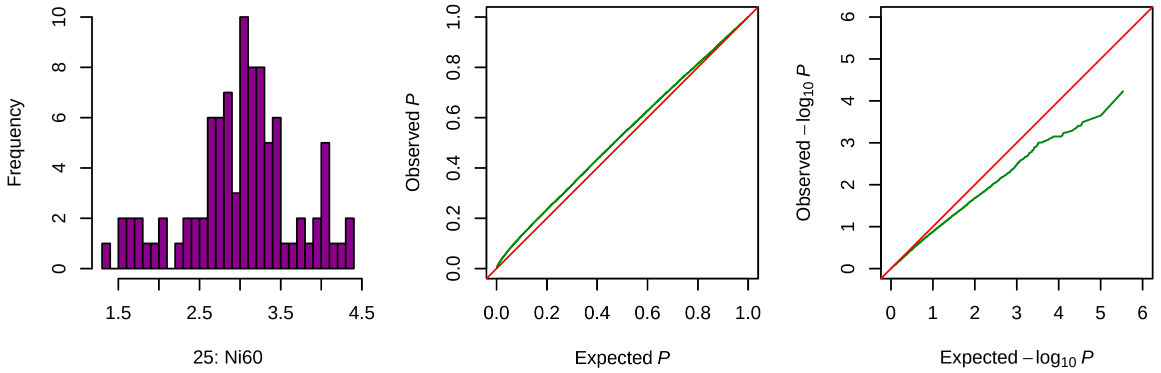

Brown-Forsythe (colored, behind) & Wilcoxon (yellow, in front) results, genomic controlled

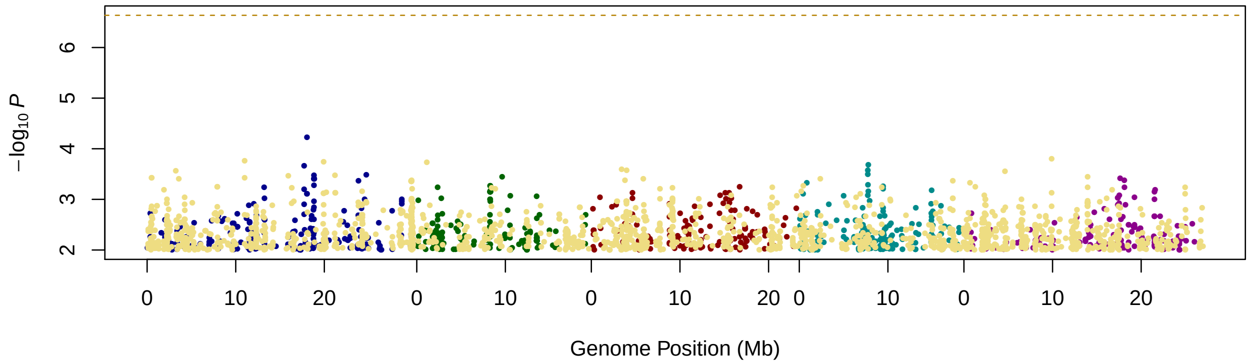

Comparison of Brown-Forsythe & Wilcoxon *p*-values (MAF: minor allele frequency)

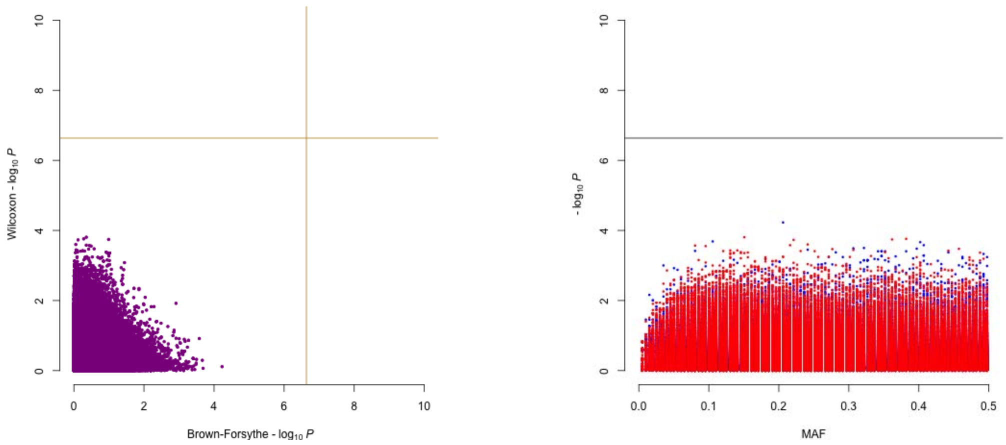

Supplementary Figure B20 - Summary of vGWA results for 25: Ni60

Histogram of the phenotype & QQ plots of the Brown-Forsythe *p*-values (blue: nominal *p*-values, green: genomic controlled)

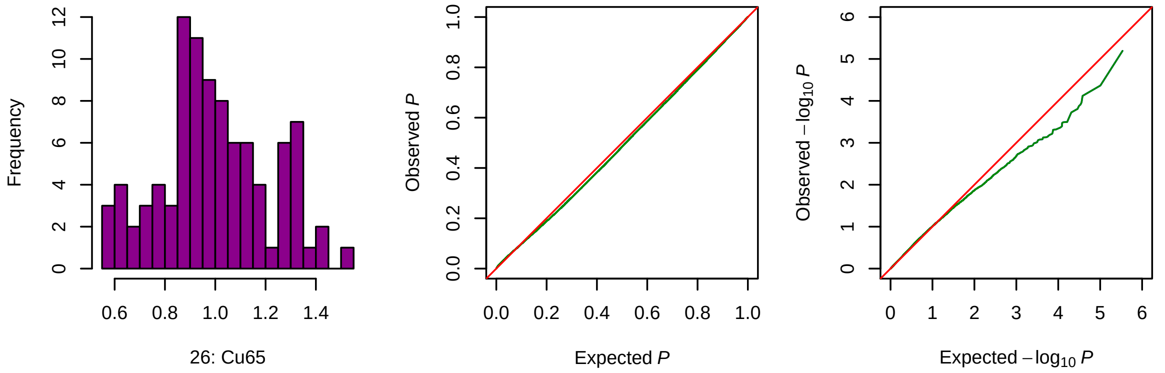

Brown-Forsythe (colored, behind) & Wilcoxon (yellow, in front) results, genomic controlled

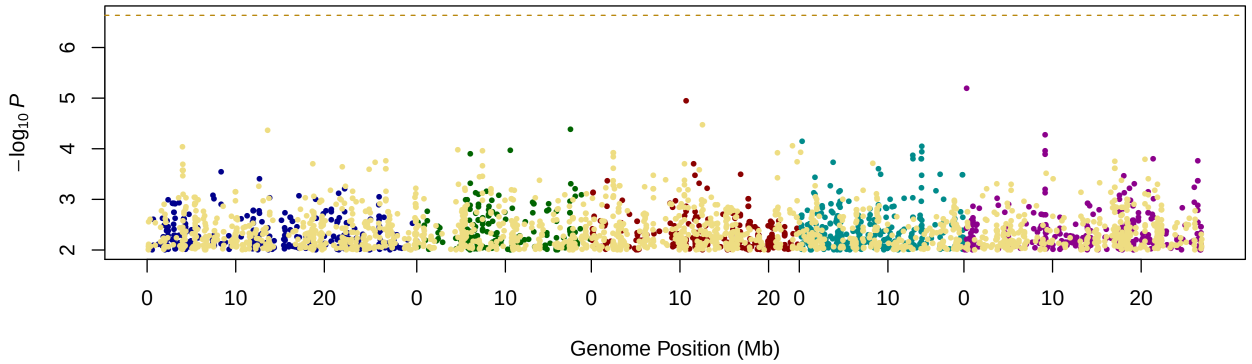

Comparison of Brown-Forsythe & Wilcoxon *p*-values (MAF: minor allele frequency)

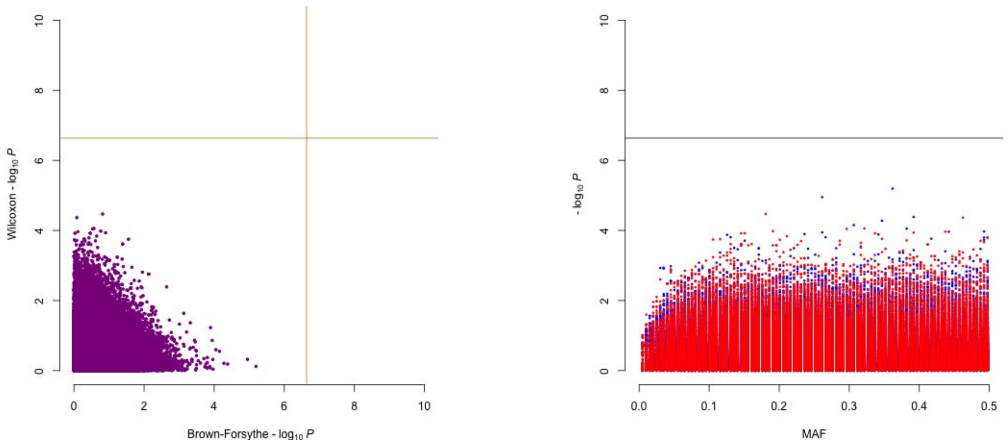

Supplementary Figure B21 - Summary of vGWA results for 26: Cu65

Histogram of the phenotype & QQ plots of the Brown-Forsythe *p*-values (blue: nominal *p*-values, green: genomic controlled)

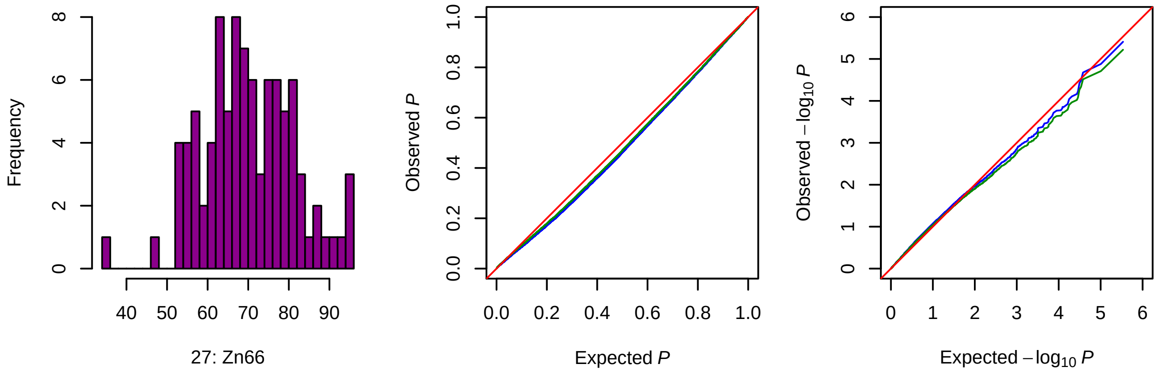

Brown-Forsythe (colored, behind) & Wilcoxon (yellow, in front) results, genomic controlled

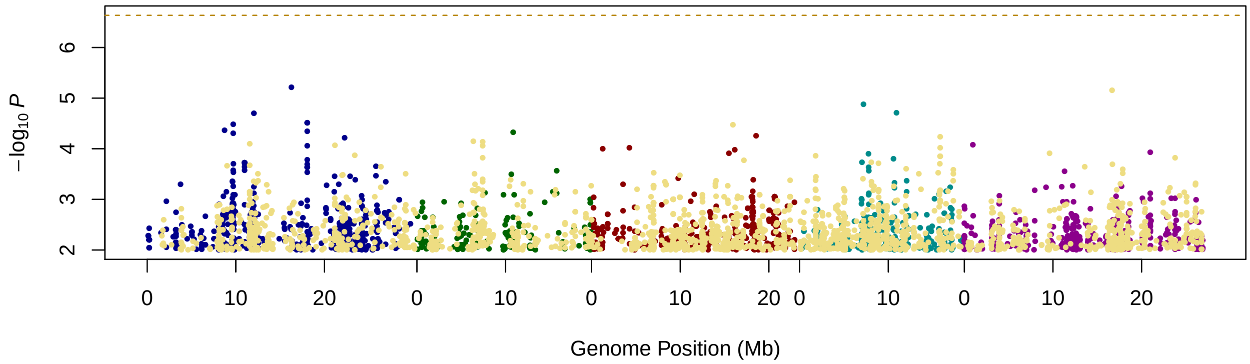

Comparison of Brown-Forsythe & Wilcoxon *p*-values (MAF: minor allele frequency)

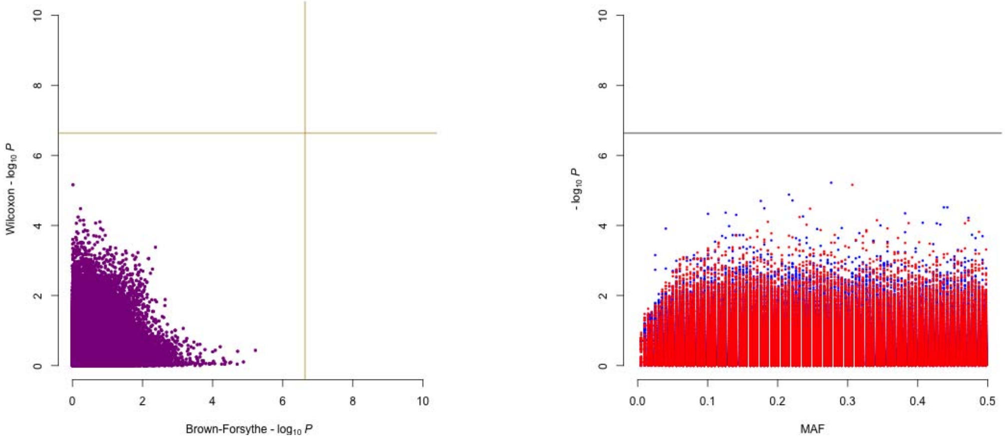

Supplementary Figure B22 - Summary of vGWA results for 27: Zn66

Histogram of the phenotype & QQ plots of the Brown-Forsythe *p*-values (blue: nominal *p*-values, green: genomic controlled)

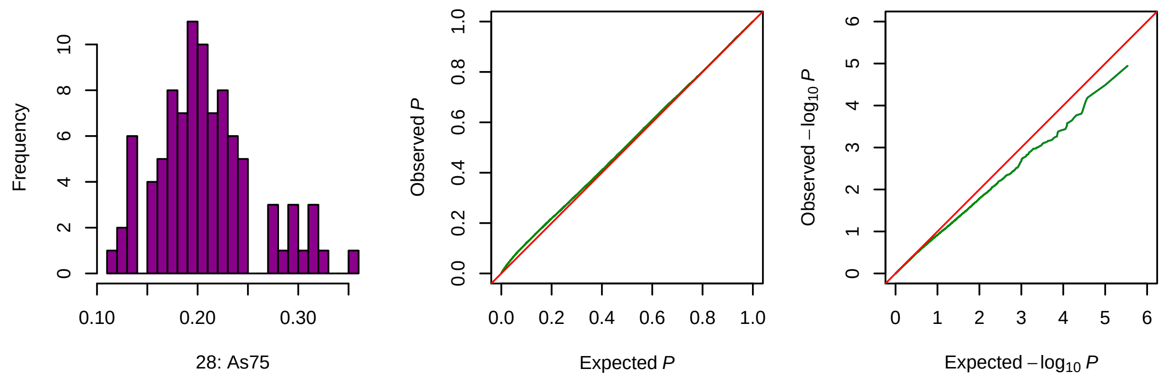

Brown-Forsythe (colored, behind) & Wilcoxon (yellow, in front) results, genomic controlled

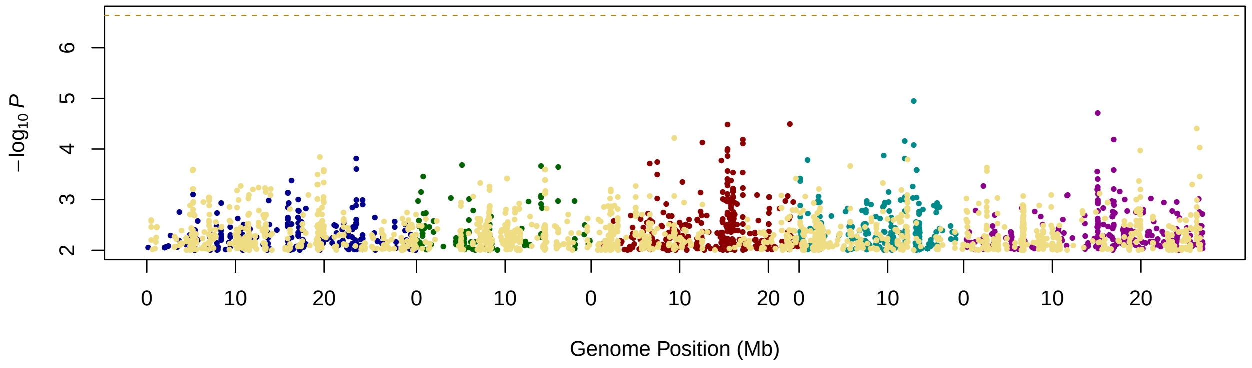

Comparison of Brown-Forsythe & Wilcoxon *p*-values (MAF: minor allele frequency)

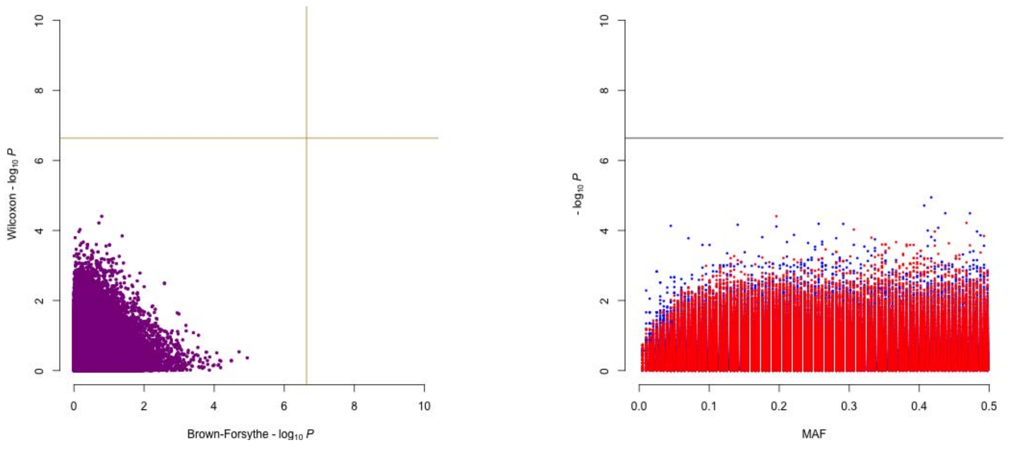

Supplementary Figure B23 - Summary of vGWA results for 28: As75

Histogram of the phenotype & QQ plots of the Brown-Forsythe *p*-values (blue: nominal *p*-values, green: genomic controlled)

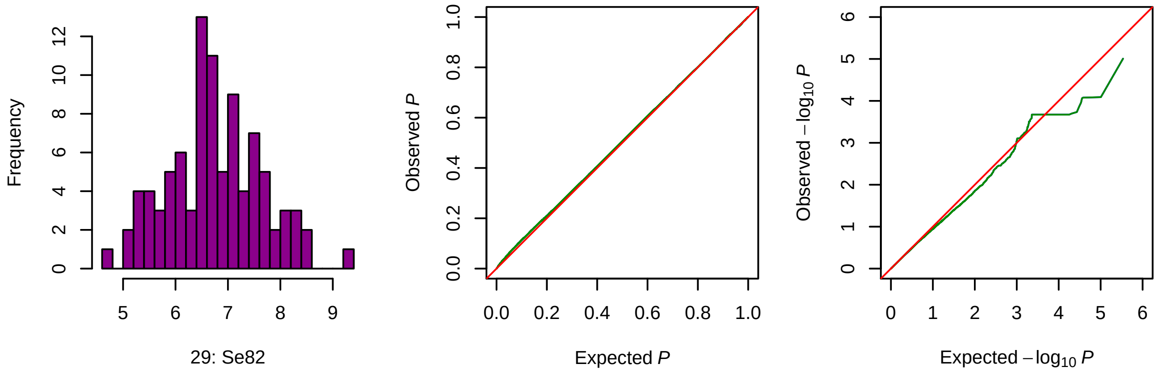

Brown-Forsythe (colored, behind) & Wilcoxon (yellow, in front) results, genomic controlled

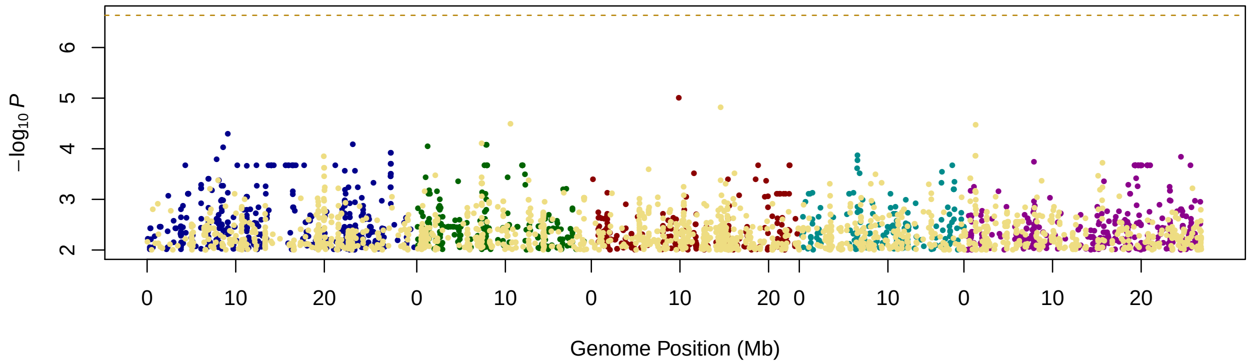

Comparison of Brown-Forsythe & Wilcoxon *p*-values (MAF: minor allele frequency)

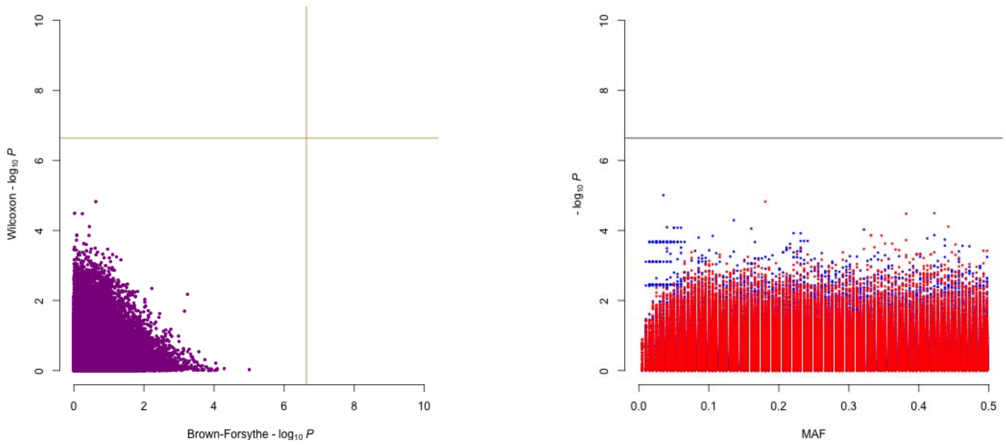

Supplementary Figure B24 - Summary of vGWA results for 29: Se82

Histogram of the phenotype & QQ plots of the Brown-Forsythe *p*-values (blue: nominal *p*-values, green: genomic controlled)

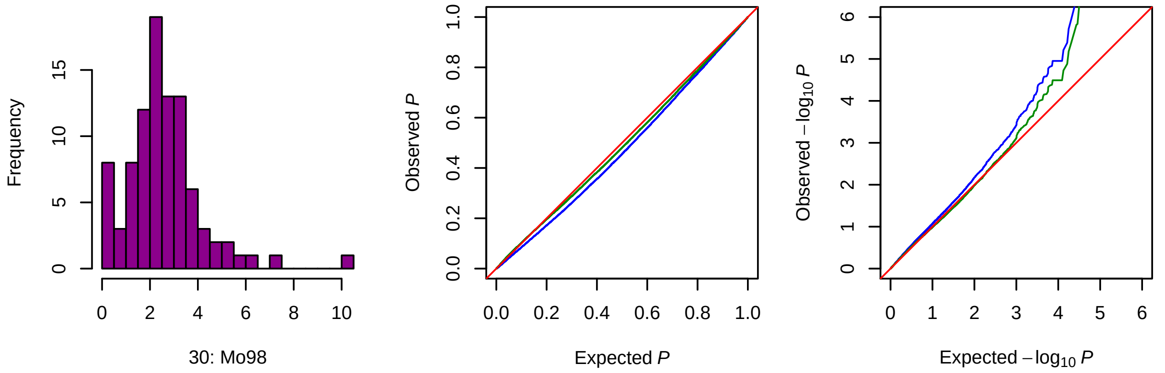

Brown-Forsythe (colored, behind) & Wilcoxon (yellow, in front) results, genomic controlled

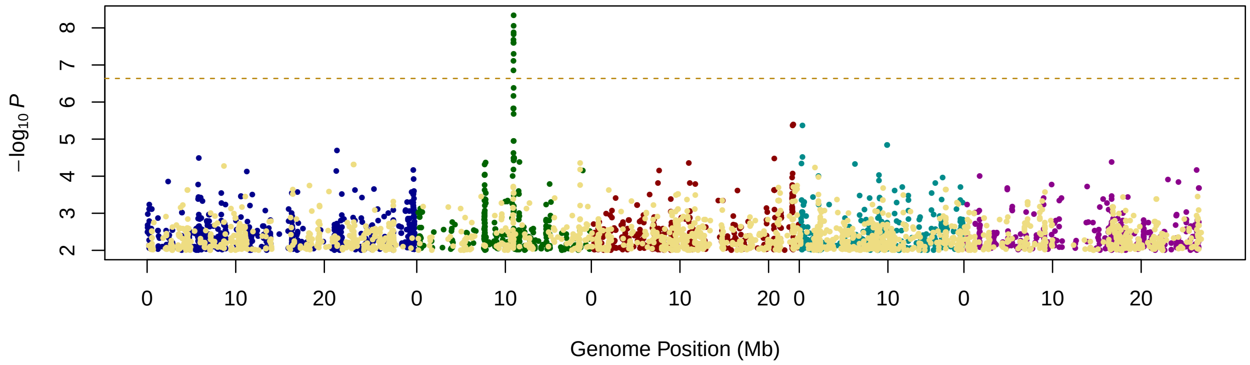

Comparison of Brown-Forsythe & Wilcoxon *p*-values (MAF: minor allele frequency)

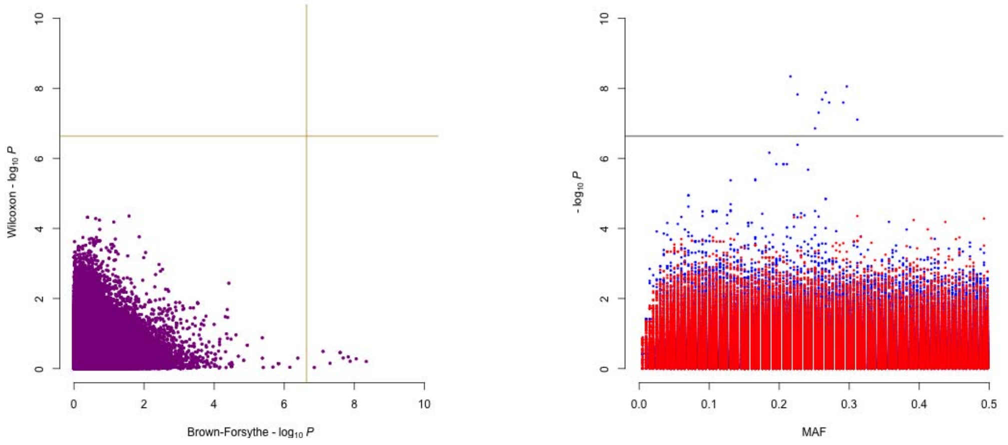

Supplementary Figure B25 - Summary of vGWA results for 30: Mo98

Histogram of the phenotype & QQ plots of the Brown-Forsythe *p*-values (blue: nominal *p*-values, green: genomic controlled)

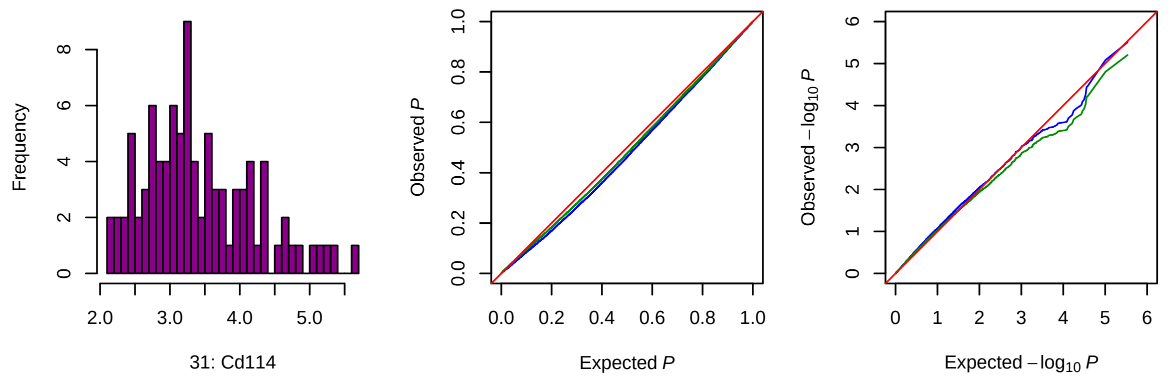

Brown-Forsythe (colored, behind) & Wilcoxon (yellow, in front) results, genomic controlled

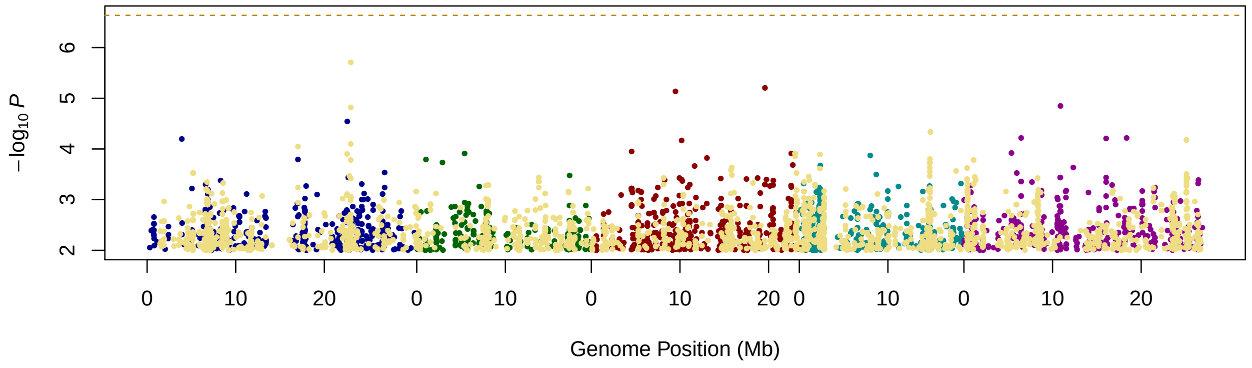

Comparison of Brown-Forsythe & Wilcoxon *p*-values (MAF: minor allele frequency)

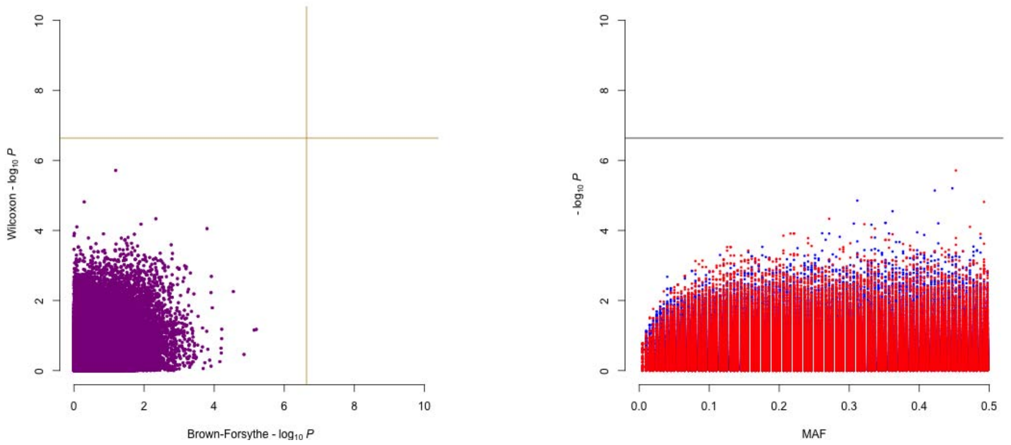

Supplementary Figure B26 - Summary of vGWA results for 31: Cd114

Histogram of the phenotype & QQ plots of the Brown-Forsythe *p*-values (blue: nominal *p*-values, green: genomic controlled)

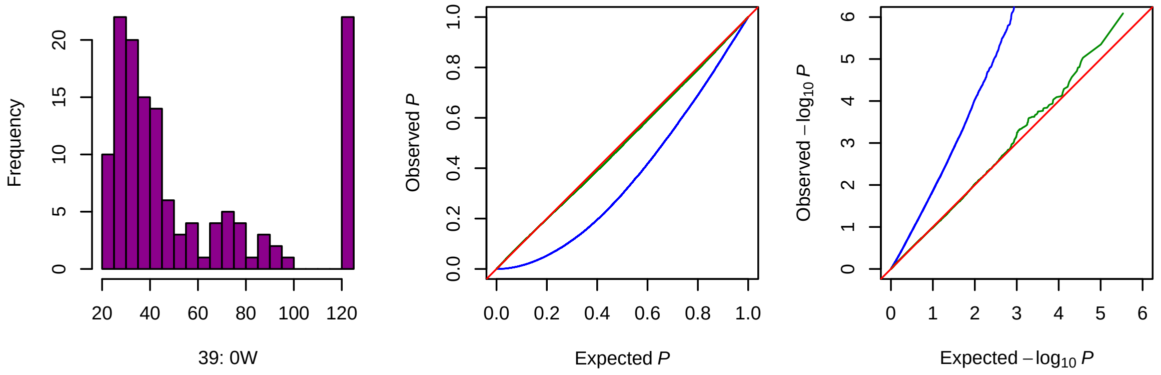

Brown-Forsythe (colored, behind) & Wilcoxon (yellow, in front) results, genomic controlled

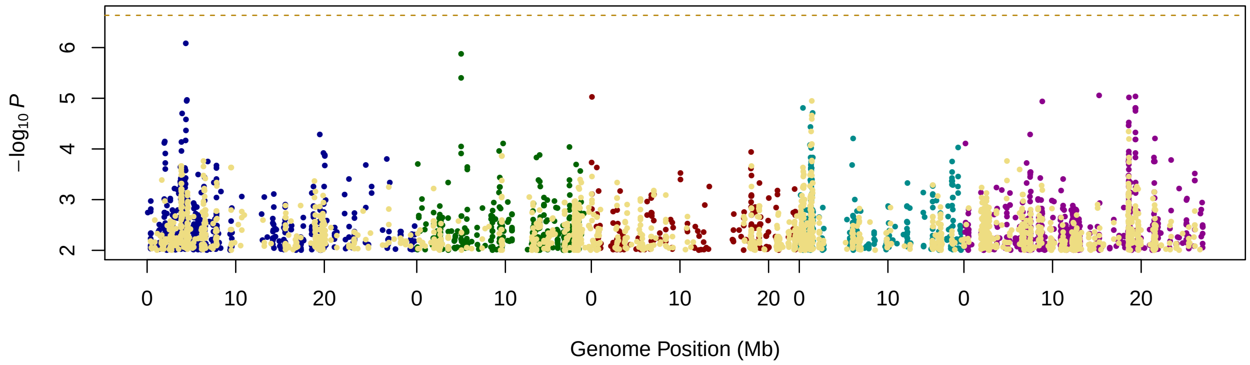

Comparison of Brown-Forsythe & Wilcoxon *p*-values (MAF: minor allele frequency)

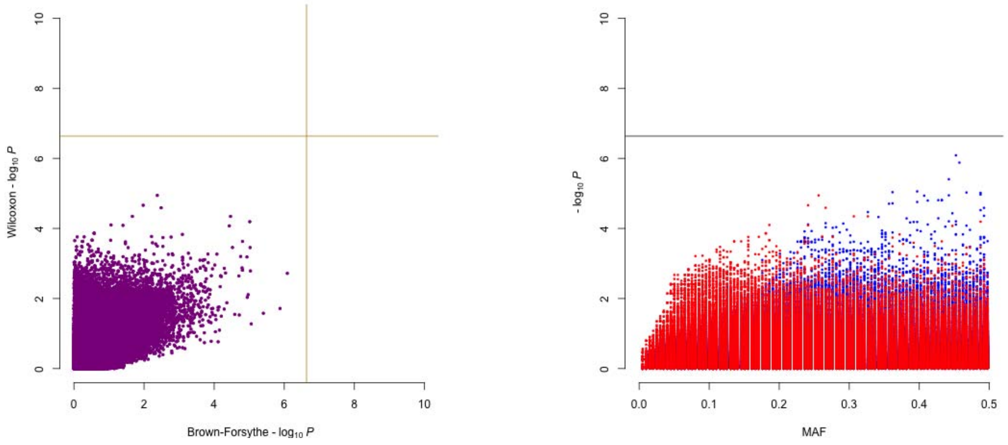

Supplementary Figure B27 - Summary of vGWA results for 39: 0W

Histogram of the phenotype & QQ plots of the Brown-Forsythe *p*-values (blue: nominal *p*-values, green: genomic controlled)

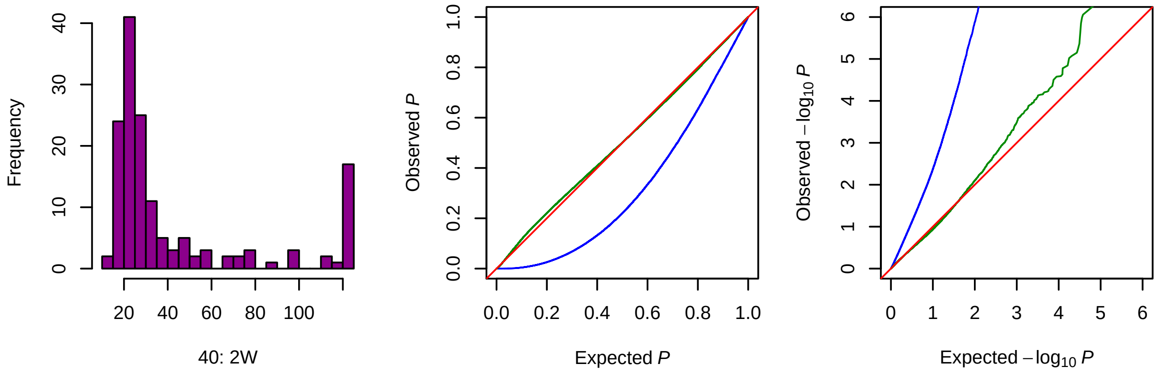

Brown-Forsythe (colored, behind) & Wilcoxon (yellow, in front) results, genomic controlled

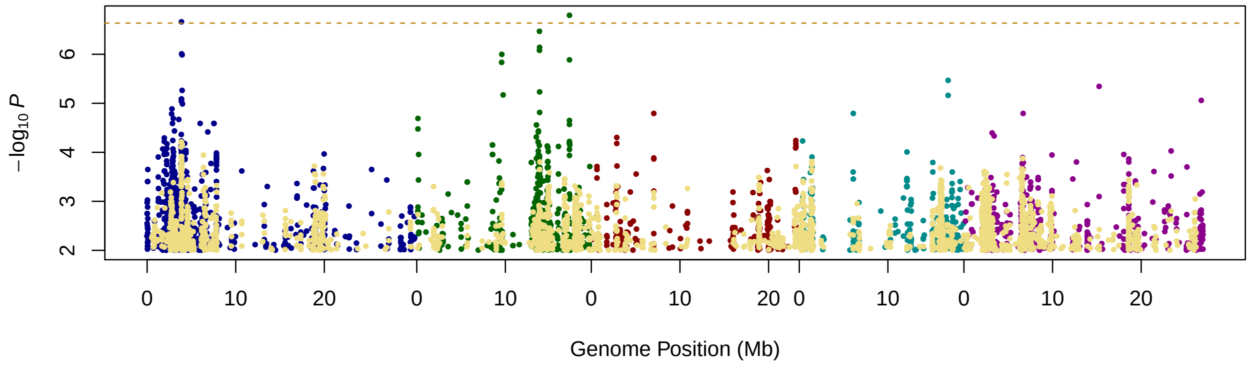

Comparison of Brown-Forsythe & Wilcoxon *p*-values (MAF: minor allele frequency)

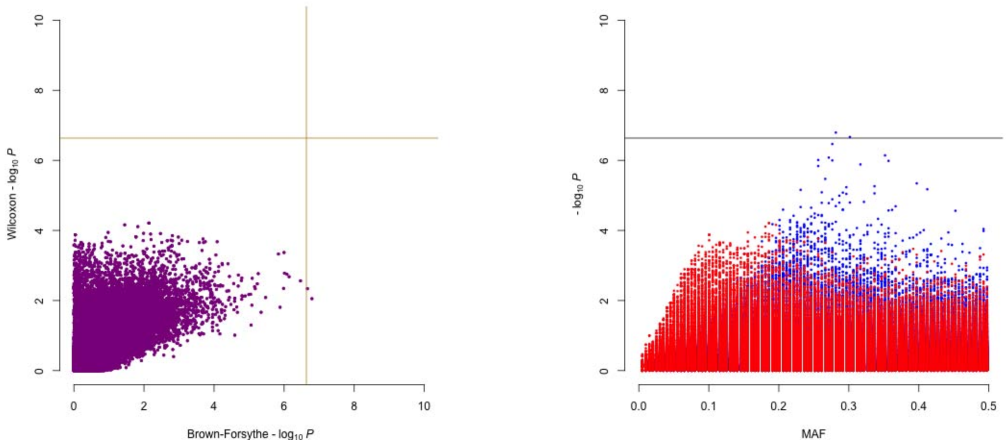

Supplementary Figure B28 - Summary of vGWA results for 40: 2W

Histogram of the phenotype & QQ plots of the Brown-Forsythe *p*-values (blue: nominal *p*-values, green: genomic controlled)

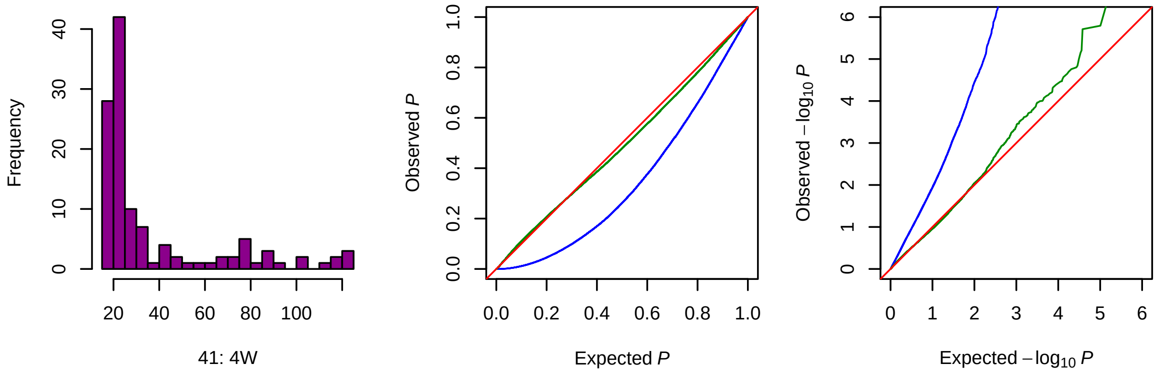

Brown-Forsythe (colored, behind) & Wilcoxon (yellow, in front) results, genomic controlled

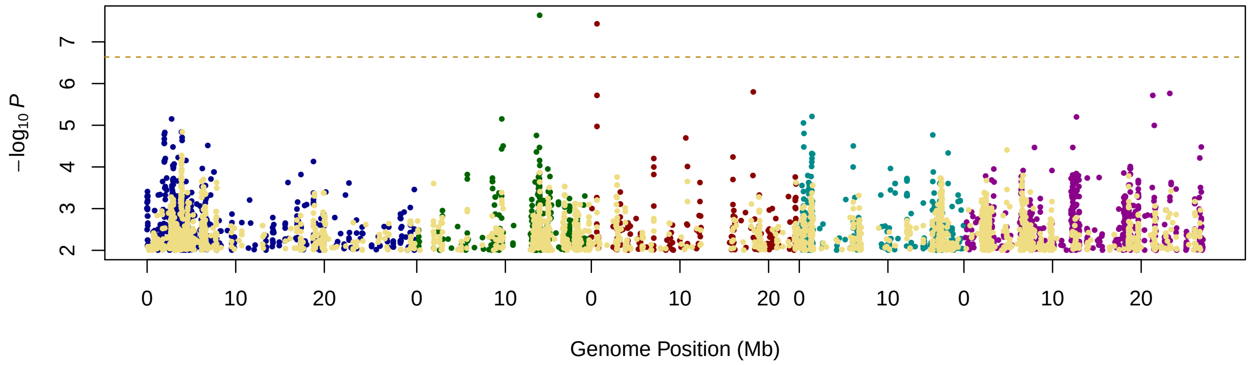

Comparison of Brown-Forsythe & Wilcoxon *p*-values (MAF: minor allele frequency)

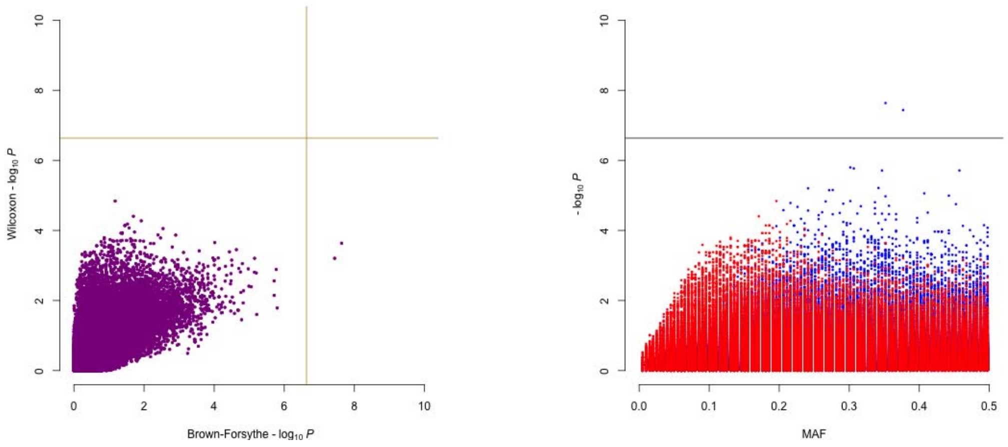

Supplementary Figure B29 - Summary of vGWA results for 41: 4W

Histogram of the phenotype & QQ plots of the Brown-Forsythe *p*-values (blue: nominal *p*-values, green: genomic controlled)

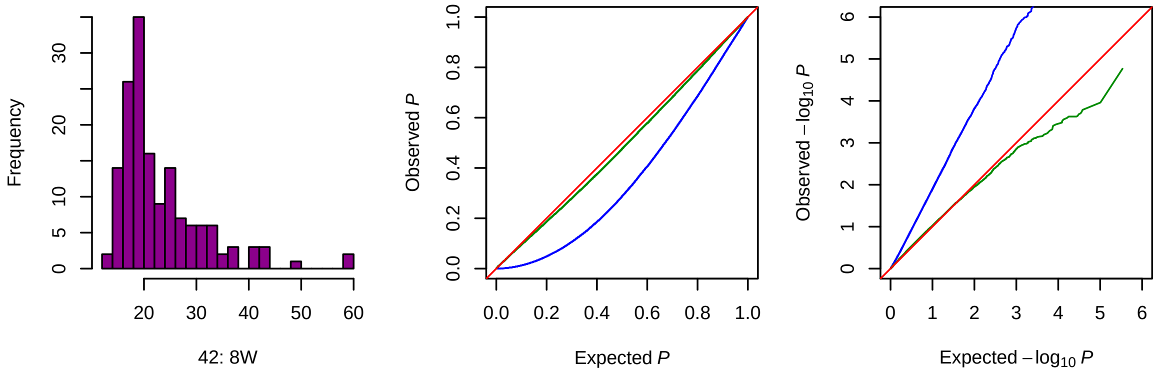

Brown-Forsythe (colored, behind) & Wilcoxon (yellow, in front) results, genomic controlled

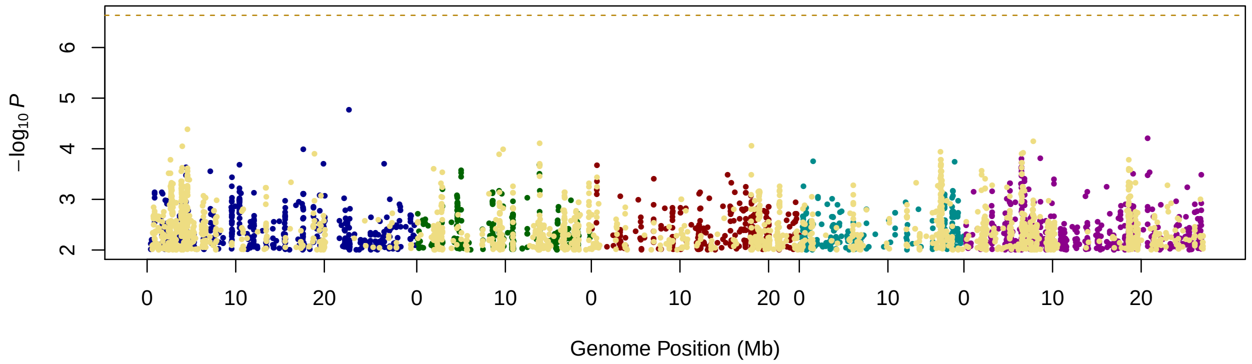

Comparison of Brown-Forsythe & Wilcoxon *p*-values (MAF: minor allele frequency)

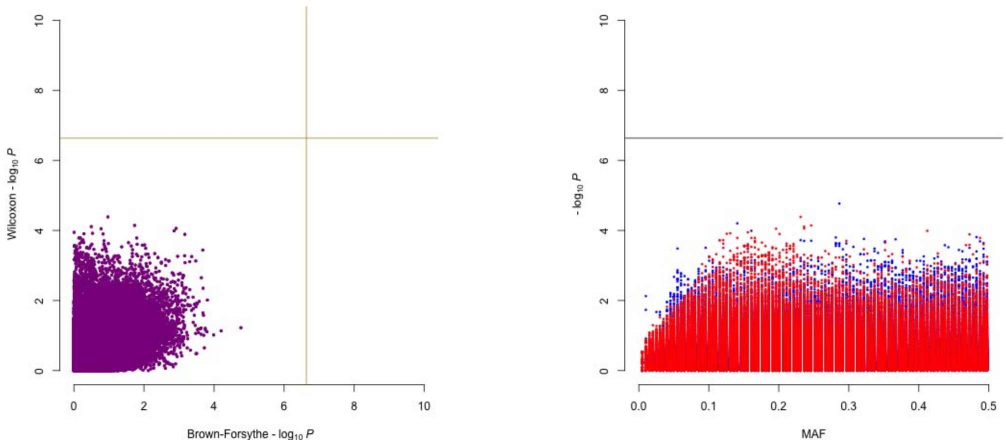

Supplementary Figure B30 - Summary of vGWA results for 42: 8W

Histogram of the phenotype & QQ plots of the Brown-Forsythe *p*-values (blue: nominal *p*-values, green: genomic controlled)

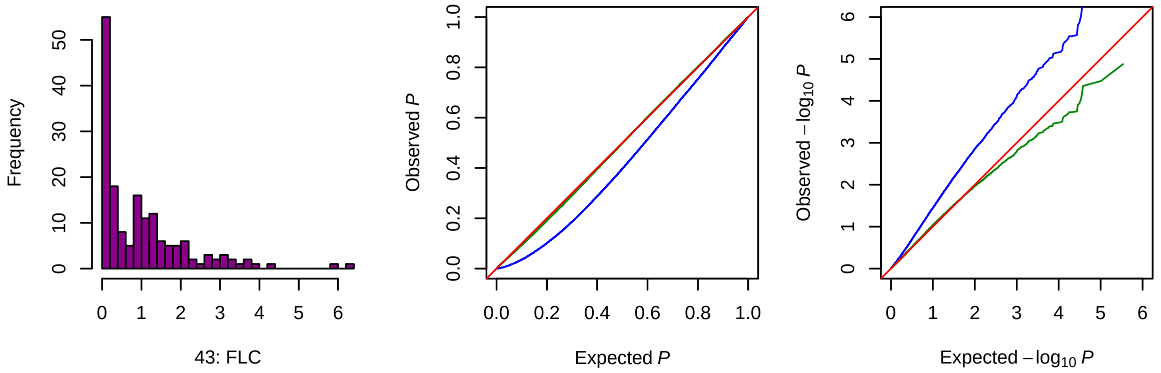

Brown-Forsythe (colored, behind) & Wilcoxon (yellow, in front) results, genomic controlled

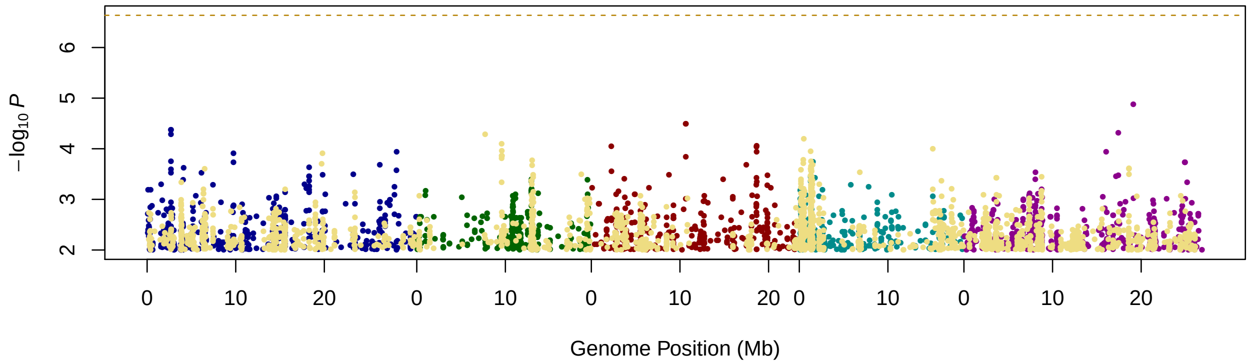

Comparison of Brown-Forsythe & Wilcoxon *p*-values (MAF: minor allele frequency)

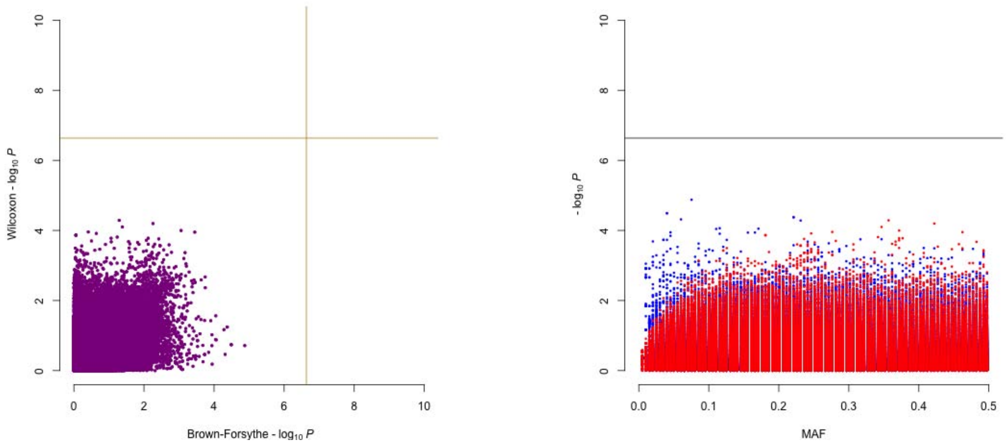

Supplementary Figure B31 - Summary of vGWA results for 43: FLC

Histogram of the phenotype & QQ plots of the Brown-Forsythe *p*-values (blue: nominal *p*-values, green: genomic controlled)

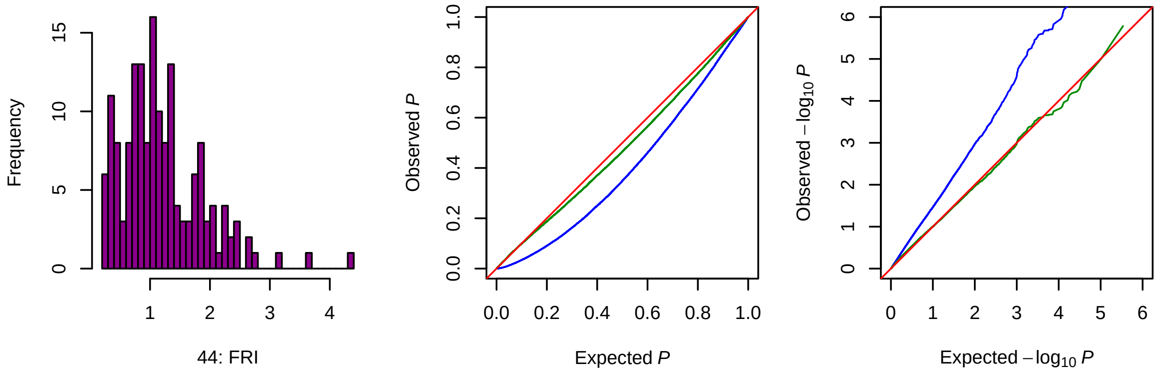

Brown-Forsythe (colored, behind) & Wilcoxon (yellow, in front) results, genomic controlled

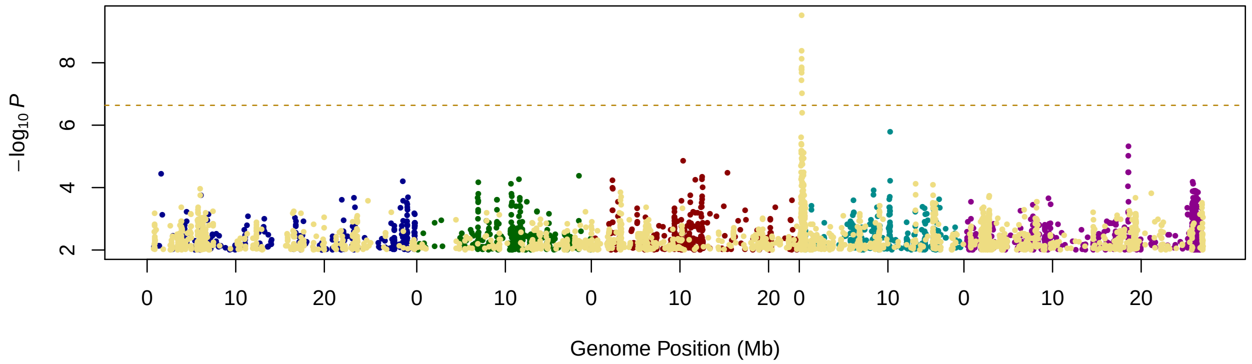

Comparison of Brown-Forsythe & Wilcoxon *p*-values (MAF: minor allele frequency)

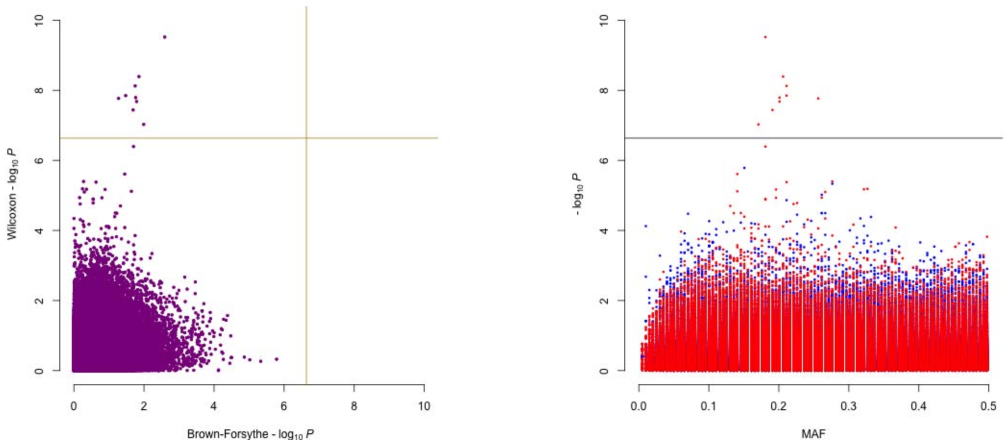

Supplementary Figure B32 - Summary of vGWA results for 44: FRI

Histogram of the phenotype & QQ plots of the Brown-Forsythe *p*-values (blue: nominal *p*-values, green: genomic controlled)

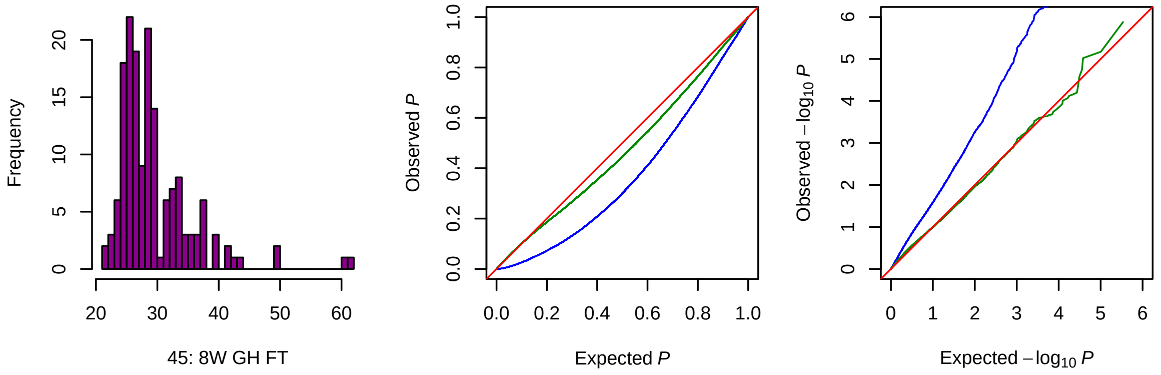

Brown-Forsythe (colored, behind) & Wilcoxon (yellow, in front) results, genomic controlled

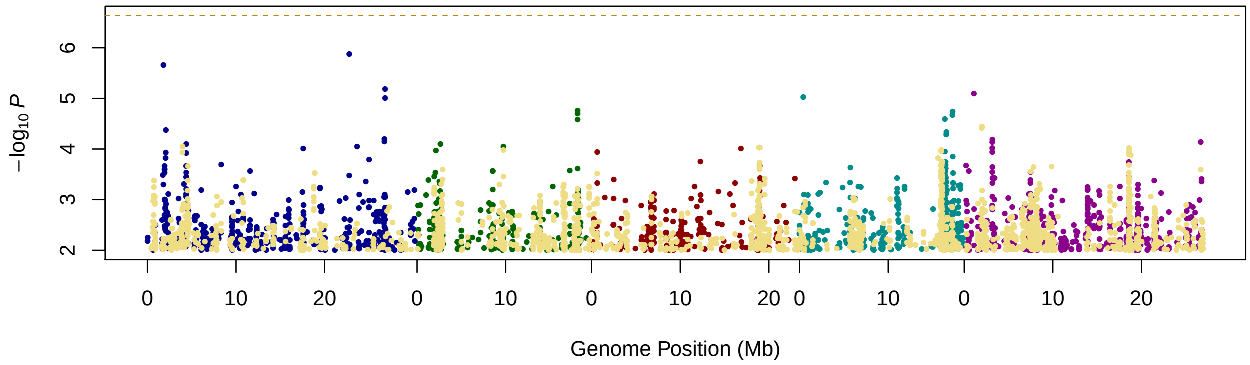

Comparison of Brown-Forsythe & Wilcoxon *p*-values (MAF: minor allele frequency)

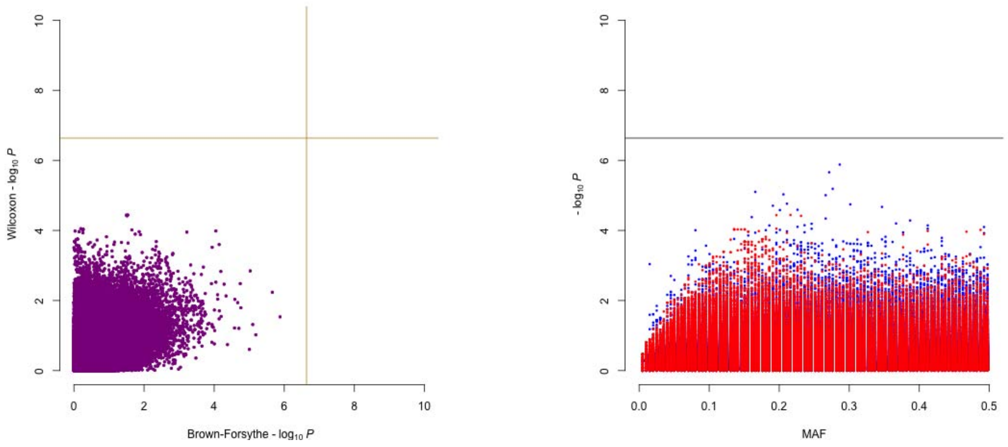

Supplementary Figure B33 - Summary of vGWA results for 45: 8W GH FT

Histogram of the phenotype & QQ plots of the Brown-Forsythe *p*-values (blue: nominal *p*-values, green: genomic controlled)

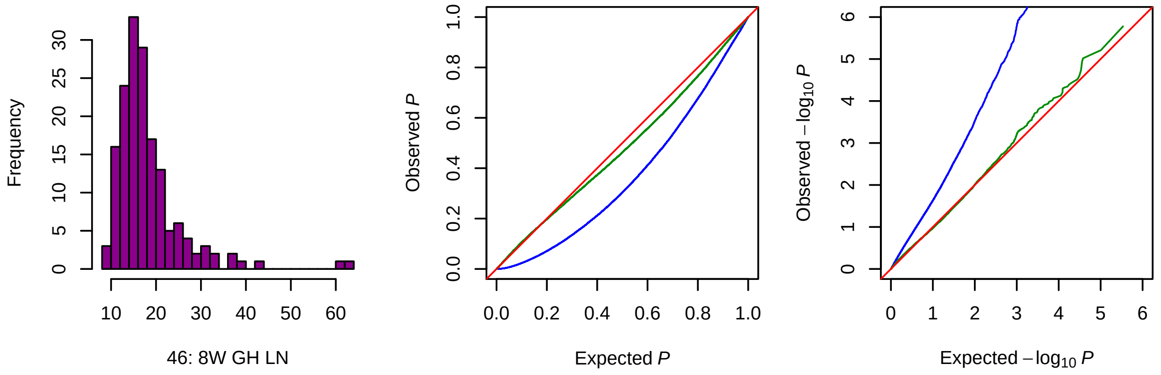

Brown-Forsythe (colored, behind) & Wilcoxon (yellow, in front) results, genomic controlled

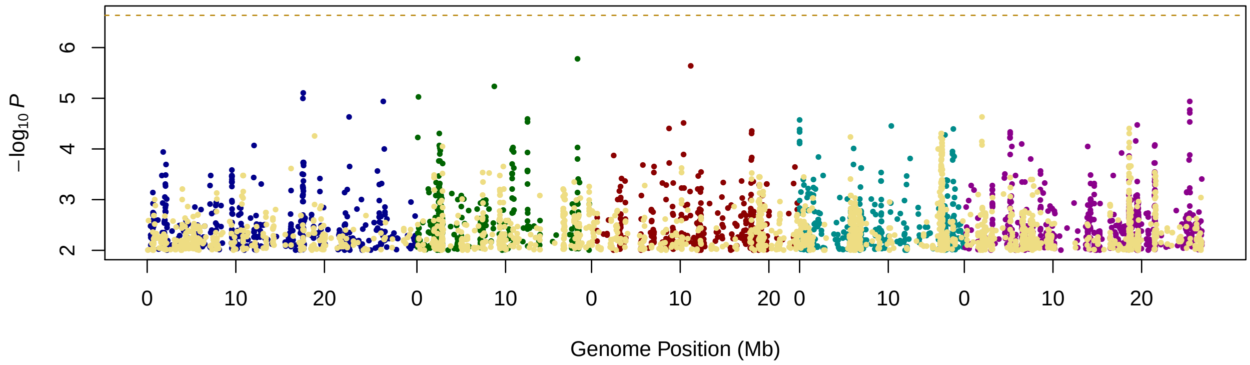

Comparison of Brown-Forsythe & Wilcoxon *p*-values (MAF: minor allele frequency)

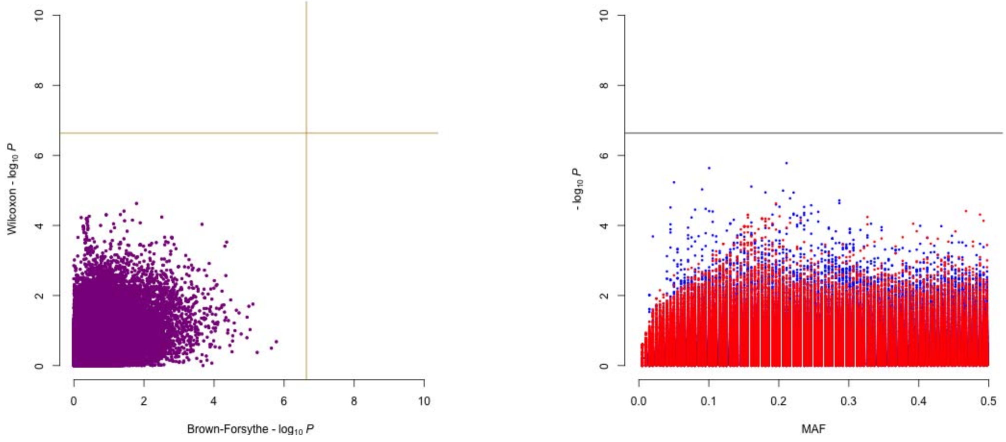

Supplementary Figure B34 - Summary of vGWA results for 46: 8W GH LN

Histogram of the phenotype & QQ plots of the Brown-Forsythe *p*-values (blue: nominal *p*-values, green: genomic controlled)

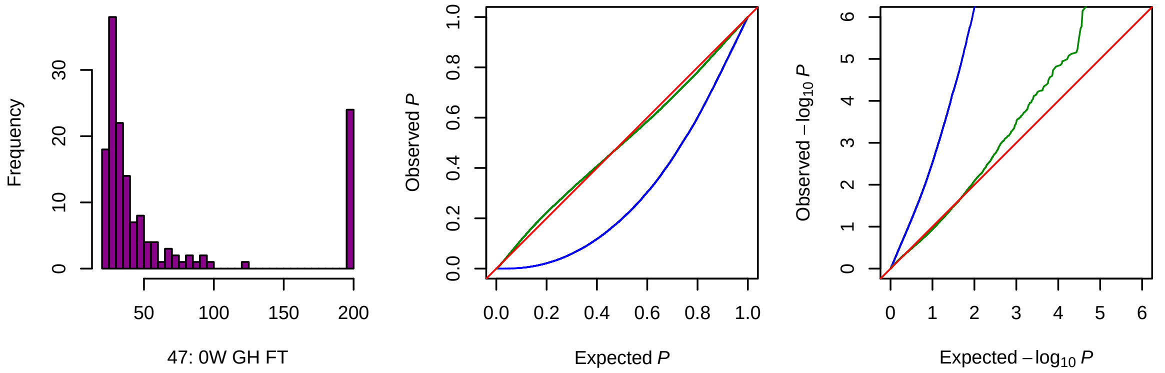

Brown-Forsythe (colored, behind) & Wilcoxon (yellow, in front) results, genomic controlled

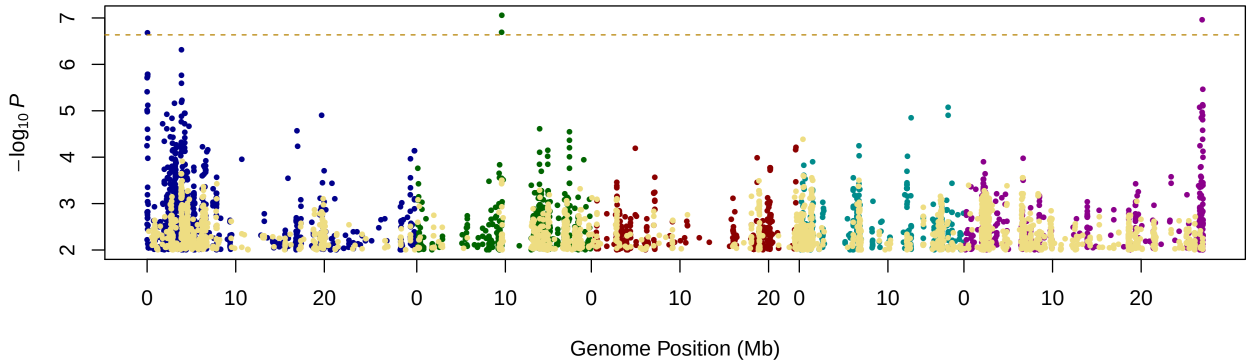

Comparison of Brown-Forsythe & Wilcoxon *p*-values (MAF: minor allele frequency)

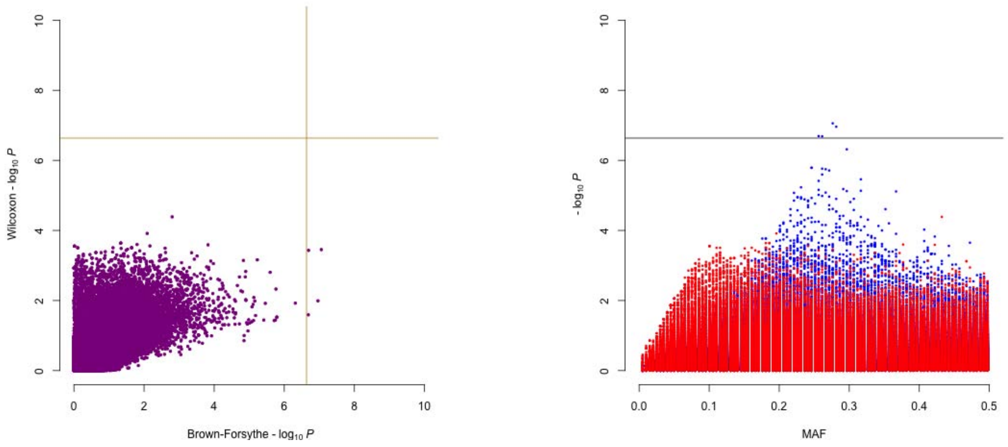

Supplementary Figure B35 - Summary of vGWA results for 47: 0W GH FT

Histogram of the phenotype & QQ plots of the Brown-Forsythe *p*-values (blue: nominal *p*-values, green: genomic controlled)

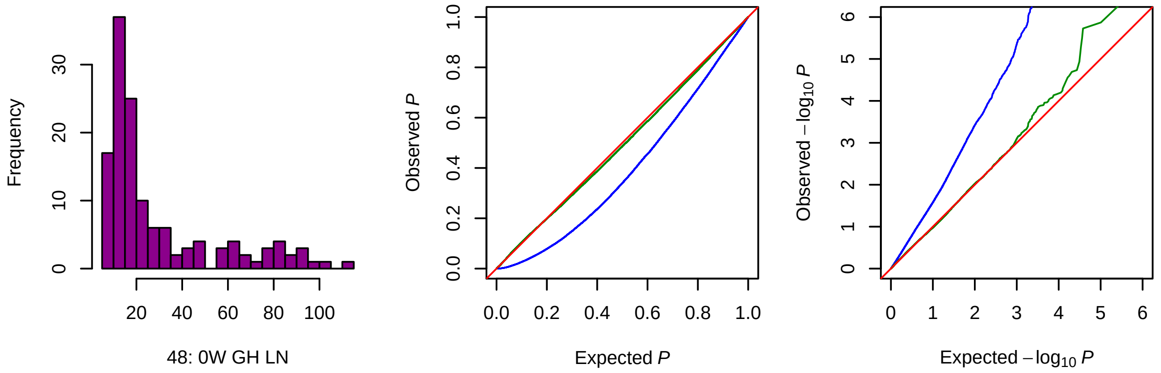

Brown-Forsythe (colored, behind) & Wilcoxon (yellow, in front) results, genomic controlled

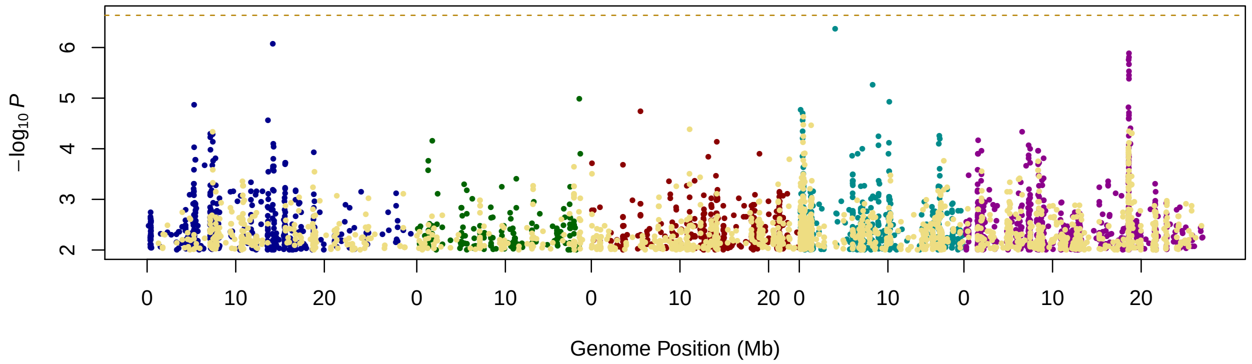

Comparison of Brown-Forsythe & Wilcoxon *p*-values (MAF: minor allele frequency)

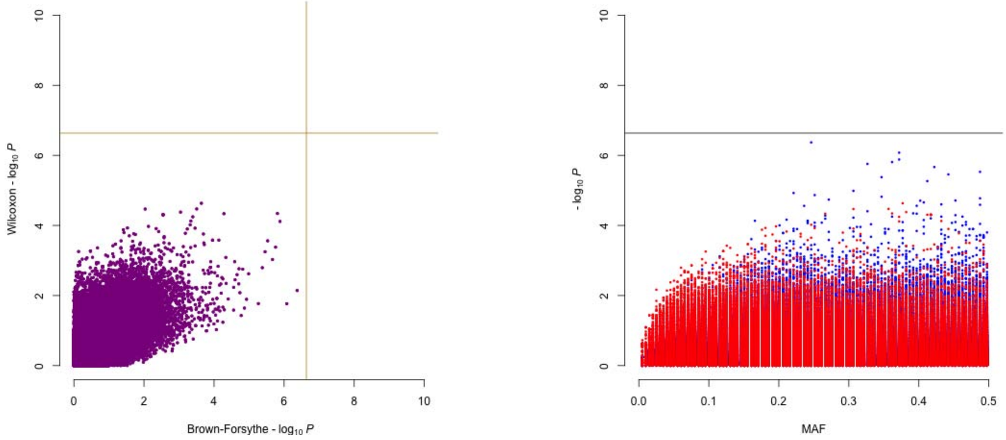

Supplementary Figure B36 - Summary of vGWA results for 48: 0W GH LN

Histogram of the phenotype & QQ plots of the Brown-Forsythe *p*-values (blue: nominal *p*-values, green: genomic controlled)

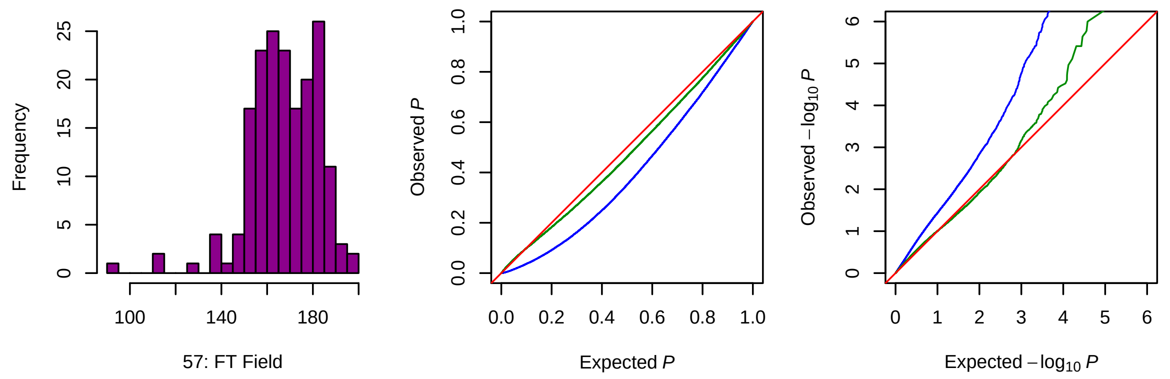

Brown-Forsythe (colored, behind) & Wilcoxon (yellow, in front) results, genomic controlled

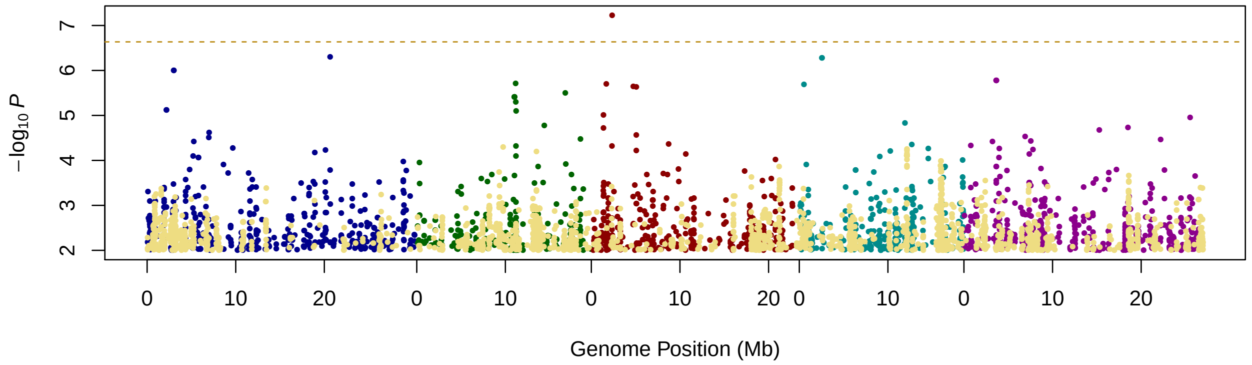

Comparison of Brown-Forsythe & Wilcoxon *p*-values (MAF: minor allele frequency)

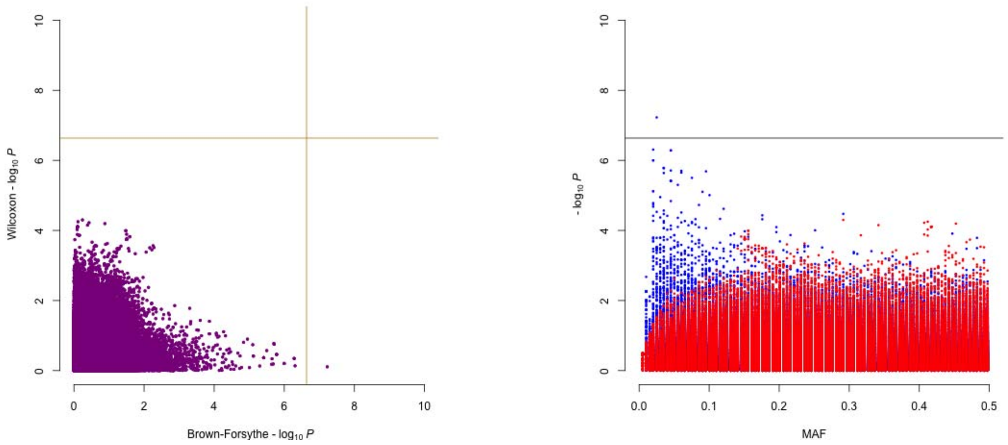

Supplementary Figure B37 - Summary of vGWA results for 57: FT Field

Histogram of the phenotype & QQ plots of the Brown-Forsythe *p*-values (blue: nominal *p*-values, green: genomic controlled)

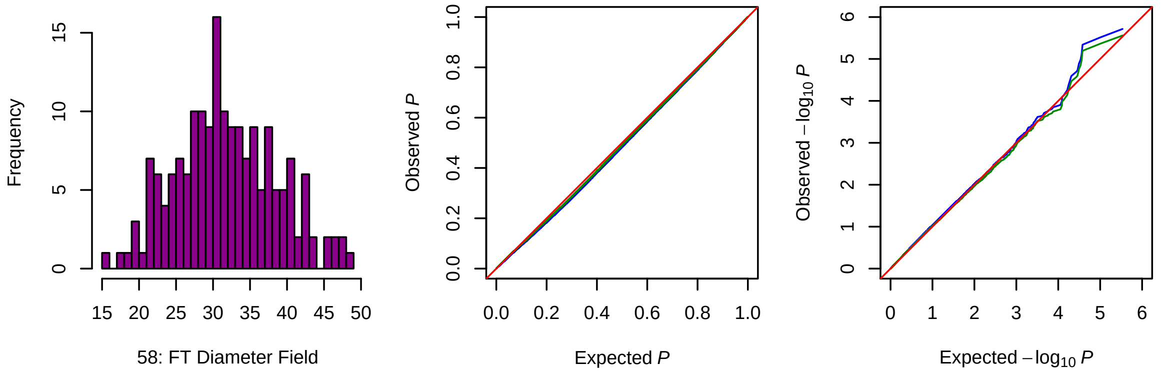

Brown-Forsythe (colored, behind) & Wilcoxon (yellow, in front) results, genomic controlled

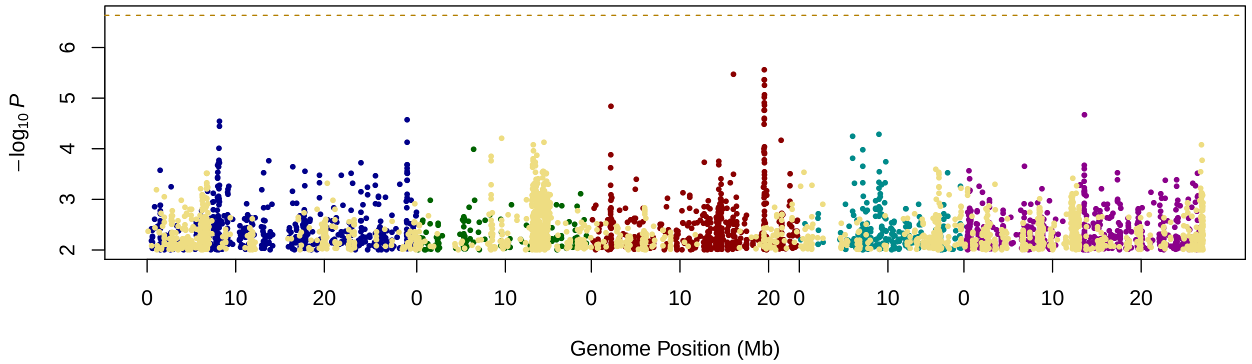

Comparison of Brown-Forsythe & Wilcoxon *p*-values (MAF: minor allele frequency)

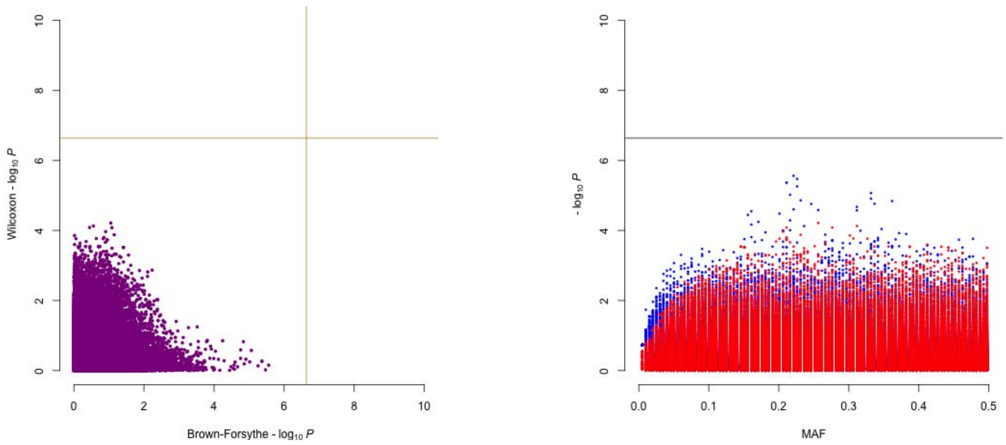

Supplementary Figure B38 - Summary of vGWA results for 58: FT Diameter Field

Histogram of the phenotype & QQ plots of the Brown-Forsythe *p*-values (blue: nominal *p*-values, green: genomic controlled)

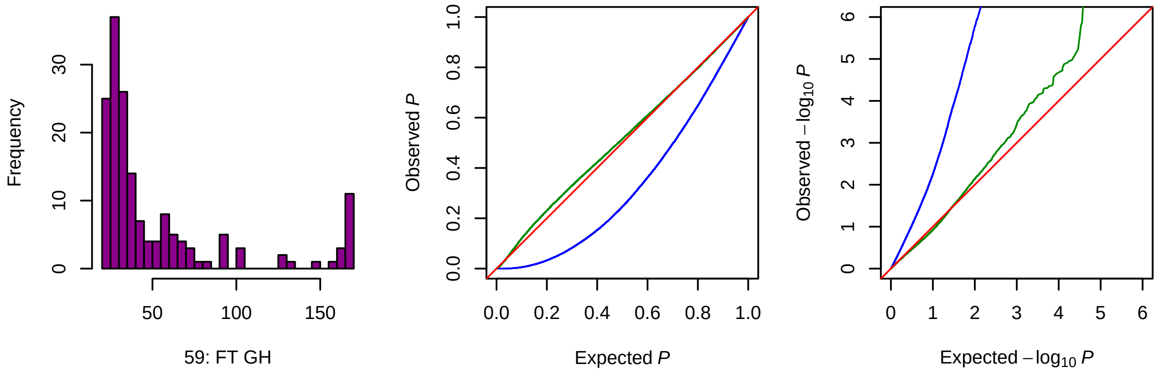

Brown-Forsythe (colored, behind) & Wilcoxon (yellow, in front) results, genomic controlled

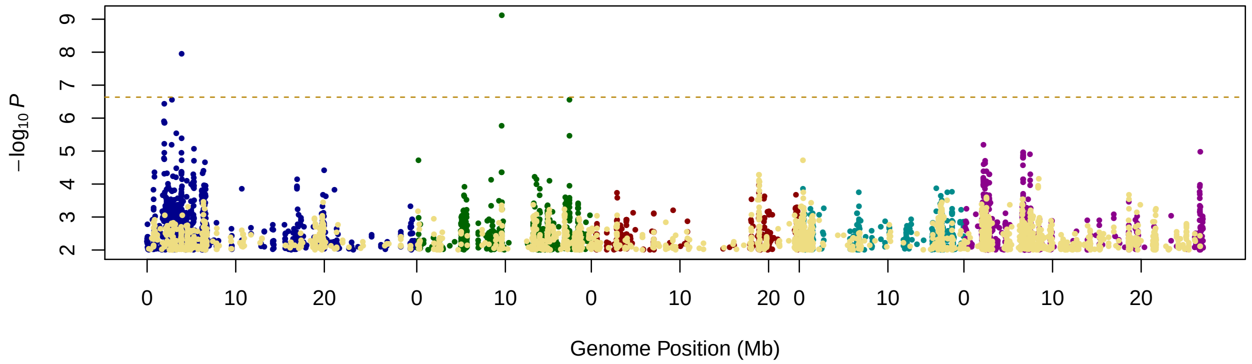

Comparison of Brown-Forsythe & Wilcoxon *p*-values (MAF: minor allele frequency)

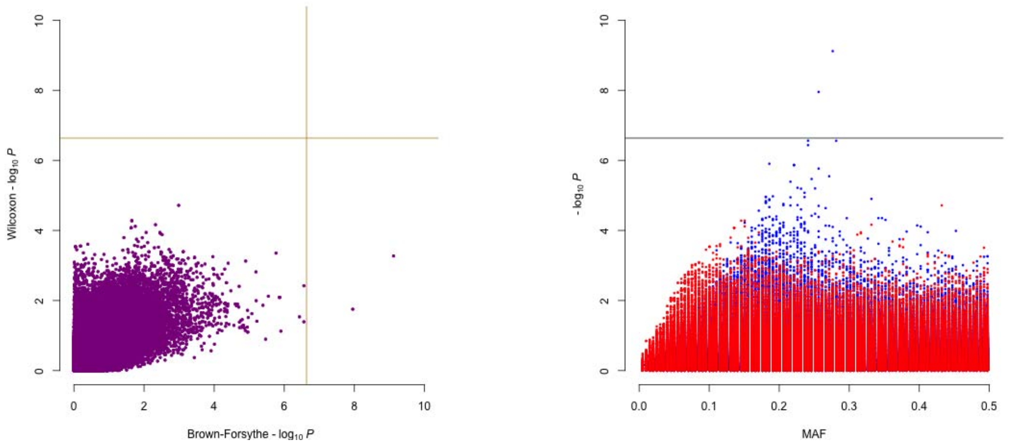

Supplementary Figure B39 - Summary of vGWA results for 59: FT GH

Histogram of the phenotype & QQ plots of the Brown-Forsythe *p*-values (blue: nominal *p*-values, green: genomic controlled)

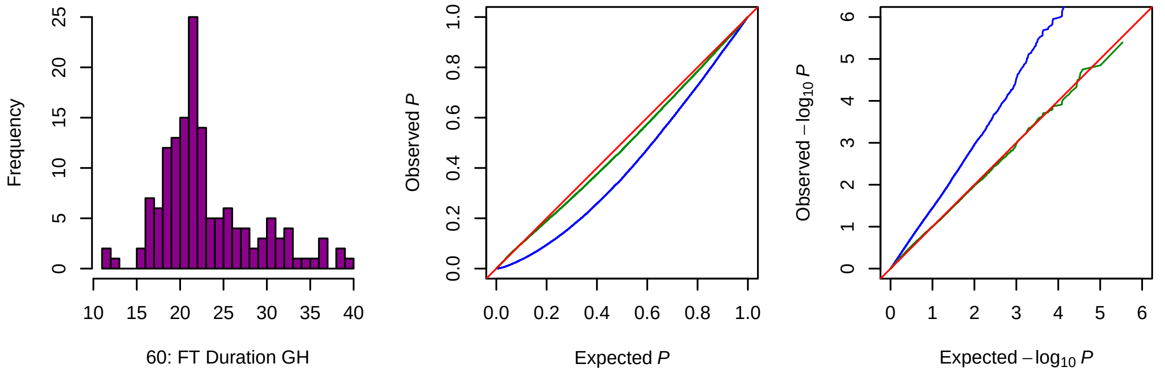

Brown-Forsythe (colored, behind) & Wilcoxon (yellow, in front) results, genomic controlled

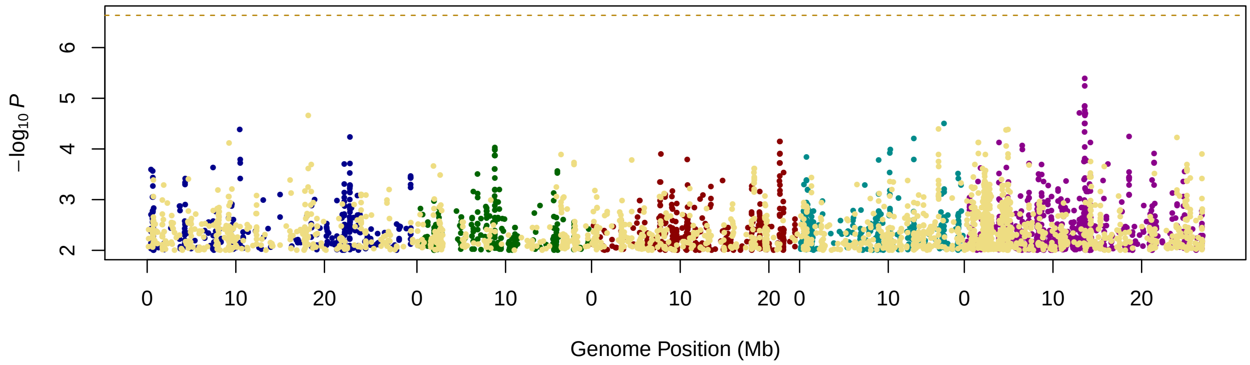

Comparison of Brown-Forsythe & Wilcoxon *p*-values (MAF: minor allele frequency)

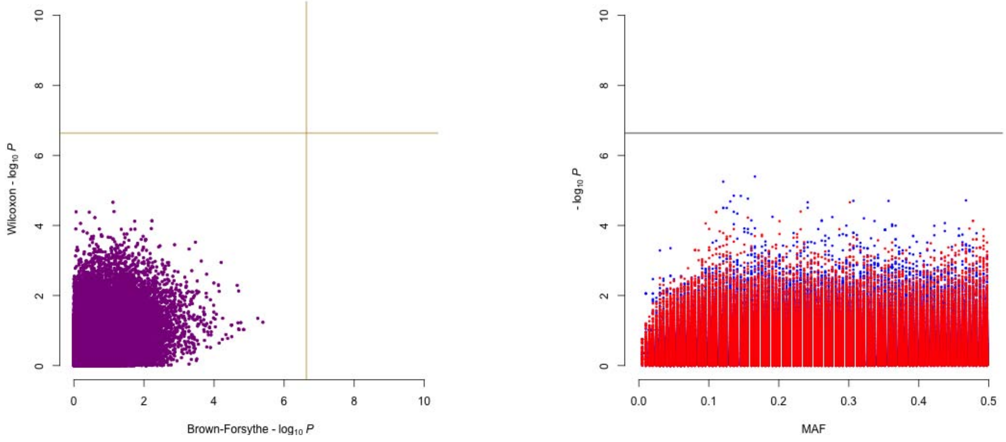

Supplementary Figure B40 - Summary of vGWA results for 60: FT Duration GH

Histogram of the phenotype & QQ plots of the Brown-Forsythe *p*-values (blue: nominal *p*-values, green: genomic controlled)

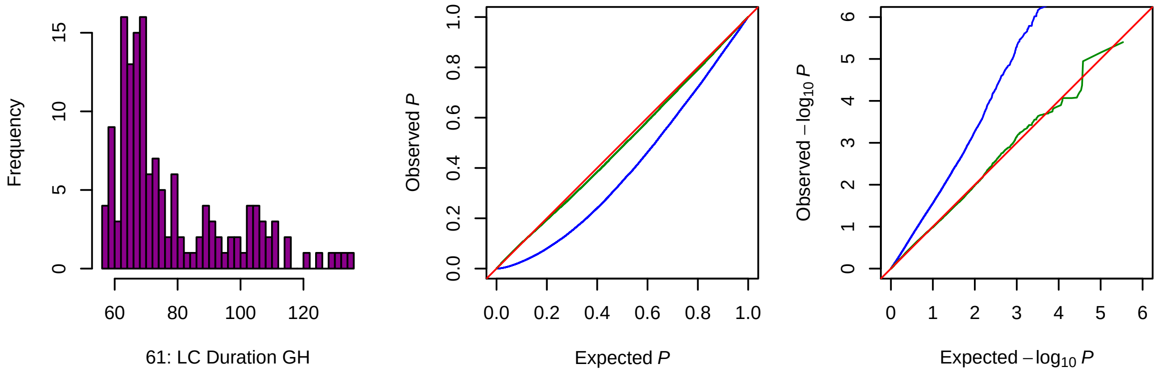

Brown-Forsythe (colored, behind) & Wilcoxon (yellow, in front) results, genomic controlled

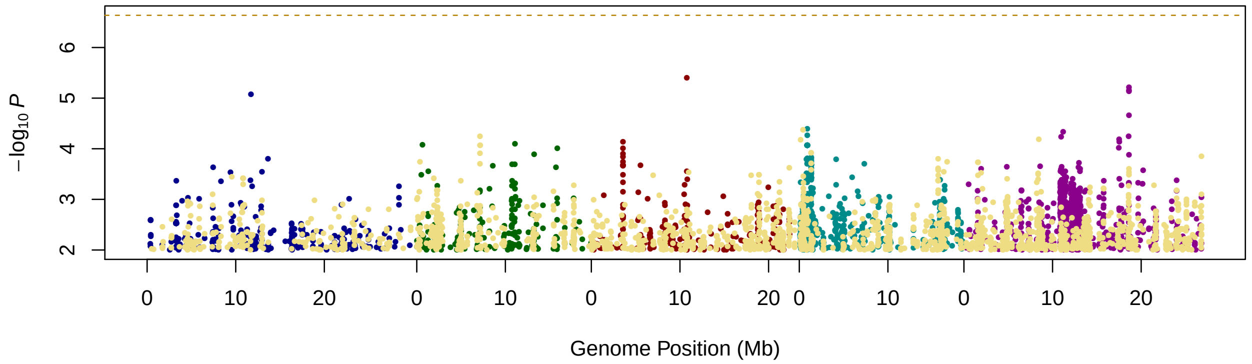

Comparison of Brown-Forsythe & Wilcoxon *p*-values (MAF: minor allele frequency)

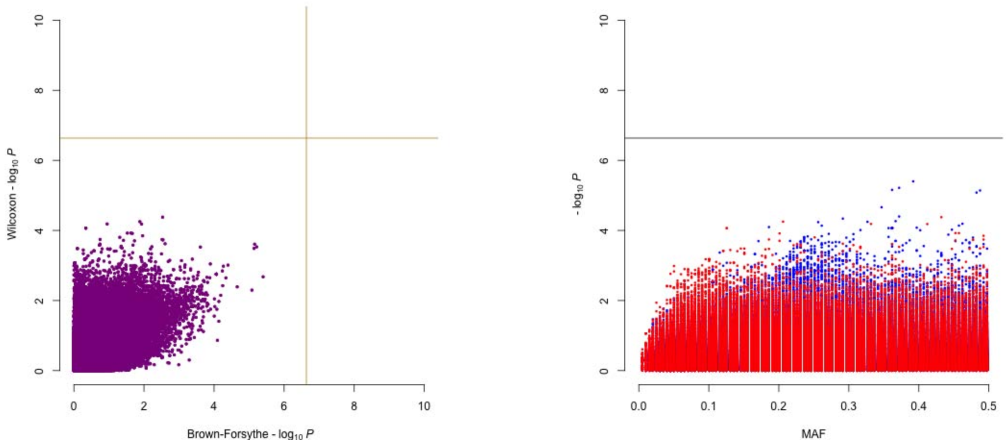

Supplementary Figure B41 - Summary of vGWA results for 61: LC Duration GH

Histogram of the phenotype & QQ plots of the Brown-Forsythe *p*-values (blue: nominal *p*-values, green: genomic controlled)

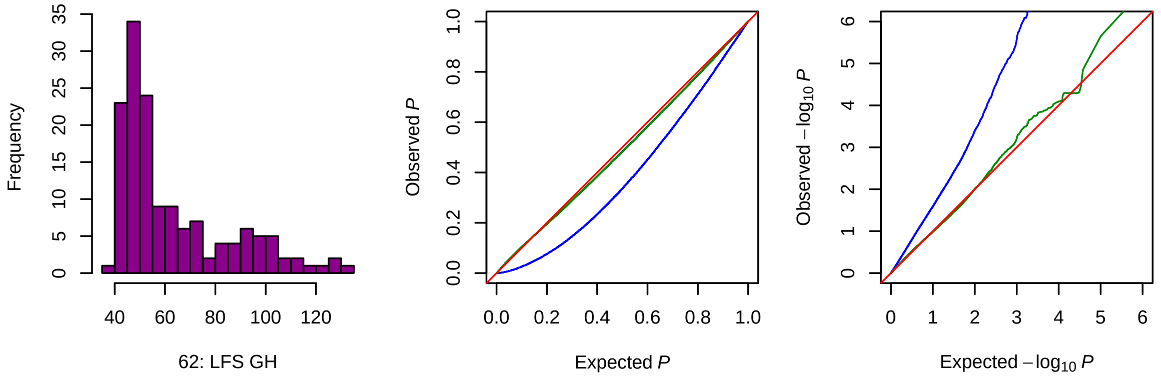

Brown-Forsythe (colored, behind) & Wilcoxon (yellow, in front) results, genomic controlled

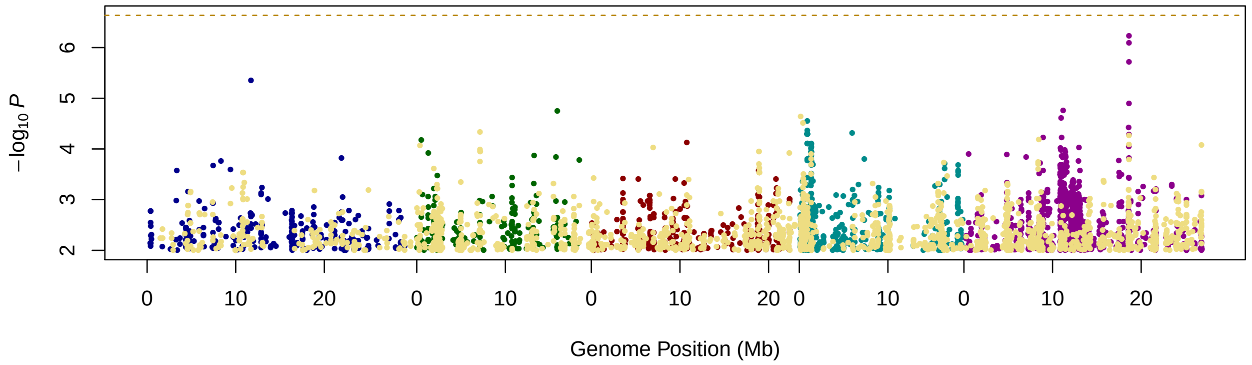

Comparison of Brown-Forsythe & Wilcoxon *p*-values (MAF: minor allele frequency)

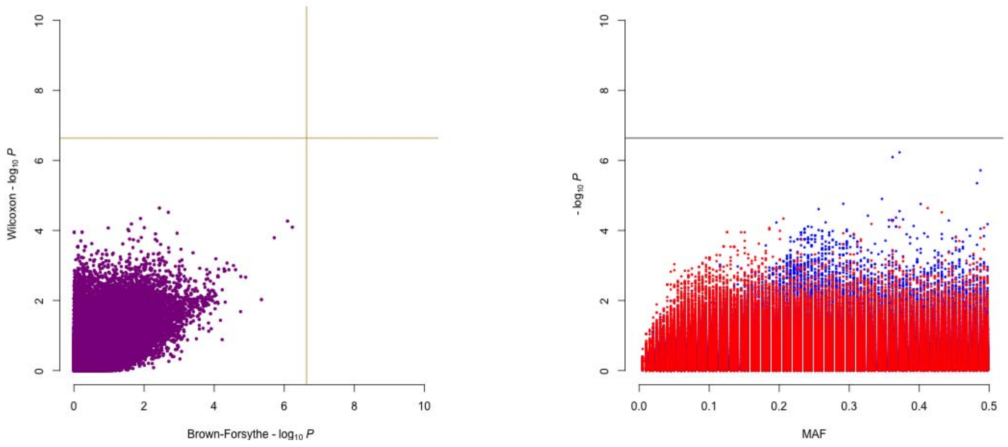

Supplementary Figure B42 - Summary of vGWA results for 62: LFS GH

Histogram of the phenotype & QQ plots of the Brown-Forsythe *p*-values (blue: nominal *p*-values, green: genomic controlled)

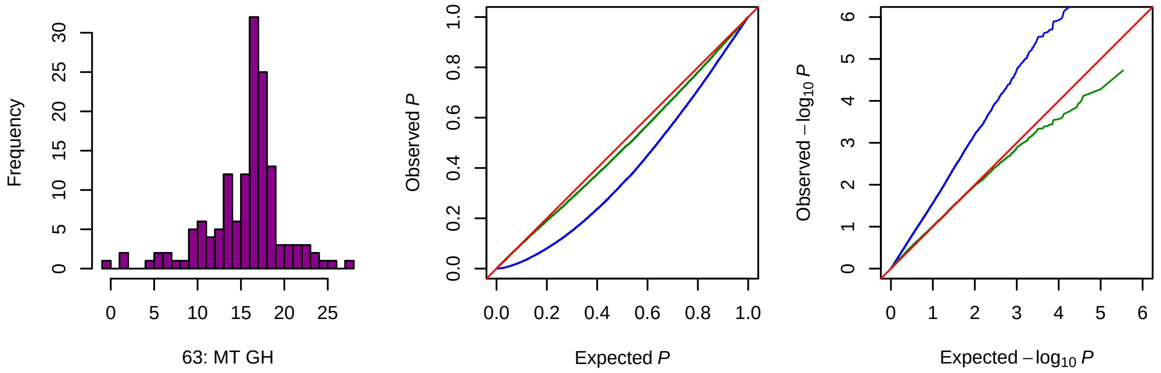

Brown-Forsythe (colored, behind) & Wilcoxon (yellow, in front) results, genomic controlled

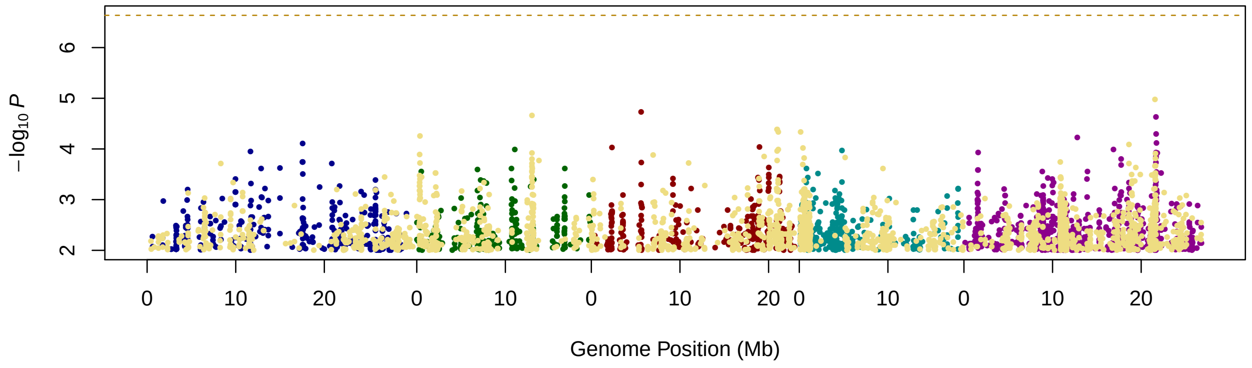

Comparison of Brown-Forsythe & Wilcoxon *p*-values (MAF: minor allele frequency)

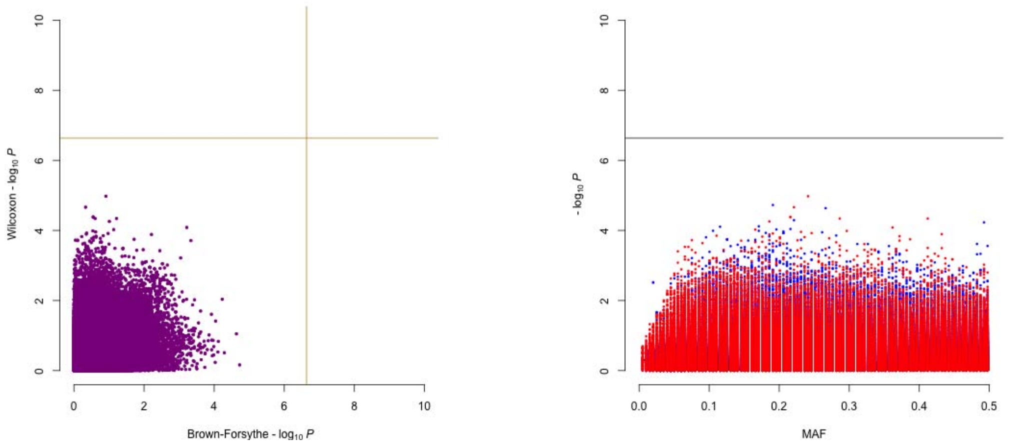

Supplementary Figure B43 - Summary of vGWA results for 63: MT GH

Histogram of the phenotype & QQ plots of the Brown-Forsythe *p*-values (blue: nominal *p*-values, green: genomic controlled)

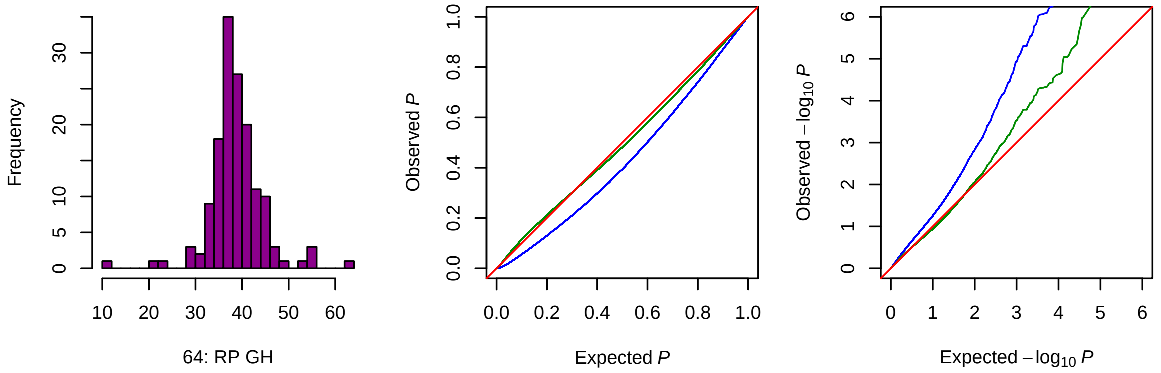

Brown-Forsythe (colored, behind) & Wilcoxon (yellow, in front) results, genomic controlled

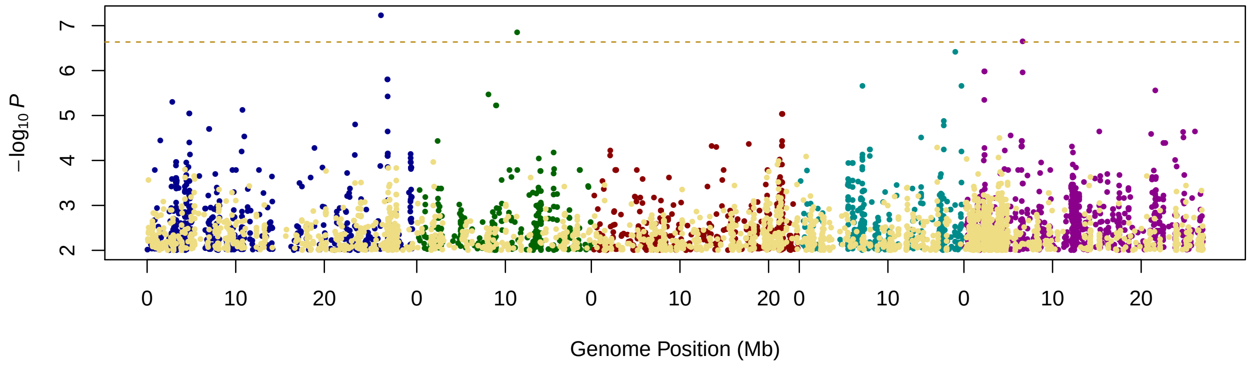

Comparison of Brown-Forsythe & Wilcoxon *p*-values (MAF: minor allele frequency)

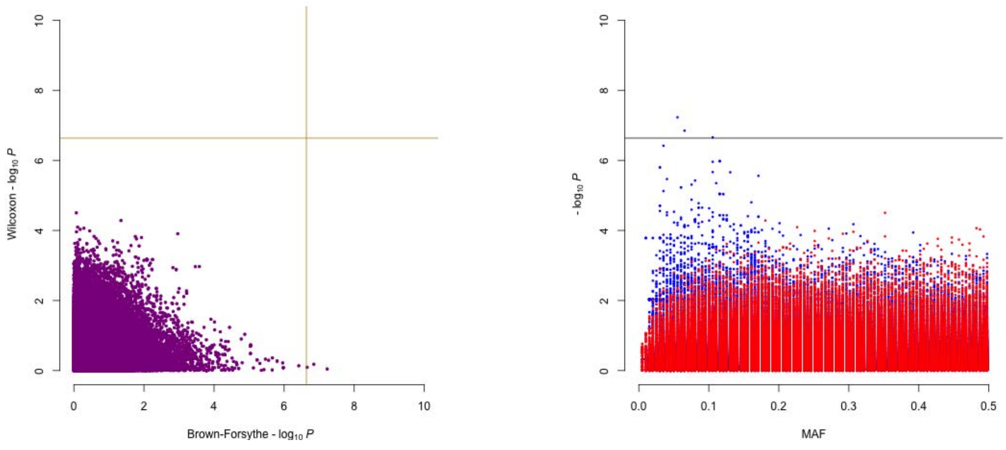

Supplementary Figure B44 - Summary of vGWA results for 64: RP GH

Histogram of the phenotype & QQ plots of the Brown-Forsythe *p*-values (blue: nominal *p*-values, green: genomic controlled)

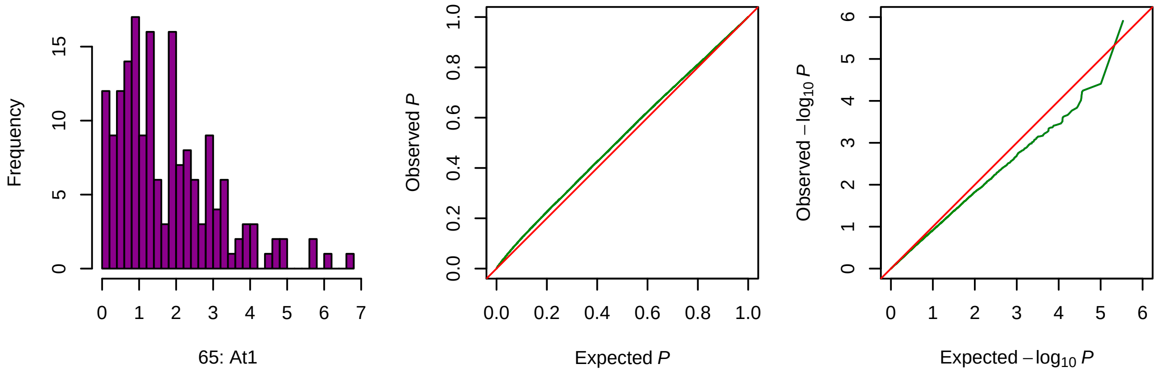

Brown-Forsythe (colored, behind) & Wilcoxon (yellow, in front) results, genomic controlled

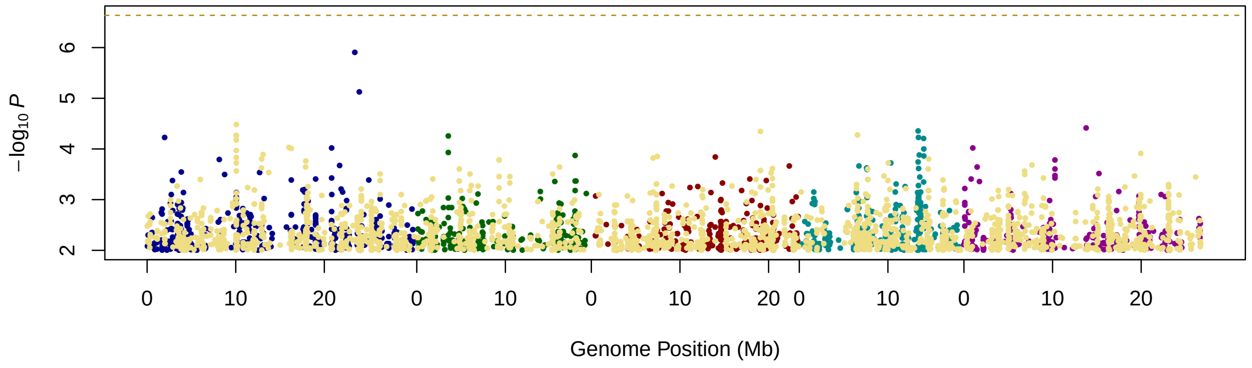

Comparison of Brown-Forsythe & Wilcoxon *p*-values (MAF: minor allele frequency)

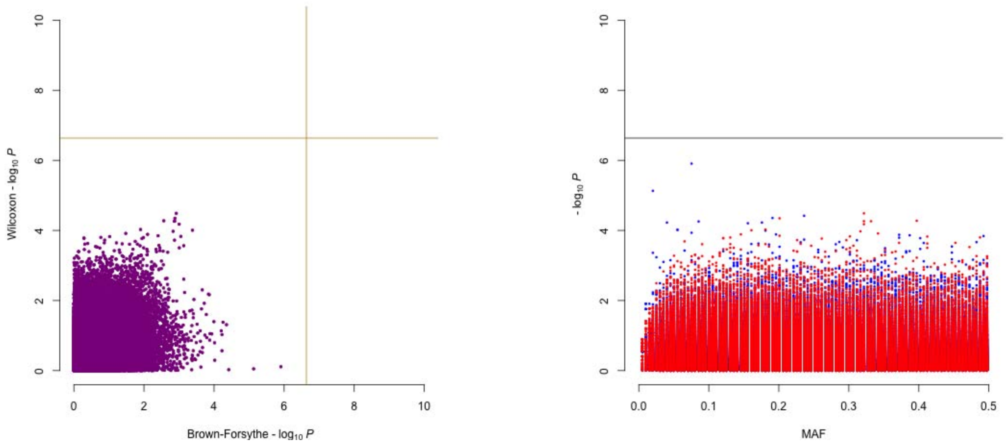

Supplementary Figure B45 - Summary of vGWA results for 65: At1

Histogram of the phenotype & QQ plots of the Brown-Forsythe *p*-values (blue: nominal *p*-values, green: genomic controlled)

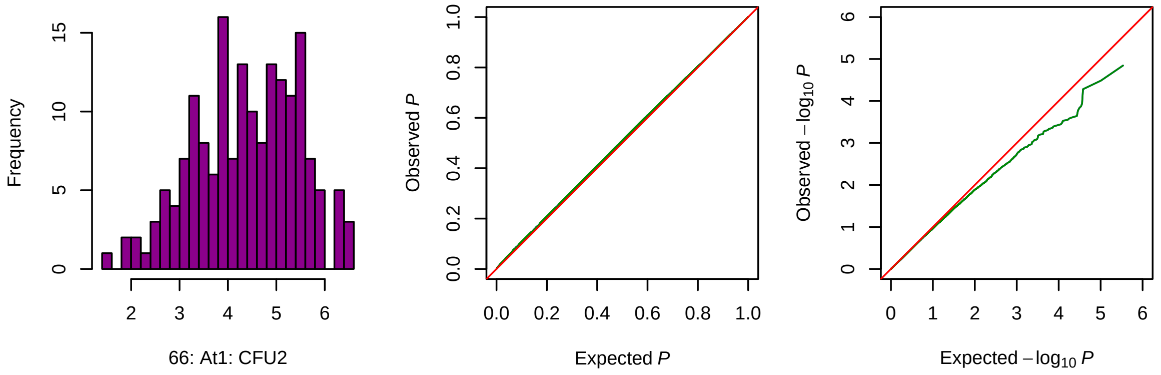

Brown-Forsythe (colored, behind) & Wilcoxon (yellow, in front) results, genomic controlled

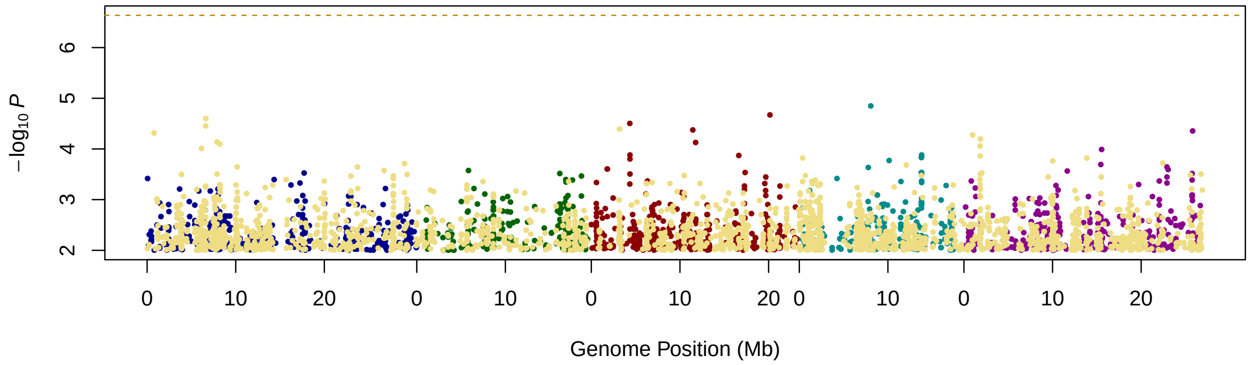

Comparison of Brown-Forsythe & Wilcoxon *p*-values (MAF: minor allele frequency)

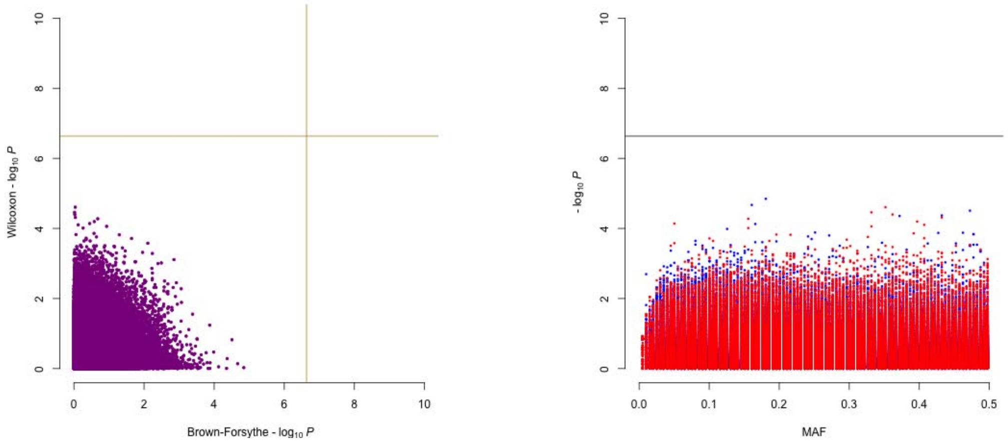

Supplementary Figure B46 - Summary of vGWA results for 66: At1: CFU2

Histogram of the phenotype & QQ plots of the Brown-Forsythe *p*-values (blue: nominal *p*-values, green: genomic controlled)

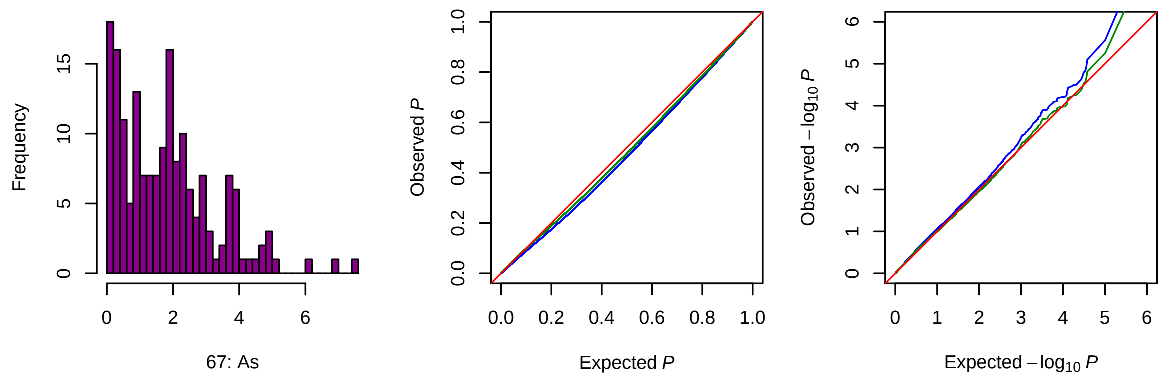

Brown-Forsythe (colored, behind) & Wilcoxon (yellow, in front) results, genomic controlled

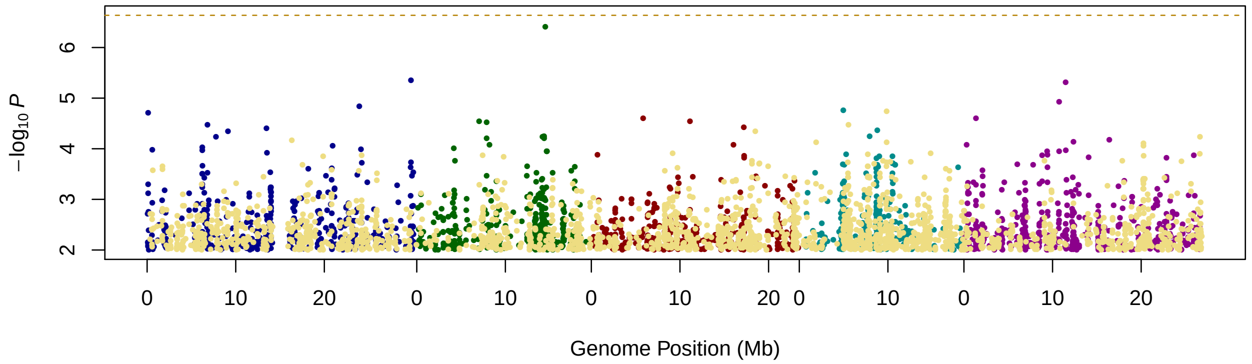

Comparison of Brown-Forsythe & Wilcoxon *p*-values (MAF: minor allele frequency)

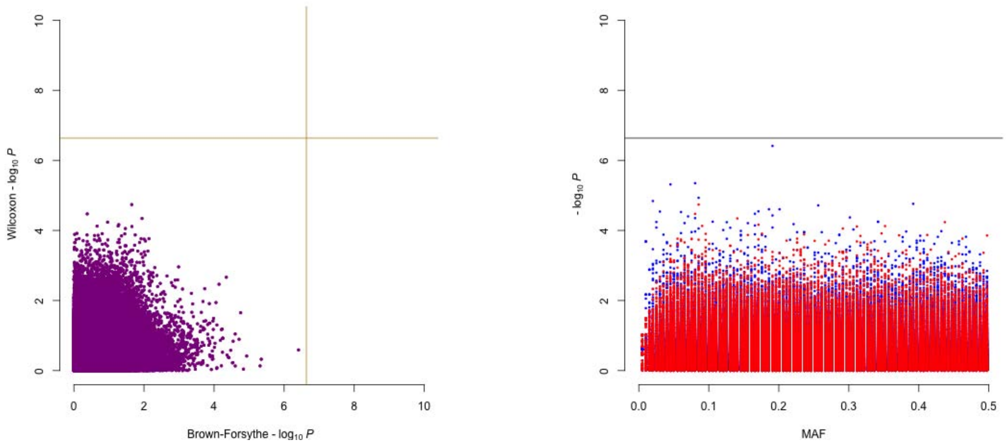

Supplementary Figure B47 - Summary of vGWA results for 67: As

Histogram of the phenotype & QQ plots of the Brown-Forsythe *p*-values (blue: nominal *p*-values, green: genomic controlled)

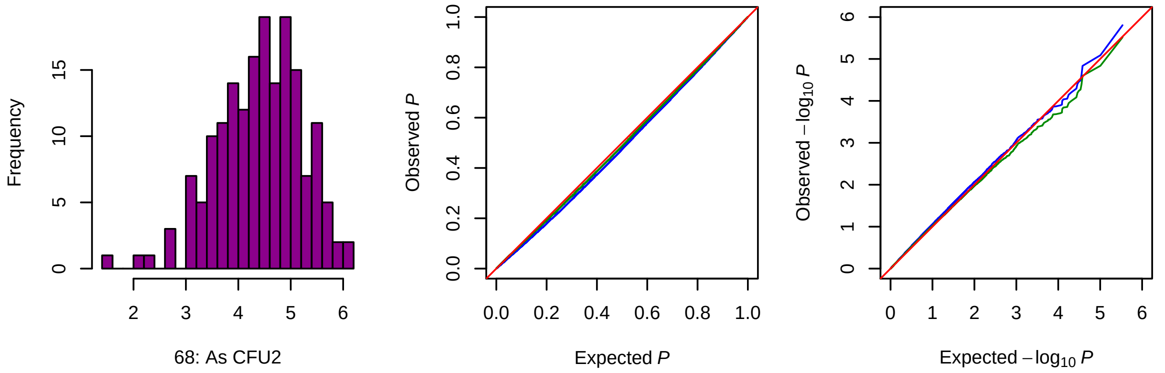

Brown-Forsythe (colored, behind) & Wilcoxon (yellow, in front) results, genomic controlled

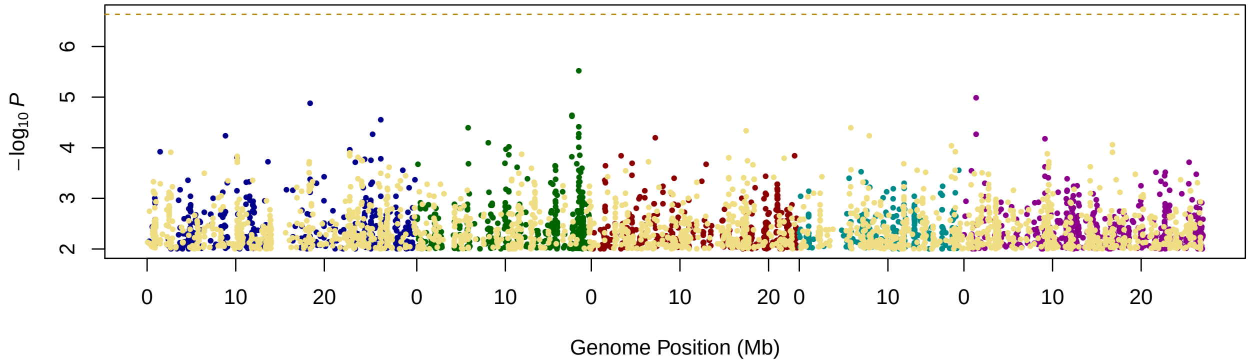

Comparison of Brown-Forsythe & Wilcoxon *p*-values (MAF: minor allele frequency)

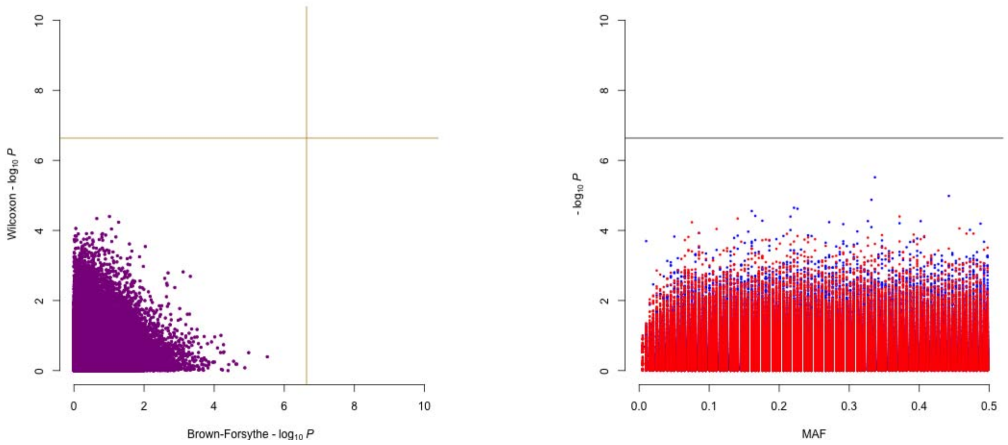

Supplementary Figure B48 - Summary of vGWA results for 68: As CFU2

Histogram of the phenotype & QQ plots of the Brown-Forsythe *p*-values (blue: nominal *p*-values, green: genomic controlled)

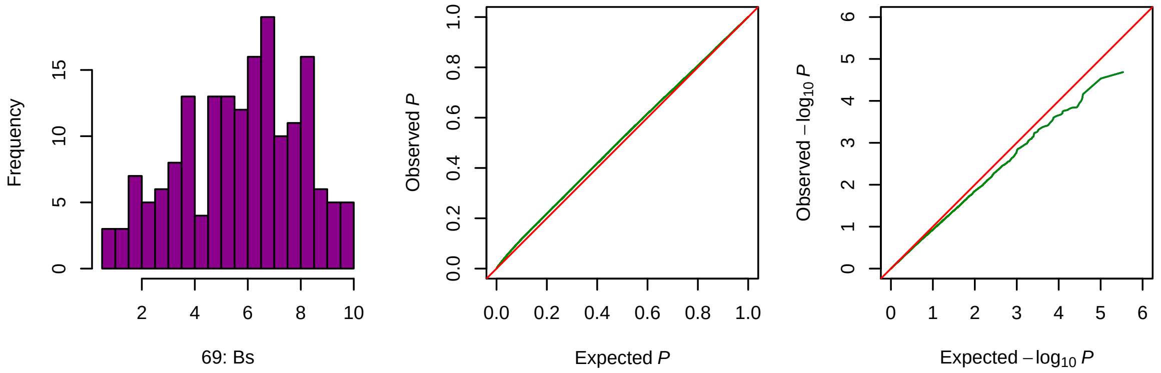

Brown-Forsythe (colored, behind) & Wilcoxon (yellow, in front) results, genomic controlled

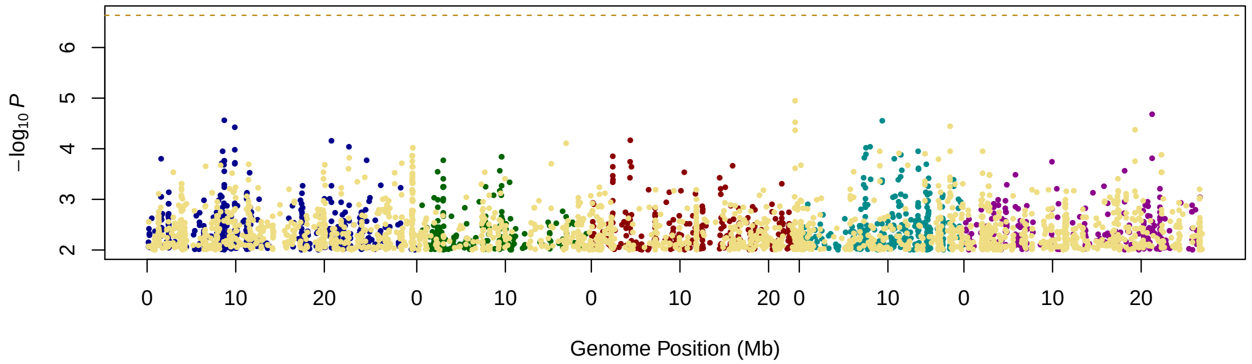

Comparison of Brown-Forsythe & Wilcoxon *p*-values (MAF: minor allele frequency)

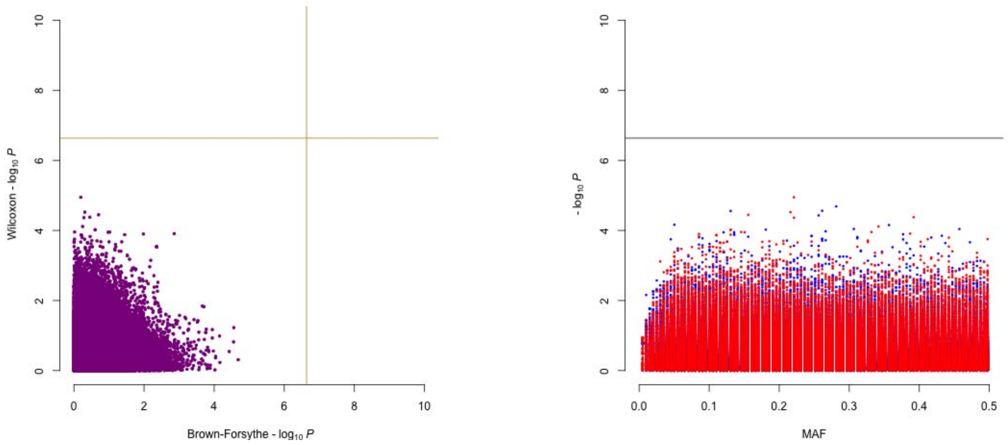

Supplementary Figure B49 - Summary of vGWA results for 69: Bs

Histogram of the phenotype & QQ plots of the Brown-Forsythe *p*-values (blue: nominal *p*-values, green: genomic controlled)

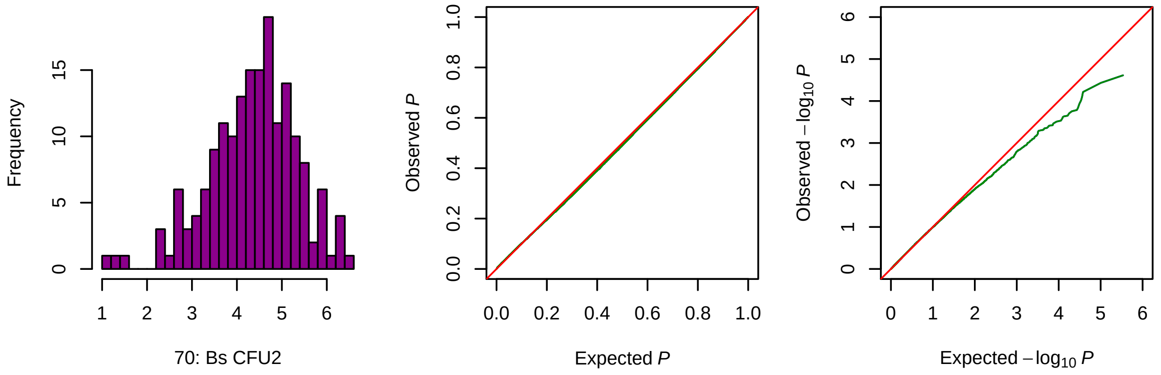

Brown-Forsythe (colored, behind) & Wilcoxon (yellow, in front) results, genomic controlled

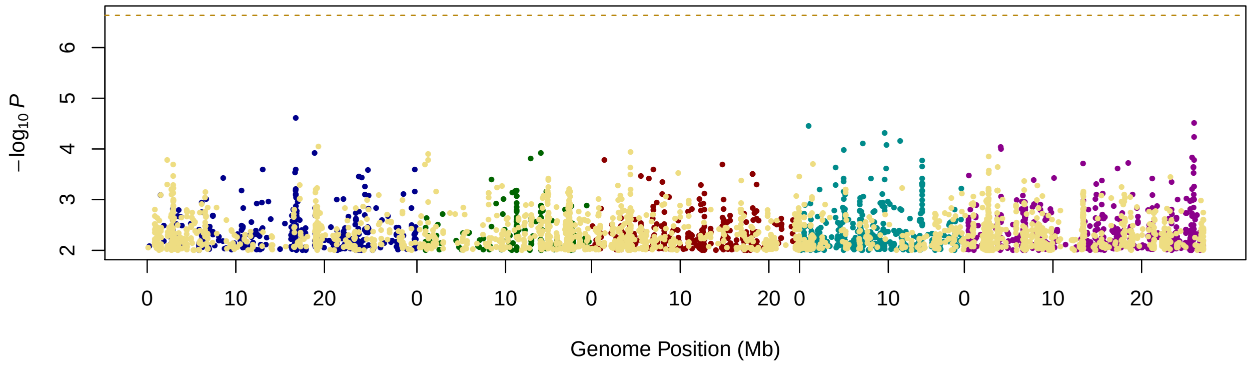

Comparison of Brown-Forsythe & Wilcoxon *p*-values (MAF: minor allele frequency)

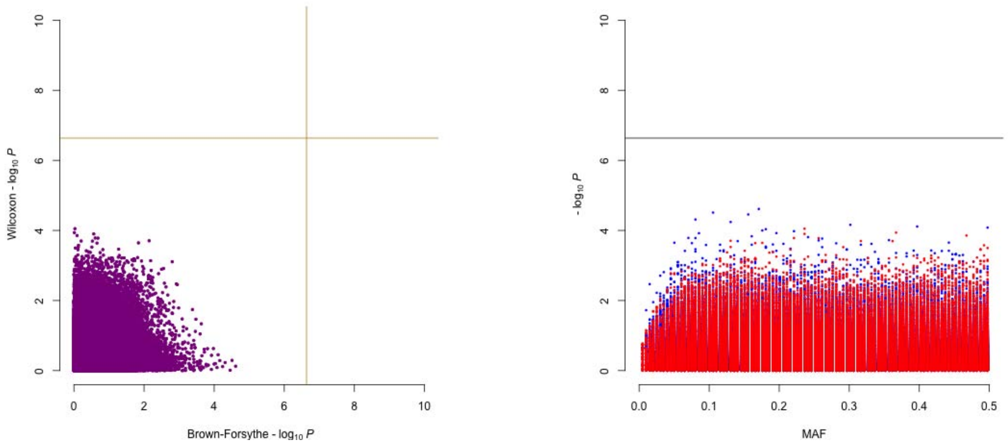

Supplementary Figure B50 - Summary of vGWA results for 70: Bs CFU2

Histogram of the phenotype & QQ plots of the Brown-Forsythe *p*-values (blue: nominal *p*-values, green: genomic controlled)

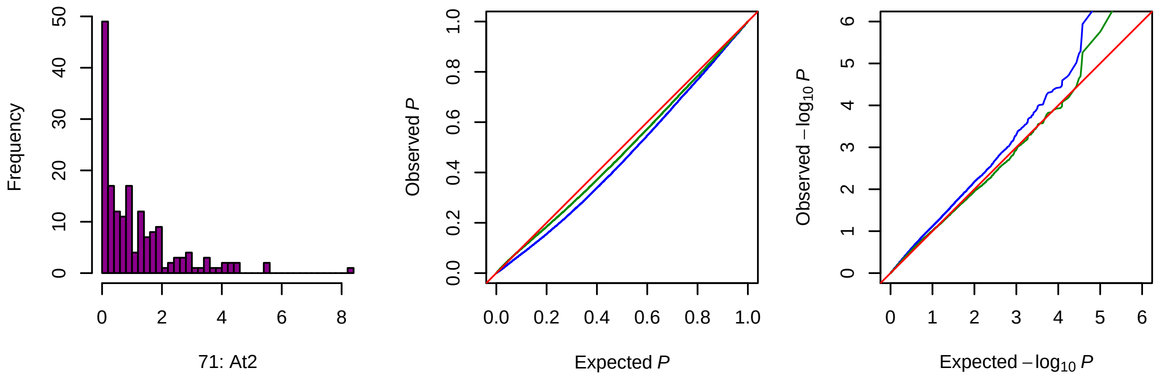

Brown-Forsythe (colored, behind) & Wilcoxon (yellow, in front) results, genomic controlled

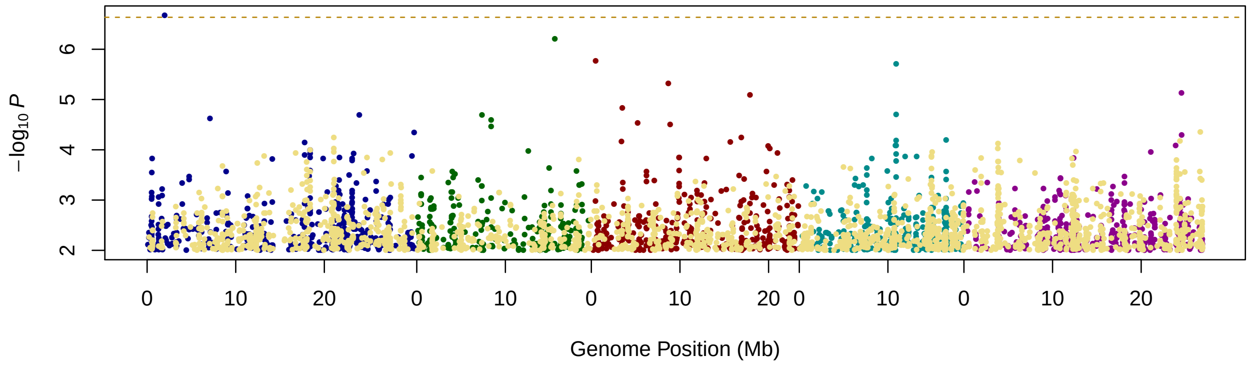

Comparison of Brown-Forsythe & Wilcoxon *p*-values (MAF: minor allele frequency)

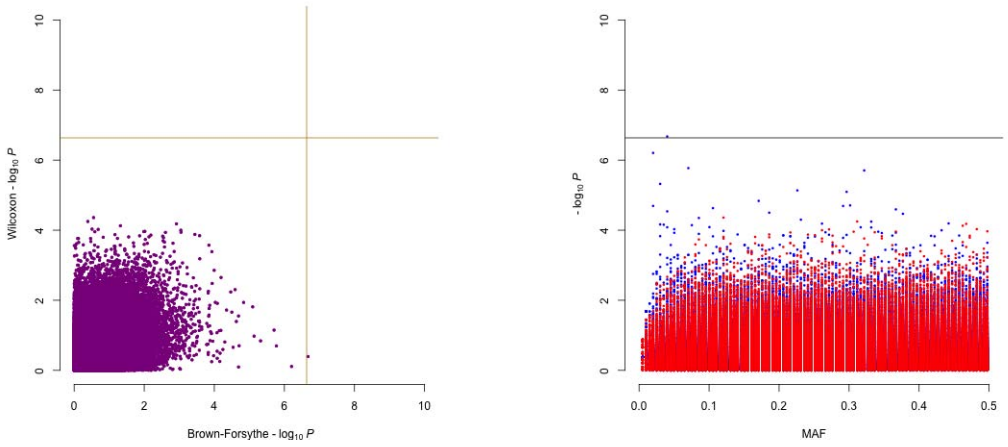

Supplementary Figure B51 - Summary of vGWA results for 71: At2

Histogram of the phenotype & QQ plots of the Brown-Forsythe *p*-values (blue: nominal *p*-values, green: genomic controlled)

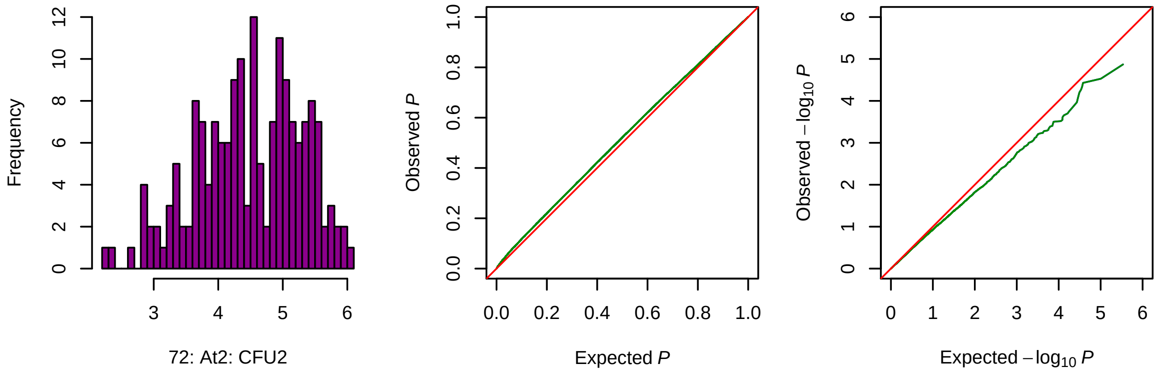

Brown-Forsythe (colored, behind) & Wilcoxon (yellow, in front) results, genomic controlled

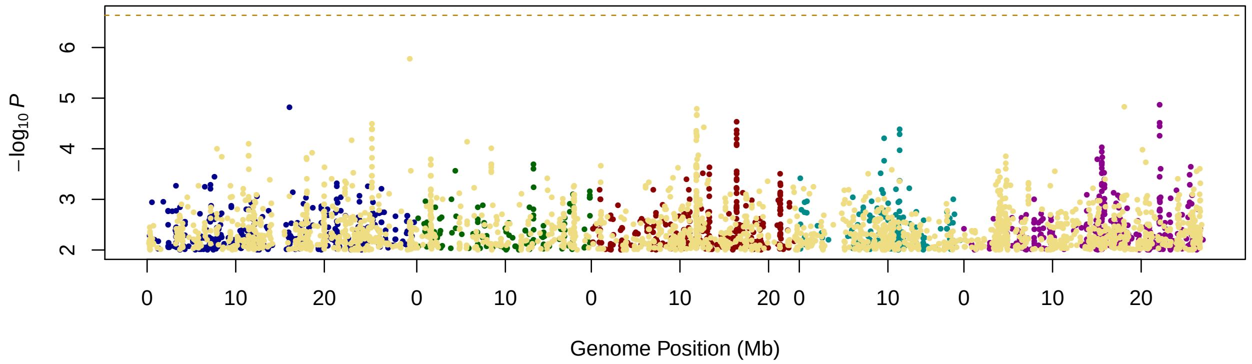

Comparison of Brown-Forsythe & Wilcoxon *p*-values (MAF: minor allele frequency)

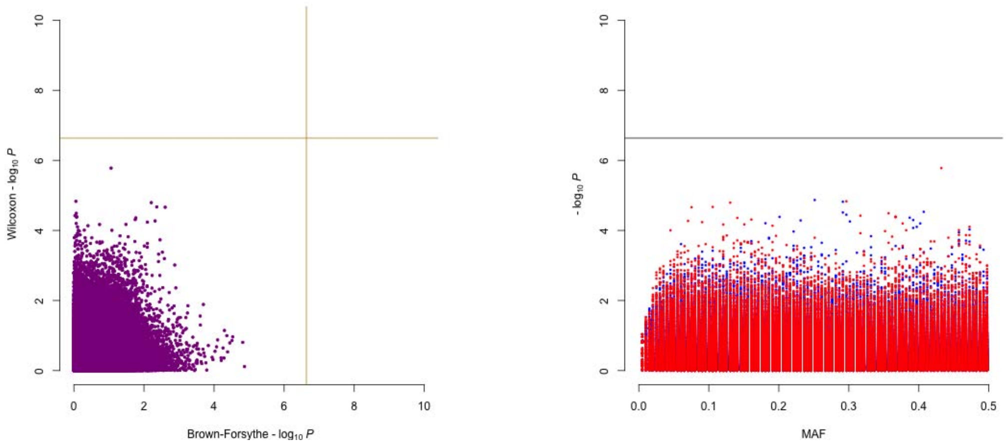

Supplementary Figure B52 - Summary of vGWA results for 72: At2: CFU2

Histogram of the phenotype & QQ plots of the Brown-Forsythe *p*-values (blue: nominal *p*-values, green: genomic controlled)

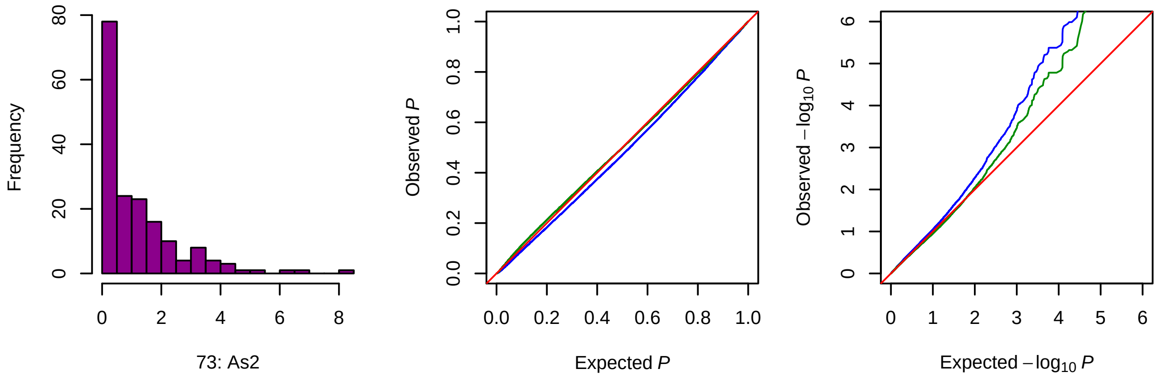

Brown-Forsythe (colored, behind) & Wilcoxon (yellow, in front) results, genomic controlled

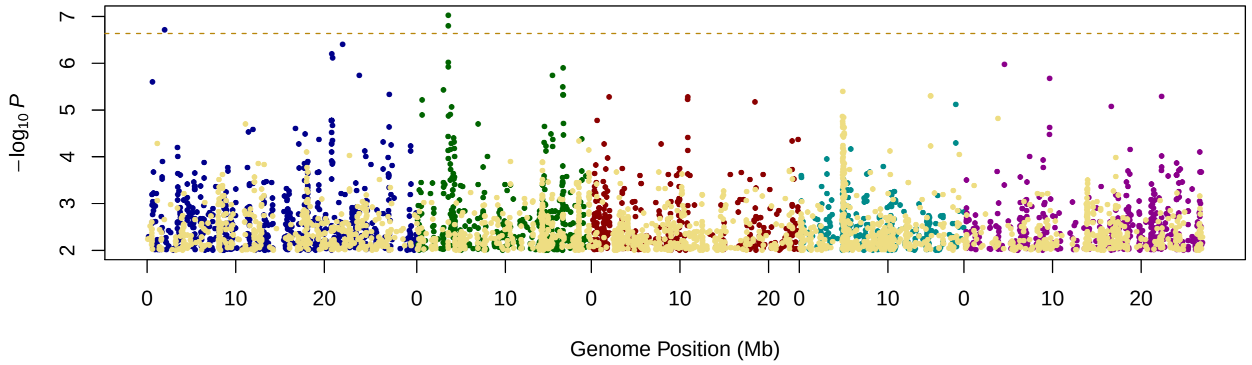

Comparison of Brown-Forsythe & Wilcoxon *p*-values (MAF: minor allele frequency)

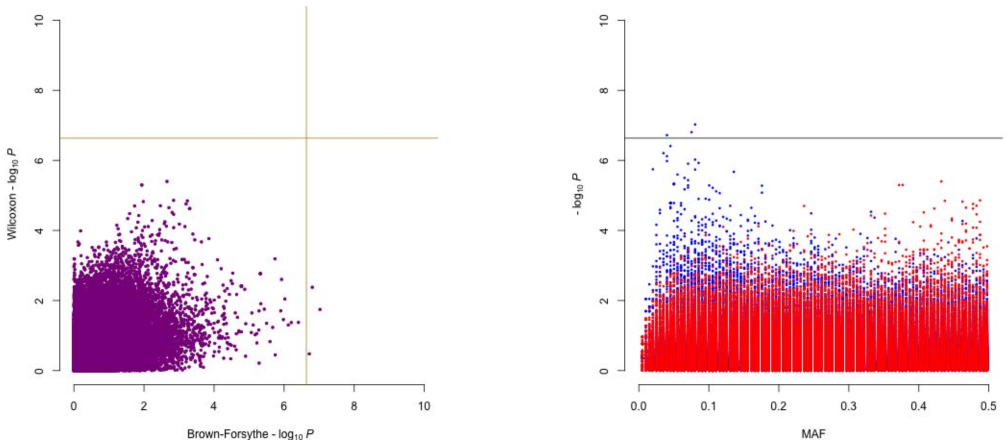

Supplementary Figure B53 - Summary of vGWA results for 73: As2

Histogram of the phenotype & QQ plots of the Brown-Forsythe *p*-values (blue: nominal *p*-values, green: genomic controlled)

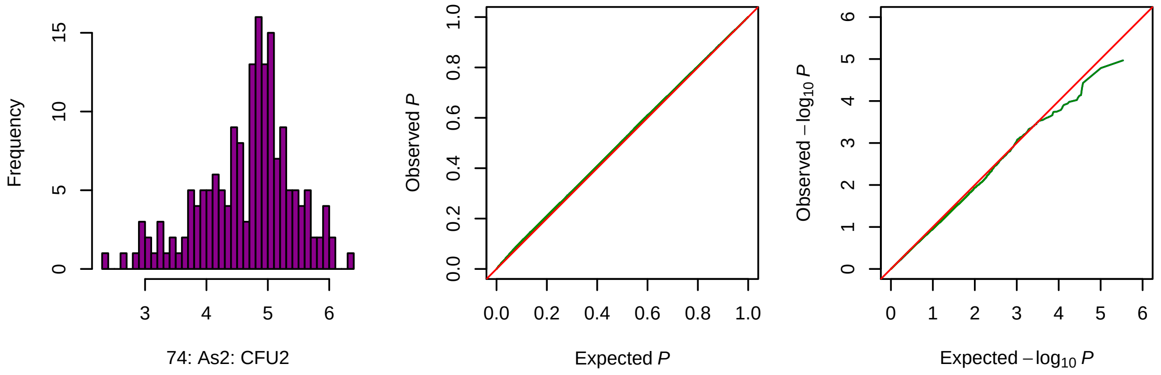

Brown-Forsythe (colored, behind) & Wilcoxon (yellow, in front) results, genomic controlled

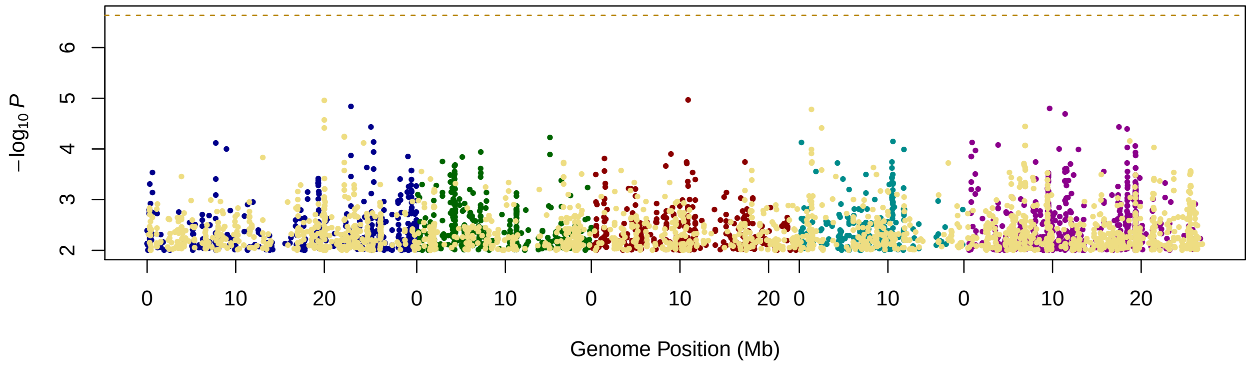

Comparison of Brown-Forsythe & Wilcoxon *p*-values (MAF: minor allele frequency)

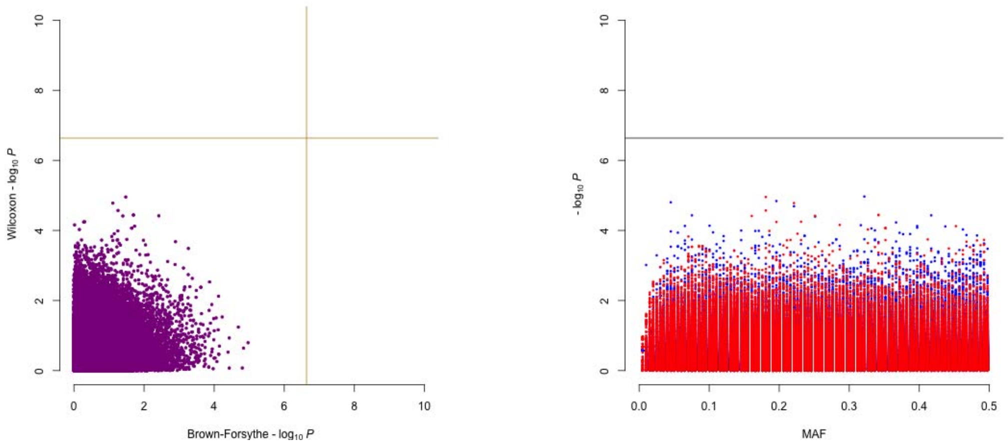

Supplementary Figure B54 - Summary of vGWA results for 74: As2: CFU2

Histogram of the phenotype & QQ plots of the Brown-Forsythe *p*-values (blue: nominal *p*-values, green: genomic controlled)

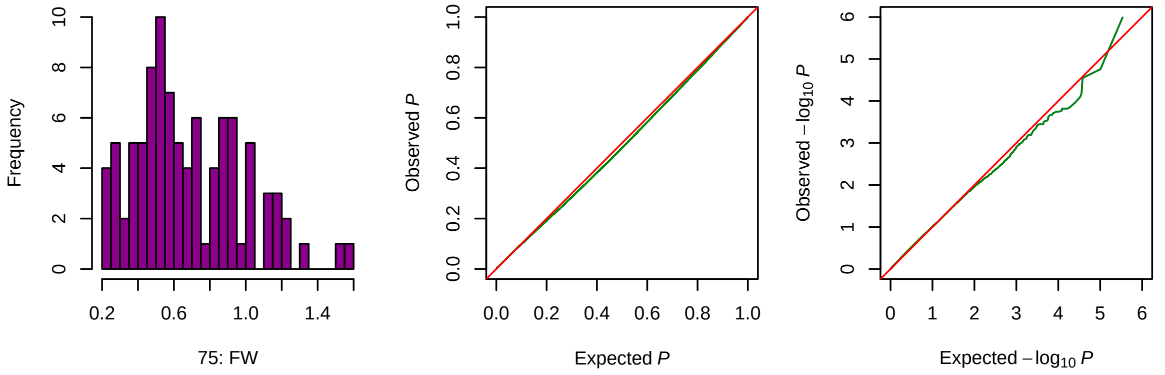

Brown-Forsythe (colored, behind) & Wilcoxon (yellow, in front) results, genomic controlled

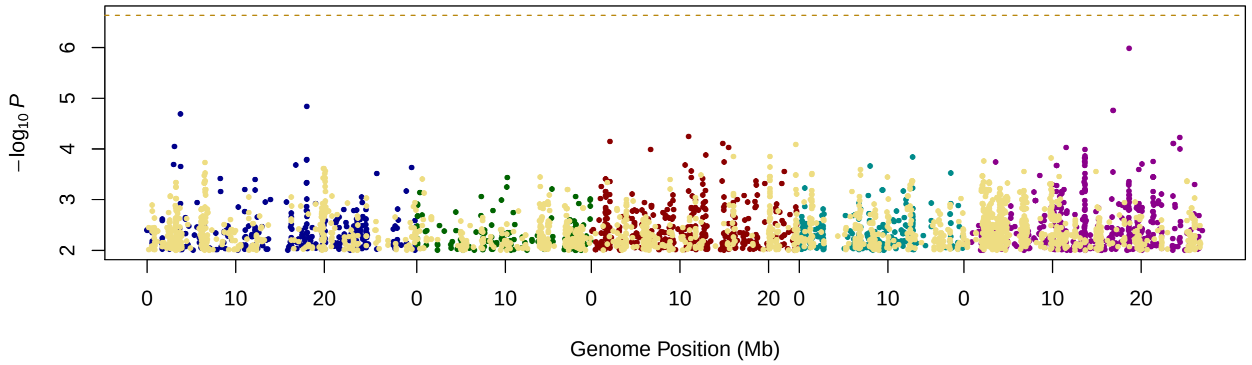

Comparison of Brown-Forsythe & Wilcoxon *p*-values (MAF: minor allele frequency)

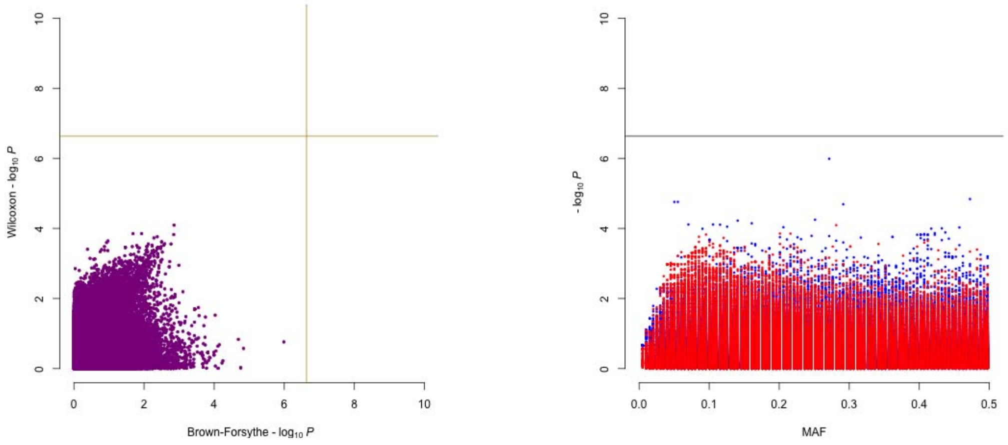

Supplementary Figure B55 - Summary of vGWA results for 75: FW

Histogram of the phenotype & QQ plots of the Brown-Forsythe *p*-values (blue: nominal *p*-values, green: genomic controlled)

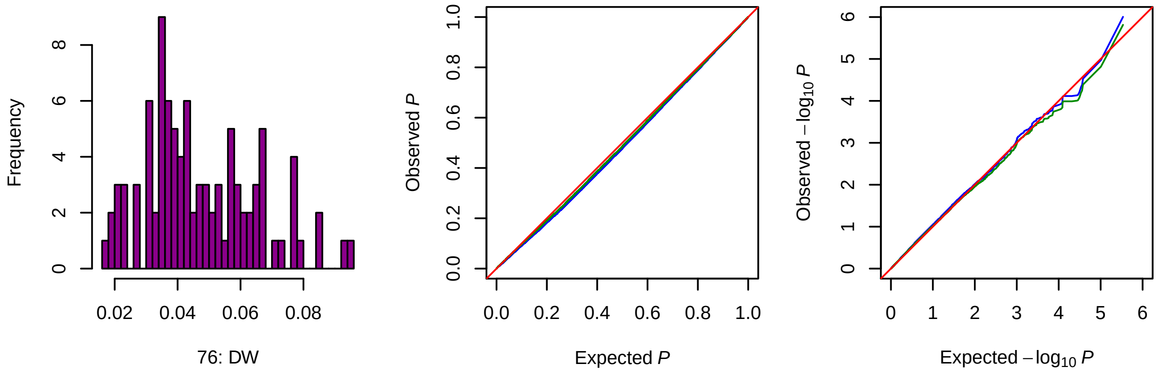

Brown-Forsythe (colored, behind) & Wilcoxon (yellow, in front) results, genomic controlled

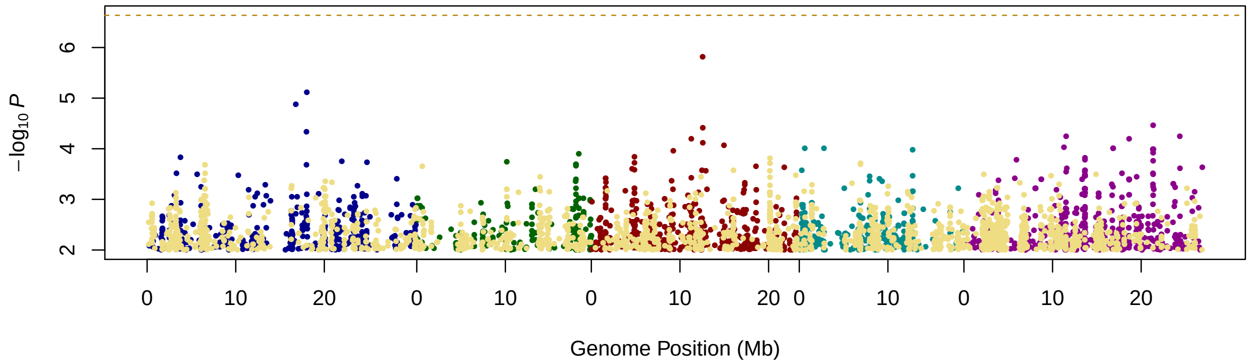

Comparison of Brown-Forsythe & Wilcoxon *p*-values (MAF: minor allele frequency)

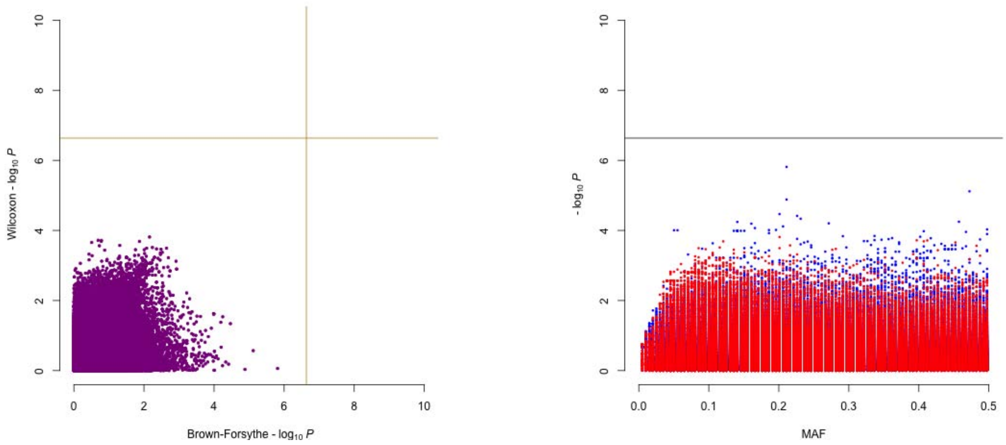

Supplementary Figure B56 - Summary of vGWA results for 76: DW

Histogram of the phenotype & QQ plots of the Brown-Forsythe *p*-values (blue: nominal *p*-values, green: genomic controlled)

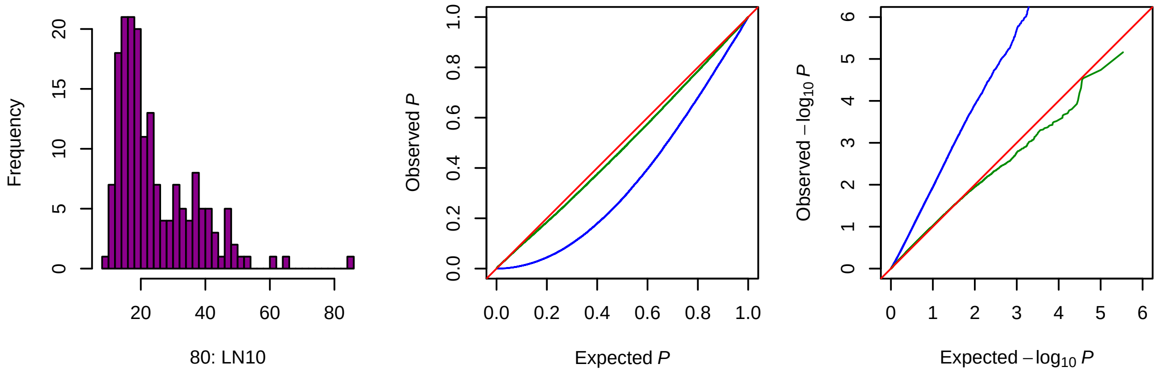

Brown-Forsythe (colored, behind) & Wilcoxon (yellow, in front) results, genomic controlled

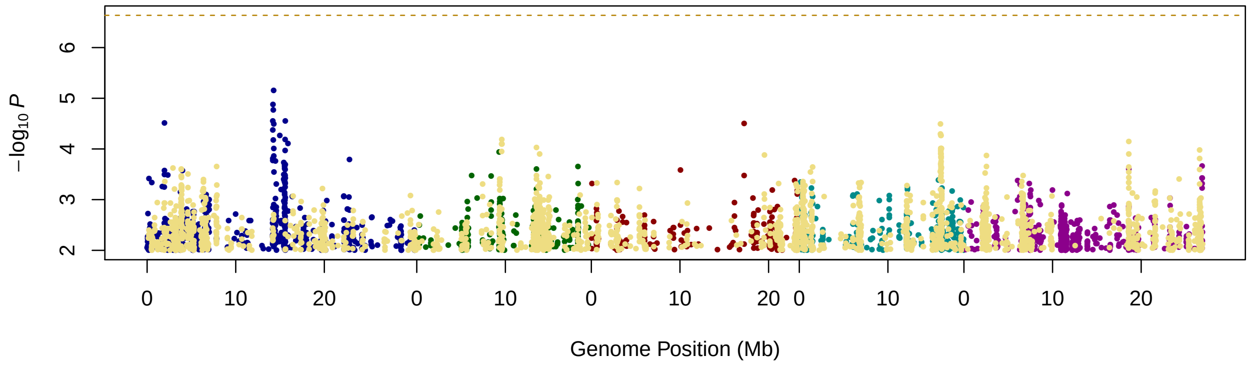

Comparison of Brown-Forsythe & Wilcoxon *p*-values (MAF: minor allele frequency)

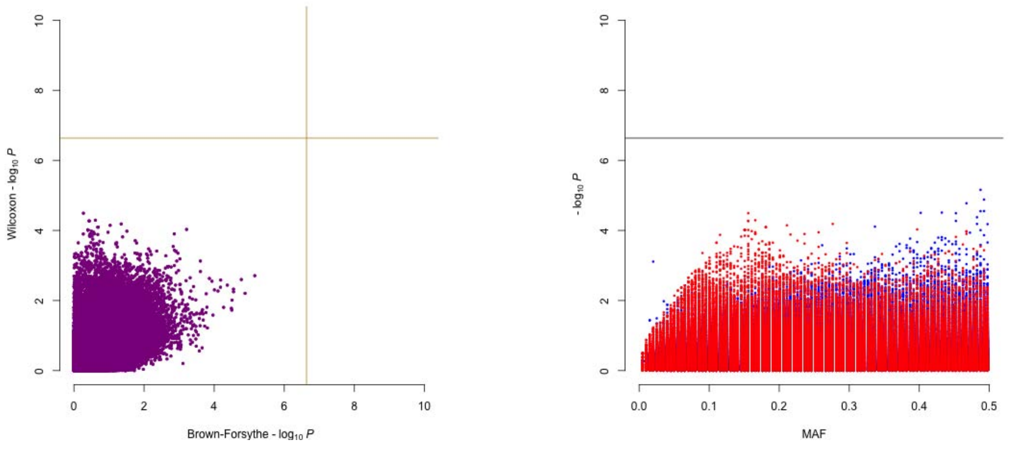

Supplementary Figure B57 - Summary of vGWA results for 80: LN10

Histogram of the phenotype & QQ plots of the Brown-Forsythe *p*-values (blue: nominal *p*-values, green: genomic controlled)

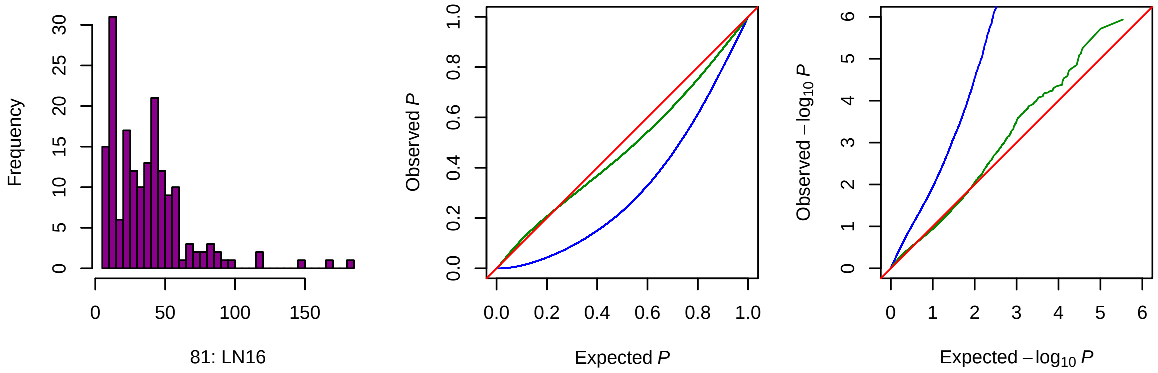

Brown-Forsythe (colored, behind) & Wilcoxon (yellow, in front) results, genomic controlled

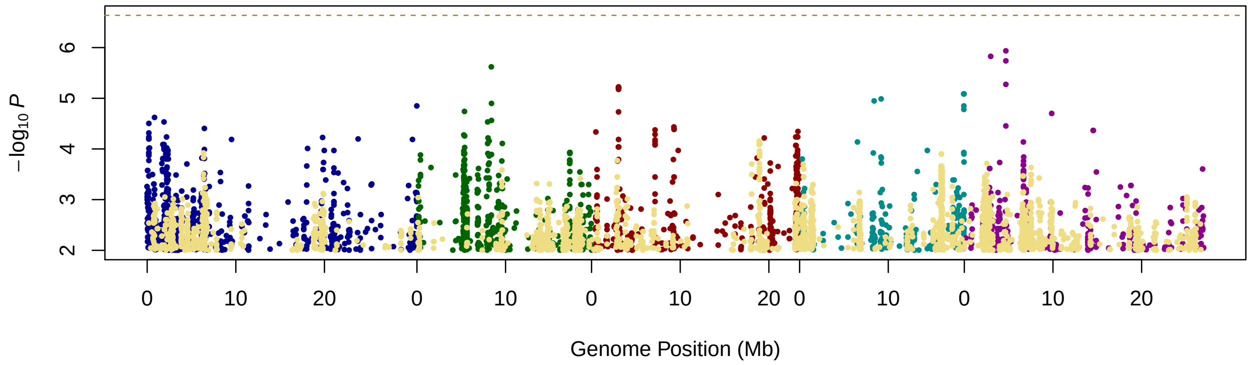

Comparison of Brown-Forsythe & Wilcoxon *p*-values (MAF: minor allele frequency)

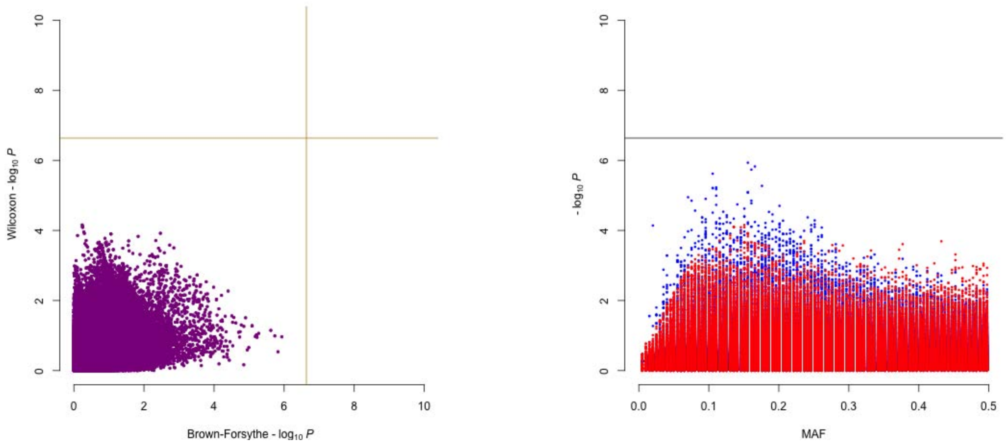

Supplementary Figure B58 - Summary of vGWA results for 81: LN16

Histogram of the phenotype & QQ plots of the Brown-Forsythe *p*-values (blue: nominal *p*-values, green: genomic controlled)

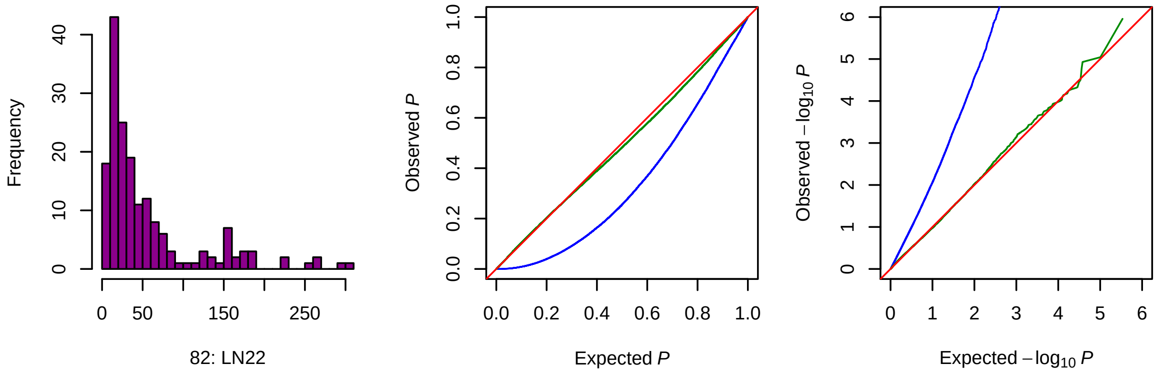

Brown-Forsythe (colored, behind) & Wilcoxon (yellow, in front) results, genomic controlled

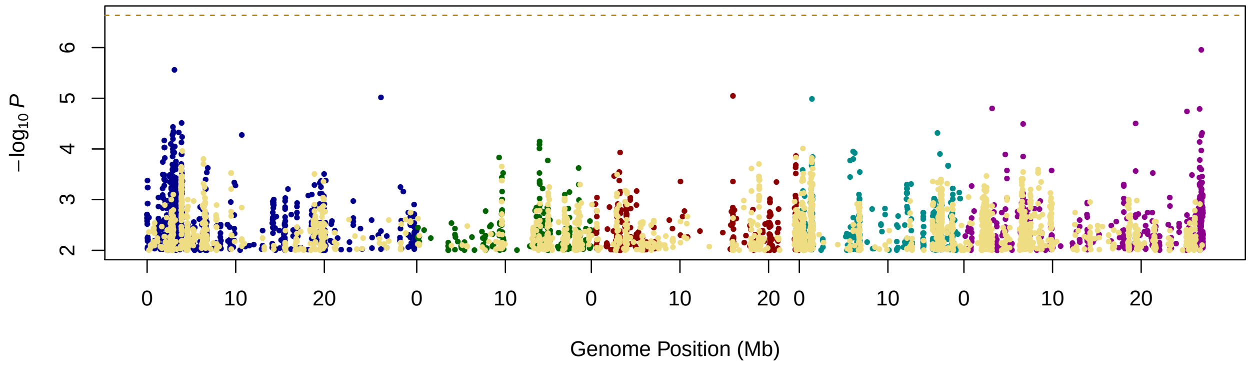

Comparison of Brown-Forsythe & Wilcoxon *p*-values (MAF: minor allele frequency)

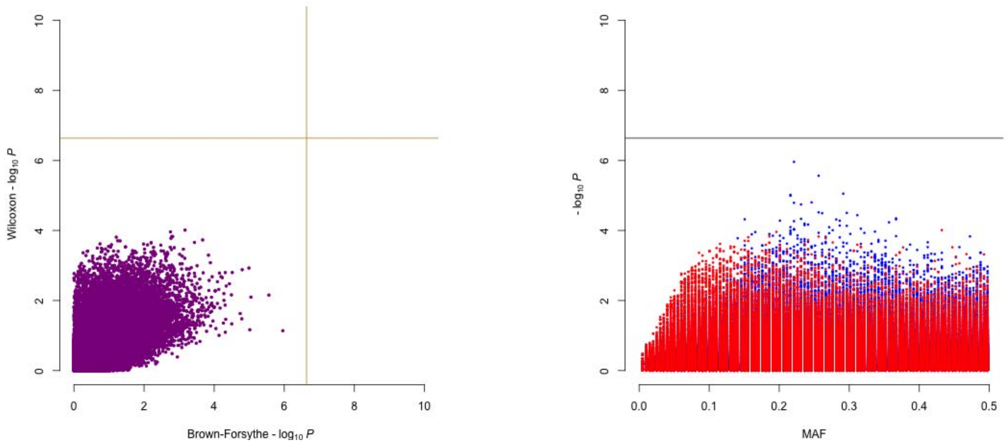

Supplementary Figure B59 - Summary of vGWA results for 82: LN22

Histogram of the phenotype & QQ plots of the Brown-Forsythe *p*-values (blue: nominal *p*-values, green: genomic controlled)

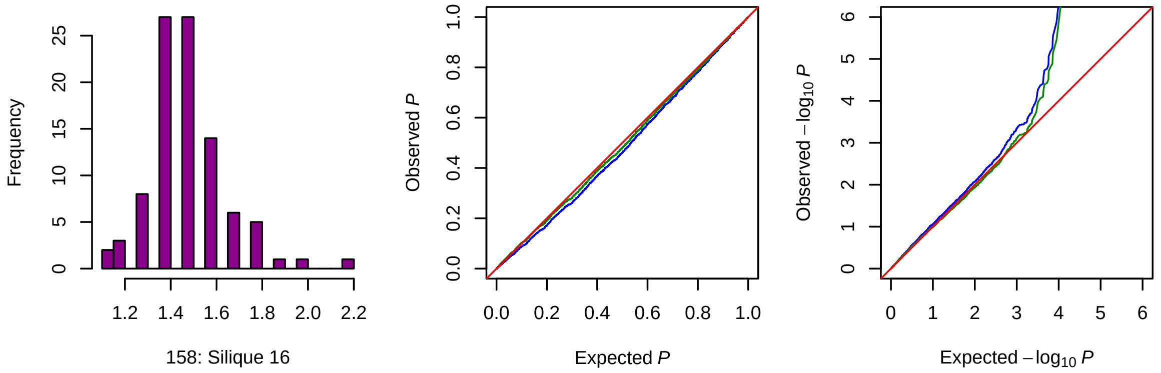

Brown-Forsythe (colored, behind) & Wilcoxon (yellow, in front) results, genomic controlled

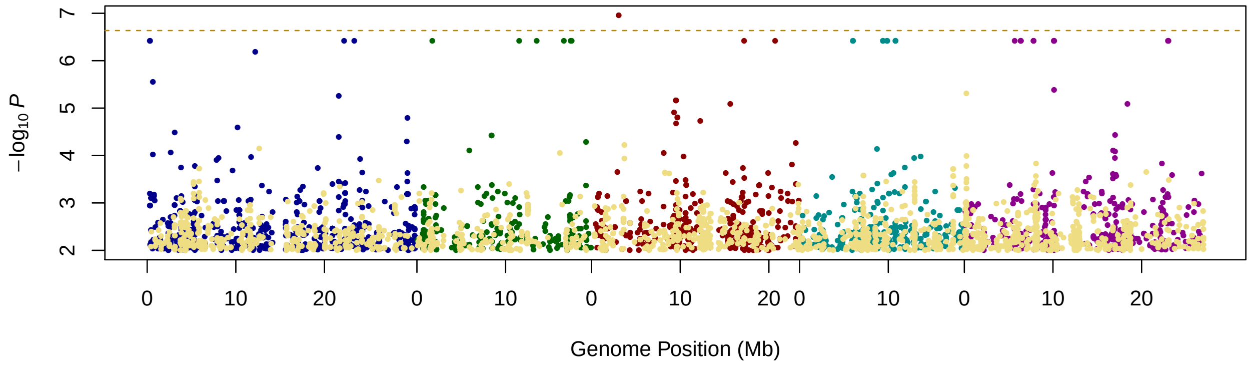

Comparison of Brown-Forsythe & Wilcoxon *p*-values (MAF: minor allele frequency)

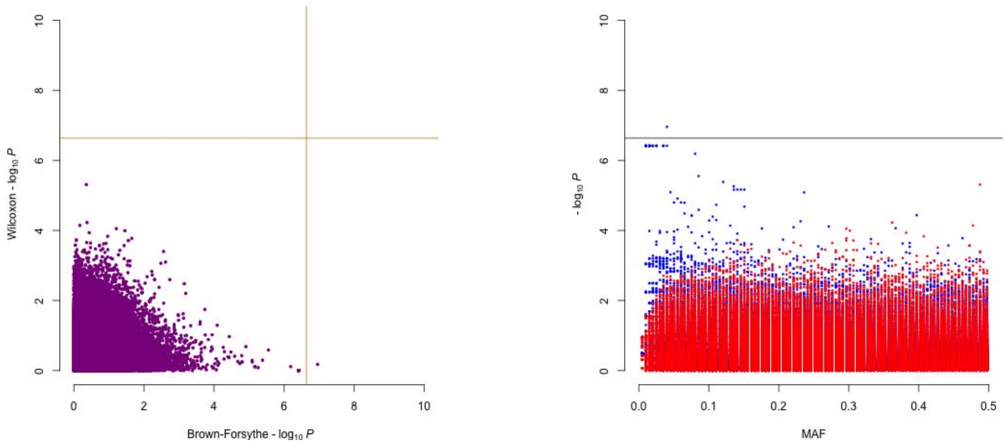

Supplementary Figure B60 - Summary of vGWA results for 158: Silique 16

Histogram of the phenotype & QQ plots of the Brown-Forsythe *p*-values (blue: nominal *p*-values, green: genomic controlled)

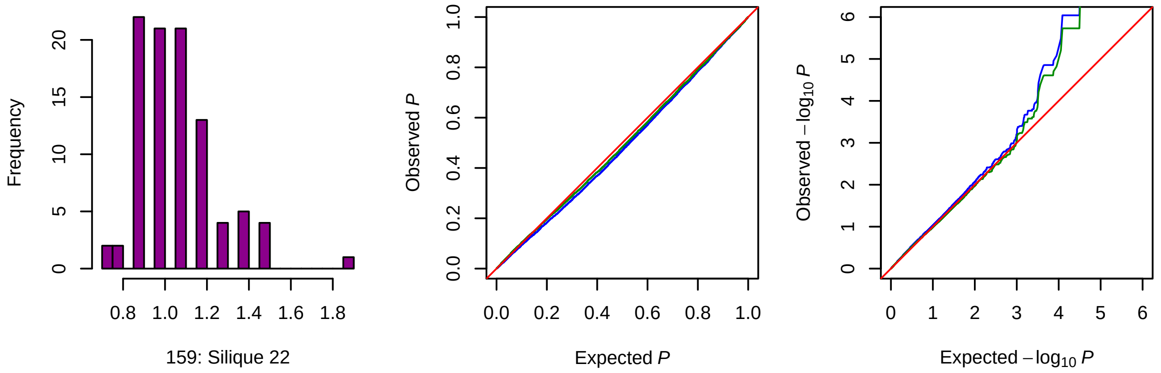

Brown-Forsythe (colored, behind) & Wilcoxon (yellow, in front) results, genomic controlled

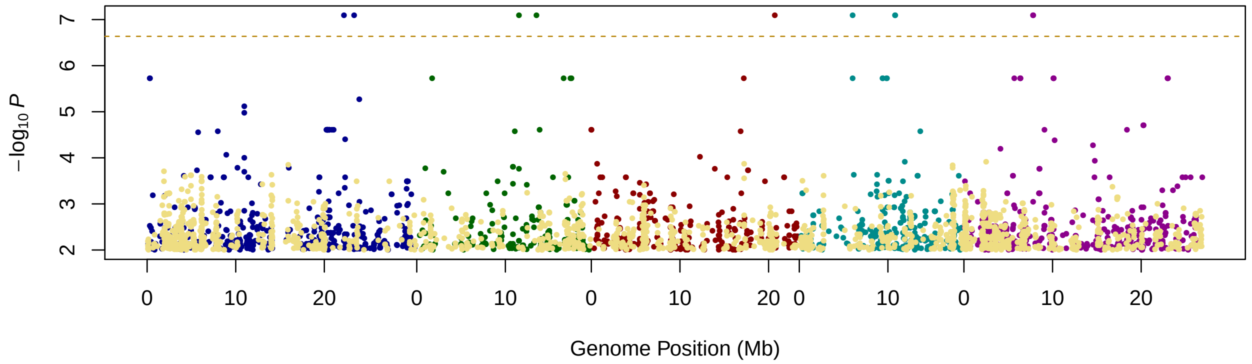

Comparison of Brown-Forsythe & Wilcoxon *p*-values (MAF: minor allele frequency)

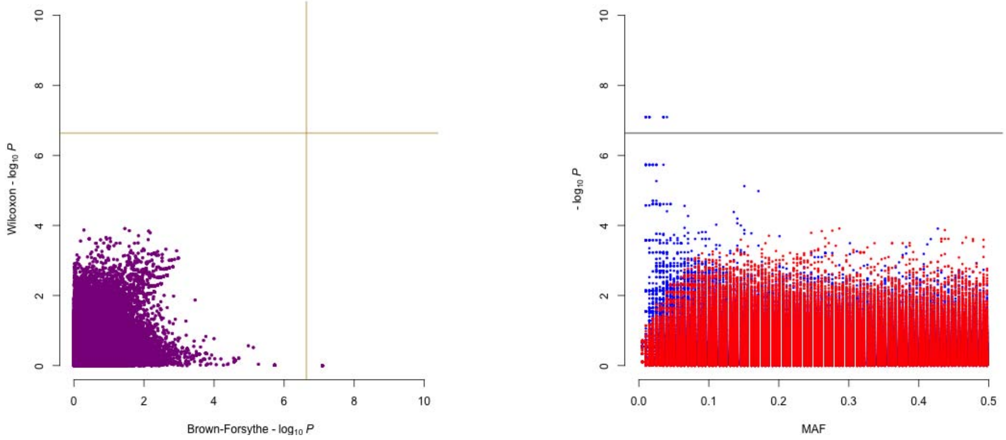

Supplementary Figure B61 - Summary of vGWA results for 159: Silique 22

Histogram of the phenotype & QQ plots of the Brown-Forsythe *p*-values (blue: nominal *p*-values, green: genomic controlled)

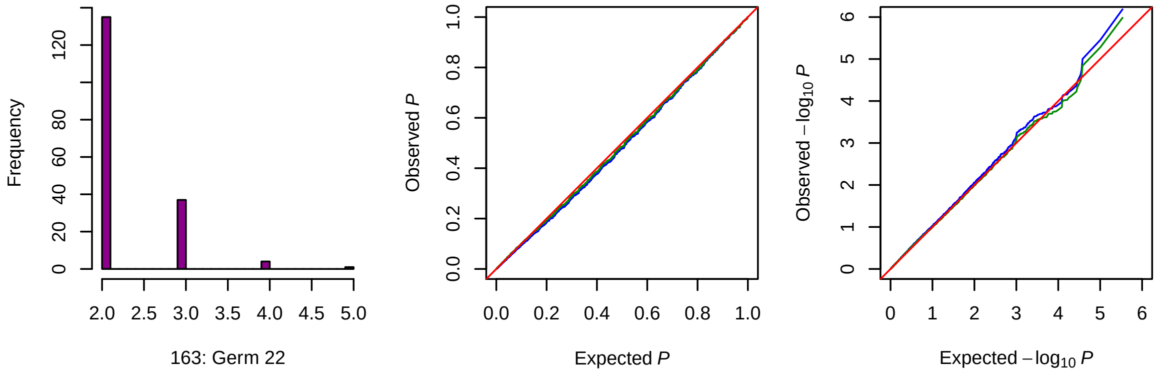

Brown-Forsythe (colored, behind) & Wilcoxon (yellow, in front) results, genomic controlled

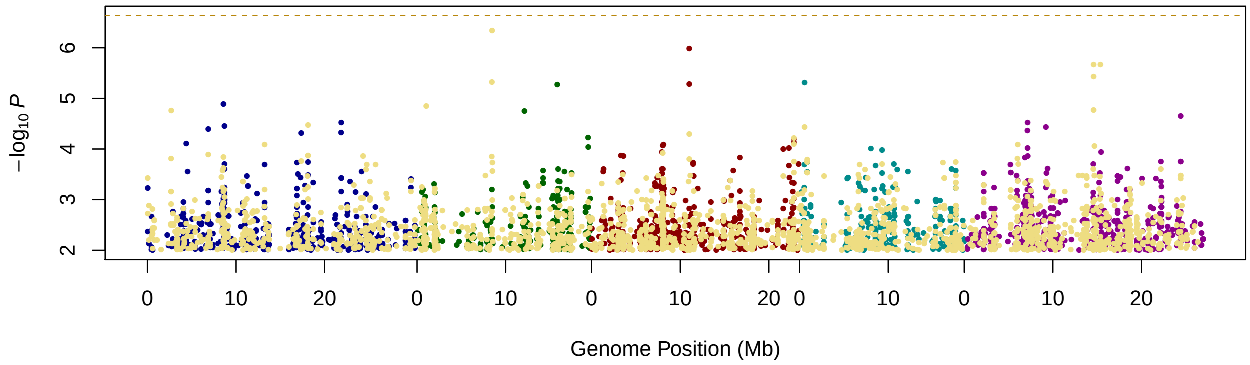

Comparison of Brown-Forsythe & Wilcoxon *p*-values (MAF: minor allele frequency)

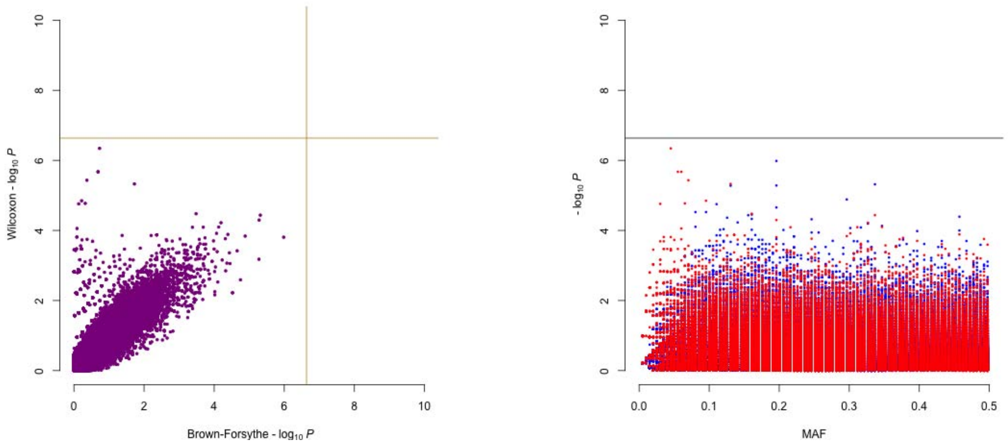

Supplementary Figure B62 - Summary of vGWA results for 163: Germ 22

Histogram of the phenotype & QQ plots of the Brown-Forsythe *p*-values (blue: nominal *p*-values, green: genomic controlled)

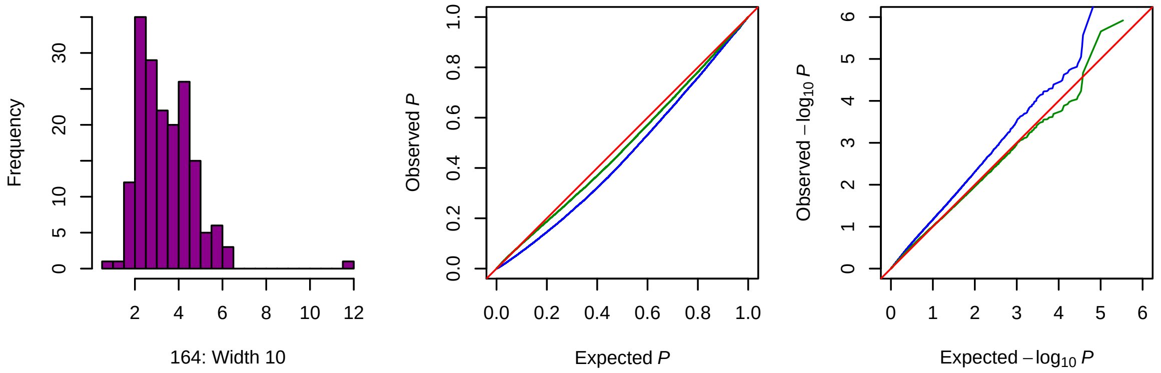

Brown-Forsythe (colored, behind) & Wilcoxon (yellow, in front) results, genomic controlled

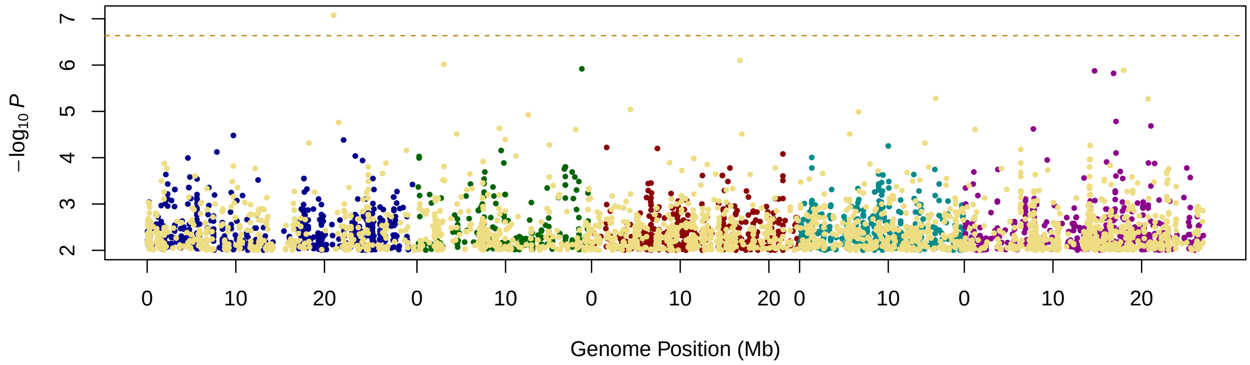

Comparison of Brown-Forsythe & Wilcoxon *p*-values (MAF: minor allele frequency)

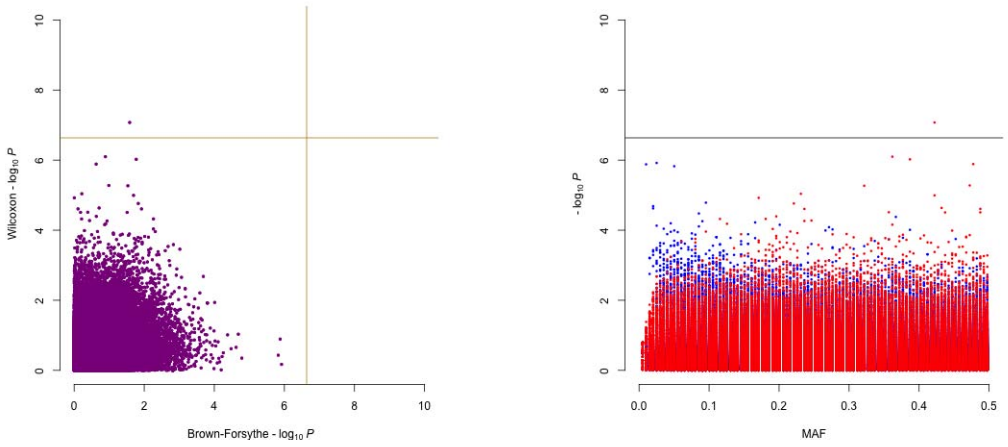

Supplementary Figure B63 - Summary of vGWA results for 164: Width 10

Histogram of the phenotype & QQ plots of the Brown-Forsythe *p*-values (blue: nominal *p*-values, green: genomic controlled)

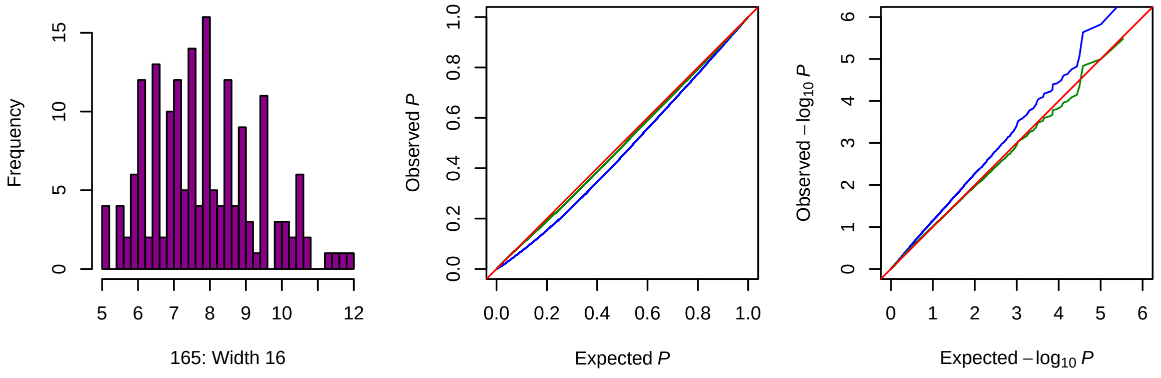

Brown-Forsythe (colored, behind) & Wilcoxon (yellow, in front) results, genomic controlled

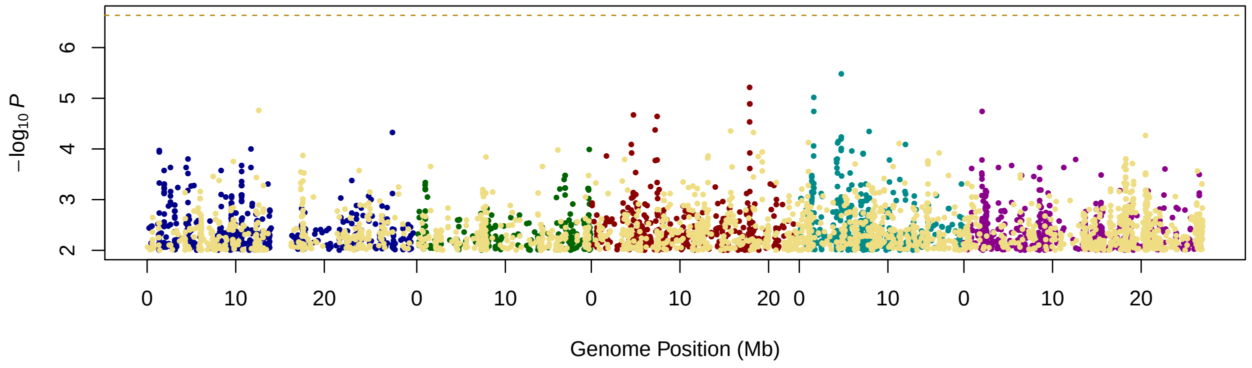

Comparison of Brown-Forsythe & Wilcoxon *p*-values (MAF: minor allele frequency)

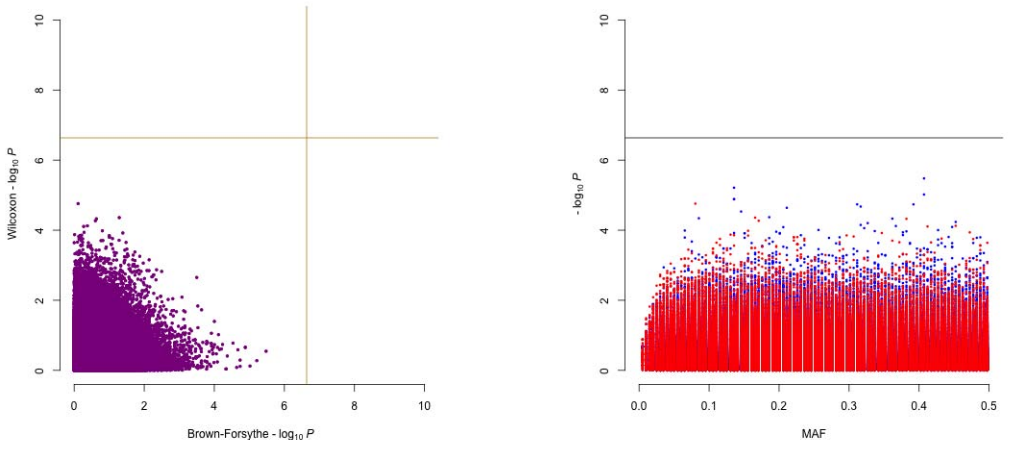

Supplementary Figure B64 - Summary of vGWA results for 165: Width 16

Histogram of the phenotype & QQ plots of the Brown-Forsythe *p*-values (blue: nominal *p*-values, green: genomic controlled)

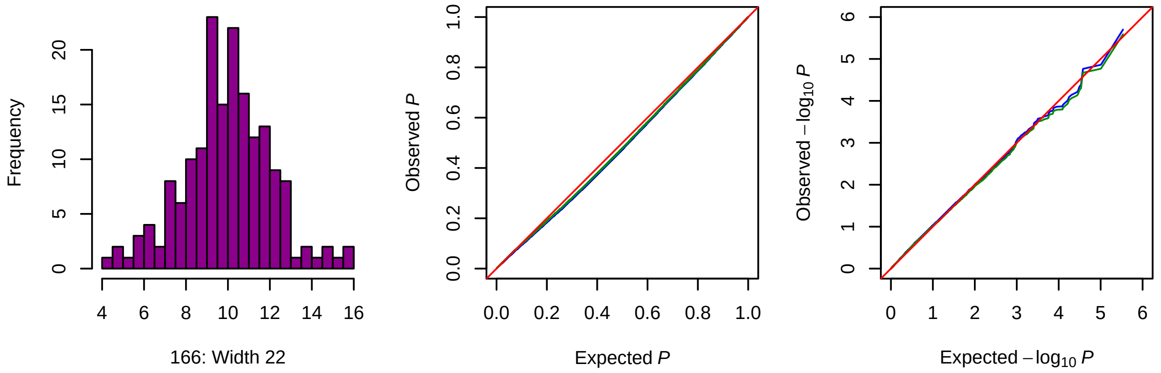

Brown-Forsythe (colored, behind) & Wilcoxon (yellow, in front) results, genomic controlled

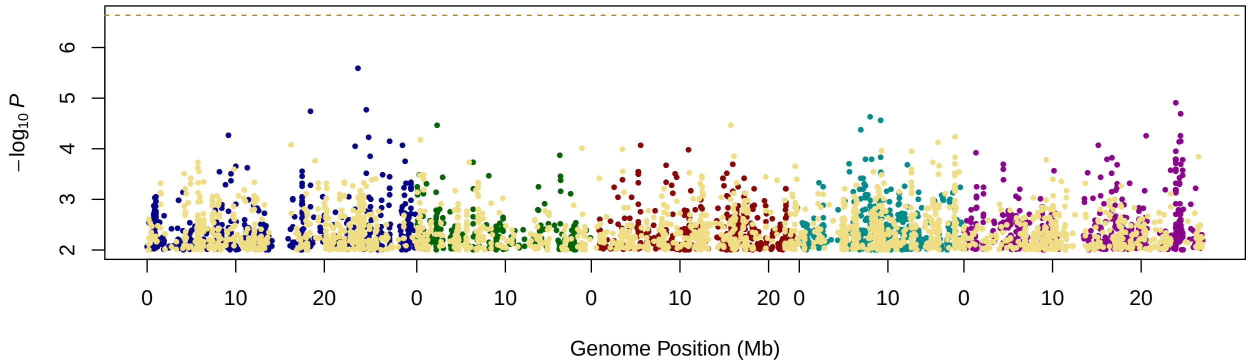

Comparison of Brown-Forsythe & Wilcoxon *p*-values (MAF: minor allele frequency)

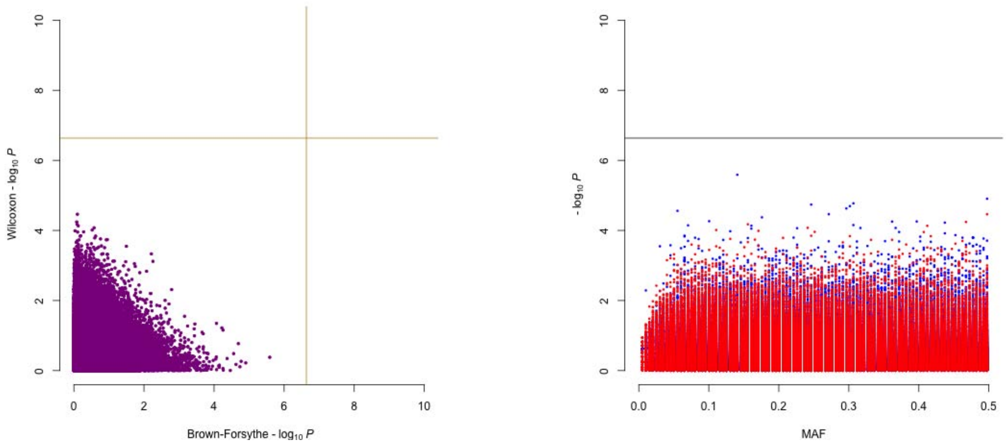

Supplementary Figure B65 - Summary of vGWA results for 166: Width 22

Histogram of the phenotype & QQ plots of the Brown-Forsythe *p*-values (blue: nominal *p*-values, green: genomic controlled)

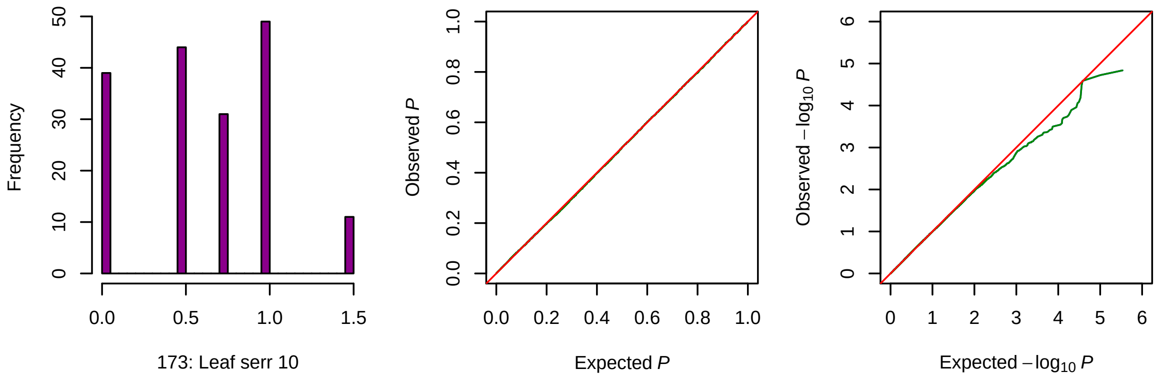

Brown-Forsythe (colored, behind) & Wilcoxon (yellow, in front) results, genomic controlled

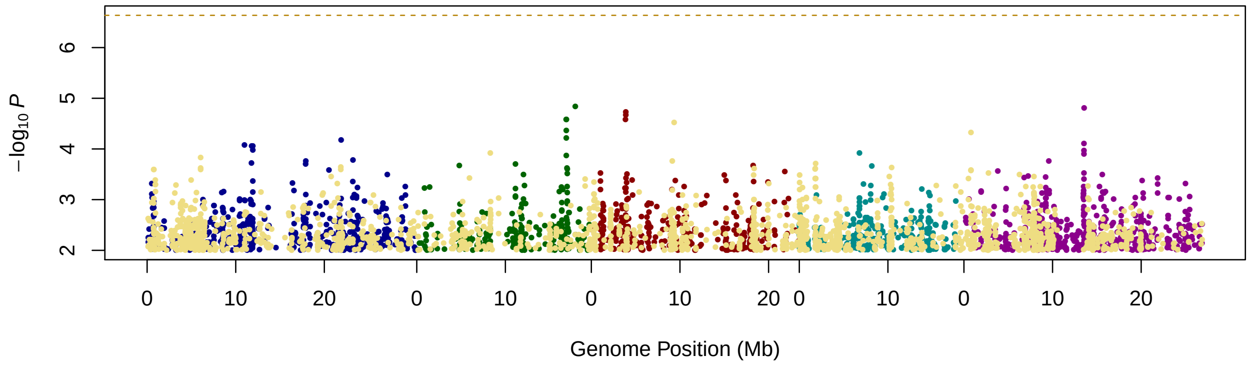

Comparison of Brown-Forsythe & Wilcoxon *p*-values (MAF: minor allele frequency)

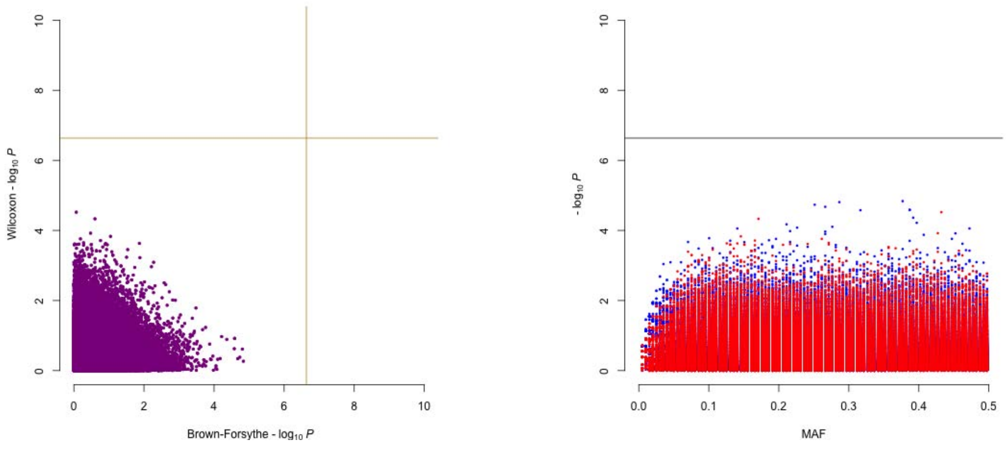

Supplementary Figure B66 - Summary of vGWA results for 173: Leaf serr 10

Histogram of the phenotype & QQ plots of the Brown-Forsythe *p*-values (blue: nominal *p*-values, green: genomic controlled)

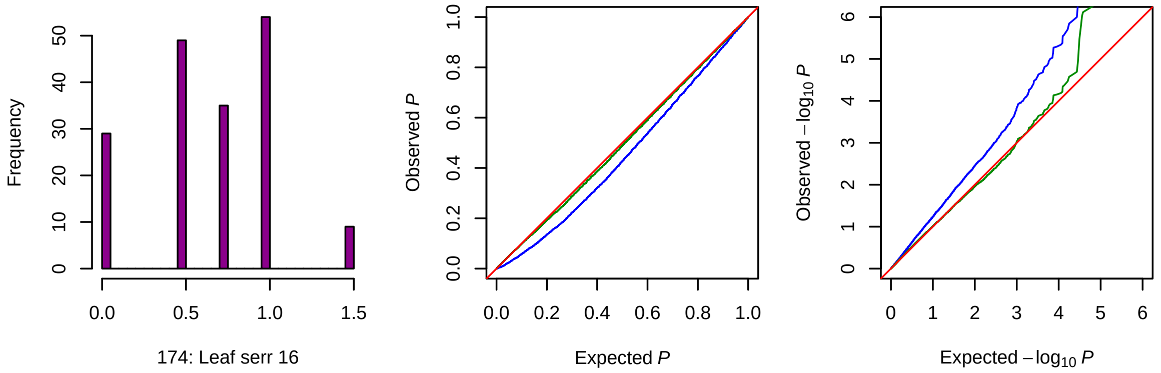

Brown-Forsythe (colored, behind) & Wilcoxon (yellow, in front) results, genomic controlled

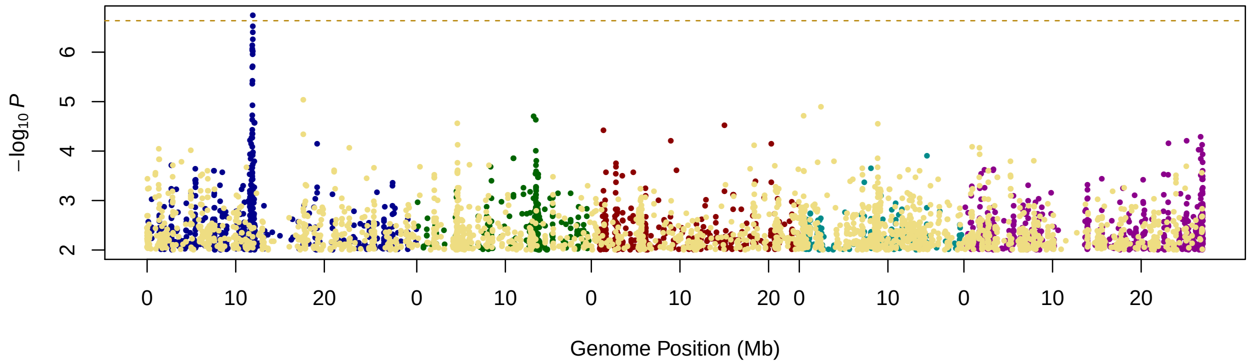

Comparison of Brown-Forsythe & Wilcoxon *p*-values (MAF: minor allele frequency)

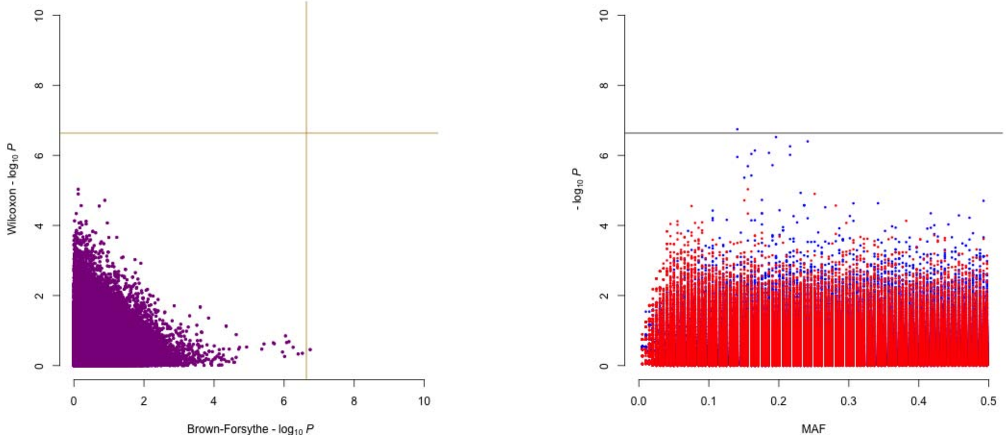

Supplementary Figure B67 - Summary of vGWA results for 174: Leaf serr 16

Histogram of the phenotype & QQ plots of the Brown-Forsythe *p*-values (blue: nominal *p*-values, green: genomic controlled)

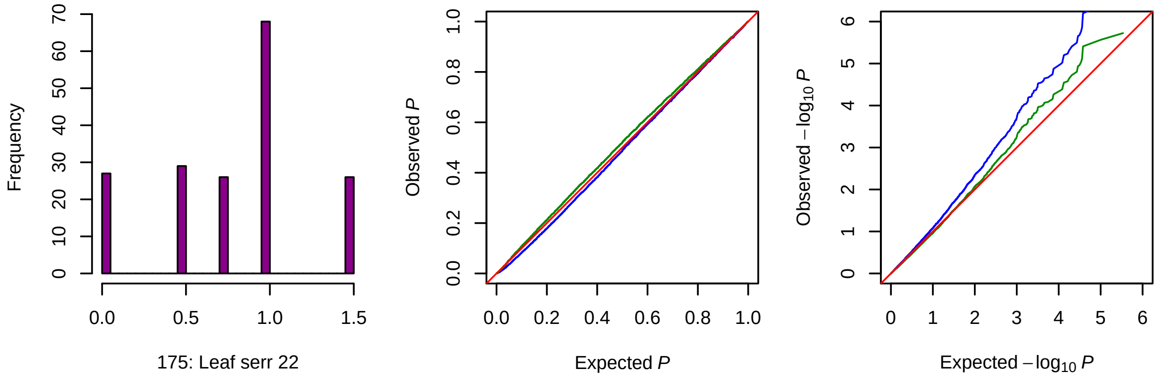

Brown-Forsythe (colored, behind) & Wilcoxon (yellow, in front) results, genomic controlled

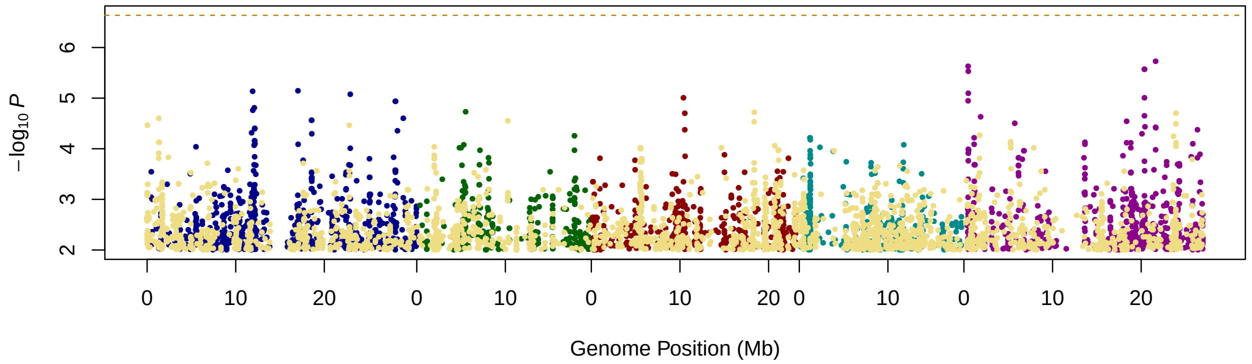

Comparison of Brown-Forsythe & Wilcoxon *p*-values (MAF: minor allele frequency)

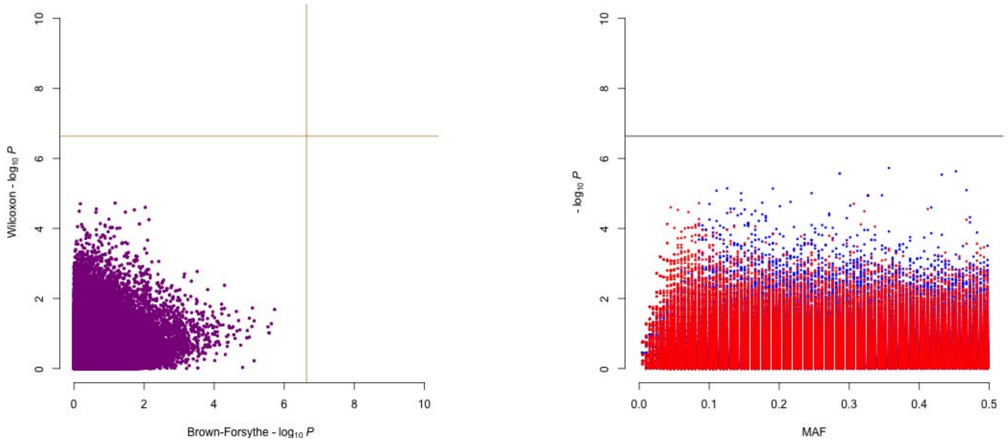

Supplementary Figure B68 - Summary of vGWA results for 175: Leaf serr 22

Histogram of the phenotype & QQ plots of the Brown-Forsythe *p*-values (blue: nominal *p*-values, green: genomic controlled)

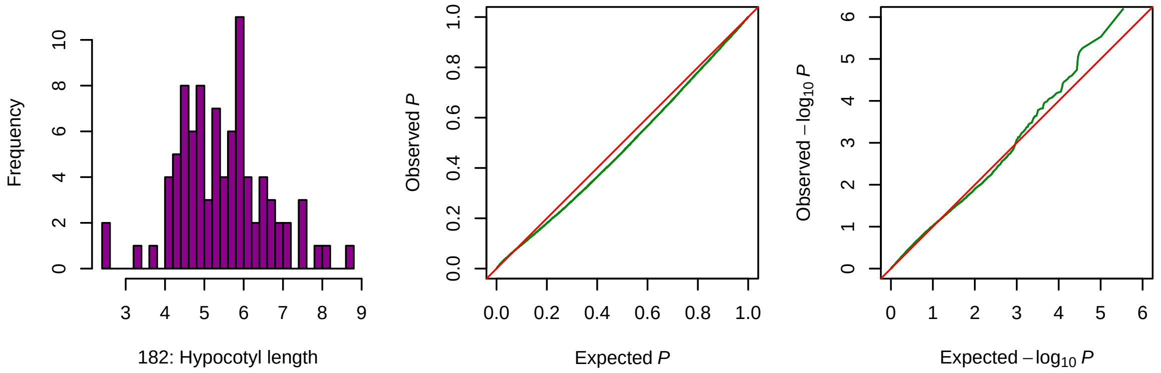

Brown-Forsythe (colored, behind) & Wilcoxon (yellow, in front) results, genomic controlled

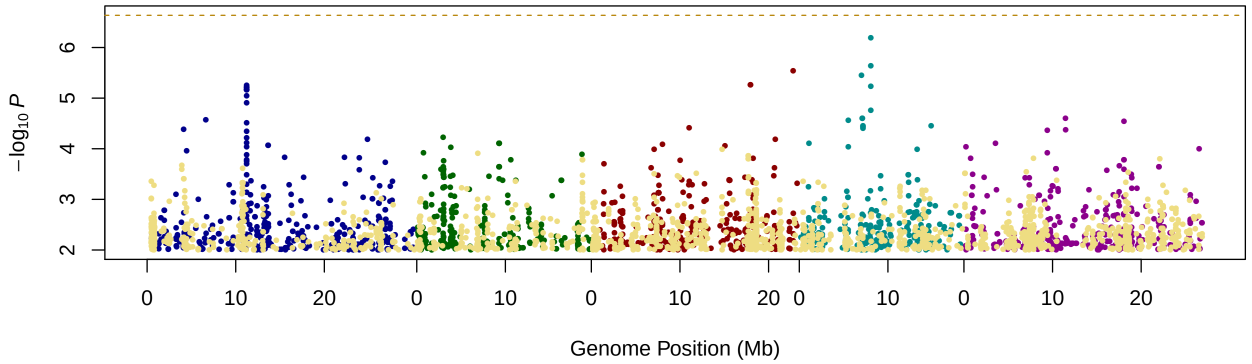

Comparison of Brown-Forsythe & Wilcoxon *p*-values (MAF: minor allele frequency)

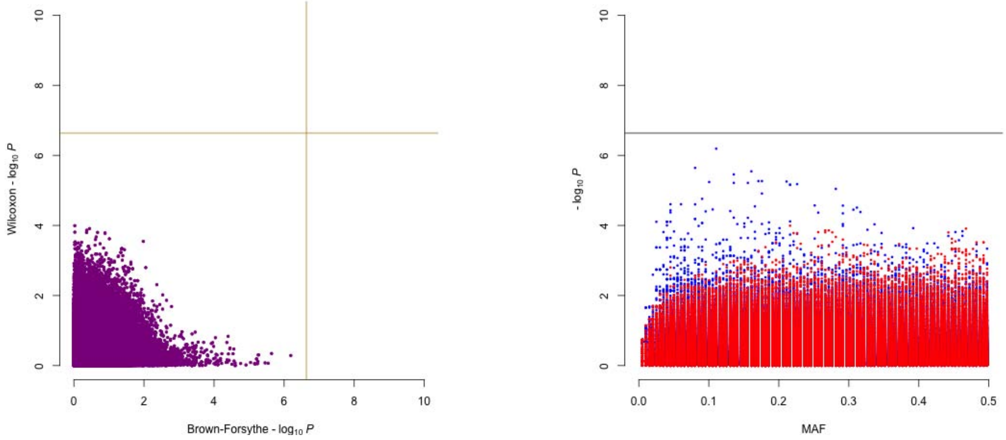

Supplementary Figure B69 - Summary of vGWA results for 182: Hypocotyl length

Histogram of the phenotype & QQ plots of the Brown-Forsythe *p*-values (blue: nominal *p*-values, green: genomic controlled)

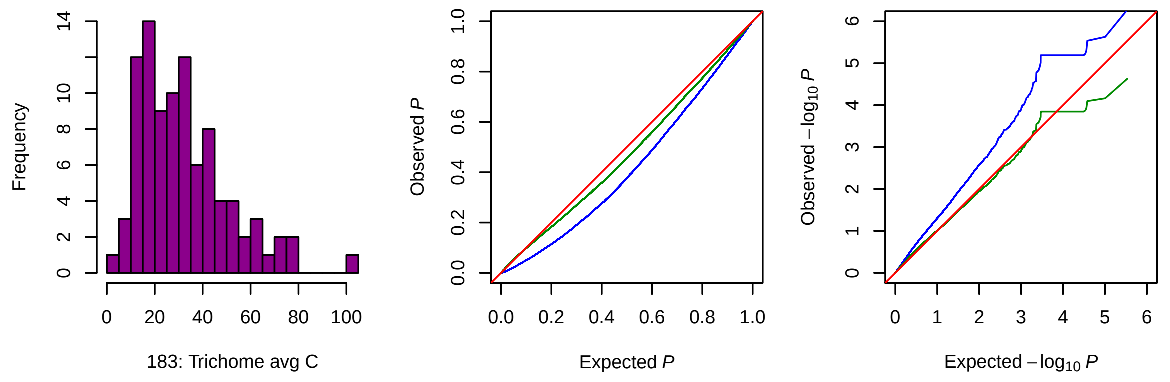

Brown-Forsythe (colored, behind) & Wilcoxon (yellow, in front) results, genomic controlled

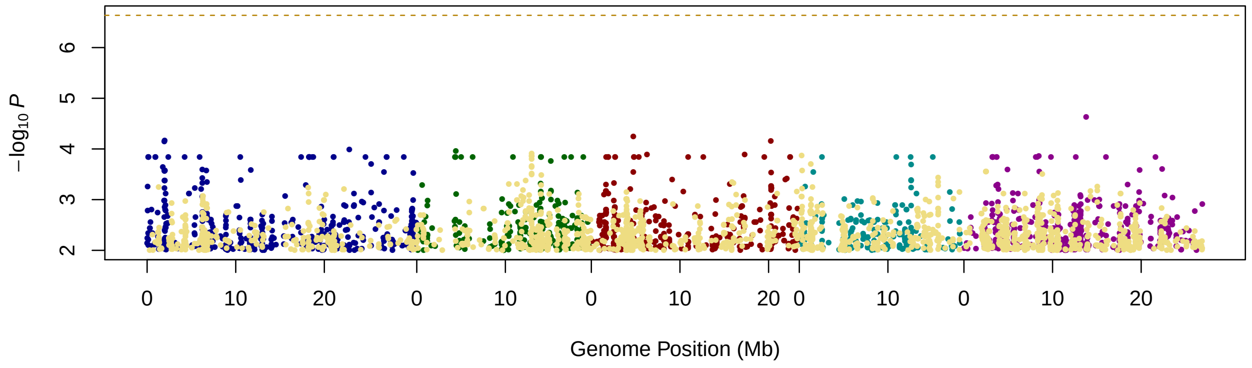

Comparison of Brown-Forsythe & Wilcoxon *p*-values (MAF: minor allele frequency)

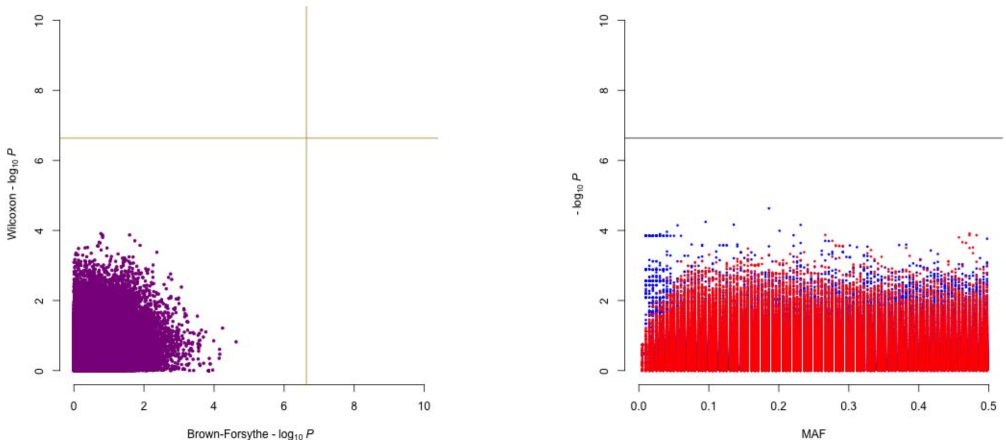

Supplementary Figure B70 - Summary of vGWA results for 183: Trichome avg C

Histogram of the phenotype & QQ plots of the Brown-Forsythe *p*-values (blue: nominal *p*-values, green: genomic controlled)

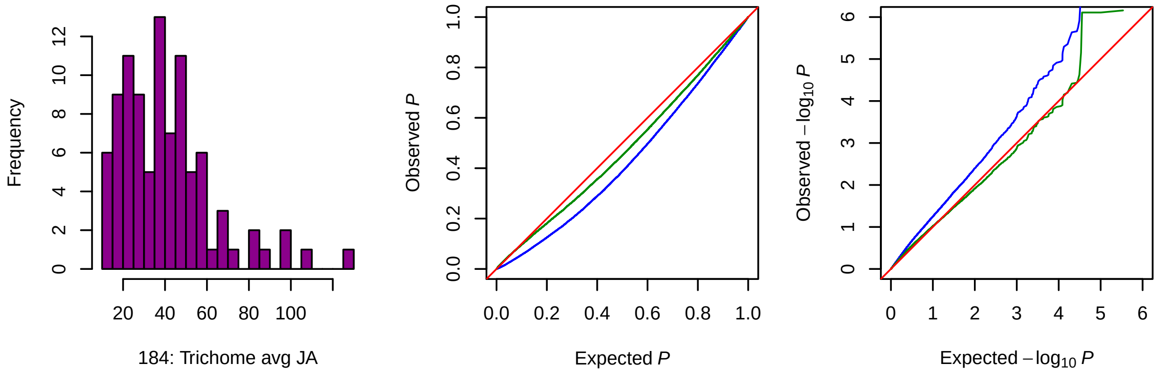

Brown-Forsythe (colored, behind) & Wilcoxon (yellow, in front) results, genomic controlled

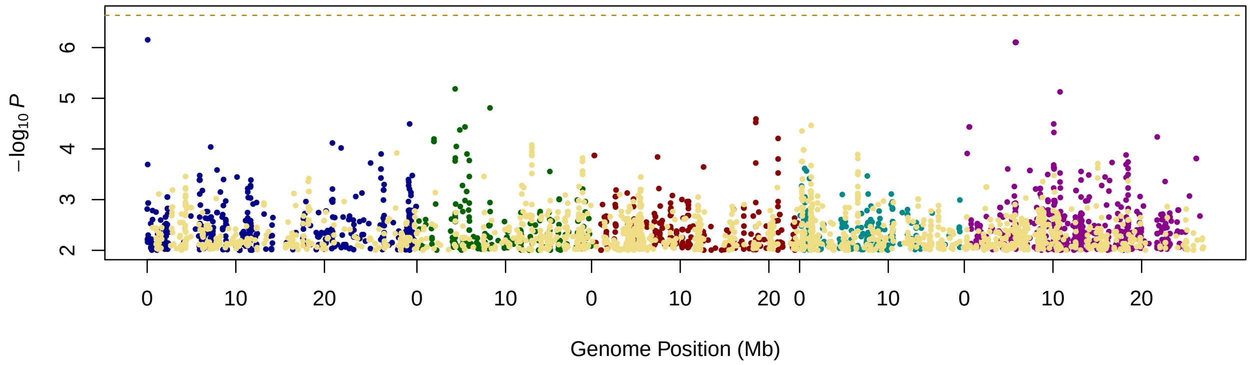

Comparison of Brown-Forsythe & Wilcoxon *p*-values (MAF: minor allele frequency)

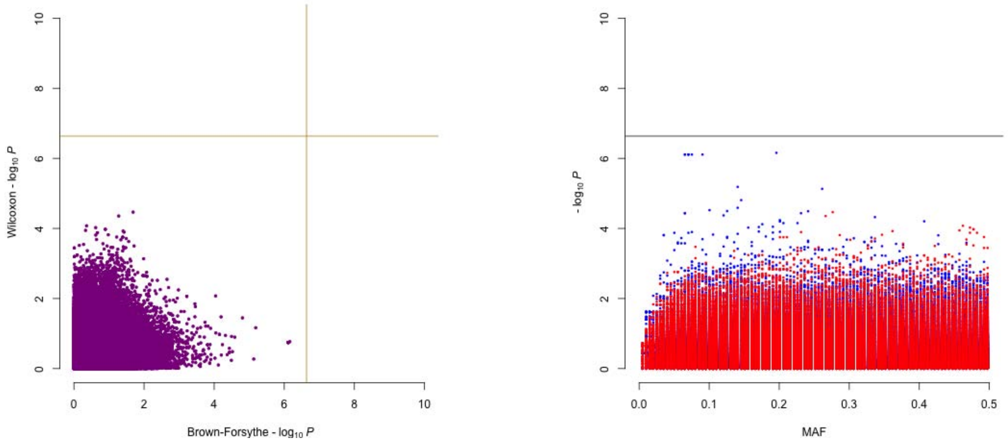

Supplementary Figure B71 - Summary of vGWA results for 184: Trichome avg JA

Histogram of the phenotype & QQ plots of the Brown-Forsythe *p*-values (blue: nominal *p*-values, green: genomic controlled)

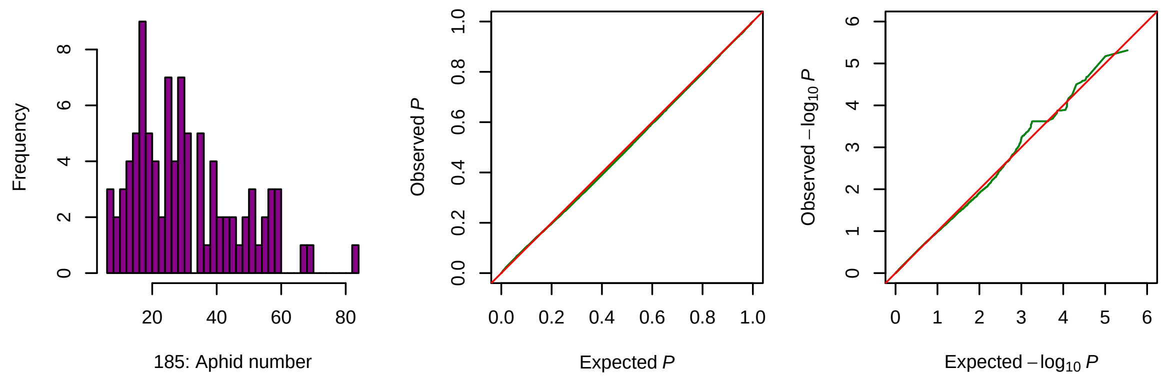

Brown-Forsythe (colored, behind) & Wilcoxon (yellow, in front) results, genomic controlled

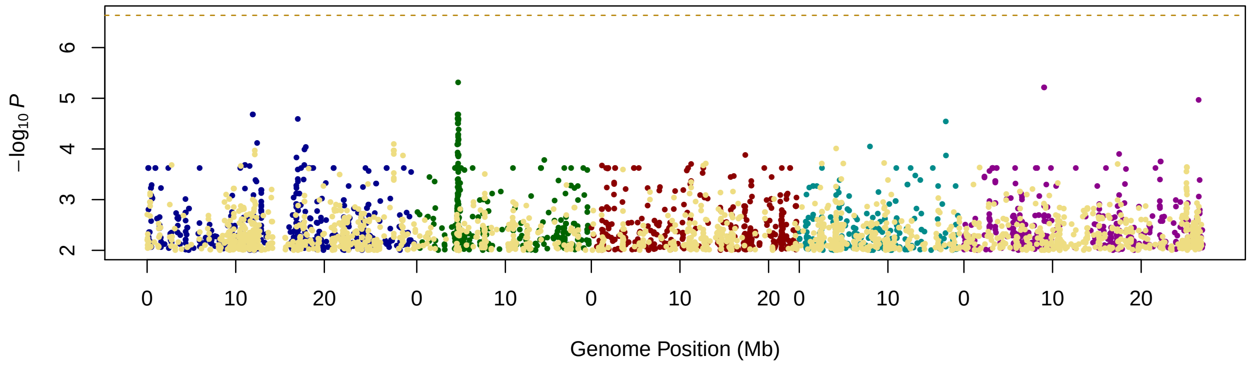

Comparison of Brown-Forsythe & Wilcoxon *p*-values (MAF: minor allele frequency)

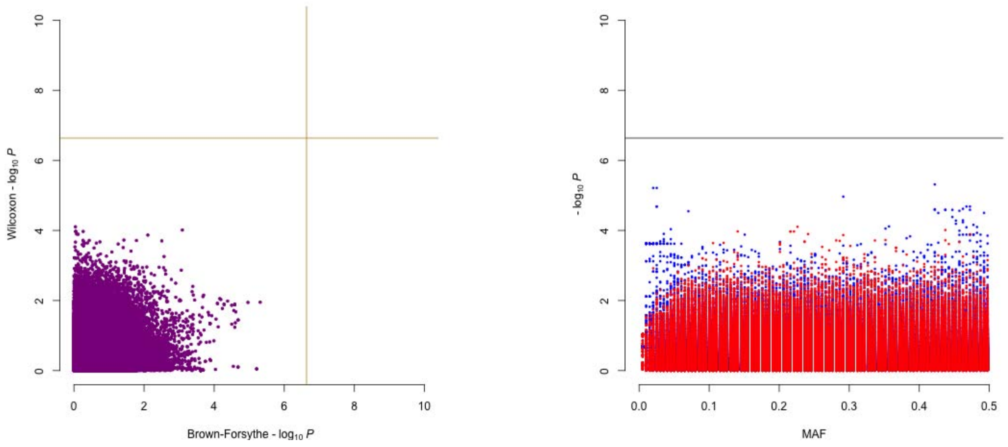

Supplementary Figure B72 - Summary of vGWA results for 185: Aphid number

Histogram of the phenotype & QQ plots of the Brown-Forsythe *p*-values (blue: nominal *p*-values, green: genomic controlled)

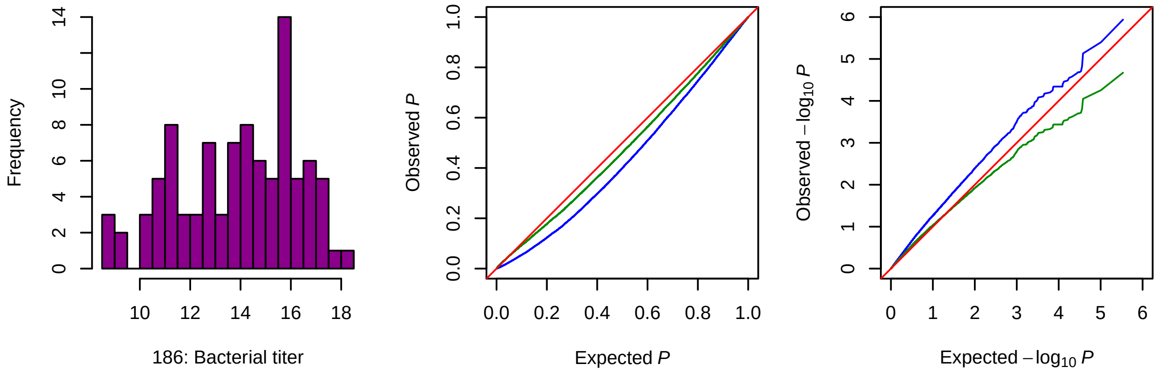

Brown-Forsythe (colored, behind) & Wilcoxon (yellow, in front) results, genomic controlled

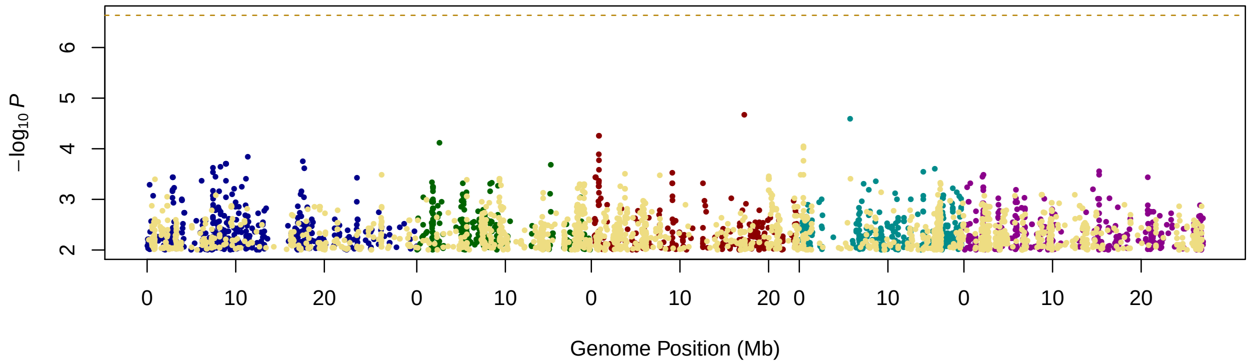

Comparison of Brown-Forsythe & Wilcoxon *p*-values (MAF: minor allele frequency)

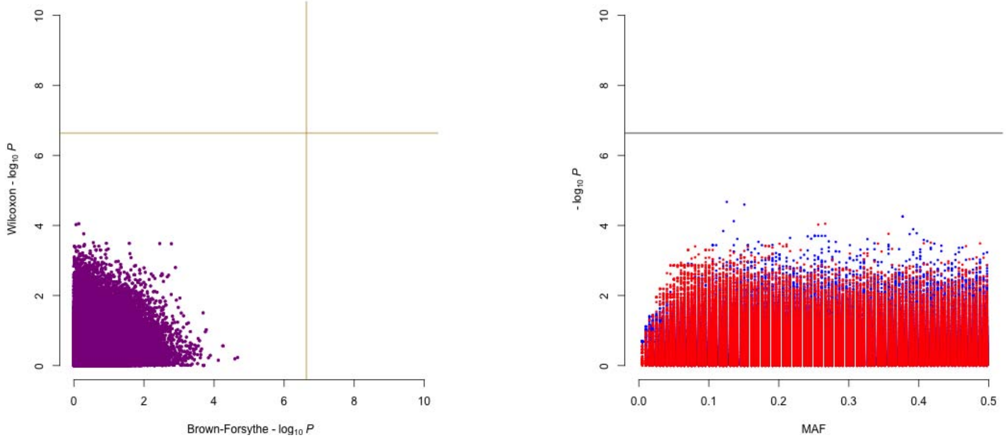

Supplementary Figure B73 - Summary of vGWA results for 186: Bacterial titer

Histogram of the phenotype & QQ plots of the Brown-Forsythe *p*-values (blue: nominal *p*-values, green: genomic controlled)

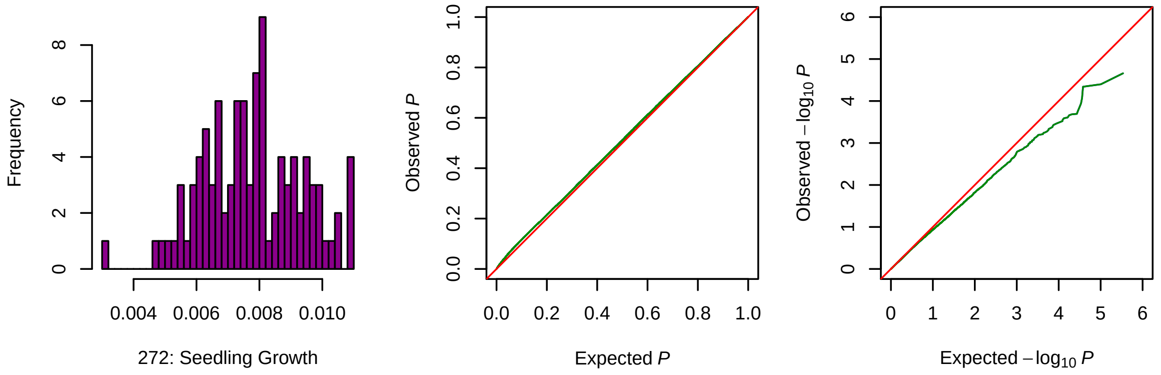

Brown-Forsythe (colored, behind) & Wilcoxon (yellow, in front) results, genomic controlled

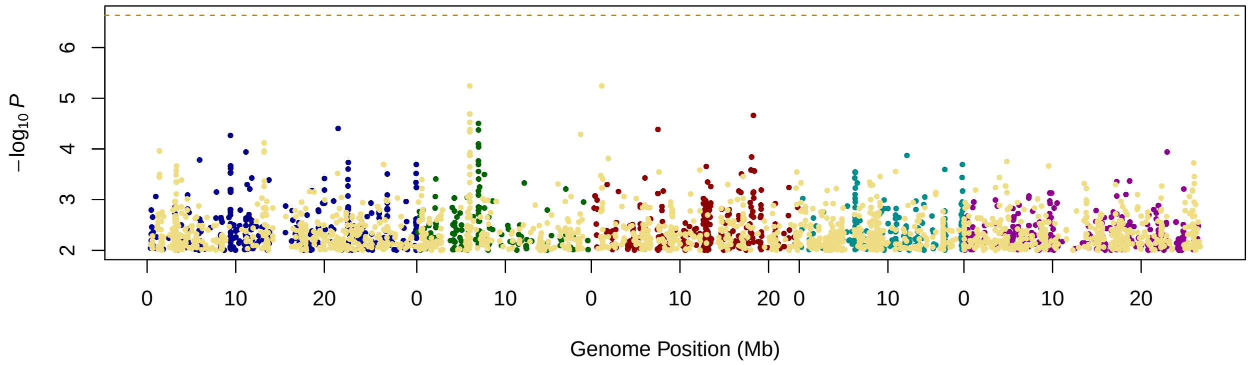

Comparison of Brown-Forsythe & Wilcoxon *p*-values (MAF: minor allele frequency)

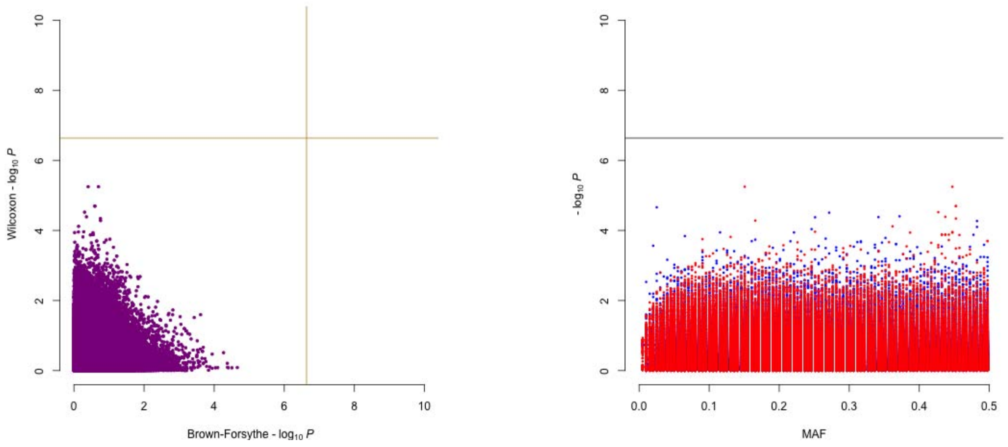

Supplementary Figure B74 - Summary of vGWA results for 272: Seedling Growth

Histogram of the phenotype & QQ plots of the Brown-Forsythe *p*-values (blue: nominal *p*-values, green: genomic controlled)

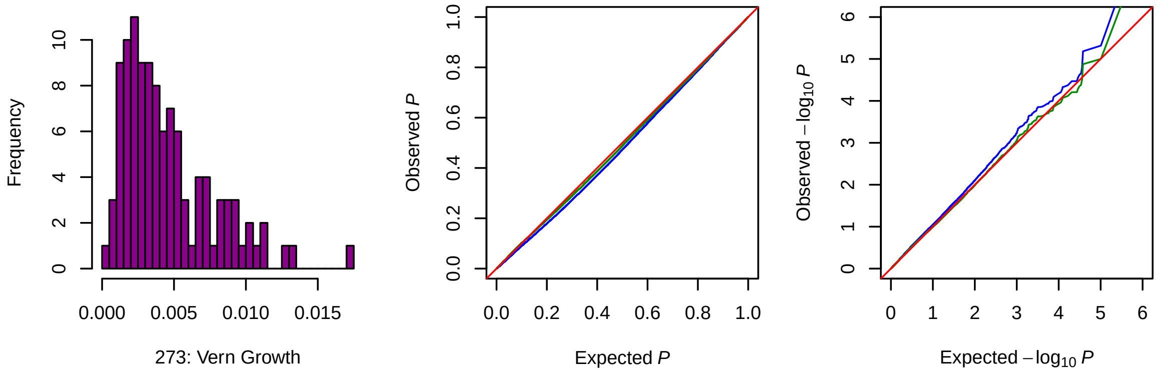

Brown-Forsythe (colored, behind) & Wilcoxon (yellow, in front) results, genomic controlled

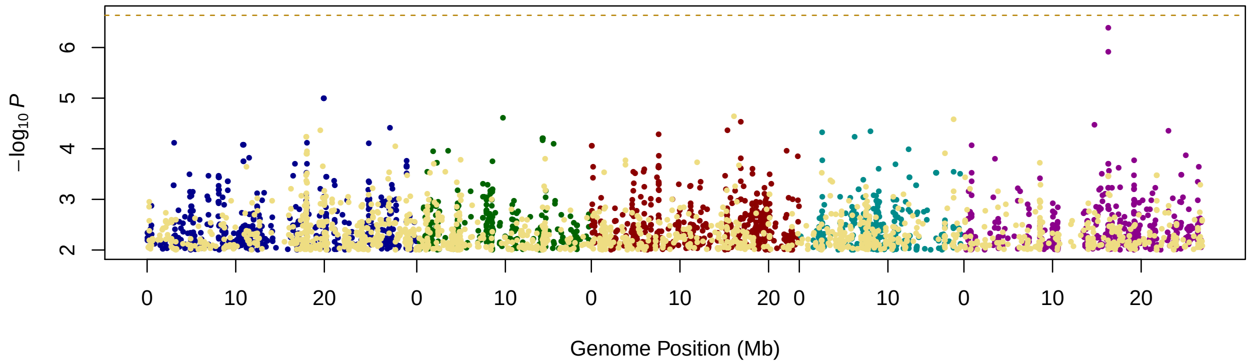

Comparison of Brown-Forsythe & Wilcoxon *p*-values (MAF: minor allele frequency)

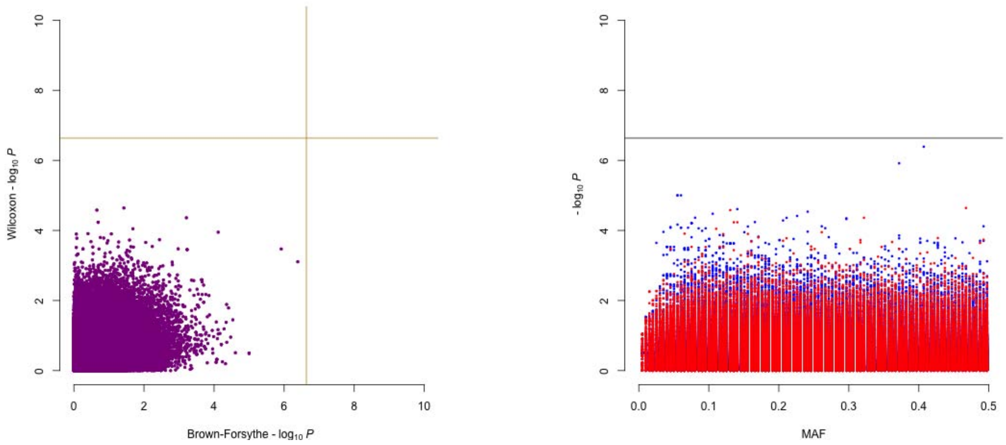

Supplementary Figure B75 - Summary of vGWA results for 273: Vern Growth

Histogram of the phenotype & QQ plots of the Brown-Forsythe *p*-values (blue: nominal *p*-values, green: genomic controlled)

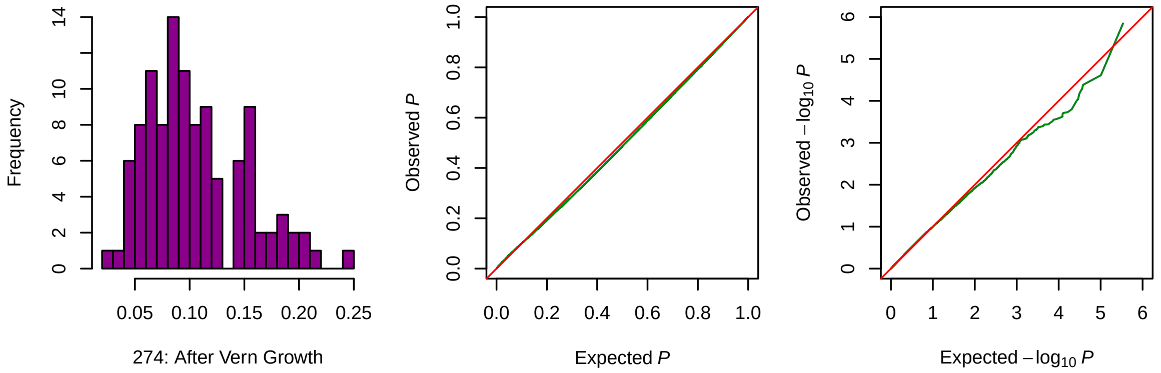

Brown-Forsythe (colored, behind) & Wilcoxon (yellow, in front) results, genomic controlled

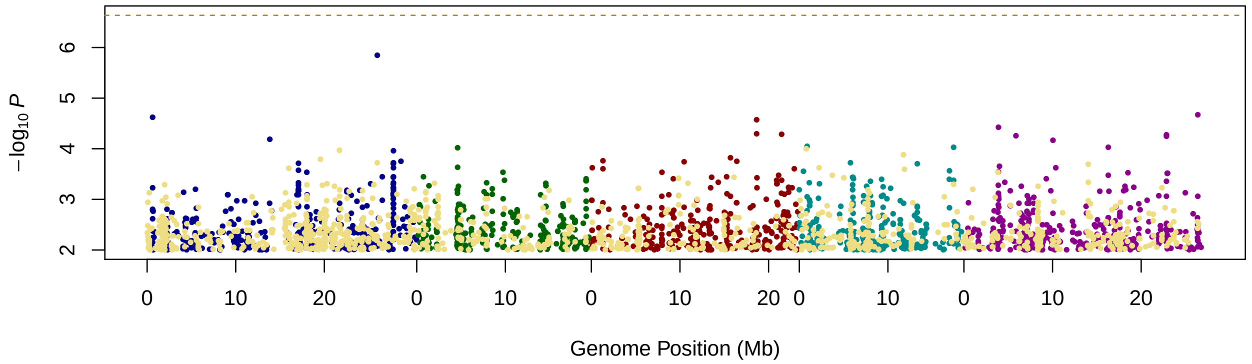

Comparison of Brown-Forsythe & Wilcoxon *p*-values (MAF: minor allele frequency)

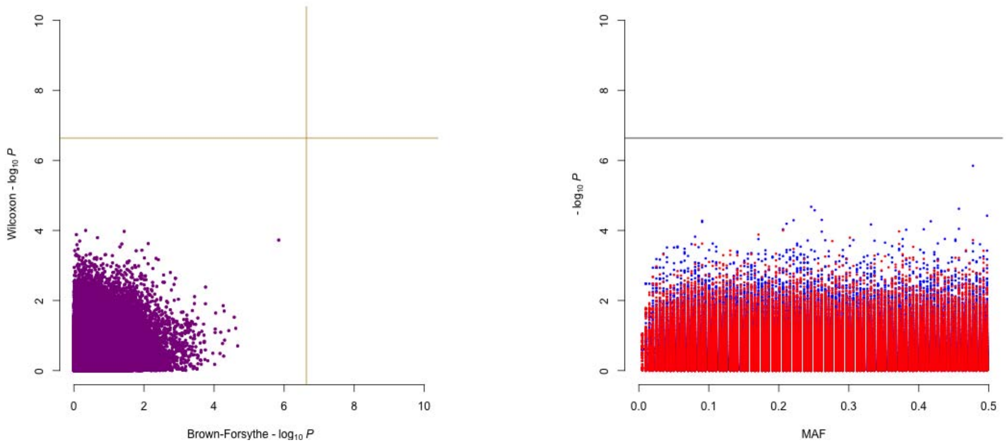

Supplementary Figure B76 - Summary of vGWA results for 274: After Vern Growth

Histogram of the phenotype & QQ plots of the Brown-Forsythe *p*-values (blue: nominal *p*-values, green: genomic controlled)

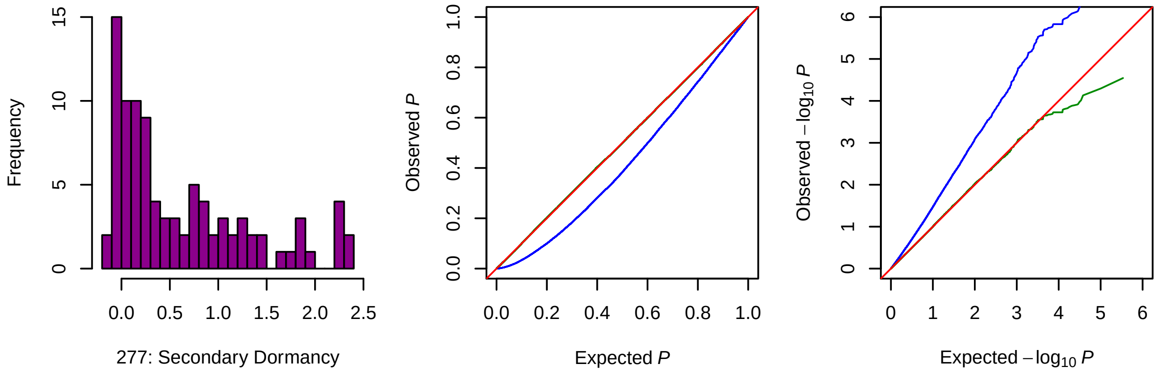

Brown-Forsythe (colored, behind) & Wilcoxon (yellow, in front) results, genomic controlled

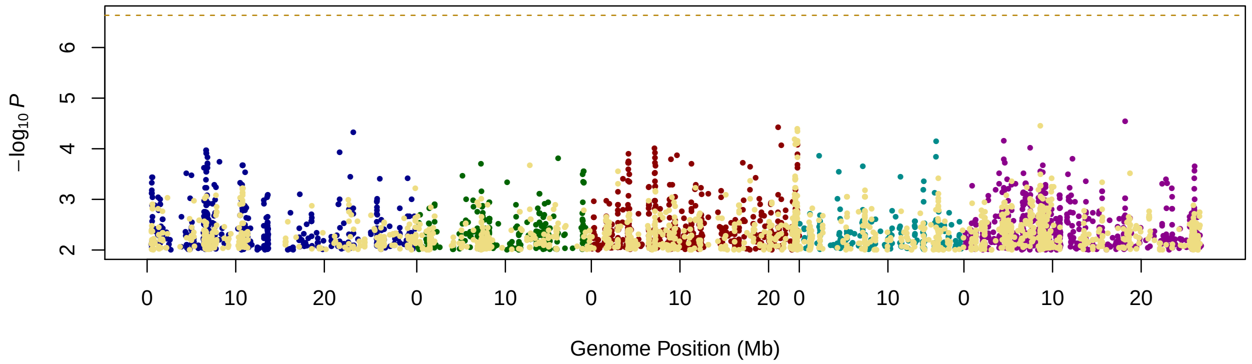

Comparison of Brown-Forsythe & Wilcoxon *p*-values (MAF: minor allele frequency)

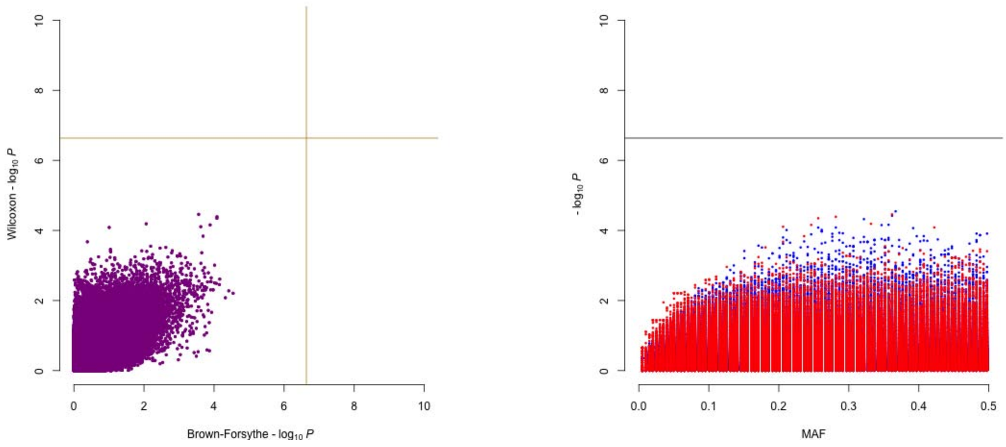

Supplementary Figure B77 - Summary of vGWA results for 277: Secondary Dormancy

Histogram of the phenotype & QQ plots of the Brown-Forsythe *p*-values (blue: nominal *p*-values, green: genomic controlled)

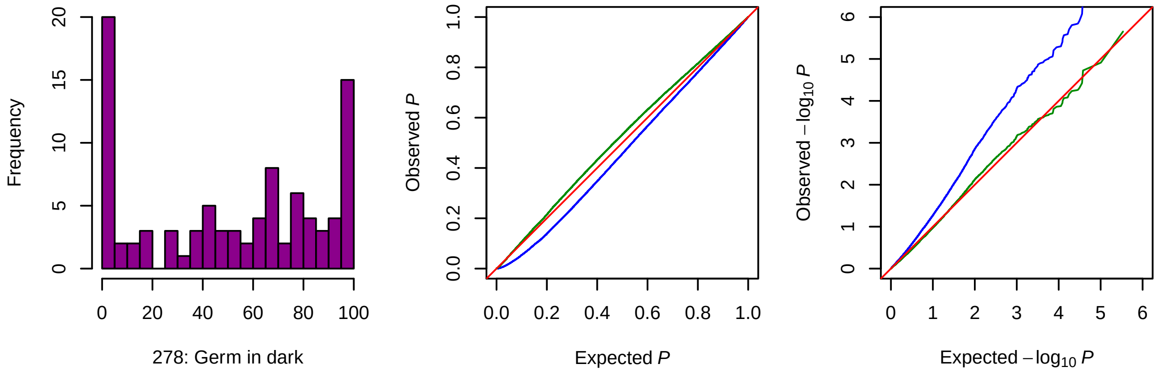

Brown-Forsythe (colored, behind) & Wilcoxon (yellow, in front) results, genomic controlled

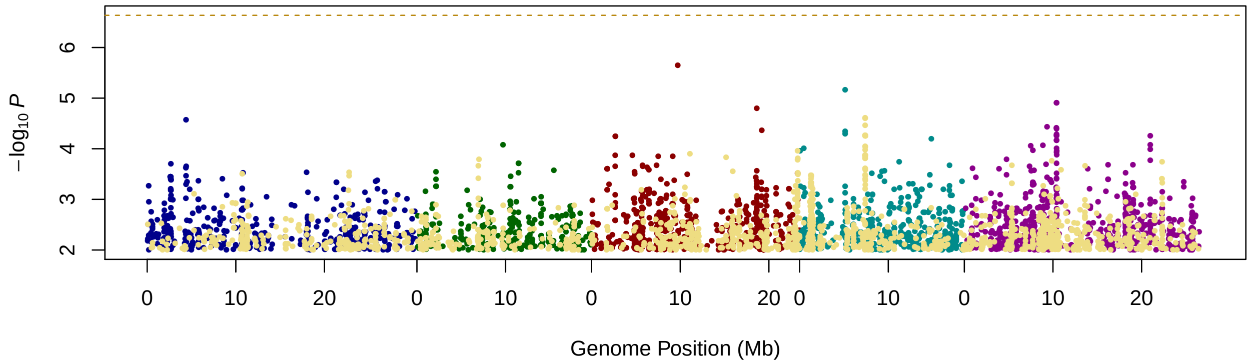

Comparison of Brown-Forsythe & Wilcoxon *p*-values (MAF: minor allele frequency)

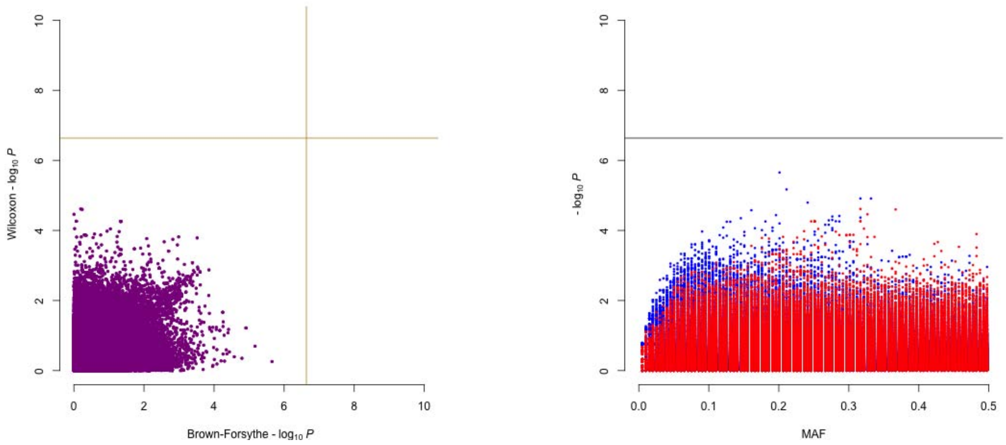

Supplementary Figure B78 - Summary of vGWA results for 278: Germ in dark

Histogram of the phenotype & QQ plots of the Brown-Forsythe *p*-values (blue: nominal *p*-values, green: genomic controlled)

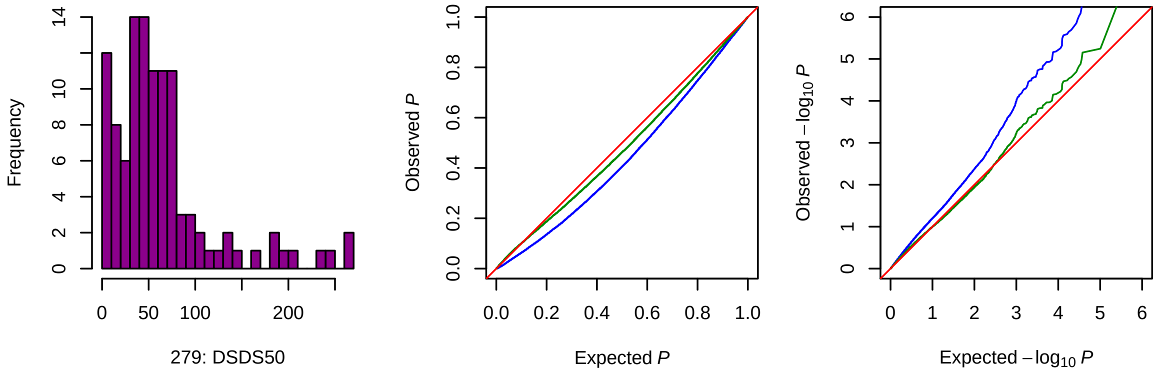

Brown-Forsythe (colored, behind) & Wilcoxon (yellow, in front) results, genomic controlled

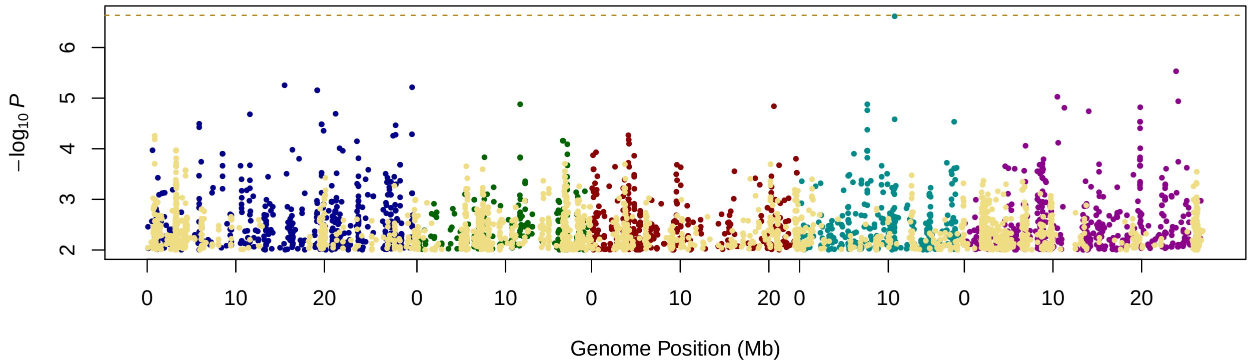

Comparison of Brown-Forsythe & Wilcoxon *p*-values (MAF: minor allele frequency)

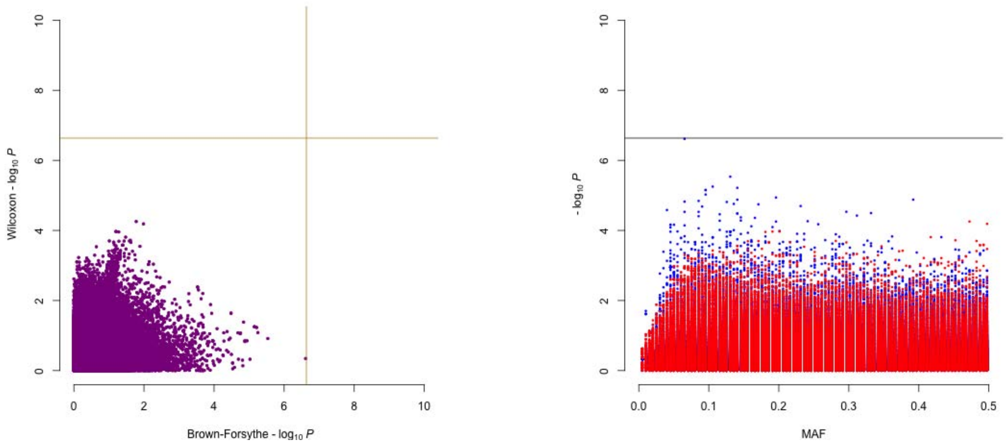

Supplementary Figure B79 - Summary of vGWA results for 279: DSDS50

Histogram of the phenotype & QQ plots of the Brown-Forsythe *p*-values (blue: nominal *p*-values, green: genomic controlled)

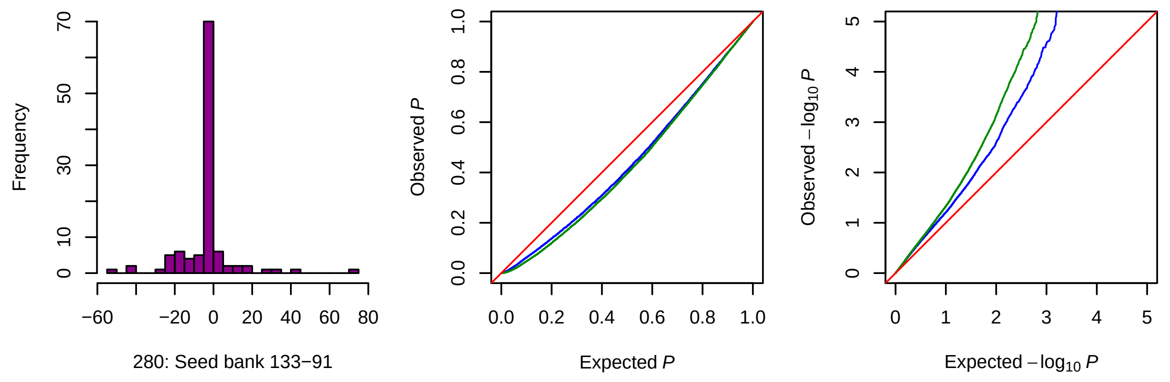

Brown-Forsythe (colored, behind) & Wilcoxon (yellow, in front) results, genomic controlled

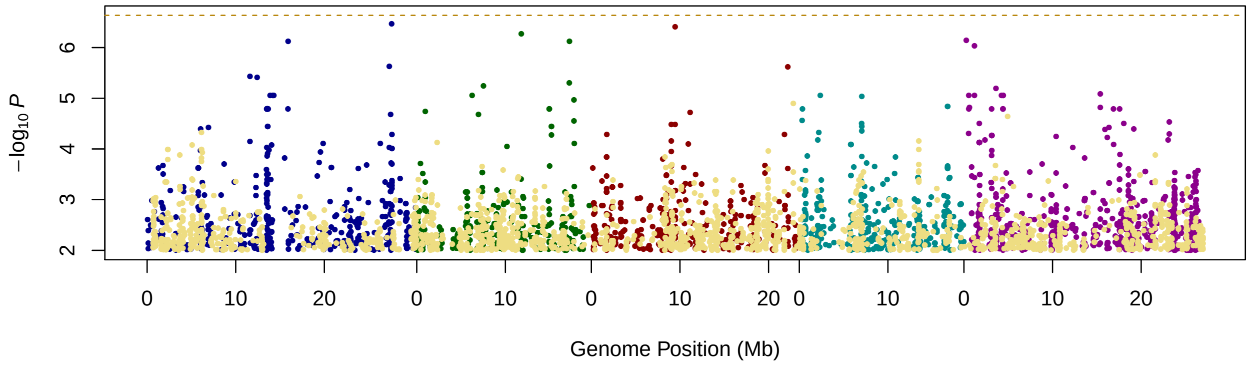

Comparison of Brown-Forsythe & Wilcoxon *p*-values (MAF: minor allele frequency)

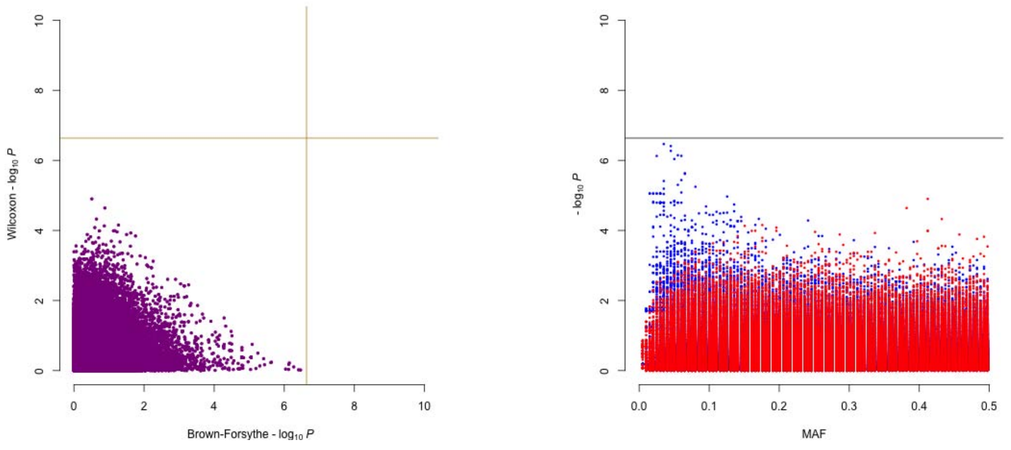

Supplementary Figure B80 - Summary of vGWA results for 280: Seed bank 133-91

Histogram of the phenotype & QQ plots of the Brown-Forsythe *p*-values (blue: nominal *p*-values, green: genomic controlled)

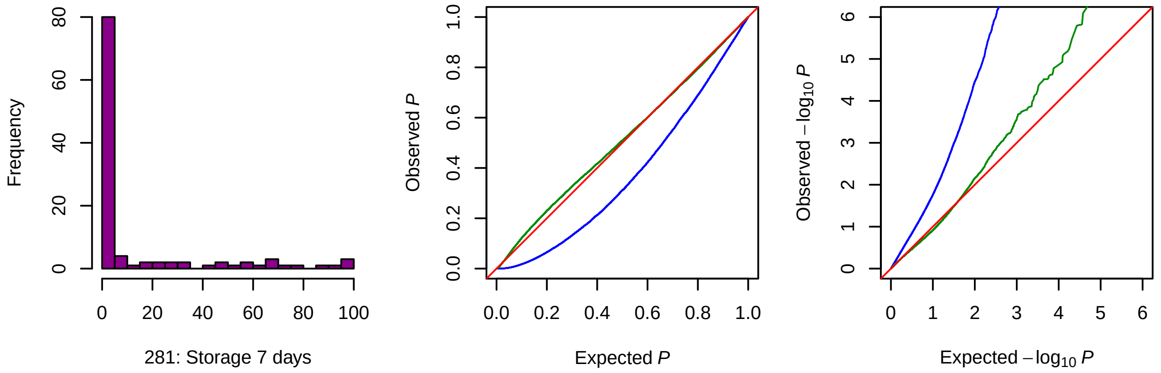

Brown-Forsythe (colored, behind) & Wilcoxon (yellow, in front) results, genomic controlled

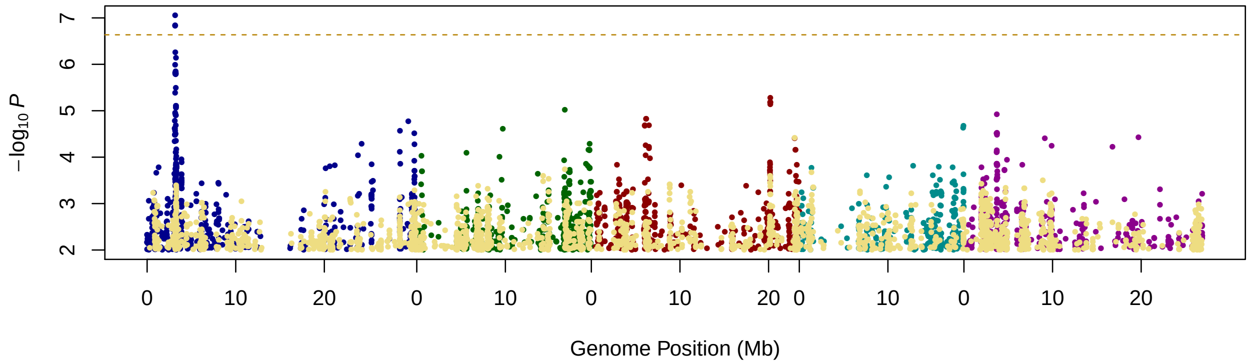

Comparison of Brown-Forsythe & Wilcoxon *p*-values (MAF: minor allele frequency)

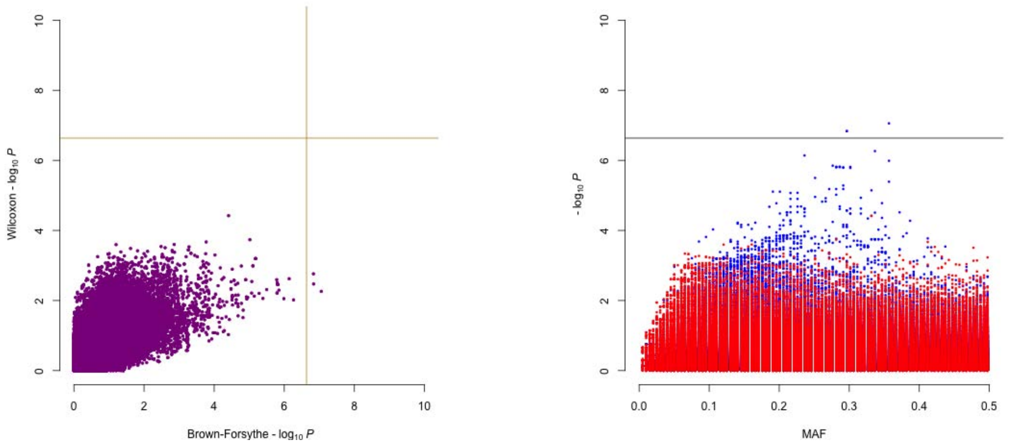

Supplementary Figure B81 - Summary of vGWA results for 281: Storage 7 days

Histogram of the phenotype & QQ plots of the Brown-Forsythe *p*-values (blue: nominal *p*-values, green: genomic controlled)

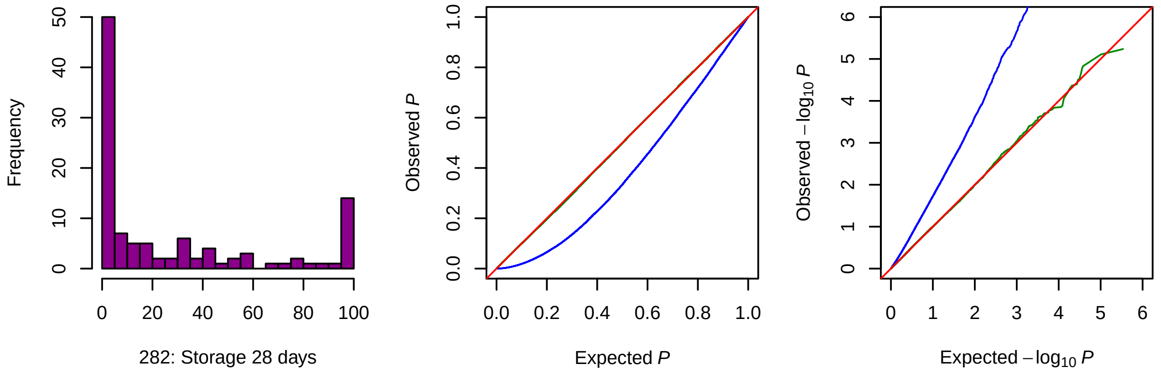

Brown-Forsythe (colored, behind) & Wilcoxon (yellow, in front) results, genomic controlled

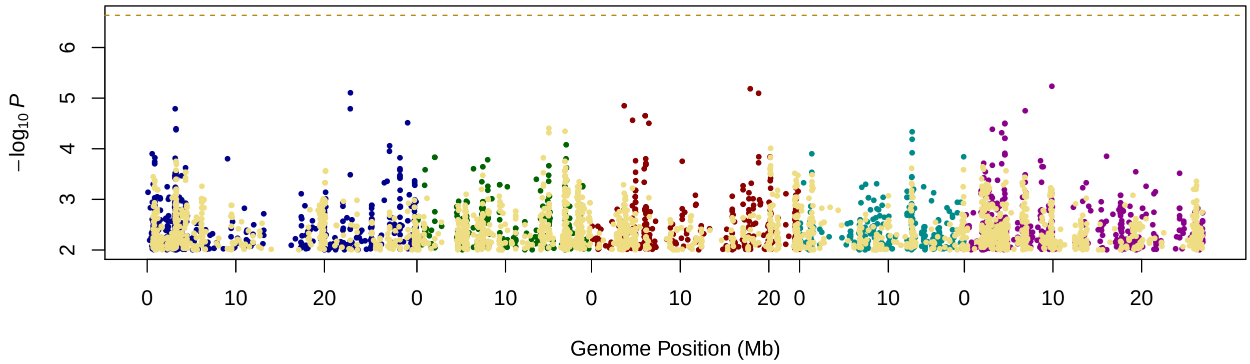

Comparison of Brown-Forsythe & Wilcoxon *p*-values (MAF: minor allele frequency)

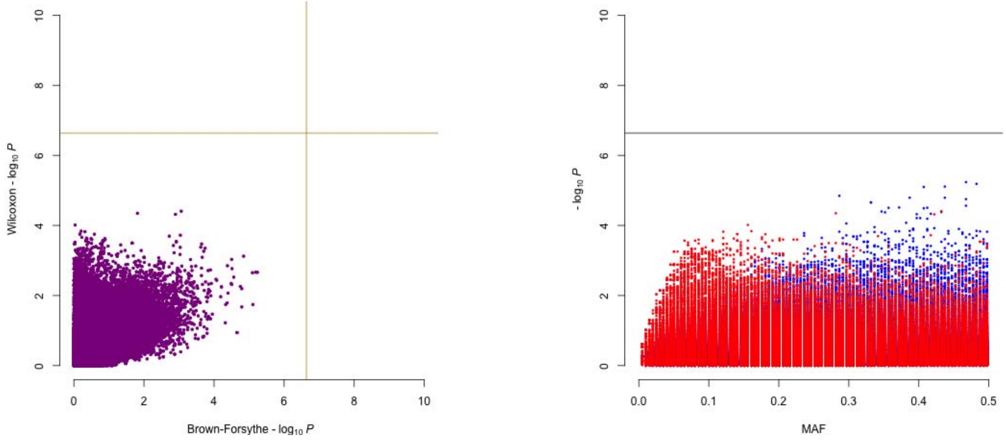

Supplementary Figure B82 - Summary of vGWA results for 282: Storage 28 days

Histogram of the phenotype & QQ plots of the Brown-Forsythe *p*-values (blue: nominal *p*-values, green: genomic controlled)

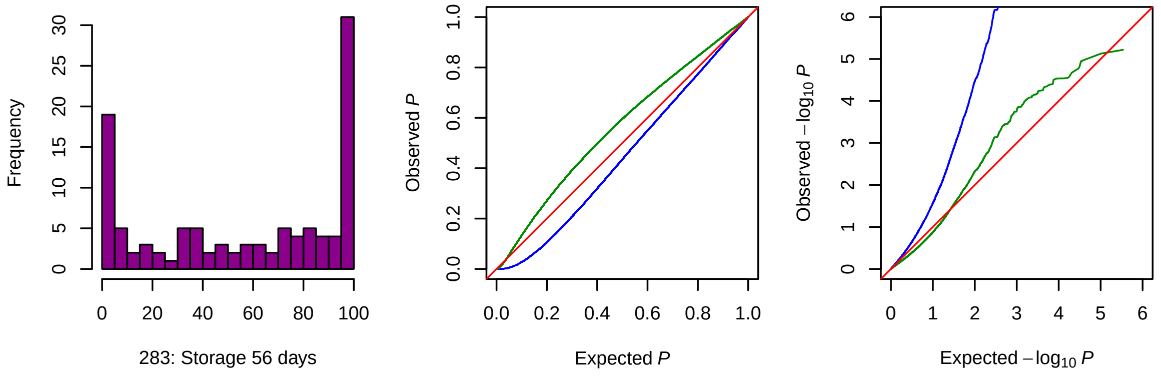

Brown-Forsythe (colored, behind) & Wilcoxon (yellow, in front) results, genomic controlled

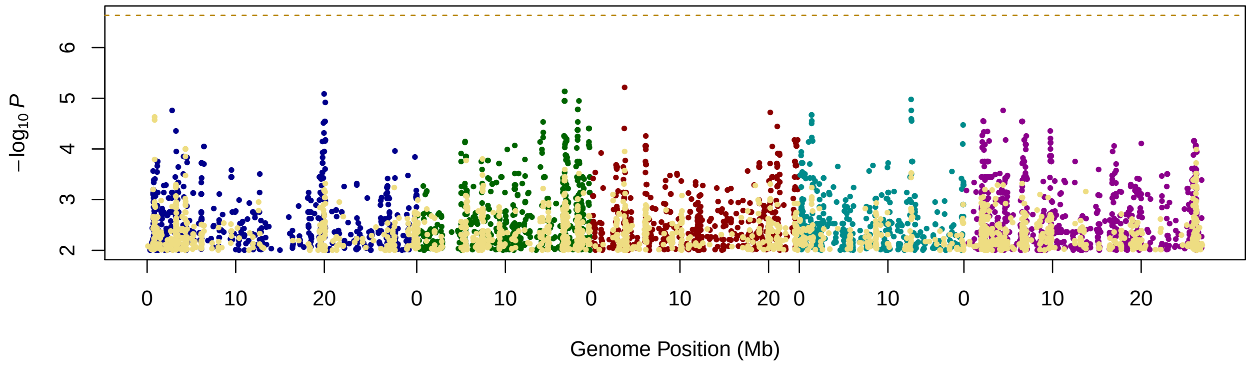

Comparison of Brown-Forsythe & Wilcoxon *p*-values (MAF: minor allele frequency)

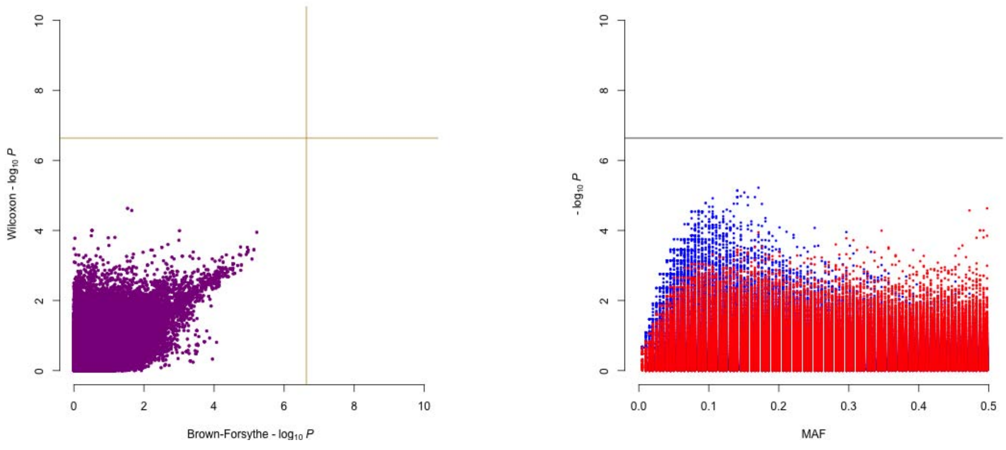

Supplementary Figure B83 - Summary of vGWA results for 283: Storage 56 days

Supplementary Table 1 - GWA (M) and vGWA (V) scores and ranks of trait 1: LD

Gene	Gene ID	Chr	Rank (M)	Score (M)	Test (M)	Rank (V)	Score (V)	Test (V)
ETC3	AT4G01060	4	2	11.7406	Wilcoxon	1208	6.8571	Brown-Forsythe
SVP	AT2G22540	2	3	11.298	Wilcoxon	5	17.96364	Brown-Forsythe
ATARP4	AT1G18450	1	5	10.4959	Wilcoxon	3606	4.93442	Brown-Forsythe
DFL2	AT4G03400	4	8	10.0205	Wilcoxon	8	16.88891	Brown-Forsythe
ATH1	AT4G32980	4	13	9.95211	Wilcoxon	1246	6.77968	Brown-Forsythe
AGL17	AT2G22630	2	15	9.84147	Wilcoxon	817	7.62161	Brown-Forsythe
PAT1	AT5G48150	5	30	9.35821	Wilcoxon	852	7.56549	Brown-Forsythe
DOG1	AT5G45830	5	47	8.90731	Wilcoxon	48	14.00288	Brown-Forsythe
sim-to-VRN1	AT4G33280	4	52	8.90358	Wilcoxon	215	10.50571	Brown-Forsythe
ATGA2OX7	AT1G50960	1	105	8.3998	Wilcoxon	619	8.23201	Brown-Forsythe
SVP	AT2G22540	2	1	8.57121	EMMA	5	17.96364	Brown-Forsythe
FLC	AT5G10140	5	2	6.24143	EMMA	1220	6.83578	Brown-Forsythe
AGL17	AT2G22630	2	4	6.12352	EMMA	817	7.62161	Brown-Forsythe
GI	AT1G22770	1	10	5.54344	EMMA	1999	5.90781	Brown-Forsythe
ETC3	AT4G01060	4	11	5.47408	EMMA	1208	6.8571	Brown-Forsythe
SPA4	AT1G53090	1	17	5.30428	EMMA	717	7.9423	Brown-Forsythe
SPL4	AT1G53160	1	17	5.30428	EMMA	717	7.9423	Brown-Forsythe
PAT1	AT5G48150	5	21	5.09294	EMMA	852	7.56549	Brown-Forsythe
AT2G30810	AT2G30810	2	24	4.88787	EMMA	752	7.83378	Brown-Forsythe
GA1	AT4G02780	4	37	4.6557	EMMA	635	8.18414	Brown-Forsythe

Supplementary Table 2 - GWA (M) and vGWA (V) scores and ranks of trait 2: LDV

Gene	Gene ID	Chr	Rank (M)	Score (M)	Test (M)	Rank (V)	Score (V)
No candidate gene suggested in Atwell et al. (2010), *Nature*.							

Supplementary Table 3 - GWA (M) and vGWA (V) scores and ranks of trait 3: SD

Gene	Gene ID	Chr	Rank (M)	Score (M)	Test (M)	Rank (V)	Score (V)	Test (V)
ATGA2OX7	AT1G50960	1	6	10.8001	Wilcoxon	249	7.36712	Brown-Forsythe
sim-to-VRN1	AT4G33280	4	9	10.7614	Wilcoxon	837	5.82012	Brown-Forsythe
ATH1	AT4G32980	4	17	10.1598	Wilcoxon	885	5.77272	Brown-Forsythe
DOG1	AT5G45830	5	23	9.38477	Wilcoxon	2	17.93296	Brown-Forsythe
RAV1	AT1G13260	1	44	8.96423	Wilcoxon	165	7.99892	Brown-Forsythe
DFL2	AT4G03400	4	53	8.84692	Wilcoxon	877	5.77889	Brown-Forsythe
PAT1	AT5G48150	5	108	8.15704	Wilcoxon	58	9.45668	Brown-Forsythe
ATARP4	AT1G18450	1	115	8.02712	Wilcoxon	608	6.21521	Brown-Forsythe
SPA2	AT4G11110	4	133	7.88967	Wilcoxon	1123	5.53711	Brown-Forsythe
DDF1	AT1G12610	1	138	7.83315	Wilcoxon	19	10.93332	Brown-Forsythe
ETC3	AT4G01060	4	3	5.7025	EMMA	310	7.10878	Brown-Forsythe
HEN2	AT2G06990	2	4	5.13541	EMMA	8	12.36783	Brown-Forsythe
ATGA2OX7	AT1G50960	1	5	5.12315	EMMA	249	7.36712	Brown-Forsythe
SVP	AT2G22540	2	17	4.6684	EMMA	52	9.68714	Brown-Forsythe
DOG1	AT5G45830	5	20	4.53821	EMMA	2	17.93296	Brown-Forsythe
PAT1	AT5G48150	5	30	4.25684	EMMA	58	9.45668	Brown-Forsythe
ATH1	AT4G32980	4	34	4.19098	EMMA	885	5.77272	Brown-Forsythe
SPA2	AT4G11110	4	51	4.06349	EMMA	1123	5.53711	Brown-Forsythe
sim-to-VRN1	AT4G33280	4	61	4.00216	EMMA	837	5.82012	Brown-Forsythe
LKP2	AT2G18915	2	84	3.85566	EMMA	1493	5.20564	Brown-Forsythe

Supplementary Table 4 - GWA (M) and vGWA (V) scores and ranks of trait 4: SDV

Gene	Gene ID	Chr	Rank (M)	Score (M)	Test (M)	Rank (V)	Score (V)	Test (V)
ATGA2OX7	AT1G50960	1	4	9.15154	Wilcoxon	187	5.24066	Brown-Forsythe
sim-to-VRN1	AT4G33280	4	17	8.32555	Wilcoxon	3676	2.65252	Brown-Forsythe
DOG1	AT5G45830	5	43	7.88579	Wilcoxon	40	6.52089	Brown-Forsythe
DFL2	AT4G03400	4	44	7.87713	Wilcoxon	111	5.72668	Brown-Forsythe
ATH1	AT4G32980	4	59	7.59222	Wilcoxon	1855	3.20412	Brown-Forsythe
FD	AT4G35900	4	93	7.21393	Wilcoxon	1326	3.46732	Brown-Forsythe
DDF1	AT1G12610	1	105	7.11653	Wilcoxon	2841	2.8674	Brown-Forsythe
HOS1	AT2G39810	2	107	7.10616	Wilcoxon	6690	2.1969	Brown-Forsythe
SPA2	AT4G11110	4	122	6.9728	Wilcoxon	3197	2.76528	Brown-Forsythe
RAV1	AT1G13260	1	149	6.81923	Wilcoxon	1429	3.39975	Brown-Forsythe
ICU2	AT5G67100	5	5	6.87858	EMMA	185	5.2498	Brown-Forsythe
AT3G10185	AT3G10185	3	84	4.21087	EMMA	1266	3.5054	Brown-Forsythe
AGL15	AT5G13790	5	102	4.0192	EMMA	1063	3.65279	Brown-Forsythe
AGL20	AT2G45660	2	117	3.91362	EMMA	1980	3.15135	Brown-Forsythe
AGL6	AT2G45650	2	117	3.91362	EMMA	1980	3.15135	Brown-Forsythe
ELF6	AT5G04240	5	120	3.89624	EMMA	1231	3.52576	Brown-Forsythe
ATGID1B/GID1B	AT3G63010	3	121	3.89149	EMMA	2166	3.08735	Brown-Forsythe
VIN3	AT5G57380	5	143	3.80016	EMMA	28	6.89848	Brown-Forsythe
ZTL	AT5G57360	5	143	3.80016	EMMA	28	6.89848	Brown-Forsythe
AGL19	AT4G22950	4	153	3.75152	EMMA	5344	2.36615	Brown-Forsythe

Supplementary Table 5 - GWA (M) and vGWA (V) scores and ranks of trait 5: FT10

Gene	Gene ID	Chr	Rank (M)	Score (M)	Test (M)	Rank (V)	Score (V)	Test (V)
ATH1	AT4G32980	4	1	11.3864	Wilcoxon	1143	5.03736	Brown-Forsythe
SVP	AT2G22540	2	2	11.3786	Wilcoxon	518	5.84446	Brown-Forsythe
AGL17	AT2G22630	2	5	10.8544	Wilcoxon	518	5.84446	Brown-Forsythe
sim-to-VRN1	AT4G33280	4	25	9.92236	Wilcoxon	428	6.07322	Brown-Forsythe
FES1	AT2G33835	2	36	9.7753	Wilcoxon	121	7.33393	Brown-Forsythe
SPL3	AT2G33810	2	36	9.7753	Wilcoxon	2430	4.29587	Brown-Forsythe
DOG1	AT5G45830	5	40	9.62578	Wilcoxon	49	8.46759	Brown-Forsythe
DFL2	AT4G03400	4	58	9.19259	Wilcoxon	142	7.16822	Brown-Forsythe
GASA5	AT3G02885	3	59	9.17769	Wilcoxon	138	7.18925	Brown-Forsythe
ATARP4	AT1G18450	1	76	8.92443	Wilcoxon	1577	4.72381	Brown-Forsythe
FLC	AT5G10140	5	4	5.83594	EMMA	623	5.64974	Brown-Forsythe
sim-to-ESD4	AT4G00690	4	21	5.14093	EMMA	140	7.17166	Brown-Forsythe
FRI	AT4G00650	4	21	5.14093	EMMA	348	6.29783	Brown-Forsythe
DOG1	AT5G45830	5	30	4.73222	EMMA	49	8.46759	Brown-Forsythe
FT	AT1G65480	1	32	4.65948	EMMA	3437	3.95529	Brown-Forsythe
BAS1	AT2G26710	2	52	4.10145	EMMA	25513	2.00598	Brown-Forsythe
NUA	AT1G79280	1	53	4.09725	EMMA	2793	4.15388	Brown-Forsythe
SVP	AT2G22540	2	61	3.98117	EMMA	518	5.84446	Brown-Forsythe
LD	AT4G02560	4	80	3.83659	EMMA	2570	4.24839	Brown-Forsythe
CRP	AT4G00450	4	135	3.42989	EMMA	167	6.99894	Brown-Forsythe

Supplementary Table 6 - GWA (M) and vGWA (V) scores and ranks of trait 6: FT16

Gene	Gene ID	Chr	Rank (M)	Score (M)	Test (M)	Rank (V)	Score (V)	Test (V)
ETC3	AT4G01060	4	2	11.69	Wilcoxon	378	11.1057	Brown-Forsythe
AGL17	AT2G22630	2	4	11.4261	Wilcoxon	3	20.57433	Brown-Forsythe
ATH1	AT4G32980	4	5	11.3772	Wilcoxon	1233	8.83944	Brown-Forsythe
ATARP4	AT1G18450	1	7	11.0602	Wilcoxon	111	13.92466	Brown-Forsythe
SVP	AT2G22540	2	8	11.0553	Wilcoxon	3	20.57433	Brown-Forsythe
sim-to-VRN1	AT4G33280	4	10	11.0289	Wilcoxon	944	9.36459	Brown-Forsythe
DFL2	AT4G03400	4	20	10.7701	Wilcoxon	129	13.57847	Brown-Forsythe
FPF1	AT5G24860	5	54	10.1493	Wilcoxon	368	11.20233	Brown-Forsythe
FLC	AT5G10140	5	71	10.0034	Wilcoxon	325	11.43774	Brown-Forsythe
GASA5	AT3G02885	3	147	9.16419	Wilcoxon	1435	8.51077	Brown-Forsythe
FLC	AT5G10140	5	1	6.76866	EMMA	325	11.43774	Brown-Forsythe
sim-to-EDS4	AT4G00690	4	6	5.15681	EMMA	450	10.73309	Brown-Forsythe
FRI	AT4G00650	4	6	5.15681	EMMA	2303	7.53779	Brown-Forsythe
DOG1	AT5G45830	5	20	4.5821	EMMA	1926	7.93548	Brown-Forsythe
FT	AT1G65480	1	54	4.33335	EMMA	4848	5.8215	Brown-Forsythe
CRP	AT4G00450	4	59	4.25287	EMMA	3106	6.83924	Brown-Forsythe
ATARP4	AT1G18450	1	68	4.205	EMMA	111	13.92466	Brown-Forsythe
LD	AT4G02560	4	82	4.02845	EMMA	827	9.60077	Brown-Forsythe
BAS1	AT2G26710	2	85	4.02267	EMMA	25167	2.63759	Brown-Forsythe
AGL17	AT2G22630	2	87	4.01148	EMMA	3	20.57433	Brown-Forsythe

Supplementary Table 7 - GWA (M) and vGWA (V) scores and ranks of trait 7: FT22

Gene	Gene ID	Chr	Rank (M)	Score (M)	Test (M)	Rank (V)	Score (V)	Test (V)
ATARP4	AT1G18450	1	4	11.6112	Wilcoxon	231	11.99001	Brown-Forsythe
DFL2	AT4G03400	4	16	10.824	Wilcoxon	197	12.31583	Brown-Forsythe
AGL17	AT2G22630	2	19	10.6444	Wilcoxon	10	19.636	Brown-Forsythe
SVP	AT2G22540	2	43	9.88639	Wilcoxon	10	19.636	Brown-Forsythe
ATH1	AT4G32980	4	52	9.74982	Wilcoxon	1295	8.06517	Brown-Forsythe
sim-to-VRN1	AT4G33280	4	97	9.14786	Wilcoxon	813	9.24459	Brown-Forsythe
ATGA2OX7	AT1G50960	1	103	9.14404	Wilcoxon	2395	6.71931	Brown-Forsythe
RAV1	AT1G13260	1	138	8.93617	Wilcoxon	705	9.46755	Brown-Forsythe
ETC3	AT4G01060	4	139	8.90838	Wilcoxon	809	9.253	Brown-Forsythe
FLC	AT5G10140	5	153	8.79855	Wilcoxon	98	14.04109	Brown-Forsythe
FLC	AT5G10140	5	1	7.35652	EMMA	98	14.04109	Brown-Forsythe
sim-to-ESD4	AT4G00690	4	21	6.02586	EMMA	967	8.7682	Brown-Forsythe
FRI	AT4G00650	4	21	6.02586	EMMA	2165	6.94445	Brown-Forsythe
DOG1	AT5G45830	5	39	4.95198	EMMA	651	9.64474	Brown-Forsythe
CDF1	AT5G62430	5	80	4.31728	EMMA	6141	4.85826	Brown-Forsythe
ATARP4	AT1G18450	1	98	4.18876	EMMA	231	11.99001	Brown-Forsythe
CRP	AT4G00450	4	180	3.62105	EMMA	3976	5.68902	Brown-Forsythe
SPA4	AT1G53090	1	188	3.58201	EMMA	749	9.39523	Brown-Forsythe
SPL4	AT1G53160	1	188	3.58201	EMMA	1515	7.69001	Brown-Forsythe
RGA1	AT2G01570	2	199	3.54707	EMMA	2248	6.85811	Brown-Forsythe

Supplementary Table 8 - GWA (M) and vGWA (V) scores and ranks of trait 8: Seed Dormancy

Gene	Gene ID	Chr	Rank (M)	Score (M)	Test (M)	Rank (V)	Score (V)	Test (V)
ATMYB65	AT3G11440	3	7	5.84953	Wilcoxon	1258	3.1394	Brown-Forsythe
ATCUL1	AT4G02570	4	12	5.65225	Wilcoxon	922	3.3557	Brown-Forsythe
LD	AT4G02560	4	12	5.65225	Wilcoxon	922	3.3557	Brown-Forsythe
CYCD4;1	AT5G65420	5	18	5.58598	Wilcoxon	1877	2.84571	Brown-Forsythe
AGL15	AT5G13790	5	19	5.54763	Wilcoxon	3047	2.50943	Brown-Forsythe
SPA4	AT1G53090	1	27	5.35615	Wilcoxon	1132	3.20495	Brown-Forsythe
PIL5	AT2G20180	2	34	5.2128	Wilcoxon	3483	2.42171	Brown-Forsythe
WER1	AT5G14750	5	73	4.92847	Wilcoxon	1128	3.2085	Brown-Forsythe
HBT	AT2G20000	2	99	4.79737	Wilcoxon	4090	2.32518	Brown-Forsythe
SDP1	AT5G04040	5	106	4.7611	Wilcoxon	693	3.57518	Brown-Forsythe
BME3	AT3G54810	3	3	5.58007	EMMA	498	3.80075	Brown-Forsythe
PSD	AT1G72560	1	19	5.16262	EMMA	3141	2.48768	Brown-Forsythe
FRS2	AT2G32250	2	23	5.02769	EMMA	3716	2.38276	Brown-Forsythe
PIL6	AT3G59060	3	29	4.68931	EMMA	327	4.15792	Brown-Forsythe
TT12	AT3G59030	3	29	4.68931	EMMA	327	4.15792	Brown-Forsythe
PIN8	AT5G15100	5	30	4.6591	EMMA	971	3.30759	Brown-Forsythe
HOS1	AT2G39810	2	35	4.4622	EMMA	322	4.1704	Brown-Forsythe
HUA2	AT5G23150	5	39	4.41261	EMMA	138	4.79807	Brown-Forsythe
SMZ	AT3G54990	3	54	4.23509	EMMA	248	4.33995	Brown-Forsythe
OLEO1	AT4G25140	4	56	4.21585	EMMA	1821	2.86409	Brown-Forsythe

Supplementary Table 9 - GWA (M) and vGWA (V) scores and ranks of trait 14: Li7

Gene	Gene ID	Chr	Rank (M)	Score (M)	Test (M)	Rank (V)	Score (V)
No candidate gene suggested in Atwell et al. (2010), *Nature*.							

Supplementary Table 10 - GWA (M) and vGWA (V) scores and ranks of trait 15: B11

Gene	Gene ID	Chr	Rank (M)	Score (M)	Test (M)	Rank (V)	Score (V)
No candidate gene suggested in Atwell et al. (2010), *Nature*.							

Supplementary Table 11 - GWA (M) and vGWA (V) scores and ranks of trait 16: Na23

Gene	Gene ID	Chr	Rank (M)	Score (M)	Test (M)	Rank (V)	Score (V)	Test (V)
HKT1	AT4G10310	4	1	6.24769	Wilcoxon	6	6.15284	Brown-Forsythe
HKT1	AT4G10310	4	1	8.3708	EMMA	6	6.15284	Brown-Forsythe

Supplementary Table 12 - GWA (M) and vGWA (V) scores and ranks of trait 17: Mg25

Gene	Gene ID	Chr	Rank (M)	Score (M)	Test (M)	Rank (V)	Score (V)
No candidate gene suggested in Atwell et al. (2010), *Nature*.							

Supplementary Table 13 - GWA (M) and vGWA (V) scores and ranks of trait 18: P31

Gene	Gene ID	Chr	Rank (M)	Score (M)	Test (M)	Rank (V)	Score (V)
No candidate gene suggested in Atwell et al. (2010), *Nature*.							

Supplementary Table 14 - GWA (M) and vGWA (V) scores and ranks of trait 19: S34

Gene	Gene ID	Chr	Rank (M)	Score (M)	Test (M)	Rank (V)	Score (V)	Test (V)
SULTR1;1	AT4G08620	4	44	3.69288	Wilcoxon	1569	1.95634	Brown-Forsythe
SULTR1;2	AT1G78000	1	140	3.17072	Wilcoxon	1107	2.0791	Brown-Forsythe
SULTR1;2	AT1G78000	1	157	3.33987	EMMA	1107	2.0791	Brown-Forsythe

Supplementary Table 15 - GWA (M) and vGWA (V) scores and ranks of trait 20: K39

Gene	Gene ID	Chr	Rank (M)	Score (M)	Test (M)	Rank (V)	Score (V)
No candidate gene suggested in Atwell et al. (2010), *Nature*.							

Supplementary Table 16 - GWA (M) and vGWA (V) scores and ranks of trait 21: Ca43

Gene	Gene ID	Chr	Rank (M)	Score (M)	Test (M)	Rank (V)	Score (V)
No candidate gene suggested in Atwell et al. (2010), *Nature*.							

Supplementary Table 17 - GWA (M) and vGWA (V) scores and ranks of trait 22: Mn55

Gene	Gene ID	Chr	Rank (M)	Score (M)	Test (M)	Rank (V)	Score (V)
No candidate gene suggested in Atwell et al. (2010), *Nature*.							

Supplementary Table 18 - GWA (M) and vGWA (V) scores and ranks of trait 23: Fe56

Gene	Gene ID	Chr	Rank (M)	Score (M)	Test (M)	Rank (V)	Score (V)
No candidate gene suggested in Atwell et al. (2010), *Nature*.							

Supplementary Table 19 - GWA (M) and vGWA (V) scores and ranks of trait 24: Co59

Gene	Gene ID	Chr	Rank (M)	Score (M)	Test (M)	Rank (V)	Score (V)
No candidate gene suggested in Atwell et al. (2010), *Nature*.							

Supplementary Table 20 - GWA (M) and vGWA (V) scores and ranks of trait 25: Ni60

Gene	Gene ID	Chr	Rank (M)	Score (M)	Test (M)	Rank (V)	Score (V)
No candidate gene suggested in Atwell et al. (2010), *Nature*.							

Supplementary Table 21 - GWA (M) and vGWA (V) scores and ranks of trait 26: Cu65

Gene	Gene ID	Chr	Rank (M)	Score (M)	Test (M)	Rank (V)	Score (V)
No candidate gene suggested in Atwell et al. (2010), *Nature*.							

Supplementary Table 22 - GWA (M) and vGWA (V) scores and ranks of trait 27: Zn66

Gene	Gene ID	Chr	Rank (M)	Score (M)	Test (M)	Rank (V)	Score (V)
No candidate gene suggested in Atwell et al. (2010), *Nature*.							

Supplementary Table 23 - GWA (M) and vGWA (V) scores and ranks of trait 28: As75

Gene	Gene ID	Chr	Rank (M)	Score (M)	Test (M)	Rank (V)	Score (V)
No candidate gene suggested in Atwell et al. (2010), *Nature*.							

Supplementary Table 24 - GWA (M) and vGWA (V) scores and ranks of trait 29: Se82

Gene	Gene ID	Chr	Rank (M)	Score (M)	Test (M)	Rank (V)	Score (V)	Test (V)
SULTR3;4	AT3G15990	3	74	3.6625	Wilcoxon	2665	1.74448	Brown-Forsythe
SULTR1;2	AT1G78000	1	154	3.15122	EMMA	1113	2.13141	Brown-Forsythe

Supplementary Table 25 - GWA (M) and vGWA (V) scores and ranks of trait 30: Mo98

Gene	Gene ID	Chr	Rank (M)	Score (M)	Test (M)	Rank (V)	Score (V)	Test (V)
MOT1	AT2G25680	2	31	4.3145	Wilcoxon	1	9.23419	Brown-Forsythe

Supplementary Table 26 - GWA (M) and vGWA (V) scores and ranks of trait 31: Cd114

Gene	Gene ID	Chr	Rank (M)	Score (M)	Test (M)	Rank (V)	Score (V)	Test (V)
HMA3	AT4G30120	4	13	4.86761	Wilcoxon	59	3.45119	Brown-Forsythe
HMA2	AT4G30110	4	17	4.77198	Wilcoxon	59	3.45119	Brown-Forsythe
HMA3	AT4G30120	4	131	3.29429	EMMA	59	3.45119	Brown-Forsythe
HMA2	AT4G30110	4	131	3.29429	EMMA	59	3.45119	Brown-Forsythe

Supplementary Table 27 - GWA (M) and vGWA (V) scores and ranks of trait 39: 0W

Gene	Gene ID	Chr	Rank (M)	Score (M)	Test (M)	Rank (V)	Score (V)	Test (V)
DOG1	AT5G45830	5	5	8.64406	Wilcoxon	20	9.37409	Brown-Forsythe
AGL17	AT2G22630	2	11	7.63685	Wilcoxon	618	5.18233	Brown-Forsythe
SVP	AT2G22540	2	11	7.63685	Wilcoxon	657	5.1129	Brown-Forsythe
ATARP4	AT1G18450	1	16	7.42352	Wilcoxon	753	4.98139	Brown-Forsythe
ETC3	AT4G01060	4	30	7.00318	Wilcoxon	732	5.00204	Brown-Forsythe
GA1	AT4G02780	4	34	6.85811	Wilcoxon	21	9.29584	Brown-Forsythe
ATGA2OX7	AT1G50960	1	46	6.6054	Wilcoxon	2199	3.97899	Brown-Forsythe
DFL2	AT4G03400	4	51	6.5377	Wilcoxon	16	9.92376	Brown-Forsythe
PAT1	AT5G48150	5	63	6.38646	Wilcoxon	1672	4.25584	Brown-Forsythe
RAV1	AT1G13260	1	91	6.13445	Wilcoxon	8	10.4829	Brown-Forsythe
DOG1	AT5G45830	5	5	5.51136	EMMA	20	9.37409	Brown-Forsythe
ETC3	AT4G01060	4	23	4.50434	EMMA	732	5.00204	Brown-Forsythe
GA1	AT4G02780	4	26	4.46126	EMMA	21	9.29584	Brown-Forsythe
ATARP4	AT1G18450	1	27	4.42836	EMMA	753	4.98139	Brown-Forsythe
SVP	AT2G22540	2	55	4.00266	EMMA	657	5.1129	Brown-Forsythe
LD	AT4G02560	4	57	3.9937	EMMA	3619	3.45828	Brown-Forsythe
CRP	AT4G00450	4	94	3.69007	EMMA	1283	4.49229	Brown-Forsythe
AGL17	AT2G22630	2	108	3.60569	EMMA	618	5.18233	Brown-Forsythe
sim-to-EDS4	AT4G00690	4	129	3.48307	EMMA	842	4.87748	Brown-Forsythe
FRI	AT4G00650	4	129	3.48307	EMMA	842	4.87748	Brown-Forsythe

Supplementary Table 28 - GWA (M) and vGWA (V) scores and ranks of trait 40: 2W

Gene	Gene ID	Chr	Rank (M)	Score (M)	Test (M)	Rank (V)	Score (V)	Test (V)
ETC3	AT4G01060	4	3	6.08452	Wilcoxon	224	10.09748	Brown-Forsythe
SVP	AT2G22540	2	8	5.55105	Wilcoxon	7	18.27943	Brown-Forsythe
ATARP4	AT1G18450	1	18	5.1009	Wilcoxon	1036	7.17769	Brown-Forsythe
FLC	AT5G10140	5	21	5.037	Wilcoxon	43	13.13255	Brown-Forsythe
ATGA2OX7	AT1G50960	1	37	4.66299	Wilcoxon	298	9.53877	Brown-Forsythe
PAT1	AT5G48150	5	46	4.49465	Wilcoxon	6313	4.08418	Brown-Forsythe
FHL	AT5G02200	5	52	4.34154	Wilcoxon	292	9.58921	Brown-Forsythe
DOG1	AT5G45830	5	57	4.26302	Wilcoxon	117	11.42124	Brown-Forsythe
sim-to-FCA	AT2G47310	2	85	4.01878	Wilcoxon	1455	6.53021	Brown-Forsythe
AGL17	AT2G22630	2	89	3.98623	Wilcoxon	7	18.27943	Brown-Forsythe
ETC3	AT4G01060	4	3	6.08452	EMMA	224	10.09748	Brown-Forsythe
SVP	AT2G22540	2	8	5.55105	EMMA	7	18.27943	Brown-Forsythe
ATARP4	AT1G18450	1	18	5.1009	EMMA	1036	7.17769	Brown-Forsythe
FLC	AT5G10140	5	21	5.037	EMMA	43	13.13255	Brown-Forsythe
ATGA2OX7	AT1G50960	1	37	4.66299	EMMA	298	9.53877	Brown-Forsythe
PAT1	AT5G48150	5	45	4.49465	EMMA	6313	4.08418	Brown-Forsythe
FHL	AT5G02200	5	50	4.34154	EMMA	292	9.58921	Brown-Forsythe
DOG1	AT5G45830	5	54	4.26302	EMMA	117	11.42124	Brown-Forsythe
AGL17	AT2G22630	2	83	3.98623	EMMA	7	18.27943	Brown-Forsythe
PIF4	AT2G43010	2	114	3.73038	EMMA	2390	5.6462	Brown-Forsythe

Supplementary Table 29 - GWA (M) and vGWA (V) scores and ranks of trait 41: 4W

Gene	Gene ID	Chr	Rank (M)	Score (M)	Test (M)	Rank (V)	Score (V)	Test (V)
DOG1	AT5G45830	5	18	8.10388	Wilcoxon	151	8.1055	Brown-Forsythe
ATH1	AT4G32980	4	21	7.99396	Wilcoxon	1174	5.12309	Brown-Forsythe
ATARP4	AT1G18450	1	33	7.89219	Wilcoxon	1723	4.64796	Brown-Forsythe
sim-to-VRN1	AT4G33280	4	52	7.59895	Wilcoxon	1009	5.3955	Brown-Forsythe
DFL2	AT4G03400	4	60	7.59393	Wilcoxon	39	9.90224	Brown-Forsythe
SVP	AT2G22540	2	98	7.20296	Wilcoxon	9	11.95759	Brown-Forsythe
RAV1	AT1G13260	1	100	7.19271	Wilcoxon	1016	5.38472	Brown-Forsythe
ATGA2OX7	AT1G50960	1	112	7.14885	Wilcoxon	1719	4.65136	Brown-Forsythe
ELF4	AT2G40080	2	136	7.04275	Wilcoxon	394	6.81857	Brown-Forsythe
PAT1	AT5G48150	5	139	7.00863	Wilcoxon	1876	4.55392	Brown-Forsythe
SVP	AT2G22540	2	1	7.83035	EMMA	9	11.95759	Brown-Forsythe
DOG1	AT5G45830	5	5	5.89822	EMMA	151	8.1055	Brown-Forsythe
PAT1	AT5G48150	5	8	5.66563	EMMA	1876	4.55392	Brown-Forsythe
ETC3	AT4G01060	4	12	5.1676	EMMA	11	11.72007	Brown-Forsythe
AGL17	AT2G22630	2	14	5.16114	EMMA	9	11.95759	Brown-Forsythe
PIL2	AT3G62090	3	40	4.47289	EMMA	309	7.20881	Brown-Forsythe
ATARP4	AT1G18450	1	41	4.47006	EMMA	1723	4.64796	Brown-Forsythe
VIN3	AT5G57380	5	42	4.46738	EMMA	4	13.46556	Brown-Forsythe
ZTL	AT5G57360	5	42	4.46738	EMMA	4	13.46556	Brown-Forsythe
DFL2	AT4G03400	4	82	3.9455	EMMA	39	9.90224	Brown-Forsythe

Supplementary Table 30 - GWA (M) and vGWA (V) scores and ranks of trait 42: 8W

Gene	Gene ID	Chr	Rank (M)	Score (M)	Test (M)	Rank (V)	Score (V)	Test (V)
RAV1	AT1G13260	1	1	10.3023	Wilcoxon	758	4.67703	Brown-Forsythe
ATH1	AT4G32980	4	7	9.20954	Wilcoxon	430	5.16467	Brown-Forsythe
ATGA2OX7	AT1G50960	1	10	9.09475	Wilcoxon	2315	3.73016	Brown-Forsythe
DOG1	AT5G45830	5	15	8.80655	Wilcoxon	242	5.61266	Brown-Forsythe
CUL4	AT5G46210	5	30	8.3178	Wilcoxon	3497	3.41771	Brown-Forsythe
sim-to-VRN1	AT4G33280	4	39	8.25028	Wilcoxon	4472	3.21927	Brown-Forsythe
HEN2	AT2G06990	2	48	8.18372	Wilcoxon	54	6.47746	Brown-Forsythe
GASA5	AT3G02885	3	67	7.95567	Wilcoxon	13	7.50691	Brown-Forsythe
sim-to-FCA	AT2G47310	2	84	7.66337	Wilcoxon	3638	3.38856	Brown-Forsythe
FLC	AT5G10140	5	107	7.55465	Wilcoxon	61	6.38903	Brown-Forsythe
FLC	AT5G10140	5	21	4.46966	EMMA	61	6.38903	Brown-Forsythe
ATH1	AT4G32980	4	27	4.30108	EMMA	430	5.16467	Brown-Forsythe
TFL2	AT5G17690	5	29	4.28395	EMMA	158	5.91751	Brown-Forsythe
CO	AT5G15840	5	31	4.19141	EMMA	160	5.91409	Brown-Forsythe
COL1	AT5G15850	5	31	4.19141	EMMA	43	6.732	Brown-Forsythe
PHYB	AT2G18790	2	42	3.94036	EMMA	505	5.04122	Brown-Forsythe
HEN2	AT2G06990	2	57	3.81037	EMMA	54	6.47746	Brown-Forsythe
RAV1	AT1G13260	1	58	3.80001	EMMA	758	4.67703	Brown-Forsythe
sim-to-FCA	AT2G47310	2	61	3.77858	EMMA	3638	3.38856	Brown-Forsythe
sim-to-VRN1	AT4G33280	4	130	3.43423	EMMA	4472	3.21927	Brown-Forsythe

Supplementary Table 31 - GWA (M) and vGWA (V) scores and ranks of trait 43: FLC

Gene	Gene ID	Chr	Rank (M)	Score (M)	Test (M)	Rank (V)	Score (V)	Test (V)
SVP	AT2G22540	2	3	8.9246	Wilcoxon	3281	2.59967	Brown-Forsythe
AGL17	AT2G22630	2	5	8.59608	Wilcoxon	3281	2.59967	Brown-Forsythe
SPA4	AT1G53090	1	7	8.49959	Wilcoxon	33	5.17117	Brown-Forsythe
SPL4	AT1G53160	1	7	8.49959	Wilcoxon	33	5.17117	Brown-Forsythe
ETC3	AT4G01060	4	12	8.18367	Wilcoxon	983	3.27492	Brown-Forsythe
DFL2	AT4G03400	4	17	7.96135	Wilcoxon	541	3.60023	Brown-Forsythe
DOG1	AT5G45830	5	20	7.81214	Wilcoxon	11700	1.83836	Brown-Forsythe
CRP	AT4G00450	4	25	7.74903	Wilcoxon	152	4.28631	Brown-Forsythe
EMF1	AT5G11530	5	40	7.37903	Wilcoxon	446	3.71607	Brown-Forsythe
sim-to-VRN1	AT4G33280	4	47	7.24378	Wilcoxon	2524	2.74382	Brown-Forsythe
ETC3	AT4G01060	4	16	4.84345	EMMA	983	3.27492	Brown-Forsythe
SPA4	AT1G53090	1	35	4.56274	EMMA	33	5.17117	Brown-Forsythe
SPL4	AT1G53160	1	35	4.56274	EMMA	33	5.17117	Brown-Forsythe
CRP	AT4G00450	4	39	4.49707	EMMA	152	4.28631	Brown-Forsythe
DFL2	AT4G03400	4	41	4.42654	EMMA	541	3.60023	Brown-Forsythe
sim-to-EDS4	AT4G00690	4	71	3.90626	EMMA	249	4.01486	Brown-Forsythe
FRI	AT4G00650	4	71	3.90626	EMMA	391	3.79432	Brown-Forsythe
SVP	AT2G22540	2	72	3.9053	EMMA	3281	2.59967	Brown-Forsythe
RGA1	AT2G01570	2	101	3.62535	EMMA	3297	2.59765	Brown-Forsythe
DOG1	AT5G45830	5	113	3.56265	EMMA	11700	1.83836	Brown-Forsythe

Supplementary Table 32 - GWA (M) and vGWA (V) scores and ranks of trait 44: FRI

Gene	Gene ID	Chr	Rank (M)	Score (M)	Test (M)	Rank (V)	Score (V)	Test (V)
FRI	AT4G00650	4	1	14.291	Wilcoxon	475	3.96965	Brown-Forsythe
sim-to-ESD4	AT4G00690	4	1	14.291	Wilcoxon	475	3.96965	Brown-Forsythe
CRP	AT4G00450	4	11	8.32062	Wilcoxon	1646	3.13048	Brown-Forsythe
ETC3	AT4G01060	4	36	6.04476	Wilcoxon	687	3.69518	Brown-Forsythe
YAP169	AT5G07200	5	93	4.9258	Wilcoxon	2183	2.94368	Brown-Forsythe
ESD4	AT4G15880	4	114	4.75766	Wilcoxon	484	3.95061	Brown-Forsythe
SPA1	AT2G46340	2	120	4.70695	Wilcoxon	4447	2.4814	Brown-Forsythe
CUL4	AT5G46210	5	136	4.64302	Wilcoxon	10146	1.94423	Brown-Forsythe
SKB1	AT4G31120	4	189	4.42435	Wilcoxon	129	5.10225	Brown-Forsythe
ATARP4	AT1G18450	1	201	4.3737	Wilcoxon	5151	2.38273	Brown-Forsythe
FRI	AT4G00650	4	1	23.4027	EMMA	475	3.96965	Brown-Forsythe
sim-to-ESD4	AT4G00690	4	1	23.4027	EMMA	475	3.96965	Brown-Forsythe
CRP	AT4G00450	4	11	11.1729	EMMA	1646	3.13048	Brown-Forsythe
ETC3	AT4G01060	4	38	6.28005	EMMA	687	3.69518	Brown-Forsythe
SKB1	AT4G31120	4	88	4.291	EMMA	129	5.10225	Brown-Forsythe
CUL4	AT5G46210	5	99	4.15739	EMMA	10146	1.94423	Brown-Forsythe
AGL18	AT3G57390	3	141	3.76624	EMMA	15010	1.69333	Brown-Forsythe
ATGID1B	AT3G63010	3	170	3.52783	EMMA	1676	3.11698	Brown-Forsythe
sim-to-REF6	AT5G46910	5	233	3.27626	EMMA	550	3.84155	Brown-Forsythe
AGL8	AT5G60910	5	234	3.27625	EMMA	7079	2.17991	Brown-Forsythe

Supplementary Table 33 - GWA (M) and vGWA (V) scores and ranks of trait 45: 8W GH FT

Gene	Gene ID	Chr	Rank (M)	Score (M)	Test (M)	Rank (V)	Score (V)	Test (V)
DOG1	AT5G45830	5	13	9.45204	Wilcoxon	38	6.43965	Brown-Forsythe
sim-to-VRN1	AT4G33280	4	27	8.78487	Wilcoxon	12128	1.97819	Brown-Forsythe
HEN2	AT2G06990	2	48	8.41001	Wilcoxon	958	3.84241	Brown-Forsythe
ATGA2OX7	AT1G50960	1	58	8.23991	Wilcoxon	1878	3.32826	Brown-Forsythe
GASA5	AT3G02885	3	60	8.21011	Wilcoxon	30	6.79464	Brown-Forsythe
ATH1	AT4G32980	4	63	8.08653	Wilcoxon	1881	3.32809	Brown-Forsythe
RAV1	AT1G13260	1	87	7.84994	Wilcoxon	2918	3.00696	Brown-Forsythe
sim-to-FCA	AT2G47310	2	100	7.55871	Wilcoxon	4902	2.62122	Brown-Forsythe
SHP2	AT2G42830	2	158	7.09915	Wilcoxon	4060	2.75258	Brown-Forsythe
AGL17	AT2G22630	2	216	6.78661	Wilcoxon	504	4.42976	Brown-Forsythe
DOG1	AT5G45830	5	3	5.68175	EMMA	38	6.43965	Brown-Forsythe
FLC	AT5G10140	5	10	4.92142	EMMA	17	7.24092	Brown-Forsythe
ELF8	AT2G06210	2	37	4.30737	EMMA	653	4.17646	Brown-Forsythe
LDL1	AT1G62830	1	62	3.96084	EMMA	3843	2.79563	Brown-Forsythe
PAT1	AT5G48150	5	71	3.89735	EMMA	722	4.08351	Brown-Forsythe
SVP	AT2G22540	2	92	3.83531	EMMA	504	4.42976	Brown-Forsythe
GASA5	AT3G02885	3	95	3.82346	EMMA	30	6.79464	Brown-Forsythe
VIP5	AT1G61040	1	124	3.61616	EMMA	1025	3.77736	Brown-Forsythe
FES1	AT2G33835	2	133	3.58601	EMMA	24289	1.50854	Brown-Forsythe
SPL3	AT2G33810	2	133	3.58601	EMMA	24289	1.50854	Brown-Forsythe

Supplementary Table 34 - GWA (M) and vGWA (V) scores and ranks of trait 46: 8W GH LN

Gene	Gene ID	Chr	Rank (M)	Score (M)	Test (M)	Rank (V)	Score (V)	Test (V)
DOG1	AT5G45830	5	2	9.12376	Wilcoxon	61	7.03691	Brown-Forsythe
ATGA2OX7	AT1G50960	1	6	8.80264	Wilcoxon	6462	2.56899	Brown-Forsythe
CRY1	AT4G08920	4	7	8.75653	Wilcoxon	570	4.72558	Brown-Forsythe
ATH1	AT4G32980	4	8	8.72322	Wilcoxon	1809	3.68375	Brown-Forsythe
sim-to-VRN1	AT4G33280	4	12	8.65723	Wilcoxon	5350	2.72149	Brown-Forsythe
HEN2	AT2G06990	2	22	8.33749	Wilcoxon	81	6.75967	Brown-Forsythe
PHYB	AT2G18790	2	65	7.19898	Wilcoxon	402	5.04094	Brown-Forsythe
ETC3	AT4G01060	4	78	7.04572	Wilcoxon	881	4.36149	Brown-Forsythe
CO	AT5G15840	5	87	7.02188	Wilcoxon	24	7.94591	Brown-Forsythe
COL1	AT5G15850	5	87	7.02188	Wilcoxon	24	7.94591	Brown-Forsythe
FRI	AT4G00650	4	2	6.8722	EMMA	15114	1.89774	Brown-Forsythe
sim-to-ESD4	AT4G00690	4	2	6.8722	EMMA	14403	1.93338	Brown-Forsythe
AGL15	AT5G13790	5	7	5.55414	EMMA	6596	2.55154	Brown-Forsythe
DOG1	AT5G45830	5	9	5.02585	EMMA	61	7.03691	Brown-Forsythe
HEN2	AT2G06990	2	13	4.75819	EMMA	81	6.75967	Brown-Forsythe
CRY1	AT4G08920	4	31	4.37153	EMMA	570	4.72558	Brown-Forsythe
ATH1	AT4G32980	4	32	4.35096	EMMA	1809	3.68375	Brown-Forsythe
CO	AT5G15840	5	36	4.19578	EMMA	24	7.94591	Brown-Forsythe
COL1	AT5G15850	5	36	4.19578	EMMA	24	7.94591	Brown-Forsythe
FRL1	AT5G16320	5	39	4.13468	EMMA	41	7.38796	Brown-Forsythe

Supplementary Table 35 - GWA (M) and vGWA (V) scores and ranks of trait 47: 0W GH FT

Gene	Gene ID	Chr	Rank (M)	Score (M)	Test (M)	Rank (V)	Score (V)	Test (V)
ETC3	AT4G01060	4	6	9.51193	Wilcoxon	1058	7.52981	Brown-Forsythe
AGL17	AT2G22630	2	13	9.25378	Wilcoxon	1	23.22219	Brown-Forsythe
SVP	AT2G22540	2	13	9.25378	Wilcoxon	1	23.22219	Brown-Forsythe
DFL2	AT4G03400	4	17	9.24797	Wilcoxon	109	12.3346	Brown-Forsythe
ATARP4	AT1G18450	1	30	8.85728	Wilcoxon	2833	5.67523	Brown-Forsythe
RAV1	AT1G13260	1	48	8.58298	Wilcoxon	152	11.55033	Brown-Forsythe
ATH1	AT4G32980	4	77	8.27901	Wilcoxon	2536	5.87017	Brown-Forsythe
FRI	AT4G00650	4	86	8.21846	Wilcoxon	459	9.38768	Brown-Forsythe
GASA5	AT3G02885	3	110	8.07613	Wilcoxon	433	9.4811	Brown-Forsythe
PAT1	AT5G48150	5	123	7.96829	Wilcoxon	1129	7.41232	Brown-Forsythe
SVP	AT2G22540	2	10	5.81731	EMMA	1	23.22219	Brown-Forsythe
AGL17	AT2G22630	2	10	5.81731	EMMA	1	23.22219	Brown-Forsythe
FRI	AT4G00650	4	24	5.26745	EMMA	459	9.38768	Brown-Forsythe
sim-to-ESD4	AT4G00690	4	24	5.26745	EMMA	459	9.38768	Brown-Forsythe
AT2G30810	AT2G30810	2	35	4.98061	EMMA	723	8.30233	Brown-Forsythe
SPA4	AT1G53090	1	39	4.85632	EMMA	218	10.77637	Brown-Forsythe
SPL4	AT1G53160	1	39	4.85632	EMMA	218	10.77637	Brown-Forsythe
SMZ	AT3G54990	3	49	4.68024	EMMA	4156	5.00853	Brown-Forsythe
ETC3	AT4G01060	4	54	4.56776	EMMA	1058	7.52981	Brown-Forsythe
ATARP4	AT1G18450	1	60	4.48035	EMMA	2833	5.67523	Brown-Forsythe

Supplementary Table 36 - GWA (M) and vGWA (V) scores and ranks of trait 48: 0W GH LN

Gene	Gene ID	Chr	Rank (M)	Score (M)	Test (M)	Rank (V)	Score (V)	Test (V)
ETC3	AT4G01060	4	1	7.21016	Wilcoxon	96	6.34154	Brown-Forsythe
DOG1	AT5G45830	5	5	6.74401	Wilcoxon	5	10.22745	Brown-Forsythe
FRI	AT4G00650	4	47	5.27161	Wilcoxon	2226	3.37527	Brown-Forsythe
sim-to-ESD4	AT4G00690	4	47	5.27161	Wilcoxon	2226	3.37527	Brown-Forsythe
CO	AT5G15840	5	61	5.13955	Wilcoxon	3262	3.07667	Brown-Forsythe
COL1	AT5G15850	5	61	5.13955	Wilcoxon	3262	3.07667	Brown-Forsythe
ATH1	AT4G32980	4	100	4.74291	Wilcoxon	850	4.10764	Brown-Forsythe
VIM1	AT1G57820	1	106	4.70271	Wilcoxon	1907	3.49043	Brown-Forsythe
sim-to-VRN1	AT4G33280	4	198	4.3151	Wilcoxon	1002	3.98791	Brown-Forsythe
FHL	AT5G02200	5	207	4.30446	Wilcoxon	2584	3.25611	Brown-Forsythe
FRI	AT4G00650	4	8	4.40313	EMMA	2226	3.37527	Brown-Forsythe
sim-to-ESD4	AT4G00690	4	8	4.40313	EMMA	2226	3.37527	Brown-Forsythe
FCA	AT4G16280	4	107	3.29776	EMMA	970	4.00956	Brown-Forsythe
PHYD	AT4G16250	4	107	3.29776	EMMA	970	4.00956	Brown-Forsythe
ETC3	AT4G01060	4	119	3.24721	EMMA	96	6.34154	Brown-Forsythe
AGL24	AT4G24540	4	151	3.1231	EMMA	10427	2.12234	Brown-Forsythe
AGL14	AT4G11880	4	156	3.09849	EMMA	50	7.00588	Brown-Forsythe
SEPALLATA2	AT3G02310	3	189	3.00315	EMMA	9267	2.22001	Brown-Forsythe
COL2	AT3G02380	3	189	3.00315	EMMA	9267	2.22001	Brown-Forsythe
FD	AT4G35900	4	204	2.97328	EMMA	6782	2.46747	Brown-Forsythe

Supplementary Table 37 - GWA (M) and vGWA (V) scores and ranks of trait 57: FT Field

Gene	Gene ID	Chr	Rank (M)	Score (M)	Test (M)	Rank (V)	Score (V)	Test (V)
ATH1	AT4G32980	4	19	10.1569	Wilcoxon	5710	2.18997	Brown-Forsythe
AGL16	AT3G57230	3	21	10.0736	Wilcoxon	1999	2.86774	Brown-Forsythe
sim-to-VRN1	AT4G33280	4	29	9.76096	Wilcoxon	4600	2.32342	Brown-Forsythe
DOG1	AT5G45830	5	35	9.5235	Wilcoxon	610	3.73985	Brown-Forsythe
AGL18	AT3G57390	3	39	9.24223	Wilcoxon	947	3.39753	Brown-Forsythe
ETC3	AT4G01060	4	68	8.73049	Wilcoxon	5185	2.25158	Brown-Forsythe
MMP	AT1G70170	1	90	8.33638	Wilcoxon	12733	1.72854	Brown-Forsythe
LKP2	AT2G18915	2	94	8.25415	Wilcoxon	6968	2.07529	Brown-Forsythe
FY	AT5G13480	5	100	8.16873	Wilcoxon	17701	1.54699	Brown-Forsythe
ATGA2OX7	AT1G50960	1	117	7.96492	Wilcoxon	7885	2.0019	Brown-Forsythe
TSF	AT4G20370	4	3	5.7133	EMMA	564	3.79348	Brown-Forsythe
APRR5	AT5G24470	5	8	4.99778	EMMA	3426	2.50235	Brown-Forsythe
ATHAP2B	AT3G05690	3	12	4.57815	EMMA	11	8.76338	Brown-Forsythe
HEN2	AT2G06990	2	17	4.41825	EMMA	1886	2.91227	Brown-Forsythe
DOG1	AT5G45830	5	31	4.10872	EMMA	610	3.73985	Brown-Forsythe
ELF5	AT5G62640	5	33	4.04498	EMMA	4820	2.29623	Brown-Forsythe
LD	AT4G02560	4	50	3.92096	EMMA	4751	2.30544	Brown-Forsythe
GI	AT1G22770	1	100	3.485	EMMA	3579	2.47414	Brown-Forsythe
DDF2	AT1G63030	1	102	3.47798	EMMA	7770	2.0099	Brown-Forsythe
sim-to-VRN1	AT4G33280	4	121	3.41989	EMMA	4600	2.32342	Brown-Forsythe

Supplementary Table 38 - GWA (M) and vGWA (V) scores and ranks of trait 58: FT Diameter Field

Gene	Gene ID	Chr	Rank (M)	Score (M)	Test (M)	Rank (V)	Score (V)	Test (V)
SVP	AT2G22540	2	1	9.74548	Wilcoxon	826	2.41492	Brown-Forsythe
ICU2	AT5G67100	5	3	9.4309	Wilcoxon	1008	2.32701	Brown-Forsythe
FHL	AT5G02200	5	82	7.30149	Wilcoxon	320	2.82008	Brown-Forsythe
FPF1	AT5G24860	5	143	6.80907	Wilcoxon	1298	2.22148	Brown-Forsythe
GA4H	AT1G80340	1	187	6.5702	Wilcoxon	593	2.57027	Brown-Forsythe
DDF1	AT1G12610	1	237	6.40908	Wilcoxon	1687	2.11484	Brown-Forsythe
ELF5	AT5G62640	5	253	6.35884	Wilcoxon	19213	1.0854	Brown-Forsythe
FES1	AT2G33835	2	257	6.35111	Wilcoxon	7466	1.48621	Brown-Forsythe
SPL3	AT2G33810	2	257	6.35111	Wilcoxon	14189	1.21289	Brown-Forsythe
FWA	AT4G25530	4	316	6.11017	Wilcoxon	17150	1.13242	Brown-Forsythe
ETC3	AT4G01060	4	16	8.70176	EMMA	5719	1.59997	Brown-Forsythe
DOG1	AT5G45830	5	17	8.53689	EMMA	692	2.49891	Brown-Forsythe
ATARP4	AT1G18450	1	31	7.98428	EMMA	5157	1.63877	Brown-Forsythe
CO	AT5G15840	5	40	7.789	EMMA	7481	1.48494	Brown-Forsythe
COL1	AT5G15850	5	40	7.789	EMMA	7481	1.48494	Brown-Forsythe
SVP	AT2G22540	2	45	7.72477	EMMA	826	2.41492	Brown-Forsythe
ATH1	AT4G32980	4	46	7.68272	EMMA	1099	2.29132	Brown-Forsythe
FRI	AT4G00650	4	47	7.64895	EMMA	2895	1.88172	Brown-Forsythe
AGL17	AT2G22630	2	54	7.52425	EMMA	3272	1.83549	Brown-Forsythe
YAP169	AT5G07200	5	58	7.47533	EMMA	665	2.51972	Brown-Forsythe

Supplementary Table 39 - GWA (M) and vGWA (V) scores and ranks of trait 59: FT GH

Gene	Gene ID	Chr	Rank (M)	Score (M)	Test (M)	Rank (V)	Score (V)	Test (V)
ICU2	AT5G67100	5	5	4.99591	Wilcoxon	929	7.06528	Brown-Forsythe
SVP	AT2G22540	2	19	4.21499	Wilcoxon	1	27.22107	Brown-Forsythe
FHL	AT5G02200	5	33	3.95209	Wilcoxon	4749	4.46467	Brown-Forsythe
DDF2	AT1G63030	1	59	3.6236	Wilcoxon	10291	3.28968	Brown-Forsythe
FLP1	AT4G31380	4	143	3.26417	Wilcoxon	2585	5.42501	Brown-Forsythe
SAR3	AT1G80680	1	215	3.07062	Wilcoxon	2320	5.61479	Brown-Forsythe
PHYD	AT4G16250	4	245	3.01107	Wilcoxon	750	7.43897	Brown-Forsythe
LIP1	AT5G64813	5	278	2.96784	Wilcoxon	16410	2.62288	Brown-Forsythe
DDF1	AT1G12610	1	304	2.91061	Wilcoxon	1196	6.67191	Brown-Forsythe
VIM1	AT1G57820	1	321	2.88968	Wilcoxon	838	7.24963	Brown-Forsythe
ATARP4	AT1G18450	1	1	7.02089	EMMA	41	12.59875	Brown-Forsythe
AGL17	AT2G22630	2	15	5.14164	EMMA	1	27.22107	Brown-Forsythe
SVP	AT2G22540	2	17	5.1299	EMMA	1	27.22107	Brown-Forsythe
YAP169	AT5G07200	5	19	5.11259	EMMA	37	13.21657	Brown-Forsythe
ETC3	AT4G01060	4	30	4.73865	EMMA	260	9.17287	Brown-Forsythe
DOG1	AT5G45830	5	31	4.7336	EMMA	199	9.75233	Brown-Forsythe
FRI	AT4G00650	4	36	4.47972	EMMA	1113	6.78325	Brown-Forsythe
DFL2	AT4G03400	4	37	4.47586	EMMA	355	8.6994	Brown-Forsythe
PAT1	AT5G48150	5	58	3.9579	EMMA	995	6.961	Brown-Forsythe
SPA4	AT1G53090	1	66	3.9173	EMMA	242	9.32867	Brown-Forsythe

Supplementary Table 40 - GWA (M) and vGWA (V) scores and ranks of trait 60: FT Duration GH

Gene	Gene ID	Chr	Rank (M)	Score (M)	Test (M)	Rank (V)	Score (V)	Test (V)
GASA4	AT5G15230	5	3	6.06219	Wilcoxon	273	4.30491	Brown-Forsythe
ICU2	AT5G67100	5	12	5.36677	Wilcoxon	323	4.2072	Brown-Forsythe
ELF6	AT5G04240	5	23	5.07589	Wilcoxon	3168	2.68453	Brown-Forsythe
EMS1	AT5G07280	5	40	4.82342	Wilcoxon	911	3.52167	Brown-Forsythe
TFL1	AT5G03840	5	44	4.73743	Wilcoxon	260	4.33155	Brown-Forsythe
ATHB51	AT5G03790	5	99	4.4044	Wilcoxon	260	4.33155	Brown-Forsythe
CO	AT5G15840	5	116	4.31182	Wilcoxon	547	3.83505	Brown-Forsythe
COL1	AT5G15850	5	116	4.31182	Wilcoxon	547	3.83505	Brown-Forsythe
ULT1	AT4G28190	4	170	4.17324	Wilcoxon	2231	2.90888	Brown-Forsythe
AT2G39540	AT2G39540	2	175	4.14769	Wilcoxon	469	3.94993	Brown-Forsythe
GASA4	AT5G15230	5	2	7.72389	EMMA	273	4.30491	Brown-Forsythe
TFL1	AT5G03840	5	30	4.89038	EMMA	260	4.33155	Brown-Forsythe
ICU2	AT5G67100	5	31	4.85869	EMMA	323	4.2072	Brown-Forsythe
ELF6	AT5G04240	5	37	4.63856	EMMA	3168	2.68453	Brown-Forsythe
EMS1	AT5G07280	5	48	4.34813	EMMA	911	3.52167	Brown-Forsythe
CO	AT5G15840	5	65	4.05765	EMMA	547	3.83505	Brown-Forsythe
COL1	AT5G15850	5	65	4.05765	EMMA	547	3.83505	Brown-Forsythe
GCR1	AT1G48270	1	71	4.00853	EMMA	3251	2.66861	Brown-Forsythe
HUA2	AT5G23150	5	83	3.89486	EMMA	7170	2.14691	Brown-Forsythe
ETC3	AT4G01060	4	125	3.67675	EMMA	317	4.21319	Brown-Forsythe

Supplementary Table 41 - GWA (M) and vGWA (V) scores and ranks of trait 61: LC Duration GH

Gene	Gene ID	Chr	Rank (M)	Score (M)	Test (M)	Rank (V)	Score (V)	Test (V)
ICU2	AT5G67100	5	10	6.60842	Wilcoxon	252	5.1213	Brown-Forsythe
ETC3	AT4G01060	4	37	5.91516	Wilcoxon	816	4.16175	Brown-Forsythe
AGL18	AT3G57390	3	48	5.70703	Wilcoxon	536	4.51159	Brown-Forsythe
SRR1	AT5G59560	5	61	5.41654	Wilcoxon	124	5.71056	Brown-Forsythe
GASA4	AT5G15230	5	90	5.18667	Wilcoxon	3084	2.95986	Brown-Forsythe
EZA1	AT4G02020	4	98	5.11184	Wilcoxon	7	7.51537	Brown-Forsythe
FAR1	AT4G15090	4	100	5.10149	Wilcoxon	2142	3.25091	Brown-Forsythe
ATGA2OX7	AT1G50960	1	107	5.0705	Wilcoxon	3435	2.87576	Brown-Forsythe
AGL14	AT4G11880	4	124	5.003	Wilcoxon	328	4.89805	Brown-Forsythe
APRR3	AT5G60100	5	133	4.94209	Wilcoxon	6489	2.40776	Brown-Forsythe
AGL14	AT4G11880	4	4	5.5404	EMMA	328	4.89805	Brown-Forsythe
ETC3	AT4G01060	4	12	4.77407	EMMA	816	4.16175	Brown-Forsythe
sim-to-VRN1	AT4G33280	4	40	4.00147	EMMA	397	4.77173	Brown-Forsythe
FAR1	AT4G15090	4	83	3.56067	EMMA	2142	3.25091	Brown-Forsythe
FT	AT1G65480	1	132	3.31958	EMMA	1800	3.39503	Brown-Forsythe
AGL18	AT3G57390	3	158	3.23721	EMMA	536	4.51159	Brown-Forsythe
CUL4	AT5G46210	5	171	3.19609	EMMA	1333	3.68188	Brown-Forsythe
AT5G59570	AT5G59570	5	174	3.19111	EMMA	124	5.71056	Brown-Forsythe
SRR1	AT5G59560	5	174	3.19111	EMMA	124	5.71056	Brown-Forsythe
SKB1	AT4G31120	4	188	3.16607	EMMA	4354	2.68994	Brown-Forsythe

Supplementary Table 42 - GWA (M) and vGWA (V) scores and ranks of trait 62: LFS GH

Gene	Gene ID	Chr	Rank (M)	Score (M)	Test (M)	Rank (V)	Score (V)	Test (V)
ICU2	AT5G67100	5	7	7.04614	Wilcoxon	258	5.33472	Brown-Forsythe
ETC3	AT4G01060	4	41	5.85085	Wilcoxon	596	4.64015	Brown-Forsythe
GASA4	AT5G15230	5	57	5.63531	Wilcoxon	3487	2.92405	Brown-Forsythe
ATGA2OX7	AT1G50960	1	74	5.42484	Wilcoxon	890	4.21286	Brown-Forsythe
SRR1	AT5G59560	5	92	5.31907	Wilcoxon	298	5.2176	Brown-Forsythe
EZA1	AT4G02020	4	100	5.23133	Wilcoxon	9	8.01982	Brown-Forsythe
FAR1	AT4G15090	4	106	5.19726	Wilcoxon	3600	2.90005	Brown-Forsythe
AT5G59570	AT5G59570	5	125	5.0691	Wilcoxon	298	5.2176	Brown-Forsythe
AGL18	AT3G57390	3	126	5.06899	Wilcoxon	1630	3.61929	Brown-Forsythe
APRR3	AT5G60100	5	133	5.03744	Wilcoxon	4877	2.6529	Brown-Forsythe
AGL15	AT5G13790	5	20	4.33222	EMMA	3390	2.94533	Brown-Forsythe
ETC3	AT4G01060	4	30	4.11271	EMMA	596	4.64015	Brown-Forsythe
sim-to-VRN1	AT4G33280	4	32	4.10289	EMMA	355	5.11159	Brown-Forsythe
AGL14	AT4G11880	4	36	3.99126	EMMA	1087	4.01876	Brown-Forsythe
FT	AT1G65480	1	48	3.88101	EMMA	1252	3.8963	Brown-Forsythe
SKB1	AT4G31120	4	57	3.83013	EMMA	4099	2.78869	Brown-Forsythe
FPF1	AT5G24860	5	70	3.67151	EMMA	1910	3.46686	Brown-Forsythe
YAP169	AT5G07200	5	149	3.27233	EMMA	2651	3.1734	Brown-Forsythe
FRI	AT4G00650	4	155	3.25513	EMMA	848	4.26303	Brown-Forsythe
PHYB	AT2G18790	2	169	3.216	EMMA	4546	2.70421	Brown-Forsythe

Supplementary Table 43 - GWA (M) and vGWA (V) scores and ranks of trait 63: MT GH

Gene	Gene ID	Chr	Rank (M)	Score (M)	Test (M)	Rank (V)	Score (V)	Test (V)
INO	AT1G23420	1	26	5.52847	Wilcoxon	592	4.04437	Brown-Forsythe
ATHB-2	AT4G16780	4	35	5.37416	Wilcoxon	547	4.09856	Brown-Forsythe
MBD9	AT3G01460	3	64	5.04365	Wilcoxon	10262	2.08803	Brown-Forsythe
VIM1	AT1G57820	1	95	4.74992	Wilcoxon	1668	3.34231	Brown-Forsythe
FRI	AT4G00650	4	108	4.66187	Wilcoxon	4196	2.72816	Brown-Forsythe
PHYB	AT2G18790	2	129	4.5873	Wilcoxon	1224	3.55423	Brown-Forsythe
CDF1	AT5G62430	5	138	4.56465	Wilcoxon	2935	2.96632	Brown-Forsythe
ELIP2	AT4G14690	4	148	4.50558	Wilcoxon	5712	2.50627	Brown-Forsythe
ATFYPP3	AT3G19980	3	185	4.35604	Wilcoxon	1157	3.59144	Brown-Forsythe
LD	AT4G02560	4	193	4.33028	Wilcoxon	9163	2.17147	Brown-Forsythe
ATFYPP3	AT3G19980	3	32	4.28567	EMMA	1157	3.59144	Brown-Forsythe
ATHB-2	AT4G16780	4	33	4.19755	EMMA	547	4.09856	Brown-Forsythe
TOC1	AT5G61380	5	50	3.80929	EMMA	1115	3.61704	Brown-Forsythe
PHYC	AT5G35840	5	62	3.67719	EMMA	321	4.44172	Brown-Forsythe
PGI1	AT4G24620	4	73	3.58915	EMMA	9258	2.163	Brown-Forsythe
GASA2	AT4G09610	4	85	3.51907	EMMA	450	4.23022	Brown-Forsythe
FKF1	AT1G68050	1	97	3.46113	EMMA	10595	2.06648	Brown-Forsythe
FRI	AT4G00650	4	107	3.43311	EMMA	4196	2.72816	Brown-Forsythe
CIP1	AT5G41790	5	149	3.21648	EMMA	7213	2.34032	Brown-Forsythe
VIM1	AT1G57820	1	188	3.11331	EMMA	1668	3.34231	Brown-Forsythe

Supplementary Table 44 - GWA (M) and vGWA (V) scores and ranks of trait 64: RP GH

Gene	Gene ID	Chr	Rank (M)	Score (M)	Test (M)	Rank (V)	Score (V)	Test (V)
DDF1	AT1G12610	1	11	5.09769	Wilcoxon	251	4.78225	Brown-Forsythe
GASA	AT5G15230	5	29	4.70264	Wilcoxon	1806	2.94381	Brown-Forsythe
ELF6	AT5G04240	5	49	4.49959	Wilcoxon	2274	2.78546	Brown-Forsythe
ICU2	AT5G67100	5	52	4.46503	Wilcoxon	1076	3.40069	Brown-Forsythe
FY	AT5G13480	5	58	4.38313	Wilcoxon	3105	2.54577	Brown-Forsythe
YAP169	AT5G07200	5	66	4.31713	Wilcoxon	94	5.61808	Brown-Forsythe
AGL18	AT3G57390	3	75	4.23367	Wilcoxon	91	5.64237	Brown-Forsythe
FLC	AT5G10140	5	84	4.21793	Wilcoxon	2227	2.78758	Brown-Forsythe
EMF1	AT5G11530	5	85	4.21759	Wilcoxon	20228	1.29188	Brown-Forsythe
ETC3	AT4G01060	4	99	4.16041	Wilcoxon	1716	2.9873	Brown-Forsythe
ICU2	AT5G67100	5	11	4.89777	EMMA	1076	3.40069	Brown-Forsythe
GA1	AT4G02780	4	24	4.36647	EMMA	751	3.70746	Brown-Forsythe
ELF6	AT5G04240	5	58	3.86547	EMMA	2274	2.78546	Brown-Forsythe
YAP169	AT5G07200	5	69	3.76682	EMMA	94	5.61808	Brown-Forsythe
TFL1	AT5G03840	5	74	3.72861	EMMA	1804	2.94474	Brown-Forsythe
TFL2	AT5G17690	5	93	3.65705	EMMA	367	4.38413	Brown-Forsythe
DDF1	AT1G12610	1	238	3.1776	EMMA	251	4.78225	Brown-Forsythe
AGL18	AT3G57390	3	244	3.15998	EMMA	91	5.64237	Brown-Forsythe
GASA2	AT4G09610	4	261	3.118	EMMA	3929	2.35647	Brown-Forsythe
LCL1	AT5G02840	5	302	3.05232	EMMA	3837	2.3741	Brown-Forsythe

Supplementary Table 45 - GWA (M) and vGWA (V) scores and ranks of trait 65: At1

Gene	Gene ID	Chr	Rank (M)	Score (M)	Test (M)	Rank (V)	Score (V)	Test (V)
AT5G49140	AT5G49140	5	10	4.57388	Wilcoxon	6154	1.40568	Brown-Forsythe
ATMKK5	AT3G21220	3	12	4.49561	Wilcoxon	467	2.4029	Brown-Forsythe
WRKY33	AT2G38470	2	24	4.25497	Wilcoxon	292	2.57577	Brown-Forsythe
AT4G16990	AT4G16990	4	39	4.04157	Wilcoxon	2074	1.8289	Brown-Forsythe
RIN4	AT3G25070	3	63	3.81092	Wilcoxon	3064	1.67792	Brown-Forsythe
AT5G45000	AT5G45000	5	67	3.78492	Wilcoxon	2351	1.78079	Brown-Forsythe
LEJ1	AT4G34120	4	75	3.75061	Wilcoxon	1868	1.86807	Brown-Forsythe
DND1	AT5G15410	5	109	3.60025	Wilcoxon	6445	1.38796	Brown-Forsythe
AT1G52900	AT1G52900	1	114	3.58772	Wilcoxon	793	2.19316	Brown-Forsythe
CDR1	AT5G33340	5	287	3.18746	Wilcoxon	6326	1.39528	Brown-Forsythe
AT5G49140	AT5G49140	5	4	5.15682	EMMA	6154	1.40568	Brown-Forsythe
AT1G43180	AT1G43180	1	23	4.02577	EMMA	23	4.02577	Brown-Forsythe
AT5G66640	AT5G66640	5	43	3.83221	EMMA	3056	1.67844	Brown-Forsythe
ATMKK5	AT3G21220	3	51	3.73997	EMMA	467	2.4029	Brown-Forsythe
AT4G19530	AT4G19530	4	56	3.65164	EMMA	457	2.41478	Brown-Forsythe
AT1G52900	AT1G52900	1	58	3.63487	EMMA	793	2.19316	Brown-Forsythe
WRKY33	AT2G38470	2	59	3.62881	EMMA	292	2.57577	Brown-Forsythe
AT5G45000	AT5G45000	5	70	3.53952	EMMA	2351	1.78079	Brown-Forsythe
PLDBETA1	AT2G42010	2	79	3.45365	EMMA	2000	1.84194	Brown-Forsythe
ACD6	AT4G14400	4	93	3.36124	EMMA	279	2.59933	Brown-Forsythe

Supplementary Table 46 - GWA (M) and vGWA (V) scores and ranks of trait 66: At1: CFU2

Gene	Gene ID	Chr	Rank (M)	Score (M)	Test (M)	Rank (V)	Score (V)	Test (V)
AT1G19160	AT1G19160	1	1	4.79892	Wilcoxon	8507	1.34115	Brown-Forsythe
AT5G66910	AT5G66910	5	24	3.64585	Wilcoxon	147	2.87982	Brown-Forsythe
AT5G27060	AT5G27060	5	34	3.58447	Wilcoxon	4340	1.60508	Brown-Forsythe
MOS4	AT3G18165	3	45	3.45712	Wilcoxon	3529	1.68251	Brown-Forsythe
AT5G46270	AT5G46270	5	62	3.39473	Wilcoxon	7116	1.41202	Brown-Forsythe
NPR4	AT4G19660	4	64	3.37843	Wilcoxon	4176	1.61921	Brown-Forsythe
WRKY11	AT4G31550	4	68	3.36308	Wilcoxon	3715	1.66487	Brown-Forsythe
RLM1	AT1G64070	1	69	3.35516	Wilcoxon	5175	1.53802	Brown-Forsythe
ER	AT2G26330	2	80	3.30235	Wilcoxon	1794	1.9371	Brown-Forsythe
AT3G26470	AT3G26470	3	83	3.29355	Wilcoxon	170	2.81437	Brown-Forsythe
JAZ1	AT1G19180	1	1	5.17929	EMMA	4921	1.55761	Brown-Forsythe
AT1G19230	AT1G19230	1	1	5.17929	EMMA	2393	1.83256	Brown-Forsythe
AT5G66910	AT5G66910	5	16	4.05476	EMMA	147	2.87982	Brown-Forsythe
RLM1	AT1G64070	1	18	4.02563	EMMA	5175	1.53802	Brown-Forsythe
AT5G27060	AT5G27060	5	29	3.77273	EMMA	4340	1.60508	Brown-Forsythe
MOS4	AT3G18165	3	44	3.69117	EMMA	3529	1.68251	Brown-Forsythe
RPP13	AT3G46530	3	54	3.57418	EMMA	970	2.169	Brown-Forsythe
AT1G77920	AT1G77920	1	56	3.56642	EMMA	406	2.4955	Brown-Forsythe
AT4G11210	AT4G11210	4	94	3.36662	EMMA	5754	1.49595	Brown-Forsythe
ER	AT2G26330	2	98	3.35	EMMA	1794	1.9371	Brown-Forsythe

Supplementary Table 47 - GWA (M) and vGWA (V) scores and ranks of trait 67: As

Gene	Gene ID	Chr	Rank (M)	Score (M)	Test (M)	Rank (V)	Score (V)	Test (V)
AT5G66640	AT5G66640	5	12	4.07289	Wilcoxon	692	2.59836	Brown-Forsythe
AT2G17060	AT2G17060	2	14	4.03954	Wilcoxon	557	2.71715	Brown-Forsythe
AT3G50950	AT3G50950	3	29	3.86687	Wilcoxon	2950	1.91419	Brown-Forsythe
AT3G23270	AT3G23270	3	45	3.71526	Wilcoxon	850	2.49296	Brown-Forsythe
AT4G16095	AT4G16095	4	47	3.66962	Wilcoxon	288	3.05332	Brown-Forsythe
AT4G19050	AT4G19050	4	51	3.59917	Wilcoxon	6572	1.56873	Brown-Forsythe
AT4G19060	AT4G19060	4	51	3.59917	Wilcoxon	9098	1.41968	Brown-Forsythe
EDR2	AT4G19040	4	51	3.59917	Wilcoxon	6572	1.56873	Brown-Forsythe
HR4	AT3G50480	3	63	3.56624	Wilcoxon	2057	2.06971	Brown-Forsythe
ATMKK3	AT5G40440	5	99	3.40708	Wilcoxon	1275	2.2887	Brown-Forsythe
AT5G66640	AT5G66640	5	2	5.82719	EMMA	692	2.59836	Brown-Forsythe
AT4G16095	AT4G16095	4	14	4.3661	EMMA	288	3.05332	Brown-Forsythe
AT4G11340	AT4G11340	4	40	3.85508	EMMA	5215	1.66217	Brown-Forsythe
JAZ7	AT2G34600	2	61	3.6394	EMMA	972	2.42538	Brown-Forsythe
AT2G17060	AT2G17060	2	62	3.63546	EMMA	557	2.71715	Brown-Forsythe
AT1G63730	AT1G63730	1	79	3.51266	EMMA	2130	2.0552	Brown-Forsythe
AT4G08450	AT4G08450	4	85	3.46815	EMMA	2134	2.05389	Brown-Forsythe
AT1G63750	AT1G63750	1	88	3.44879	EMMA	2130	2.0552	Brown-Forsythe
AT3G50950	AT3G50950	3	94	3.41773	EMMA	2950	1.91419	Brown-Forsythe
AT3G24020	AT3G24020	3	106	3.3816	EMMA	4477	1.72855	Brown-Forsythe

Supplementary Table 48 - GWA (M) and vGWA (V) scores and ranks of trait 68: As CFU2

Gene	Gene ID	Chr	Rank (M)	Score (M)	Test (M)	Rank (V)	Score (V)	Test (V)
AT4G13580	AT4G13580	4	3	4.44123	Wilcoxon	6981	1.5523	Brown-Forsythe
RLM1	AT1G64070	1	14	4.00032	Wilcoxon	964	2.41428	Brown-Forsythe
ATPP2-A5	AT1G65390	1	19	3.9191	Wilcoxon	5688	1.64216	Brown-Forsythe
LACS2	AT1G49430	1	21	3.90579	Wilcoxon	1868	2.11074	Brown-Forsythe
SOBER1	AT4G22300	4	25	3.86091	Wilcoxon	232	3.01884	Brown-Forsythe
AT1G72520	AT1G72520	1	30	3.78648	Wilcoxon	2295	2.02594	Brown-Forsythe
AT5G27060	AT5G27060	5	31	3.77779	Wilcoxon	62	3.5793	Brown-Forsythe
RDR6	AT3G49500	3	59	3.53862	Wilcoxon	3824	1.81065	Brown-Forsythe
AT4G12010	AT4G12010	4	71	3.47602	Wilcoxon	590	2.62398	Brown-Forsythe
AT1G69545	AT1G69545	1	76	3.44469	Wilcoxon	2413	2.00459	Brown-Forsythe
SOBER1	AT4G22300	4	5	4.09743	EMMA	232	3.01884	Brown-Forsythe
LACS2	AT1G49430	1	8	4.0383	EMMA	1868	2.11074	Brown-Forsythe
AT5G27060	AT5G27060	5	23	3.78357	EMMA	62	3.5793	Brown-Forsythe
ATPP2-A5	AT1G65390	1	29	3.70066	EMMA	5688	1.64216	Brown-Forsythe
AT1G72520	AT1G72520	1	39	3.56872	EMMA	2295	2.02594	Brown-Forsythe
AT5G43730	AT5G43730	5	44	3.53052	EMMA	317	2.8771	Brown-Forsythe
ATTRX3	AT5G42980	5	47	3.487	EMMA	3545	1.84371	Brown-Forsythe
OCP3	AT5G11270	5	49	3.47673	EMMA	1572	2.19283	Brown-Forsythe
AT1G69545	AT1G69545	1	55	3.40595	EMMA	2413	2.00459	Brown-Forsythe
AT5G11250	AT5G11250	5	78	3.29824	EMMA	1572	2.19283	Brown-Forsythe

Supplementary Table 49 - GWA (M) and vGWA (V) scores and ranks of trait 69: Bs

Gene	Gene ID	Chr	Rank (M)	Score (M)	Test (M)	Rank (V)	Score (V)	Test (V)
AT4G16095	AT4G16095	4	10	4.41328	Wilcoxon	4048	1.58729	Brown-Forsythe
AT4G23440	AT4G23440	4	27	4.09883	Wilcoxon	3871	1.60467	Brown-Forsythe
AT1G19160	AT1G19160	1	30	4.07674	Wilcoxon	3743	1.61904	Brown-Forsythe
JAZ1	AT1G19180	1	30	4.07674	Wilcoxon	3743	1.61904	Brown-Forsythe
MLO6	AT1G61560	1	36	4.01612	Wilcoxon	4557	1.53858	Brown-Forsythe
AT1G53350	AT1G53350	1	38	3.94515	Wilcoxon	3408	1.6548	Brown-Forsythe
ATRBOHB	AT1G09090	1	45	3.93928	Wilcoxon	5297	1.47691	Brown-Forsythe
AT5G43310	AT5G43310	5	58	3.79667	Wilcoxon	1109	2.10283	Brown-Forsythe
AT4G16095	AT4G16095	4	67	3.74302	Wilcoxon	4048	1.58729	Brown-Forsythe
AT1G63880	AT1G63880	1	68	3.72825	Wilcoxon	1718	1.91986	Brown-Forsythe
AT4G16095	AT4G16095	4	13	4.41998	EMMA	4048	1.58729	Brown-Forsythe
AT1G19160	AT1G19160	1	28	4.09252	EMMA	3743	1.61904	Brown-Forsythe
AT1G53350	AT1G53350	1	37	3.96331	EMMA	3408	1.6548	Brown-Forsythe
ATRBOHB	AT1G09090	1	43	3.8499	EMMA	5297	1.47691	Brown-Forsythe
AT3G05370	AT3G05370	3	45	3.84659	EMMA	2315	1.80678	Brown-Forsythe
AT4G23440	AT4G23440	4	53	3.75531	EMMA	3871	1.60467	Brown-Forsythe
AT5G43310	AT5G43310	5	54	3.75196	EMMA	1109	2.10283	Brown-Forsythe
AT5G66630	AT5G66630	5	61	3.66655	EMMA	6672	1.38542	Brown-Forsythe
EFR	AT5G20480	5	82	3.47834	EMMA	4752	1.52068	Brown-Forsythe
MLO6	AT1G61560	1	83	3.44776	EMMA	4557	1.53858	Brown-Forsythe

Supplementary Table 50 - GWA (M) and vGWA (V) scores and ranks of trait 70: Bs CFU2

Gene	Gene ID	Chr	Rank (M)	Score (M)	Test (M)	Rank (V)	Score (V)	Test (V)
ATRBOHB	AT1G09090	1	9	5.07903	Wilcoxon	187	2.81339	Brown-Forsythe
AT3G45860	AT3G45860	3	21	4.62539	Wilcoxon	1993	1.92142	Brown-Forsythe
IQD1	AT3G09710	3	26	4.4954	Wilcoxon	7614	1.41715	Brown-Forsythe
AT2G21030	AT2G21030	2	31	4.4307	Wilcoxon	3201	1.74144	Brown-Forsythe
ATMKK4	AT1G51660	1	33	4.41642	Wilcoxon	3097	1.75448	Brown-Forsythe
AT2G34930	AT2G34930	2	54	4.33938	Wilcoxon	3851	1.675	Brown-Forsythe
AT5G35180	AT5G35180	5	57	4.32595	Wilcoxon	20	3.71566	Brown-Forsythe
AT1G69545	AT1G69545	1	85	4.22319	Wilcoxon	3239	1.73768	Brown-Forsythe
AT4G36150	AT4G36150	4	101	4.14919	Wilcoxon	896	2.20897	Brown-Forsythe
AT5G44870	AT5G44870	5	104	4.13855	Wilcoxon	5682	1.53053	Brown-Forsythe
ATRBOHB	AT1G09090	1	9	4.28973	EMMA	187	2.81339	Brown-Forsythe
AT2G34930	AT2G34930	2	13	4.20684	EMMA	3851	1.675	Brown-Forsythe
HSP81-1	AT5G52640	5	22	4.10379	EMMA	22	4.10379	Brown-Forsythe
AT2G28670	AT2G28670	2	27	3.99455	EMMA	3934	1.66489	Brown-Forsythe
AT1G69545	AT1G69545	1	28	3.99212	EMMA	3239	1.73768	Brown-Forsythe
AT5G46270	AT5G46270	5	29	3.9514	EMMA	460	2.47384	Brown-Forsythe
AT5G17890	AT5G17890	5	87	3.46148	EMMA	125	2.995	Brown-Forsythe
RPP13	AT3G46530	3	102	3.37514	EMMA	14955	1.14407	Brown-Forsythe
IAR3	AT1G51760	1	174	3.11228	EMMA	5978	1.51147	Brown-Forsythe
PDF1.2c	AT5G44430	5	184	3.08855	EMMA	184	3.08855	Brown-Forsythe

Supplementary Table 51 - GWA (M) and vGWA (V) scores and ranks of trait 71: At2

Gene	Gene ID	Chr	Rank (M)	Score (M)	Test (M)	Rank (V)	Score (V)	Test (V)
AT1G72950	AT1G72950	1	11	4.85613	Wilcoxon	166	3.4232	Brown-Forsythe
NHL3	AT5G06320	5	17	4.7289	Wilcoxon	2543	2.08512	Brown-Forsythe
AT5G66640	AT5G66640	5	38	4.39514	Wilcoxon	527	2.83158	Brown-Forsythe
SGT1A	AT4G23570	4	53	4.20204	Wilcoxon	4792	1.80484	Brown-Forsythe
CDR1	AT5G33340	5	88	3.96357	Wilcoxon	607	2.75597	Brown-Forsythe
AT2G38870	AT2G38870	2	112	3.83425	Wilcoxon	411	2.95084	Brown-Forsythe
NPR4	AT4G19660	4	125	3.7961	Wilcoxon	1812	2.24134	Brown-Forsythe
AT5G49040	AT5G49040	5	130	3.77479	Wilcoxon	2271	2.13785	Brown-Forsythe
AGB1	AT4G34460	4	148	3.71499	Wilcoxon	379	2.98169	Brown-Forsythe
AT1G52660	AT1G52660	1	156	3.68745	Wilcoxon	1804	2.24339	Brown-Forsythe
AT5G63020	AT5G63020	5	1	6.60309	EMMA	476	2.87689	Brown-Forsythe
AT5G66640	AT5G66640	5	7	5.32394	EMMA	527	2.83158	Brown-Forsythe
AGB1	AT4G34460	4	10	5.07307	EMMA	379	2.98169	Brown-Forsythe
NPR4	AT4G19660	4	12	4.98988	EMMA	1812	2.24134	Brown-Forsythe
BAP1	AT3G61190	3	29	4.45171	EMMA	197	3.32819	Brown-Forsythe
ATNFXL1	AT1G10170	1	52	4.0498	EMMA	356	3.01201	Brown-Forsythe
AT2G33020	AT2G33020	2	54	4.04479	EMMA	1583	2.3022	Brown-Forsythe
BON1	AT5G61900	5	81	3.91437	EMMA	860	2.57479	Brown-Forsythe
AT4G13820	AT4G13820	4	85	3.89682	EMMA	5009	1.78328	Brown-Forsythe
RPS4	AT5G45250	5	90	3.85755	EMMA	1637	2.28505	Brown-Forsythe

Supplementary Table 52 - GWA (M) and vGWA (V) scores and ranks of trait 72: At2: CFU2

Gene	Gene ID	Chr	Rank (M)	Score (M)	Test (M)	Rank (V)	Score (V)	Test (V)
AT5G66640	AT5G66640	5	55	3.64309	Wilcoxon	1637	1.91458	Brown-Forsythe
AT4G19050	AT4G19050	4	58	3.61456	Wilcoxon	570	2.32396	Brown-Forsythe
EDR2	AT4G19040	4	58	3.61456	Wilcoxon	570	2.32396	Brown-Forsythe
AT2G34930	AT2G34930	2	78	3.45104	Wilcoxon	17583	1.01294	Brown-Forsythe
AT1G61100	AT1G61100	1	96	3.34982	Wilcoxon	3160	1.65708	Brown-Forsythe
OCP3	AT5G11270	5	101	3.32215	Wilcoxon	736	2.21983	Brown-Forsythe
AT2G15010	AT2G15010	2	115	3.2633	Wilcoxon	6064	1.40999	Brown-Forsythe
AT5G36930	AT5G36930	5	119	3.25328	Wilcoxon	5909	1.41869	Brown-Forsythe
PROPEP2	AT5G64890	5	127	3.22717	Wilcoxon	312	2.58244	Brown-Forsythe
AT3G50950	AT3G50950	3	138	3.19277	Wilcoxon	1512	1.94467	Brown-Forsythe
EDR2	AT4G19040	4	21	4.11656	EMMA	570	2.32396	Brown-Forsythe
AOC4	AT1G13280	1	54	3.64005	EMMA	7329	1.3393	Brown-Forsythe
AT5G66640	AT5G66640	5	57	3.62269	EMMA	1637	1.91458	Brown-Forsythe
AT1G61100	AT1G61100	1	59	3.61226	EMMA	3160	1.65708	Brown-Forsythe
OCP3	AT5G11270	5	70	3.53168	EMMA	736	2.21983	Brown-Forsythe
AT5G44870	AT5G44870	5	81	3.44641	EMMA	567	2.32615	Brown-Forsythe
RRS1	AT5G45260	5	83	3.44054	EMMA	2578	1.73799	Brown-Forsythe
AT1G69550	AT1G69550	1	96	3.36687	EMMA	495	2.38971	Brown-Forsythe
AT2G39430	AT2G39430	2	108	3.32689	EMMA	10513	1.20379	Brown-Forsythe
AT5G36930	AT5G36930	5	140	3.19907	EMMA	5909	1.41869	Brown-Forsythe

Supplementary Table 53 - GWA (M) and vGWA (V) scores and ranks of trait 73: As2

Gene	Gene ID	Chr	Rank (M)	Score (M)	Test (M)	Rank (V)	Score (V)	Test (V)
AT4G38700	AT4G38700	4	30	4.41049	Wilcoxon	10501	1.39534	Brown-Forsythe
AT4G13820	AT4G13820	4	60	3.99506	Wilcoxon	3590	1.97181	Brown-Forsythe
AT1G69710	AT1G69710	1	76	3.82345	Wilcoxon	4734	1.80875	Brown-Forsythe
AT1G22900	AT1G22900	1	77	3.82323	Wilcoxon	4804	1.80078	Brown-Forsythe
SGT1A	AT4G23570	4	87	3.75712	Wilcoxon	1662	2.42533	Brown-Forsythe
AT1G72950	AT1G72950	1	106	3.63602	Wilcoxon	786	2.94137	Brown-Forsythe
AT3G23270	AT3G23270	3	107	3.61739	Wilcoxon	3156	2.04076	Brown-Forsythe
ACD6	AT4G14400	4	112	3.60145	Wilcoxon	2101	2.27558	Brown-Forsythe
AT5G42140	AT5G42140	5	121	3.55917	Wilcoxon	5063	1.77084	Brown-Forsythe
AT1G12280	AT1G12280	1	135	3.49843	Wilcoxon	2540	2.16262	Brown-Forsythe
AT5G66640	AT5G66640	5	1	7.36257	EMMA	98	4.59999	Brown-Forsythe
NPR4	AT4G19660	4	27	5.00279	EMMA	289	3.65202	Brown-Forsythe
BAP1	AT3G61190	3	29	4.95259	EMMA	72	4.86886	Brown-Forsythe
AT5G41550	AT5G41550	5	42	4.67348	EMMA	29	5.71197	Brown-Forsythe
AOC4	AT1G13280	1	66	4.34368	EMMA	228	3.82442	Brown-Forsythe
AT1G72520	AT1G72520	1	70	4.33315	EMMA	17	6.00295	Brown-Forsythe
AT5G36930	AT5G36930	5	74	4.31233	EMMA	816	2.91534	Brown-Forsythe
AT3G28890	AT3G28890	3	75	4.30719	EMMA	24	5.93701	Brown-Forsythe
AT4G13820	AT4G13820	4	163	3.78358	EMMA	3590	1.97181	Brown-Forsythe
AT1G58390	AT1G58390	1	175	3.75097	EMMA	805	2.92324	Brown-Forsythe

Supplementary Table 54 - GWA (M) and vGWA (V) scores and ranks of trait 74: As2: CFU2

Gene	Gene ID	Chr	Rank (M)	Score (M)	Test (M)	Rank (V)	Score (V)	Test (V)
EFR	AT5G20480	5	5	4.67751	Wilcoxon	2344	1.87819	Brown-Forsythe
CYT1	AT2G39770	2	20	3.92728	Wilcoxon	2032	1.94303	Brown-Forsythe
AT4G14610	AT4G14610	4	27	3.82944	Wilcoxon	27	3.82944	Brown-Forsythe
AT5G23400	AT5G23400	5	63	3.55016	Wilcoxon	687	2.46189	Brown-Forsythe
AT3G14460	AT3G14460	3	68	3.51042	Wilcoxon	8911	1.3252	Brown-Forsythe
AT2G32660	AT2G32660	2	103	3.36218	Wilcoxon	4918	1.56897	Brown-Forsythe
LSD1	AT4G20380	4	126	3.2606	Wilcoxon	5343	1.53365	Brown-Forsythe
AT3G23270	AT3G23270	3	134	3.22429	Wilcoxon	2078	1.93458	Brown-Forsythe
AT5G46260	AT5G46260	5	143	3.20083	Wilcoxon	436	2.6835	Brown-Forsythe
EDR3	AT3G60190	3	202	3.02868	Wilcoxon	2661	1.82689	Brown-Forsythe
YSL7	AT1G65730	1	8	4.512	EMMA	250	2.96168	Brown-Forsythe
CYT1	AT2G39770	2	14	4.20155	EMMA	2032	1.94303	Brown-Forsythe
AT3G13650	AT3G13650	3	31	3.77221	EMMA	1699	2.01256	Brown-Forsythe
AT5G23400	AT5G23400	5	44	3.67826	EMMA	687	2.46189	Brown-Forsythe
OCP3	AT5G11270	5	47	3.65842	EMMA	2485	1.85503	Brown-Forsythe
EFR	AT5G20480	5	51	3.63768	EMMA	2344	1.87819	Brown-Forsythe
AT2G32660	AT2G32660	2	64	3.54676	EMMA	4918	1.56897	Brown-Forsythe
AT1G57830	AT1G57830	1	66	3.51751	EMMA	3654	1.68698	Brown-Forsythe
AT3G23270	AT3G23270	3	68	3.49712	EMMA	2078	1.93458	Brown-Forsythe
AT5G49040	AT5G49040	5	71	3.48929	EMMA	83	3.42965	Brown-Forsythe

Supplementary Table 55 - GWA (M) and vGWA (V) scores and ranks of trait 75: FW

Gene	Gene ID	Chr	Rank (M)	Score (M)	Test (M)	Rank (V)	Score (V)	Test (V)
ACD6	AT4G14400	4	108	3.67151	EMMA	317	2.70339	Brown-Forsythe

Supplementary Table 56 - GWA (M) and vGWA (V) scores and ranks of trait 76: DW

Gene	Gene ID	Chr	Rank (M)	Score (M)	Test (M)	Rank (V)	Score (V)	Test (V)
ACD6	AT4G14400	4	163	4.07261	Wilcoxon	597	2.53351	Brown-Forsythe
ACD6	AT4G14400	4	21	4.71524	EMMA	597	2.53351	Brown-Forsythe

Supplementary Table 57 - GWA (M) and vGWA (V) scores and ranks of trait 80: LN10

Gene	Gene ID	Chr	Rank (M)	Score (M)	Test (M)	Rank (V)	Score (V)	Test (V)
ATH1	AT4G32980	4	1	10.7383	Wilcoxon	196	5.73855	Brown-Forsythe
AGL17	AT2G22630	2	5	9.96091	Wilcoxon	307	5.3338	Brown-Forsythe
DOG1	AT5G45830	5	6	9.86254	Wilcoxon	29	7.56108	Brown-Forsythe
SVP	AT2G22540	2	8	9.73402	Wilcoxon	226	5.61853	Brown-Forsythe
sim-to-VRN1	AT4G33280	4	15	9.52932	Wilcoxon	92	6.37853	Brown-Forsythe
DFL2	AT4G03400	4	41	8.60168	Wilcoxon	97	6.35557	Brown-Forsythe
ATARP4	AT1G18450	1	70	7.9712	Wilcoxon	1651	4.10412	Brown-Forsythe
GASA5	AT3G02885	3	88	7.80368	Wilcoxon	218	5.65129	Brown-Forsythe
ATGID1B	AT3G63010	3	108	7.61248	Wilcoxon	3033	3.62857	Brown-Forsythe
SPA4	AT1G53090	1	117	7.54299	Wilcoxon	430	5.09252	Brown-Forsythe
DOG1	AT5G45830	5	7	4.96507	EMMA	29	7.56108	Brown-Forsythe
DFL1	AT5G54510	5	10	4.75953	EMMA	10599	2.57239	Brown-Forsythe
sim-to-VRN1	AT4G33280	4	11	4.72347	EMMA	92	6.37853	Brown-Forsythe
ATH1	AT4G32980	4	23	4.37626	EMMA	196	5.73855	Brown-Forsythe
SPA4	AT1G53090	1	24	4.27912	EMMA	430	5.09252	Brown-Forsythe
SPL4	AT1G53160	1	24	4.27912	EMMA	430	5.09252	Brown-Forsythe
FT	AT1G65480	1	36	4.16855	EMMA	918	4.56793	Brown-Forsythe
CIP7	AT4G27430	4	94	3.75715	EMMA	19797	2.02187	Brown-Forsythe
SVP	AT2G22540	2	102	3.72526	EMMA	226	5.61853	Brown-Forsythe
AGL17	AT2G22630	2	110	3.68391	EMMA	307	5.3338	Brown-Forsythe

Supplementary Table 58 - GWA (M) and vGWA (V) scores and ranks of trait 81: LN16

Gene	Gene ID	Chr	Rank (M)	Score (M)	Test (M)	Rank (V)	Score (V)	Test (V)
ATARP4	AT1G18450	1	9	10.8254	Wilcoxon	406	6.99885	Brown-Forsythe
sim-to-VRN1	AT4G33280	4	25	10.0518	Wilcoxon	7108	3.04935	Brown-Forsythe
ETC3	AT4G01060	4	34	9.90458	Wilcoxon	235	7.93987	Brown-Forsythe
AGL17	AT2G22630	2	35	9.8285	Wilcoxon	61	9.57657	Brown-Forsythe
ATH1	AT4G32980	4	42	9.81923	Wilcoxon	1732	4.81402	Brown-Forsythe
SVP	AT2G22540	2	88	9.12519	Wilcoxon	137	8.72269	Brown-Forsythe
DFL2	AT4G03400	4	94	8.99851	Wilcoxon	1198	5.39183	Brown-Forsythe
FLC	AT5G10140	5	154	8.28101	Wilcoxon	1076	5.5463	Brown-Forsythe
YAP169	AT5G07200	5	162	8.23112	Wilcoxon	874	5.84203	Brown-Forsythe
SPA2	AT4G11110	4	199	8.04169	Wilcoxon	3082	3.9956	Brown-Forsythe
ETC3	AT4G01060	4	2	5.5663	EMMA	235	7.93987	Brown-Forsythe
sim-to-FRI	AT4G00690	4	29	4.27582	EMMA	127	8.82991	Brown-Forsythe
DFL2	AT4G00650	4	29	4.27582	EMMA	5016	3.41872	Brown-Forsythe
sim-to-SRR1	AT4G03400	4	41	4.05769	EMMA	1198	5.39183	Brown-Forsythe
GA1	AT5G59570	5	92	3.65582	EMMA	4694	3.49108	Brown-Forsythe
YAP169	AT5G59560	5	92	3.65582	EMMA	4694	3.49108	Brown-Forsythe
sim-to-VRN1	AT4G02780	4	100	3.61767	EMMA	1931	4.65843	Brown-Forsythe
FPF1	AT5G07200	5	118	3.52327	EMMA	874	5.84203	Brown-Forsythe
ESD4	AT4G33280	4	137	3.45341	EMMA	7108	3.04935	Brown-Forsythe
PCL1	AT5G24860	5	182	3.26357	EMMA	2752	4.15135	Brown-Forsythe

Supplementary Table 59 - GWA (M) and vGWA (V) scores and ranks of trait 82: LN22

Gene	Gene ID	Chr	Rank (M)	Score (M)	Test (M)	Rank (V)	Score (V)	Test (V)
ATARP4	AT1G18450	1	5	10.2239	Wilcoxon	384	6.64017	Brown-Forsythe
AGL17	AT2G22630	2	10	9.78994	Wilcoxon	105	8.09152	Brown-Forsythe
DFL2	AT4G03400	4	13	9.67532	Wilcoxon	48	9.09848	Brown-Forsythe
ETC3	AT4G01060	4	24	9.38415	Wilcoxon	643	6.01769	Brown-Forsythe
ATGA2OX7	AT1G50960	1	25	9.36401	Wilcoxon	150	7.71517	Brown-Forsythe
SVP	AT2G22540	2	54	8.97539	Wilcoxon	105	8.09152	Brown-Forsythe
ATH1	AT4G32980	4	56	8.96646	Wilcoxon	390	6.61824	Brown-Forsythe
sim-to-VRN1	AT4G33280	4	97	8.54422	Wilcoxon	1211	5.20285	Brown-Forsythe
FRI	AT4G00650	4	137	8.26798	Wilcoxon	788	5.76969	Brown-Forsythe
DOG1	AT5G45830	5	179	7.91885	Wilcoxon	1171	5.23283	Brown-Forsythe
ETC3	AT4G01060	4	4	5.30509	EMMA	643	6.01769	Brown-Forsythe
ATGA2OX7	AT1G50960	1	18	4.70901	EMMA	150	7.71517	Brown-Forsythe
DOG1	AT5G45830	5	29	4.37363	EMMA	1171	5.23283	Brown-Forsythe
DFL2	AT4G03400	4	37	4.19623	EMMA	48	9.09848	Brown-Forsythe
GA1	AT4G02780	4	38	4.18079	EMMA	327	6.85181	Brown-Forsythe
sim-to-ESD4	AT4G00690	4	47	4.0692	EMMA	451	6.45105	Brown-Forsythe
FRI	AT4G00650	4	47	4.0692	EMMA	788	5.76969	Brown-Forsythe
FLC	AT5G10140	5	52	3.96494	EMMA	6	11.5397	Brown-Forsythe
CRY1	AT4G08920	4	67	3.86083	EMMA	53	8.94445	Brown-Forsythe
ATHB51	AT5G03790	5	69	3.8371	EMMA	6201	3.37727	Brown-Forsythe

Supplementary Table 60 - GWA (M) and vGWA (V) scores and ranks of trait 158: Silique 16

Gene	Gene ID	Chr	Rank (M)	Score (M)	Test (M)	Rank (V)	Score (V)	Test (V)
GA5	AT4G25420	4	28	4.0222	Wilcoxon	18970	1.11379	Brown-Forsythe
CUL4	AT5G46210	5	34	3.95142	Wilcoxon	344	3.07096	Brown-Forsythe
RGA1	AT2G01570	2	54	3.73416	Wilcoxon	4021	1.77721	Brown-Forsythe
ATMYB65	AT3G11440	3	64	3.65914	Wilcoxon	4162	1.76877	Brown-Forsythe
SPY	AT3G11540	3	64	3.65914	Wilcoxon	836	2.52117	Brown-Forsythe
AGL15	AT5G13790	5	88	3.51928	Wilcoxon	2064	2.07794	Brown-Forsythe
AGL14	AT4G11880	4	91	3.50155	Wilcoxon	1011	2.43534	Brown-Forsythe
FUS3	AT3G26790	3	94	3.49514	Wilcoxon	13715	1.25722	Brown-Forsythe
LIP1	AT5G64813	5	99	3.48472	Wilcoxon	388	3.00956	Brown-Forsythe
SNZ	AT2G39250	2	109	3.45798	Wilcoxon	1438	2.27417	Brown-Forsythe
FLK	AT3G04610	3	8	4.89322	EMMA	2792	1.96129	Brown-Forsythe
AGL15	AT5G13790	5	11	4.86601	EMMA	2064	2.07794	Brown-Forsythe
GA5	AT4G25420	4	19	4.24695	EMMA	18970	1.11379	Brown-Forsythe
DFL1	AT5G54510	5	35	3.96054	EMMA	758	2.56297	Brown-Forsythe
AGL14	AT4G11880	4	53	3.69279	EMMA	1011	2.43534	Brown-Forsythe
AT3G04510	AT3G04510	3	57	3.65124	EMMA	934	2.4696	Brown-Forsythe
ATMYB65	AT3G11440	3	63	3.56935	EMMA	4162	1.76877	Brown-Forsythe
SPY	AT3G11540	3	63	3.56935	EMMA	836	2.52117	Brown-Forsythe
CUL4	AT5G46210	5	64	3.56468	EMMA	344	3.07096	Brown-Forsythe
ATHXK1	AT4G29130	4	91	3.44387	EMMA	474	2.81581	Brown-Forsythe

Supplementary Table 61 - GWA (M) and vGWA (V) scores and ranks of trait 159: Silique 22

Gene	Gene ID	Chr	Rank (M)	Score (M)	Test (M)	Rank (V)	Score (V)	Test (V)
DDF1	AT1G12610	1	15	5.9721	Wilcoxon	3122	1.89109	Brown-Forsythe
CIP1	AT5G41790	5	31	5.9721	Wilcoxon	3024	1.90832	Brown-Forsythe
AP2	AT4G36920	4	41	5.39903	Wilcoxon	1815	2.15319	Brown-Forsythe
FCA	AT4G16280	4	66	5.25912	Wilcoxon	1240	2.32571	Brown-Forsythe
PHYD	AT4G16250	4	66	5.25912	Wilcoxon	1240	2.32571	Brown-Forsythe
AMP1	AT3G54720	3	206	4.62771	Wilcoxon	800	2.50882	Brown-Forsythe
HY5	AT5G11260	5	210	4.60944	Wilcoxon	553	2.64642	Brown-Forsythe
YAP169	AT5G07200	5	222	4.58577	Wilcoxon	307	2.96771	Brown-Forsythe
HAP2A	AT5G12840	5	240	4.55483	Wilcoxon	801	2.50538	Brown-Forsythe
VIP1	AT1G43700	1	376	4.30731	Wilcoxon	3004	1.9095	Brown-Forsythe
GA1	AT4G02780	4	8	4.60044	EMMA	2421	2.00567	Brown-Forsythe
ICU2	AT5G67100	5	23	4.09424	EMMA	3763	1.79941	Brown-Forsythe
DFL1	AT5G54510	5	33	3.89471	EMMA	5402	1.63757	Brown-Forsythe
TCH2	AT5G37770	5	46	3.81935	EMMA	5518	1.62681	Brown-Forsythe
DDF1	AT1G12610	1	51	3.74794	EMMA	3122	1.89109	Brown-Forsythe
SPL4	AT1G53160	1	58	3.7192	EMMA	545	2.64769	Brown-Forsythe
SEPALLATA4	AT2G03710	2	77	3.68256	EMMA	2513	1.98143	Brown-Forsythe
AT3G21320	AT3G21320	3	85	3.59299	EMMA	2983	1.91	Brown-Forsythe
ELF4	AT2G40080	2	153	3.19364	EMMA	1230	2.3346	Brown-Forsythe
CRY1	AT4G08920	4	171	3.13874	EMMA	2772	1.95188	Brown-Forsythe

Supplementary Table 62 - GWA (M) and vGWA (V) scores and ranks of trait 163: Germ 22

Gene	Gene ID	Chr	Rank (M)	Score (M)	Test (M)	Rank (V)	Score (V)	Test (V)
EER5	AT2G19560	2	1	6.89057	Wilcoxon	171	3.29939	Brown-Forsythe
SCD1	AT1G49040	1	9	4.85116	Wilcoxon	39	3.85964	Brown-Forsythe
FT	AT1G65480	1	22	4.17583	Wilcoxon	2145	2.05289	Brown-Forsythe
SEPALLATA3	AT1G24260	1	24	4.15532	Wilcoxon	5	5.04646	Brown-Forsythe
SPA2	AT4G11110	4	37	4.00096	Wilcoxon	351	2.89198	Brown-Forsythe
RGL1	AT1G66350	1	39	3.99895	Wilcoxon	3258	1.85072	Brown-Forsythe
EZA1	AT4G02020	4	48	3.82397	Wilcoxon	38	3.8598	Brown-Forsythe
sim-to-DOG1	AT4G18660	4	68	3.73996	Wilcoxon	7530	1.47331	Brown-Forsythe
LHY	AT1G01060	1	74	3.70853	Wilcoxon	154	3.33482	Brown-Forsythe
EER5	AT2G19560	2	1	5.5983	EMMA	171	3.29939	Brown-Forsythe
SCD1	AT1G49040	1	4	4.95534	EMMA	39	3.85964	Brown-Forsythe
SEPALLATA3	AT1G24260	1	5	4.77771	EMMA	5	5.04646	Brown-Forsythe
SPA2	AT4G11110	4	12	4.36052	EMMA	351	2.89198	Brown-Forsythe
FHY1	AT2G37678	2	27	4.01556	EMMA	4	5.45331	Brown-Forsythe
TIC	AT3G22380	3	29	3.99637	EMMA	29	4.06876	Brown-Forsythe
HLS1	AT4G37580	4	34	3.975	EMMA	64	3.68967	Brown-Forsythe
EZA1	AT4G02020	4	45	3.82084	EMMA	38	3.8598	Brown-Forsythe
LHY	AT1G01060	1	76	3.57447	EMMA	154	3.33482	Brown-Forsythe
FRS1	AT4G19990	4	85	3.50848	EMMA	151	3.33993	Brown-Forsythe

Supplementary Table 63 - GWA (M) and vGWA (V) scores and ranks of trait 164: Width 10

Gene	Gene ID	Chr	Rank (M)	Score (M)	Test (M)	Rank (V)	Score (V)	Test (V)
TET3	AT3G45600	3	2	7.83245	Wilcoxon	3358	2.0686	Brown-Forsythe
SAB	AT1G58250	1	10	6.09059	Wilcoxon	2868	2.15045	Brown-Forsythe
BIN4	AT5G24630	5	106	4.17852	Wilcoxon	3355	2.06912	Brown-Forsythe
CCH	AT3G56240	3	282	3.60952	Wilcoxon	2812	2.15914	Brown-Forsythe
TIP1	AT5G20350	5	424	3.3769	Wilcoxon	147	3.6826	Brown-Forsythe
REM1	AT4G31610	4	493	3.28949	Wilcoxon	118	3.78288	Brown-Forsythe
TET3	AT3G45600	3	5	6.99357	EMMA	3358	2.0686	Brown-Forsythe
SAB	AT1G58250	1	38	4.13247	EMMA	2868	2.15045	Brown-Forsythe

Supplementary Table 64 - GWA (M) and vGWA (V) scores and ranks of trait 165: Width 16

Gene	Gene ID	Chr	Rank (M)	Score (M)	Test (M)	Rank (V)	Score (V)	Test (V)
WUS	AT2G17950	2	13	4.63002	Wilcoxon	22164	1.13848	Brown-Forsythe
CCH	AT3G56240	3	63	4.00711	Wilcoxon	232	3.36007	Brown-Forsythe
CPC	AT2G46410	2	133	3.65904	Wilcoxon	1872	2.32503	Brown-Forsythe
BAM1	AT5G65700	5	164	3.51707	Wilcoxon	19901	1.19012	Brown-Forsythe
BIN4	AT5G24630	5	202	3.43691	Wilcoxon	516	2.94741	Brown-Forsythe
BAM2	AT3G49670	3	245	3.35871	Wilcoxon	3942	1.96774	Brown-Forsythe
TWD1	AT3G21640	3	251	3.35373	Wilcoxon	727	2.78206	Brown-Forsythe
ELO3	AT5G50320	5	17	4.34187	EMMA	14530	1.34022	Brown-Forsythe
CCH	AT3G56240	3	23	4.18171	EMMA	232	3.36007	Brown-Forsythe
CPC	AT2G46410	2	50	3.72607	EMMA	1872	2.32503	Brown-Forsythe
BIN4	AT5G24630	5	90	3.41288	EMMA	516	2.94741	Brown-Forsythe
WUS	AT2G17950	2	260	2.89848	EMMA	22164	1.13848	Brown-Forsythe

Supplementary Table 65 - GWA (M) and vGWA (V) scores and ranks of trait 166: Width 22

Gene	Gene ID	Chr	Rank (M)	Score (M)	Test (M)	Rank (V)	Score (V)	Test (V)
ELO1	AT3G11220	3	7	4.42509	Wilcoxon	88	3.45032	Brown-Forsythe
SHR	AT4G37650	4	234	3.13121	Wilcoxon	6977	1.50395	Brown-Forsythe
ZCW32	AT1G59640	1	352	2.94932	Wilcoxon	5746	1.58302	Brown-Forsythe
SMP2	AT4G37120	4	356	2.93903	Wilcoxon	5664	1.58791	Brown-Forsythe
BIN4	AT5G24630	5	357	2.93893	Wilcoxon	1785	2.06544	Brown-Forsythe
SHD	AT4G24190	4	403	2.88857	Wilcoxon	2870	1.87163	Brown-Forsythe
ELO1	AT3G11220	3	52	3.4997	EMMA	88	3.45032	Brown-Forsythe
SHR	AT4G37650	4	291	2.79222	EMMA	6977	1.50395	Brown-Forsythe
MSL3	AT1G58200	1	363	2.70024	EMMA	1479	2.13985	Brown-Forsythe
SHR	AT4G37650	4	375	2.68857	EMMA	6977	1.50395	Brown-Forsythe
OLEO2	AT5G40420	5	382	2.67872	EMMA	28	3.86851	Brown-Forsythe

Supplementary Table 66 - GWA (M) and vGWA (V) scores and ranks of trait 173: Leaf serr 10

Gene	Gene ID	Chr	Rank (M)	Score (M)	Test (M)	Rank (V)	Score (V)	Test (V)
AT4G18390	AT4G18390	4	24	5.27395	Wilcoxon	2467	1.90772	Brown-Forsythe
PAS1	AT3G54010	3	39	5.05486	Wilcoxon	2608	1.88206	Brown-Forsythe
PTF1	AT3G02150	3	91	4.56632	Wilcoxon	11499	1.26279	Brown-Forsythe
SE	AT2G27100	2	196	4.17463	Wilcoxon	1122	2.22147	Brown-Forsythe
RPS15AD	AT3G46040	3	197	4.17441	Wilcoxon	4739	1.6307	Brown-Forsythe
ATS	AT5G42630	5	205	4.15439	Wilcoxon	5437	1.5741	Brown-Forsythe
MIR173	AT3G23125	3	258	4.05334	Wilcoxon	258	4.05334	Brown-Forsythe
ATL5	AT3G25520	3	368	3.85108	Wilcoxon	2091	1.96603	Brown-Forsythe
CYC1	AT4G37490	4	376	3.84515	Wilcoxon	14426	1.16374	Brown-Forsythe
RIK	AT3G29390	3	395	3.81904	Wilcoxon	9830	1.33158	Brown-Forsythe
AT4G18390	AT4G18390	4	14	4.07285	EMMA	2467	1.90772	Brown-Forsythe
RPS15AD	AT3G46040	3	92	3.25956	EMMA	4739	1.6307	Brown-Forsythe
TAS1A	AT2G27400	2	204	2.95069	EMMA	204	2.95069	Brown-Forsythe
SE	AT2G27100	2	208	2.94344	EMMA	1122	2.22147	Brown-Forsythe
ATS	AT5G42630	5	221	2.92632	EMMA	5437	1.5741	Brown-Forsythe
AXR1	AT1G05180	1	235	2.90649	EMMA	2015	1.98904	Brown-Forsythe
AT1G33140	AT1G33140	1	269	2.8609	EMMA	1077	2.23989	Brown-Forsythe
AT1G29890	AT1G29890	1	299	2.80461	EMMA	144	3.01333	Brown-Forsythe
PAS1	AT3G54010	3	314	2.79022	EMMA	2608	1.88206	Brown-Forsythe
PTF1	AT3G02150	3	371	2.71172	EMMA	11499	1.26279	Brown-Forsythe

Supplementary Table 67 - GWA (M) and vGWA (V) scores and ranks of trait 174: Leaf serr 16

Gene	Gene ID	Chr	Rank (M)	Score (M)	Test (M)	Rank (V)	Score (V)	Test (V)
AGO1	AT1G48410	1	84	3.95945	Wilcoxon	21946	1.2292	Brown-Forsythe
ANAC065	AT3G56560	3	129	3.78797	Wilcoxon	3284	2.23167	Brown-Forsythe
ANAC082	AT5G09330	5	274	3.40502	Wilcoxon	607	3.1074	Brown-Forsythe
AT1G29890	AT1G29890	1	280	3.39571	Wilcoxon	1058	2.80565	Brown-Forsythe
TCP3	AT1G53230	1	295	3.36742	Wilcoxon	3371	2.21832	Brown-Forsythe
AT4G32700	AT4G32700	4	402	3.20388	Wilcoxon	4042	2.12273	Brown-Forsythe
TCP4	AT3G15030	3	481	3.11765	Wilcoxon	6030	1.92521	Brown-Forsythe
CYP72C1	AT1G17060	1	498	3.10215	Wilcoxon	3119	2.25445	Brown-Forsythe
RIK	AT3G29390	3	145	3.13165	EMMA	2735	2.31421	Brown-Forsythe
ANAC013	AT1G32870	1	155	3.08794	EMMA	1	8.67585	Brown-Forsythe
ANAC065	AT3G56560	3	204	2.94685	EMMA	3284	2.23167	Brown-Forsythe
CYP72C1	AT1G17060	1	205	2.93996	EMMA	3119	2.25445	Brown-Forsythe
AGO1	AT1G48410	1	230	2.88852	EMMA	21946	1.2292	Brown-Forsythe
ANAC079	AT5G07680	5	264	2.8361	EMMA	2214	2.42188	Brown-Forsythe
ANAC074	AT4G28530	4	288	2.80952	EMMA	2647	2.32946	Brown-Forsythe
AT1G33140	AT1G33140	1	306	2.78485	EMMA	233	3.74141	Brown-Forsythe
CAS1	AT2G07050	2	340	2.75006	EMMA	3705	2.16549	Brown-Forsythe

Supplementary Table 68 - GWA (M) and vGWA (V) scores and ranks of trait 175: Leaf serr 22

Gene	Gene ID	Chr	Rank (M)	Score (M)	Test (M)	Rank (V)	Score (V)	Test (V)
KNAT3	AT5G25220	5	62	4.30933	Wilcoxon	1307	2.61596	Brown-Forsythe
STV1	AT3G53020	3	76	4.15559	Wilcoxon	2813	2.16822	Brown-Forsythe
MIR164	AT5G01747	5	81	4.07561	Wilcoxon	81	4.07561	Brown-Forsythe
RIK	AT3G29390	3	82	4.05479	Wilcoxon	6278	1.73208	Brown-Forsythe
ANAC074	AT4G28530	4	129	3.85693	Wilcoxon	428	3.25967	Brown-Forsythe
NPH4	AT5G20730	5	375	3.35214	Wilcoxon	2307	2.274	Brown-Forsythe
KNAT3	AT5G25220	5	4	5.0011	EMMA	1307	2.61596	Brown-Forsythe
STV1	AT3G53020	3	11	4.36132	EMMA	2813	2.16822	Brown-Forsythe
ANAC074	AT4G28530	4	66	3.42028	EMMA	428	3.25967	Brown-Forsythe
ANAC103	AT5G64060	5	100	3.24533	EMMA	13870	1.30539	Brown-Forsythe
MIR164	AT5G01747	5	104	3.22861	EMMA	104	3.22861	Brown-Forsythe
CUC1	AT3G15170	3	281	2.79598	EMMA	1072	2.72444	Brown-Forsythe
MIR160	AT5G46845	5	408	2.63898	EMMA	408	2.63898	Brown-Forsythe
AGO1	AT1G48410	1	416	2.62825	EMMA	2811	2.16862	Brown-Forsythe
ANAC104/XND1	AT5G64530	5	436	2.60966	EMMA	414	3.27534	Brown-Forsythe
AT1G29890	AT1G29890	1	444	2.60077	EMMA	245	3.61462	Brown-Forsythe

Supplementary Table 69 - GWA (M) and vGWA (V) scores and ranks of trait 182: Hypocotyl length

Gene	Gene ID	Chr	Rank (M)	Score (M)	Test (M)	Rank (V)	Score (V)	Test (V)
LSH1	AT5G28490	5	28	5.12859	Wilcoxon	5947	1.49923	Brown-Forsythe
HFR1	AT1G02340	1	29	5.0957	Wilcoxon	11000	1.27509	Brown-Forsythe
CYCD1;1	AT1G70210	1	96	4.55502	Wilcoxon	2133	1.88787	Brown-Forsythe
ATPSK4	AT3G49780	3	112	4.46003	Wilcoxon	2744	1.78508	Brown-Forsythe
DFL1	AT5G54510	5	120	4.4513	Wilcoxon	2122	1.88922	Brown-Forsythe
HLS1	AT4G37580	4	148	4.32914	Wilcoxon	2651	1.79967	Brown-Forsythe
DOG1	AT5G45830	5	167	4.23165	Wilcoxon	2546	1.81422	Brown-Forsythe
PKL	AT2G25170	2	178	4.21388	Wilcoxon	1069	2.17219	Brown-Forsythe
GA20OX2	AT5G51810	5	191	4.16765	Wilcoxon	10266	1.30008	Brown-Forsythe
DFL1	AT5G54510	5	5	5.35688	EMMA	2122	1.88922	Brown-Forsythe
DDF1	AT1G12610	1	31	4.31065	EMMA	1394	2.06502	Brown-Forsythe
VIP5	AT1G61040	1	62	3.88562	EMMA	2592	1.80652	Brown-Forsythe
VRN2	AT4G16845	4	74	3.873	EMMA	1828	1.95372	Brown-Forsythe
DFL2	AT4G03400	4	93	3.71775	EMMA	623	2.4259	Brown-Forsythe
EMF1	AT5G11530	5	103	3.65649	EMMA	167	3.18733	Brown-Forsythe
AGL15	AT5G13790	5	109	3.60681	EMMA	8798	1.35634	Brown-Forsythe
LEP	AT5G13910	5	109	3.60681	EMMA	8798	1.35634	Brown-Forsythe
LIP1	AT5G64813	5	124	3.55111	EMMA	7541	1.41006	Brown-Forsythe
GIL1	AT5G58960	5	125	3.54923	EMMA	4002	1.63766	Brown-Forsythe

Supplementary Table 70 - GWA (M) and vGWA (V) scores and ranks of trait 183: Trichome avg C

Gene	Gene ID	Chr	Rank (M)	Score (M)	Test (M)	Rank (V)	Score (V)	Test (V)
TCL2	AT2G30424	2	1	6.03849	Wilcoxon	6085	1.98674	Brown-Forsythe
TCL1	AT2G30432	2	1	6.03849	Wilcoxon	6085	1.98674	Brown-Forsythe
ETC2	AT2G30420	2	1	6.03849	Wilcoxon	4064	2.20327	Brown-Forsythe
ACD6	AT4G14400	4	66	4.63223	Wilcoxon	2111	2.56886	Brown-Forsythe
TTG1	AT5G24520	5	107	4.45996	Wilcoxon	3129	2.35108	Brown-Forsythe
HDG9	AT5G17320	5	133	4.3383	Wilcoxon	675	3.24627	Brown-Forsythe
CPC	AT2G46410	2	284	4.0376	Wilcoxon	1609	2.72076	Brown-Forsythe
ELC	AT3G12400	3	402	3.87148	Wilcoxon	836	3.0924	Brown-Forsythe
TCL2	AT2G30424	2	6	4.75326	EMMA	6085	1.98674	Brown-Forsythe
TCL1	AT2G30432	2	6	4.75326	EMMA	6085	1.98674	Brown-Forsythe
ETC2	AT2G30420	2	6	4.75326	EMMA	4064	2.20327	Brown-Forsythe
RHL2	AT5G02820	5	103	3.24969	EMMA	1928	2.61223	Brown-Forsythe
ACD6	AT4G14400	4	123	3.19616	EMMA	2111	2.56886	Brown-Forsythe
HDG1	AT3G61150	3	219	2.92683	EMMA	882	3.06287	Brown-Forsythe
STI	AT2G02480	2	253	2.86963	EMMA	3762	2.24372	Brown-Forsythe
GL1	AT3G27920	3	259	2.85844	EMMA	142	4.23851	Brown-Forsythe
TTG1	AT5G24520	5	469	2.59518	EMMA	3129	2.35108	Brown-Forsythe

Supplementary Table 71 - GWA (M) and vGWA (V) scores and ranks of trait 184: Trichome avg JA

Gene	Gene ID	Chr	Rank (M)	Score (M)	Test (M)	Rank (V)	Score (V)	Test (V)
TCL2	AT2G30424	2	3	6.44673	Wilcoxon	7902	1.74515	Brown-Forsythe
TCL1	AT2G30432	2	3	6.44673	Wilcoxon	7902	1.74515	Brown-Forsythe
ETC2	AT2G30420	2	3	6.44673	Wilcoxon	7902	1.74515	Brown-Forsythe
ETC3	AT4G01060	4	5	6.28312	Wilcoxon	380	3.30902	Brown-Forsythe
HDG9	AT5G17320	5	120	4.52655	Wilcoxon	1839	2.4655	Brown-Forsythe
ACD6	AT4G14400	4	174	4.40995	Wilcoxon	4225	2.04613	Brown-Forsythe
TTG1	AT5G24520	5	187	4.35215	Wilcoxon	4840	1.98879	Brown-Forsythe
ELC	AT3G12400	3	359	4.00356	Wilcoxon	5003	1.97207	Brown-Forsythe
KINESIN	AT3G16630	3	450	3.87782	Wilcoxon	15480	1.41409	Brown-Forsythe
TCL2	AT2G30424	2	1	7.28113	EMMA	7902	1.74515	Brown-Forsythe
TCL1	AT2G30432	2	1	7.28113	EMMA	7902	1.74515	Brown-Forsythe
ETC2	AT2G30420	2	1	7.28113	EMMA	7902	1.74515	Brown-Forsythe
ETC3	AT4G01060	4	30	4.16251	EMMA	380	3.30902	Brown-Forsythe
KINESIN	AT3G16630	3	55	3.70109	EMMA	15480	1.41409	Brown-Forsythe
TTG1	AT5G24520	5	154	3.17772	EMMA	4840	1.98879	Brown-Forsythe
GL1	AT3G27920	3	213	3.02522	EMMA	6312	1.85371	Brown-Forsythe
RHL2	AT5G02820	5	220	3.01372	EMMA	21909	1.24395	Brown-Forsythe
HDG9	AT5G17320	5	239	2.97729	EMMA	1839	2.4655	Brown-Forsythe
KLK	AT5G18410	5	277	2.90741	EMMA	3028	2.21072	Brown-Forsythe

Supplementary Table 72 - GWA (M) and vGWA (V) scores and ranks of trait 185: Aphid number

Gene	Gene ID	Chr	Rank (M)	Score (M)	Test (M)	Rank (V)	Score (V)	Test (V)
IMS1	AT1G74040	1	1	4.15926	Wilcoxon	3565	1.69682	Brown-Forsythe
MOS2	AT1G33520	1	3	4.02606	Wilcoxon	955	2.26378	Brown-Forsythe
AT4G16990	AT4G16990	4	10	3.77507	Wilcoxon	1567	2.03517	Brown-Forsythe
MAM3	AT5G23020	5	47	3.25185	Wilcoxon	1196	2.15197	Brown-Forsythe
NPR1	AT1G64280	1	167	2.85258	Wilcoxon	5854	1.49756	Brown-Forsythe
NIMIN-2	AT3G25882	3	402	2.53541	Wilcoxon	215	3.17022	Brown-Forsythe
MAM1	AT5G23010	5	439	2.5144	Wilcoxon	1196	2.15197	Brown-Forsythe
MOS2	AT1G33520	1	7	4.17368	EMMA	955	2.26378	Brown-Forsythe
IMS1	AT1G74040	1	9	4.11427	EMMA	3565	1.69682	Brown-Forsythe
AT4G16990	AT4G16990	4	20	3.84907	EMMA	1567	2.03517	Brown-Forsythe
MAM3	AT5G23020	5	62	3.307	EMMA	1196	2.15197	Brown-Forsythe
NIMIN-2	AT3G25882	3	132	3.05833	EMMA	215	3.17022	Brown-Forsythe
ATMYC2	AT1G32640	1	308	2.71974	EMMA	2726	1.79943	Brown-Forsythe
CYP83A1	AT4G13770	4	327	2.70766	EMMA	29	4.05282	Brown-Forsythe
ATTPS03	AT4G16740	4	345	2.68785	EMMA	1169	2.16164	Brown-Forsythe
AT5G51630	AT5G51630	5	410	2.61498	EMMA	750	2.39705	Brown-Forsythe
AT3G28740	AT3G28740	3	500	2.54324	EMMA	129	3.57823	Brown-Forsythe

Supplementary Table 73 - GWA (M) and vGWA (V) scores and ranks of trait 186: Bacterial titer

Gene	Gene ID	Chr	Rank (M)	Score (M)	Test (M)	Rank (V)	Score (V)	Test (V)
ATMPK6	AT2G43790	2	35	5.11922	Wilcoxon	13993	1.47872	Brown-Forsythe
AT5G44870	AT5G44870	5	105	4.57335	Wilcoxon	9011	1.69118	Brown-Forsythe
SSI2	AT2G43710	2	109	4.55281	Wilcoxon	1516	2.55933	Brown-Forsythe
AT5G67160	AT5G67160	5	119	4.5348	Wilcoxon	1646	2.51655	Brown-Forsythe
PEN2	AT2G44490	2	181	4.50298	Wilcoxon	1313	2.6287	Brown-Forsythe
AT1G31540	AT1G31540	1	192	4.45531	Wilcoxon	469	3.10452	Brown-Forsythe
AT4G19530	AT4G19530	4	223	4.37942	Wilcoxon	641	2.95146	Brown-Forsythe
AT4G13880	AT4G13880	4	259	4.30514	Wilcoxon	4009	2.08002	Brown-Forsythe
AT2G17050	AT2G17050	2	264	4.29739	Wilcoxon	7932	1.75247	Brown-Forsythe
AT2G17055	AT2G17055	2	266	4.2833	Wilcoxon	7932	1.75247	Brown-Forsythe
AT4G19530	AT4G19530	4	25	4.04952	EMMA	641	2.95146	Brown-Forsythe
AT5G46270	AT5G46270	5	54	3.64599	EMMA	311	3.28777	Brown-Forsythe
AT1G31540	AT1G31540	1	84	3.49207	EMMA	469	3.10452	Brown-Forsythe
AT5G44870	AT5G44870	5	91	3.47703	EMMA	9011	1.69118	Brown-Forsythe
AT5G35450	AT5G35450	5	97	3.44027	EMMA	8877	1.69867	Brown-Forsythe
AT4G38700	AT4G38700	4	104	3.38666	EMMA	99	3.86278	Brown-Forsythe
AT4G13880	AT4G13880	4	121	3.34218	EMMA	4009	2.08002	Brown-Forsythe
ST	AT2G03760	2	122	3.32311	EMMA	624	2.96126	Brown-Forsythe
AT5G27060	AT5G27060	5	123	3.31858	EMMA	664	2.94061	Brown-Forsythe

Supplementary Table 74 - GWA (M) and vGWA (V) scores and ranks of trait 272: Seedling Growth

Gene	Gene ID	Chr	Rank (M)	Score (M)	Test (M)	Rank (V)	Score (V)	Test (V)
TWD1	AT3G21640	3	7	4.49477	Wilcoxon	712	2.25889	Brown-Forsythe
ABH1	AT2G13540	2	56	3.47779	Wilcoxon	1377	1.99501	Brown-Forsythe
IAA7	AT3G23050	3	61	3.4256	Wilcoxon	814	2.21392	Brown-Forsythe
SHD	AT4G24190	4	62	3.41825	Wilcoxon	2544	1.74173	Brown-Forsythe
SLY1	AT4G24210	4	62	3.41825	Wilcoxon	2544	1.74173	Brown-Forsythe
ABA2	AT1G52340	1	79	3.3408	Wilcoxon	4934	1.49356	Brown-Forsythe
BIN4	AT5G24630	5	95	3.25878	Wilcoxon	4811	1.50275	Brown-Forsythe
SPT	AT4G36930	4	146	3.0556	Wilcoxon	23085	0.9083	Brown-Forsythe
CIP1	AT5G41790	5	173	3.00128	Wilcoxon	4741	1.50776	Brown-Forsythe
DAG1	AT3G61850	3	194	2.93296	Wilcoxon	707	2.26189	Brown-Forsythe
IAA7	AT3G23050	3	17	4.10221	EMMA	814	2.21392	Brown-Forsythe
CAND1	AT2G02560	2	19	4.0981	EMMA	199	2.79259	Brown-Forsythe
AGL31	AT5G65050	5	25	4.01706	EMMA	887	2.18008	Brown-Forsythe
NUA	AT1G79280	1	46	3.61854	EMMA	4569	1.52074	Brown-Forsythe
sim-to-LSH1	AT3G04510	3	56	3.54362	EMMA	2008	1.84016	Brown-Forsythe
LIP1	AT5G64813	5	58	3.52199	EMMA	11369	1.17483	Brown-Forsythe
BIN4	AT5G24630	5	62	3.4735	EMMA	4811	1.50275	Brown-Forsythe
ABA2	AT1G52340	1	64	3.47115	EMMA	4934	1.49356	Brown-Forsythe
ABH1	AT2G13540	2	66	3.46134	EMMA	1377	1.99501	Brown-Forsythe
SLY1	AT4G24210	4	69	3.45162	EMMA	2544	1.74173	Brown-Forsythe

Supplementary Table 75 - GWA (M) and vGWA (V) scores and ranks of trait 273: Vern Growth

Gene	Gene ID	Chr	Rank (M)	Score (M)	Test (M)	Rank (V)	Score (V)	Test (V)
SPT	AT4G36930	4	2	4.55017	Wilcoxon	78	3.76633	Brown-Forsythe
sim-to-DRM1	AT2G33830	2	23	3.51761	Wilcoxon	20879	1.06264	Brown-Forsythe
IMB1	AT2G34900	2	96	3.0418	Wilcoxon	3462	1.868	Brown-Forsythe
AT1G54070	AT1G54070	1	132	2.93044	Wilcoxon	347	3.01069	Brown-Forsythe
ABI1	AT4G26080	4	140	2.89996	Wilcoxon	1858	2.16679	Brown-Forsythe
EMS1	AT5G07280	5	153	2.8754	Wilcoxon	11126	1.34195	Brown-Forsythe
SPT	AT4G36930	4	1	6.87733	EMMA	78	3.76633	Brown-Forsythe
ELF8	AT2G06210	2	9	4.12721	EMMA	271	3.13137	Brown-Forsythe
EER5	AT2G19560	2	19	3.80692	EMMA	237	3.17178	Brown-Forsythe
TWD1	AT3G21640	3	37	3.64544	EMMA	14	4.55211	Brown-Forsythe
EIR1	AT5G57090	5	42	3.59043	EMMA	11	4.63103	Brown-Forsythe
YAP169	AT5G07200	5	50	3.50462	EMMA	10258	1.38057	Brown-Forsythe
ATS3	AT5G07190	5	50	3.50462	EMMA	10258	1.38057	Brown-Forsythe
AT1G54070	AT1G54070	1	60	3.4876	EMMA	347	3.01069	Brown-Forsythe
MSI1	AT5G58230	5	64	3.47149	EMMA	224	3.2008	Brown-Forsythe
ZFP1	AT1G80730	1	80	3.4151	EMMA	1421	2.3175	Brown-Forsythe

Supplementary Table 76 - GWA (M) and vGWA (V) scores and ranks of trait 274: After Vern Growth

Gene	Gene ID	Chr	Rank (M)	Score (M)	Test (M)	Rank (V)	Score (V)	Test (V)
ATS3	AT5G07190	5	7	4.11622	Wilcoxon	4233	1.64064	Brown-Forsythe
VIN3	AT5G57380	5	23	3.53972	Wilcoxon	4244	1.63846	Brown-Forsythe
CLV2	AT1G65380	1	29	3.44251	Wilcoxon	249	2.84795	Brown-Forsythe
GA4H	AT1G80340	1	30	3.44061	Wilcoxon	910	2.24929	Brown-Forsythe
FUS6	AT3G61140	3	36	3.36984	Wilcoxon	853	2.28131	Brown-Forsythe
SPT	AT4G36930	4	42	3.34915	Wilcoxon	15	4.03004	Brown-Forsythe
AHG2	AT1G55870	1	60	3.19901	Wilcoxon	1160	2.14298	Brown-Forsythe
ABA2	AT1G52340	1	64	3.18473	Wilcoxon	2167	1.9058	Brown-Forsythe
FUS3	AT3G26790	3	74	3.11408	Wilcoxon	1441	2.05853	Brown-Forsythe
CIP1	AT5G41790	5	114	2.93266	Wilcoxon	4386	1.6288	Brown-Forsythe
ATS3	AT5G07190	5	2	5.10822	EMMA	4233	1.64064	Brown-Forsythe
SPT	AT4G36930	4	4	4.77153	EMMA	15	4.03004	Brown-Forsythe
AP1	AT1G69120	1	15	3.88694	EMMA	1	5.84543	Brown-Forsythe
ELF8	AT2G06210	2	21	3.7115	EMMA	2130	1.913	Brown-Forsythe
ABA2	AT1G52340	1	26	3.65243	EMMA	2167	1.9058	Brown-Forsythe
AHG2	AT1G55870	1	35	3.58327	EMMA	1160	2.14298	Brown-Forsythe
FUS3	AT3G26790	3	40	3.54669	EMMA	1441	2.05853	Brown-Forsythe
APRR5	AT5G24470	5	48	3.45903	EMMA	1637	2.01339	Brown-Forsythe
SPA3	AT3G15354	3	53	3.35717	EMMA	237	2.86411	Brown-Forsythe
SPL5	AT3G15270	3	53	3.35717	EMMA	237	2.86411	Brown-Forsythe

Supplementary Table 77 - GWA (M) and vGWA (V) scores and ranks of trait 277: Secondary Dormancy

Gene	Gene ID	Chr	Rank (M)	Score (M)	Test (M)	Rank (V)	Score (V)	Test (V)
CUL4	AT5G46210	5	1	6.84223	Wilcoxon	478	4.05293	Brown-Forsythe
PIN5	AT5G16530	5	7	5.80704	Wilcoxon	120	5.11431	Brown-Forsythe
AT3G29970	AT3G29970	3	17	5.32313	Wilcoxon	300	4.45006	Brown-Forsythe
SMZ	AT3G54990	3	50	4.91147	Wilcoxon	1058	3.53925	Brown-Forsythe
RGA1	AT2G01570	2	68	4.79768	Wilcoxon	595	3.90128	Brown-Forsythe
VRN5	AT3G24440	3	74	4.73996	Wilcoxon	116	5.12407	Brown-Forsythe
MAF5	AT5G65080	5	113	4.5054	Wilcoxon	61	5.56908	Brown-Forsythe
EMS1	AT5G07280	5	163	4.33035	Wilcoxon	1138	3.49157	Brown-Forsythe
GA1	AT4G02780	4	195	4.24459	Wilcoxon	395	4.1927	Brown-Forsythe
CIPK3	AT2G26980	2	210	4.21064	Wilcoxon	1017	3.55579	Brown-Forsythe
GA4	AT1G15550	1	6	4.42936	EMMA	18155	1.59808	Brown-Forsythe
CUL4	AT5G46210	5	15	3.95779	EMMA	478	4.05293	Brown-Forsythe
CIPK3	AT2G26980	2	40	3.62182	EMMA	1017	3.55579	Brown-Forsythe
sim-to-FRL2	AT5G27230	5	58	3.47661	EMMA	2440	2.97076	Brown-Forsythe
AT1G56220	AT1G56220	1	78	3.37052	EMMA	2842	2.86873	Brown-Forsythe
SEPALLATA3	AT1G24260	1	89	3.32306	EMMA	6454	2.29785	Brown-Forsythe
HY5	AT5G11260	5	93	3.30501	EMMA	142	4.9614	Brown-Forsythe
VRN5	AT3G24440	3	96	3.29316	EMMA	116	5.12407	Brown-Forsythe
SPA1	AT2G46340	2	101	3.26845	EMMA	741	3.74326	Brown-Forsythe
AT3G29970	AT3G29970	3	110	3.2306	EMMA	300	4.45006	Brown-Forsythe

Supplementary Table 78 - GWA (M) and vGWA (V) scores and ranks of trait 278: Germ in dark

Gene	Gene ID	Chr	Rank (M)	Score (M)	Test (M)	Rank (V)	Score (V)	Test (V)
RCI2B	AT3G05890	3	18	5.25646	Wilcoxon	60	4.91711	Brown-Forsythe
VRN5	AT3G24440	3	42	4.80407	Wilcoxon	3660	2.44051	Brown-Forsythe
ELF8	AT2G06210	2	43	4.79027	Wilcoxon	2839	2.62626	Brown-Forsythe
GA1	AT4G02780	4	49	4.70829	Wilcoxon	4434	2.30181	Brown-Forsythe
SHP2	AT2G42830	2	52	4.67725	Wilcoxon	1157	3.25256	Brown-Forsythe
DFL2	AT4G03400	4	60	4.62212	Wilcoxon	3499	2.46945	Brown-Forsythe
TEL1	AT3G26120	3	81	4.45089	Wilcoxon	10633	1.72155	Brown-Forsythe
PIN3	AT1G70940	1	95	4.34536	Wilcoxon	3366	2.4998	Brown-Forsythe
HAB1	AT1G72770	1	121	4.19497	Wilcoxon	14555	1.5157	Brown-Forsythe
HAP2C	AT1G72830	1	121	4.19497	Wilcoxon	11985	1.63948	Brown-Forsythe
DWF1	AT3G19820	3	30	4.65885	EMMA	2474	2.72223	Brown-Forsythe
SHP2	AT2G42830	2	42	4.01552	EMMA	1157	3.25256	Brown-Forsythe
VRN5	AT3G24440	3	47	3.89569	EMMA	3660	2.44051	Brown-Forsythe
ELF4	AT2G40080	2	49	3.87479	EMMA	5823	2.1119	Brown-Forsythe
DFL2	AT4G03400	4	55	3.82547	EMMA	3499	2.46945	Brown-Forsythe
VIN3	AT5G57380	5	57	3.80704	EMMA	1531	3.06734	Brown-Forsythe
TEL1	AT3G26120	3	93	3.52172	EMMA	10633	1.72155	Brown-Forsythe
PIF3	AT1G09530	1	159	3.20315	EMMA	1876	2.92593	Brown-Forsythe
AT2G33830	AT2G33830	2	162	3.1966	EMMA	3651	2.44199	Brown-Forsythe
DOG1	AT5G45830	5	172	3.1607	EMMA	266	4.11905	Brown-Forsythe

Supplementary Table 79 - GWA (M) and vGWA (V) scores and ranks of trait 279: DSDS50

Gene	Gene ID	Chr	Rank (M)	Score (M)	Test (M)	Rank (V)	Score (V)	Test (V)
COMATOSE	AT4G39850	4	32	6.31068	Wilcoxon	6548	1.78083	Brown-Forsythe
ELF4	AT2G40080	2	53	6.00096	Wilcoxon	1824	2.45201	Brown-Forsythe
HBT	AT2G20000	2	62	5.92629	Wilcoxon	203	4.00536	Brown-Forsythe
CYCD4;1	AT5G65420	5	71	5.88552	Wilcoxon	2048	2.38593	Brown-Forsythe
DFL2	AT4G03400	4	107	5.65344	Wilcoxon	3526	2.10047	Brown-Forsythe
ATS3	AT5G07190	5	111	5.62377	Wilcoxon	3613	2.08875	Brown-Forsythe
EMS1	AT5G07280	5	151	5.46186	Wilcoxon	984	2.82227	Brown-Forsythe
ATMYB65	AT3G11440	3	156	5.46076	Wilcoxon	211	3.98381	Brown-Forsythe
ATRAB28	AT1G03120	1	170	5.37576	Wilcoxon	3925	2.04223	Brown-Forsythe
FLC	AT5G10140	5	190	5.2813	Wilcoxon	4829	1.94188	Brown-Forsythe
ELF4	AT2G40080	2	18	4.35971	EMMA	1824	2.45201	Brown-Forsythe
IAA6	AT1G52830	1	91	3.54974	EMMA	23	5.58665	Brown-Forsythe
PKL	AT2G25170	2	98	3.50137	EMMA	683	3.09265	Brown-Forsythe
HY2	AT3G09150	3	111	3.39456	EMMA	1152	2.73275	Brown-Forsythe
FRS1	AT4G19990	4	129	3.27598	EMMA	193	4.05025	Brown-Forsythe
COMATOSE	AT4G39850	4	158	3.15411	EMMA	6548	1.78083	Brown-Forsythe
DFL1	AT5G54510	5	173	3.12085	EMMA	4932	1.93257	Brown-Forsythe
UVI4	AT2G42260	2	188	3.07406	EMMA	5961	1.83217	Brown-Forsythe
DOG1	AT5G45830	5	197	3.04985	EMMA	4884	1.93836	Brown-Forsythe

Supplementary Table 80 - GWA (M) and vGWA (V) scores and ranks of trait 280: Seed bank 133-91

Gene	Gene ID	Chr	Rank (M)	Score (M)	Test (M)	Rank (V)	Score (V)	Test (V)
ARR3	AT1G59940	1	9	5.24948	Wilcoxon	11447	1.55733	Brown-Forsythe
GRV2	AT2G26890	2	15	4.92119	Wilcoxon	449	3.80359	Brown-Forsythe
COL9	AT3G07650	3	16	4.91378	Wilcoxon	6556	1.8833	Brown-Forsythe
GASA4	AT5G15230	5	21	4.81076	Wilcoxon	3905	2.20801	Brown-Forsythe
EPR1	AT1G18330	1	26	4.74926	Wilcoxon	2317	2.53716	Brown-Forsythe
ATGA2OX8	AT4G21200	4	48	4.50613	Wilcoxon	712	3.43567	Brown-Forsythe
EER5	AT2G19560	2	58	4.37476	Wilcoxon	12199	1.52606	Brown-Forsythe
SPK-2-2	AT3G50500	3	60	4.34696	Wilcoxon	60	4.34696	Brown-Forsythe
HY1	AT2G26670	2	69	4.30587	Wilcoxon	2281	2.54941	Brown-Forsythe
ELF5	AT5G62640	5	96	4.15025	Wilcoxon	2131	2.60209	Brown-Forsythe
AP1	AT1G69120	1	1	4.87018	EMMA	9574	1.6558	Brown-Forsythe
PHYD	AT4G16250	4	42	3.69464	EMMA	1673	2.7899	Brown-Forsythe
RGL3	AT5G17490	5	63	3.55835	EMMA	7690	1.78398	Brown-Forsythe
FVE	AT2G19520	2	76	3.48837	EMMA	10917	1.58187	Brown-Forsythe
VRN2	AT4G16845	4	89	3.42541	EMMA	247	4.36104	Brown-Forsythe
sim-to-NPH4	AT2G03070	2	146	3.21059	EMMA	1077	3.14519	Brown-Forsythe
ABI5	AT2G36270	2	149	3.20396	EMMA	69	5.90485	Brown-Forsythe
EER5	AT2G19560	2	155	3.19319	EMMA	12199	1.52606	Brown-Forsythe
AT4G21540	AT4G21540	4	160	3.1833	EMMA	9313	1.67212	Brown-Forsythe
DFL2	AT4G03400	4	161	3.18163	EMMA	2235	2.56563	Brown-Forsythe

Supplementary Table 81 - GWA (M) and vGWA (V) scores and ranks of trait 281: Storage 7 days

Gene	Gene ID	Chr	Rank (M)	Score (M)	Test (M)	Rank (V)	Score (V)	Test (V)
NCED9	AT1G78390	1	41	5.87448	Wilcoxon	38	10.40402	Brown-Forsythe
PHYB	AT2G18790	2	54	5.73754	Wilcoxon	1394	4.98396	Brown-Forsythe
SHY2	AT1G04240	1	132	5.47282	Wilcoxon	1384	4.98966	Brown-Forsythe
EMS1	AT5G07280	5	137	5.46925	Wilcoxon	3029	3.9432	Brown-Forsythe
COMATOSE	AT4G39850	4	142	5.4688	Wilcoxon	44	10.07424	Brown-Forsythe
LKP2	AT2G18915	2	154	5.40765	Wilcoxon	599	6.21166	Brown-Forsythe
ZFP1	AT1G80730	1	155	5.40389	Wilcoxon	2551	4.18783	Brown-Forsythe
DFL2	AT4G03400	4	157	5.39549	Wilcoxon	3290	3.83806	Brown-Forsythe
ATS3	AT5G07190	5	164	5.35569	Wilcoxon	225	7.54102	Brown-Forsythe
ABA1	AT5G67030	5	173	5.31955	Wilcoxon	5275	3.25846	Brown-Forsythe
NCED9	AT1G78390	1	32	4.66173	EMMA	38	10.40402	Brown-Forsythe
EMF1	AT5G11530	5	33	4.65738	EMMA	32	10.73674	Brown-Forsythe
VRN5	AT3G24440	3	34	4.60894	EMMA	833	5.7713	Brown-Forsythe
FY	AT5G13480	5	61	4.21009	EMMA	480	6.43196	Brown-Forsythe
HYL1	AT1G09700	1	63	4.18653	EMMA	3	15.14327	Brown-Forsythe
FLC	AT5G10140	5	66	4.15523	EMMA	3609	3.73368	Brown-Forsythe
TFL1	AT5G03840	5	134	3.73271	EMMA	1658	4.73216	Brown-Forsythe
COMATOSE	AT4G39850	4	177	3.55875	EMMA	44	10.07424	Brown-Forsythe
AT5G44300	AT5G44300	5	232	3.39237	EMMA	11852	2.35309	Brown-Forsythe
DOG1	AT5G45830	5	367	3.07196	EMMA	2011	4.49863	Brown-Forsythe

Supplementary Table 82 - GWA (M) and vGWA (V) scores and ranks of trait 282: Storage 28 days

Gene	Gene ID	Chr	Rank (M)	Score (M)	Test (M)	Rank (V)	Score (V)	Test (V)
COMATOSE	AT4G39850	4	21	6.20501	Wilcoxon	36	7.2343	Brown-Forsythe
ATMYB65	AT3G11440	3	23	6.17584	Wilcoxon	2275	3.53521	Brown-Forsythe
SPA4	AT1G53090	1	26	6.10511	Wilcoxon	1113	4.21423	Brown-Forsythe
ELF4	AT2G40080	2	37	6.01469	Wilcoxon	321	5.32299	Brown-Forsythe
ATGID1B	AT3G63010	3	38	6.00612	Wilcoxon	168	5.88431	Brown-Forsythe
AGL15	AT5G13790	5	41	5.9587	Wilcoxon	1239	4.10387	Brown-Forsythe
IMB1	AT2G34900	2	56	5.82535	Wilcoxon	2334	3.5106	Brown-Forsythe
DFL2	AT4G03400	4	70	5.7262	Wilcoxon	9465	2.38169	Brown-Forsythe
PGI1	AT4G24620	4	75	5.69726	Wilcoxon	19	8.20662	Brown-Forsythe
EMS1	AT5G07280	5	100	5.62778	Wilcoxon	1060	4.25541	Brown-Forsythe
FLC	AT5G10140	5	2	5.89834	EMMA	17	8.30546	Brown-Forsythe
ACT7	AT5G09810	5	15	4.46862	EMMA	191	5.69628	Brown-Forsythe
ZFP1	AT1G80730	1	17	4.37073	EMMA	261	5.48581	Brown-Forsythe
IMB1	AT2G34900	2	50	3.80453	EMMA	2334	3.5106	Brown-Forsythe
AT5G01560	AT5G01560	5	52	3.78542	EMMA	516	4.97408	Brown-Forsythe
NCED9	AT1G78390	1	79	3.52273	EMMA	2688	3.39209	Brown-Forsythe
GIL1	AT5G58960	5	87	3.47038	EMMA	1972	3.66482	Brown-Forsythe
ELF5	AT5G62640	5	109	3.36139	EMMA	2356	3.50021	Brown-Forsythe
PKS2	AT1G14280	1	149	3.20885	EMMA	4163	3.02765	Brown-Forsythe
PSD	AT1G72560	1	153	3.20207	EMMA	25	7.65891	Brown-Forsythe

Supplementary Table 83 - GWA (M) and vGWA (V) scores and ranks of trait 283: Storage 56 days

Gene	Gene ID	Chr	Rank (M)	Score (M)	Test (M)	Rank (V)	Score (V)	Test (V)
HBT	AT2G20000	2	31	6.39096	Wilcoxon	2634	4.12965	Brown-Forsythe
FLC	AT5G10140	5	33	6.29298	Wilcoxon	965	5.58238	Brown-Forsythe
AGL15	AT5G13790	5	46	5.95613	Wilcoxon	15	9.57402	Brown-Forsythe
ATGID1B	AT3G63010	3	60	5.76202	Wilcoxon	74	8.34573	Brown-Forsythe
COMATOSE	AT4G39850	4	62	5.73333	Wilcoxon	39	8.97004	Brown-Forsythe
ATMYB65	AT3G11440	3	67	5.69039	Wilcoxon	304	7.22187	Brown-Forsythe
IMB1	AT2G34900	2	77	5.57797	Wilcoxon	815	5.92912	Brown-Forsythe
ELF4	AT2G40080	2	86	5.56102	Wilcoxon	2	10.36637	Brown-Forsythe
AGL20	AT2G45660	2	94	5.50112	Wilcoxon	1220	5.26641	Brown-Forsythe
ATS3	AT5G07190	5	95	5.49346	Wilcoxon	24	9.10971	Brown-Forsythe
FLC	AT5G10140	5	8	4.37042	EMMA	965	5.58238	Brown-Forsythe
LBA1	AT5G47010	5	16	4.15566	EMMA	7427	2.75259	Brown-Forsythe
ZFP1	AT1G80730	1	22	4.05406	EMMA	791	5.98748	Brown-Forsythe
DDF1	AT1G12610	1	29	3.95268	EMMA	4827	3.31738	Brown-Forsythe
KNAT3	AT5G25220	5	37	3.84108	EMMA	1901	4.54614	Brown-Forsythe
PKL	AT2G25170	2	41	3.80086	EMMA	6852	2.85854	Brown-Forsythe
GA1	AT4G02780	4	67	3.51887	EMMA	2049	4.49337	Brown-Forsythe
IAA6	AT1G52830	1	76	3.42835	EMMA	443	6.7722	Brown-Forsythe
HBT	AT2G20000	2	79	3.40602	EMMA	2634	4.12965	Brown-Forsythe
COL9	AT3G07650	3	88	3.36822	EMMA	1993	4.52936	Brown-Forsythe

A Brief Tutorial of the R Package vGWAS

Setup

To use the **vGWAS** package, of course, an R environment is required. Visit:

http://www.r-project.org

and install R for the operating system.

Start R and in the R console, type the following command to install the package:

```
install.packages('vGWAS', repos = 'http://r-forge.r-project.org')
```

If everything works fine, something like the following should show:

```
trying URL ...
Content type ... length ... bytes (... Kb)
opened URL
==================================================
downloaded ... Kb
```

Now the package is installed in the R library.

Example

In the R console, the command:

```
vignette('vGWAS')
```

opens this PDF document together with the package documentation. An example can be found in the documentation. Type:

```
require(vGWAS)
```

to load the package (two depended packages **hglm** and **dglm** are required to be installed as well), then four main functions in the package are ready to use - `brown.forsythe.test`, `vGWAS`, `vGWAS.heva`, `plot` (S3 method for `vGWAS` object) and `vGWAS.variance`. Run the following commands to load the example data:

```
data(pheno)
data(geno)
data(chr)
data(map)
```

`pheno` is a numeric vector of the simulated phenotypic values. By running:

```
hist(pheno, breaks = 30, density = 15, col = 'darkred')
```

a histogram of the phenotype distribution is produced as Figure 1.

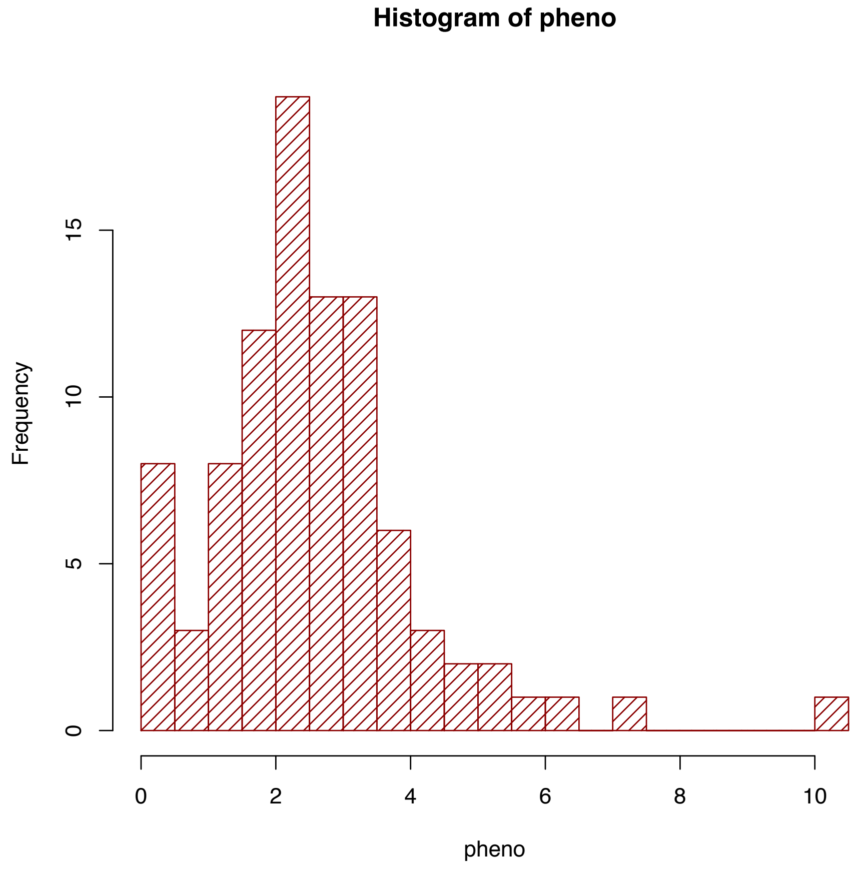

Figure 1: Histogram of the simulated phenotype.

The command:

```
table(chr)
```

produces:

```
chr
   1    2    3    4    5
5000 3000 4000 2000 6000
```

This shows exactly the number of markers on each of the five simulated chromosomes. Now, the *objects* loaded in R are ready for a vGWA scan, which can be done using the single command:

```
vgwa <- vGWAS(phenotype = pheno, geno.matrix = geno, marker.map = map,
              chr.index = chr)
```

A progress bar will show to indicate the progress of the scan, such like:

```
|===                                                                  |   4%
```

When the scan is finished, all the output statistics will be returned as a *list* into the object `vgwa`, which belongs to the *class* 'vGWAS'. Any object that has a *structure* belonging to class 'vGWAS' can be directly passed into S3 method function `plot`. For instance, simply run the following command, we can plot the results in `vgwa`:

```
plot(vgwa)
```

which produces Figure 2. There is a clear peak above the Bonferroni corrected threshold (dashed orange line).

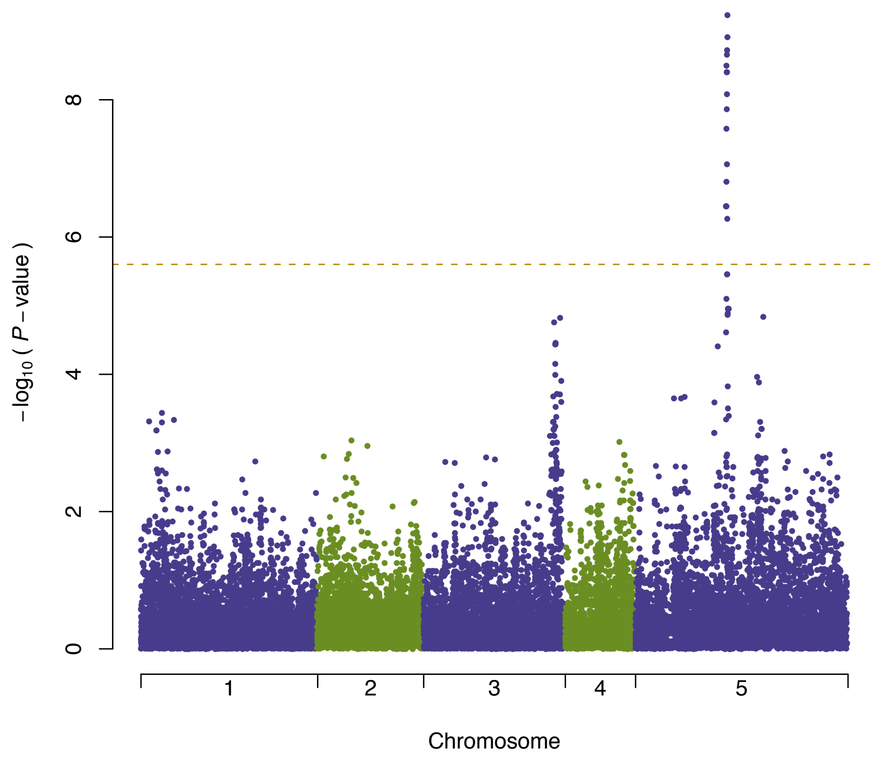

Figure 2: vGWAS results of the simulated data.

Regarding the marker that gave the highest score, the heritability explained by the mean and variance can be split and calculated via:

```
vGWAS.variance(phenotype = pheno,
                   marker.genotype = geno[,vgwa$p.value == min(vgwa$p.value)])
```

which prints out:

```
variance explained by the mean part of model:
4.2 %, p-value = 0.1930359
variance explained by the variance part of model:
```

```
23.06 %, p-value = 7.922846e-12
variance explained in total:
27.26 %
```

The output can also be stored if assigning the function call to an object.

To correct for population confounding, the package is capable of applying genomic control to the obtained *p*-values. For instance, one can simply run:

```
vgwa2 <- vGWAS.gc(vgwa)
```

By default, this command takes all the obtained *p*-values for genomic control and plots the QQ plot as shown in Figure 3.

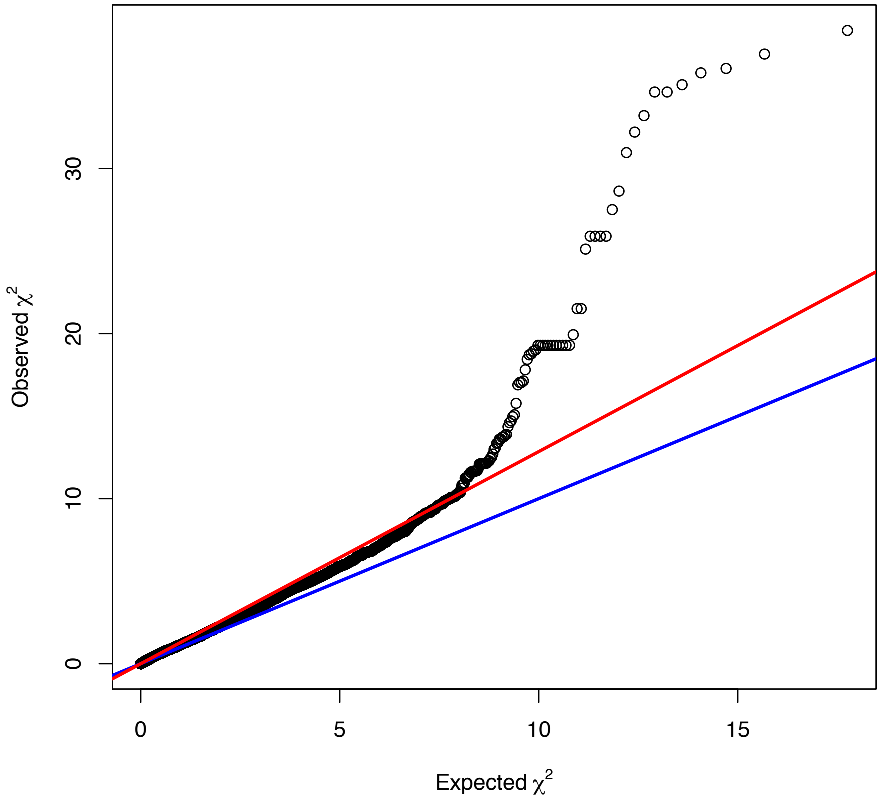

Figure 3: QQ plot for genomic control.

The estimated inflation ratio and its standard error are:

```
vgwa2$lambda
[1] 1.285152
vgwa2$lambda.se
[1] 0.002133788
```

Plotting the this object as follows will generate a new vGWAS plot in Figure 4.

```
plot(vgwa2, col.manhattan = c('darkmagenta', 'darkcyan'))
```

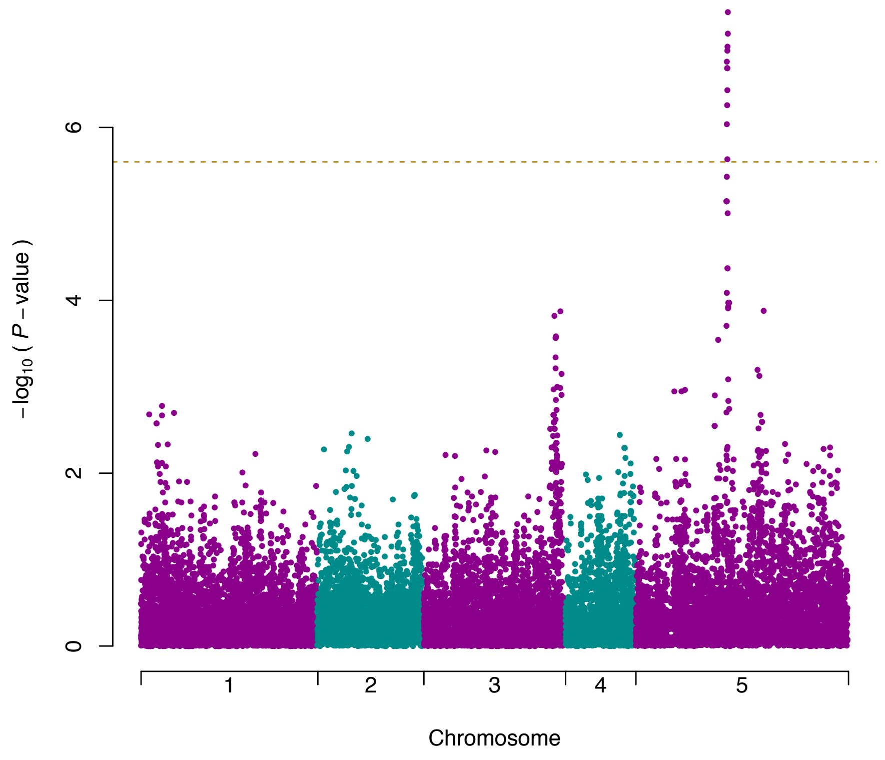

Figure 4: vGWAS results of the simulated data after genomic control.

Remarks

The package source and further development information are on the R-Forge project page: https://r-forge.r-project.org/projects/vgwas/

Package ‘vGWAS’

July 29, 2011

Type Package

Title Variance Genome-wide Association

Version 2011.07.25

Date 2011-07-25

Author Xia Shen

Maintainer Xia Shen <xia.shen@icm.uu.se>

Description The package provides models and tests for variance genome-wide association study (vGWAS).

License GPL

LazyLoad yes

Depends hglm, dglm

R topics documented:

vGWAS-package 2
brown.forsythe.test 3
chr 5
geno 5
map 6
pheno 6
plot.vGWAS 7
vGWAS 8
vGWAS.gc 10
vGWAS.variance 11

Index **13**

`vGWAS-package` *Variance Genome-wide Association*

Description

The package provides models and tests for variance genome-wide association study (vGWAS).

Details

Package:	vGWAS
Type:	Package
Version:	2011.07.25
Date:	2011-07-25
License:	GPL
LazyLoad:	yes
Depends:	hglm, dglm

Author(s)

Xia Shen

Maintainer: Xia Shen <xia.shen@icm.uu.se>

References

Shen, X., Pettersson, M., Ronnegard, L. and Carlborg, O. (2011): **Inheritance beyond plain heritability: variance-controlling genes in *Arabidopsis thaliana***. *Submitted*.

Ronnegard, L., Shen, X. and Alam, M. (2011): **hglm: A Package for Fitting Hierarchical Generalized Linear Models**. *The R Journal*, **2**(2), 20-28.

Brown, M. B. and Forsythe, A.B. (1974). **Robust tests for equality of variances.** *Journal of the American Statistical Association*, **69**, 364-367.

Levene, H. (1960). **Robust Tests for Equality of Variances**, *in Contributions to Probability and Statistics*, ed. I. Olkin, Palo Alto, CA: Stanford Univ. Press.

See Also

R package `lawstat` for other types of nonparametric variance tests.

`brown.forsythe.test` *Brown-Forsythe's Test of Equality of Variances*

Description

The function performs the robust Brown-Forsythe test using the group medians. Instead of the ANOVA statistic, the Kruskal-Wallis ANOVA may also be applied using this function.

Usage

```
brown.forsythe.test(y, group, kruskal.test=FALSE)
```

Arguments

`y`	a numeric vector of data values.
`group`	factor of the data.
`kruskal.test`	a `logical` value specifying whether to use Kruskal-Wallis statistic. The default option is `FALSE`, i.e., the usual ANOVA statistic is used in place of Kruskal-Wallis statistic.

Details

Levene (1960) proposed a test for homogeneity of variances in k groups which is based on the ANOVA statistic applied to absolute deviations of observations from the corresponding group mean. The robust Brown-Forsythe version of the Levene-type test substites the group mean by the group median in the classical Levene statistic.

Value

A list with the following numeric components.

`statistic`	the value of the test statistic.
`p.value`	the p-value of the test.
`method`	type of test performed.
`data.name`	a character string giving the name of the data.

Acknowledgement

The authors of package `lawstat` is ackownledged for their source code under free GPL license.

Note

Modified from the `lawstat` package.

Author(s)

Xia Shen

References

Brown, M. B. and Forsythe, A.B. (1974). **Robust tests for equality of variances.** *Journal of the American Statistical Association*, **69**, 364-367.

Levene, H. (1960). **Robust Tests for Equality of Variances**, *in Contributions to Probability and Statistics*, ed. I. Olkin, Palo Alto, CA: Stanford Univ. Press.

Examples

```
## Not run:

data(pheno)
data(geno)
brown.forsythe.test(pheno, geno[,911])


## End(Not run)
```

chr *Chromosome Indices for The Markers of The Simulated Data*

Description

Chromosome indices for the markers of the simulated data

Usage

```
data(chr)
```

Format

A numeric vector of chromosome indices for the 20K simulated markers.

Examples

```
data(chr)
table(chr)
```

geno *The Marker Genotypes of The Simulated Data*

Description

The marker genotypes of the simulated data

Usage

```
data(geno)
```

Format

A character matrix of size (number of individuals) times (number of markers in the genome).

Details

Note that there is only one column for each marker.

Examples

```
data(geno)
```

`map` *Map Positions for The Markers of The Simulated Data*

Description

Map positions for the markers of the simulated data

Usage

```
data(chr)
```

Format

A numeric vector of chromosomal map positions of the 20K simulated markers.

Examples

```
data(map)
```

`pheno` *Phenotypic Values for The Markers of The Simulated Data*

Description

Phenotypic values for the markers of the simulated data

Usage

```
data(pheno)
```

Format

A numeric vector of the phenotypic values of 93 simulated individuals.

Examples

```
data(pheno)
hist(pheno, breaks = 30)
```

`plot.vGWAS` *Variance GWA Manhattan Plot*

Description

The function plots the variance GWA result for the giving scan object.

Usage

```
## S3 method for class 'vGWAS'
plot(x, sig.threshold = NULL, low.log.p = 0, pch = 16,
           cex = 0.6, col.manhattan = c("slateblue4", "olivedrab"),
           col.sig.threshold = "darkgoldenrod", ...)
```

Arguments

- `x` — a result object from `vGWAS` scan. It can be any `list` or `data.frame` that contains `chromosome`, `marker.map`, and `p.value`, with `class = 'vGWAS'`. See `vGWAS`.
- `sig.threshold` — a numeric value giving the significance threshold for `-log(pvalues, 10)`. If `NULL`, Bonferroni correction will be used.
- `low.log.p` — a numeric value giving the lower limit of the `-log(pvalues, 10)` to plot.
- `pch` — point character. See `par`.
- `cex` — size of points. See `par`.
- `col.manhattan` — two colors as a vector for the Manhattan plot.
- `col.sig.threshold` — one color for the significance threshold.
- `...` — not used.

Value

a plot for viewing vGWAS result.

Author(s)

Xia Shen

References

Shen, X., Pettersson, M., Ronnegard, L. and Carlborg, O. (2011): **Inheritance beyond plain heritability: variance-controlling genes in *Arabidopsis thaliana***. *Submitted.*

See Also

`vGWAS-package`, `vGWAS`

Examples

```
## Not run:

# ----- load data ----- #

data(pheno)
data(geno)
data(chr)
data(map)

# ----- variance GWA scan ----- #

vgwa <- vGWAS(phenotype = pheno, geno.matrix = geno,
              marker.map = map, chr.index = chr)

# ----- visualize the scan ----- #

plot(vgwa)

# ----- calculate the variance explained by strongest the marker ----- #

vGWAS.heritability(phenotype = pheno,
                   marker.genotype = geno[,vgwa$p.value == min(vgwa$p.value)])

# ----- genomic control ----- #

vgwa2 <- vGWAS.gc(vgwa)

plot(vgwa2)

## End(Not run)
```

`vGWAS` *Variance Genome-wide Association*

Description

Variance Genome-wide association for using nonparametric variance test

Usage

```
vGWAS(phenotype, geno.matrix, kruskal.test = FALSE,
      marker.map = NULL, chr.index = NULL)
```

Arguments

`phenotype`	a `numeric` or `logical` vector of the phenotyic values. See **Examples**.
`geno.matrix`	a `matrix` or `data.frame` with individuals as rows and markers as columns. The marker genotypes for each marker are coded as one column. See **Examples**.
`kruskal.test`	a `logical` value specifying whether to use Kruskal-Wallis statistic. The default option is `FALSE`, i.e., the usual ANOVA statistic is used in place of Kruskal-Wallis statistic.

`marker.map` a `numeric` vector giving the marker map positions for each chromosome. See **Examples**.

`chr.index` a `numeric` vector giving the chromosome index for each marker. See **Examples**.

Value

a `data.frame` containing columns of `marker` names, `chromosome` indices, `marker.map` positions, test `statistic` values, and `p.value` for each position.

Author(s)

Xia Shen

References

Shen, X., Pettersson, M., Ronnegard, L. and Carlborg, O. (2011): **Inheritance beyond plain heritability: variance-controlling genes in *Arabidopsis thaliana***. *Submitted*.

Ronnegard, L., Shen, X. and Alam, M. (2011): **hglm: A Package for Fitting Hierarchical Generalized Linear Models**. *The R Journal*, **2**(2), 20-28.

See Also

`vGWAS-package`

Examples

```
## Not run:

# ----- load data ----- #

data(pheno)
data(geno)
data(chr)
data(map)

# ----- variance GWA scan ----- #

vgwa <- vGWAS(phenotype = pheno, geno.matrix = geno,
              marker.map = map, chr.index = chr)

# ----- visualize the scan ----- #

plot(vgwa)

# ----- calculate the variance explained by strongest the marker ----- #

vGWAS.variance(phenotype = pheno,
                   marker.genotype = geno[,vgwa$p.value == min(vgwa$p.value)])

# ----- genomic control ----- #

vgwa2 <- vGWAS.gc(vgwa)
```

```
plot(vgwa2)

## End(Not run)
```

---

vGWAS.gc *Genomic Control for vGWAS*

Description

The function does genomic control for the variance GWA result object.

Usage

```
vGWAS.gc(object, plot = TRUE, proportion = 1, ...)
```

Arguments

object	a result object from `vGWAS` scan. It can be any `list` or `data.frame` that contains `p.value`. See `vGWAS`.
plot	a logical value turning on/off the QQ plot for genomic control.
proportion	a numeric value between 0 and 1 giving the proportion of obtained p-values to be used for genomic control.
...	not used.

Value

lambda	estimated inflation ratio.
lambda.se	standard error of the estimated inflation ratio.
gc.p.value	p-values after genomic control.

Author(s)

Xia Shen

References

Shen, X., Pettersson, M., Ronnegard, L. and Carlborg, O. (2011): **Inheritance beyond plain heritability: variance-controlling genes in *Arabidopsis thaliana***. *Submitted.*

See Also

`vGWAS-package`, `vGWAS`

Examples

```
## Not run:

# ----- load data ----- #

data(pheno)
data(geno)
data(chr)
data(map)

# ----- variance GWA scan ----- #

vgwa <- vGWAS(phenotype = pheno, geno.matrix = geno,
              marker.map = map, chr.index = chr)

# ----- visualize the scan ----- #

plot(vgwa)

# ----- genomic control ----- #

vgwa2 <- vGWAS.gc(vgwa)

plot(vgwa2)

## End(Not run)
```

`vGWAS.variance` *Calculating Variance Explained by A Single Marker*

Description

The function calculates and reports the variance explained for a single marker by fitting a double generalized linear model. It gives both the variance explained by the mean and variance parts of model.

Usage

```
vGWAS.variance(phenotype, marker.genotype, print = TRUE)
```

Arguments

`phenotype`	a `numeric` vector of the phenotyic values. See **Examples**.
`marker.genotype`	a `numeric` or `character` or `factor` vector of the genotypes of a single marker. See **Examples**.
`print`	a `logical` value. If `FALSE`, the heritability values will be returned for storage.

Details

The **Value** will only be available if `only.print = FALSE`.

Value

`variance.mean`
: the variance explained by the mean part of model.

`variance.disp`
: the variance explained by the variance part of model.

Author(s)

Xia Shen

References

Shen, X., Pettersson, M., Ronnegard, L. and Carlborg, O. (2011): **Inheritance beyond plain heritability: variance-controlling genes in *Arabidopsis thaliana***. *Submitted.*

See Also

`vGWAS-package`, `vGWAS`, `plot.vGWAS`

Examples

```
## Not run:

# ----- load data ----- #

data(pheno)
data(geno)
data(chr)
data(map)

# ----- variance GWA scan ----- #

vgwa <- vGWAS(phenotype = pheno, geno.matrix = geno,
              marker.map = map, chr.index = chr)

# ----- visualize the scan ----- #

plot(vgwa)

# ----- calculate the variance explained by strongest the marker ----- #

vGWAS.variance(phenotype = pheno,
                   marker.genotype = geno[,vgwa$p.value == min(vgwa$p.value)])


## End(Not run)
```

Index

∗Topic **GWAS**
vGWAS-package, 2
∗Topic **datasets**
pheno, 6
∗Topic **htest**
brown.forsythe.test, 3

brown.forsythe.test, 3

chr, 5

geno, 5

map, 6

par, *7*
pheno, 6
plot.vGWAS, 7, *12*

vGWAS, *7*, 8, *10*, *12*
vGWAS-package, *7*, *9*, *10*, *12*
vGWAS-package, 2
vGWAS.gc, 10
vGWAS.variance, 11
